# Stereoretentive Norrish–Yang Photocyclization Mediated by Hydrogen Bonding

**DOI:** 10.1021/jacs.6c09924

**Published:** 2026-06-26

**Authors:** Enrico Sfreddo, Aleksa Durdevic, Andrea Palone, Tristan von Münchow, Nunzio Matera, Andrea Mazzanti, Paolo Melchiorre

**Affiliations:** University of Bologna, Department of Industrial Chemistry Toso Montanari, via Piero Gobetti 85, 40129 Bologna, Italy

## Abstract

The Norrish–Yang photocyclization of aryl alkyl ketones is a classical photochemical transformation that proceeds through triplet 1,4-diradicals whose conformations govern the fate of the reaction. Directing the reactivity of these diradicals is difficult because they can evolve through competing pathways, including cyclization and Norrish type II fragmentation. Achieving stereocontrol is even more challenging because radicals generated from enantiopure substrates are expected to undergo rapid configurational scrambling. Here we report a stereoretentive Norrish–Yang photocyclization of enantiopure aryl alkyl ketones enabled by hydrogen-bond-guided conformational control of the photogenerated 1,4-diradical. Hydrogen-bonding units encoded within the chiral substrate bias the reactive diradical manifold toward cyclization while suppressing fragmentation and stereochemical erosion. The reaction affords highly enantioenriched spirocyclobutanol-containing amines and related cyclobutanols bearing two stereocenters, with high enantiospecificity across a range of amino-acid-derived substrates.

## Graphical abstract

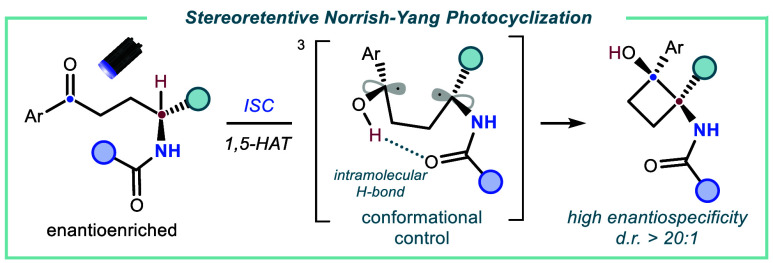

Carbon radicals are powerful intermediates in synthesis,^1^ yet they are stereochemically labile because open-shell centers can undergo rapid configurational scrambling (Figure 1a).^2^ Consequently, stereospecific radical transformations in solution remain difficult^3^ unless the radical is captured before stereochemical erosion or constrained within an organized environment. Recent examples in metal-bound coupling pathways^4^ and enzymatic active sites^5^ suggest that stereochemical information can be preserved and transmitted when transient open-shell intermediates are conformationally restricted.

**Figure 1:**
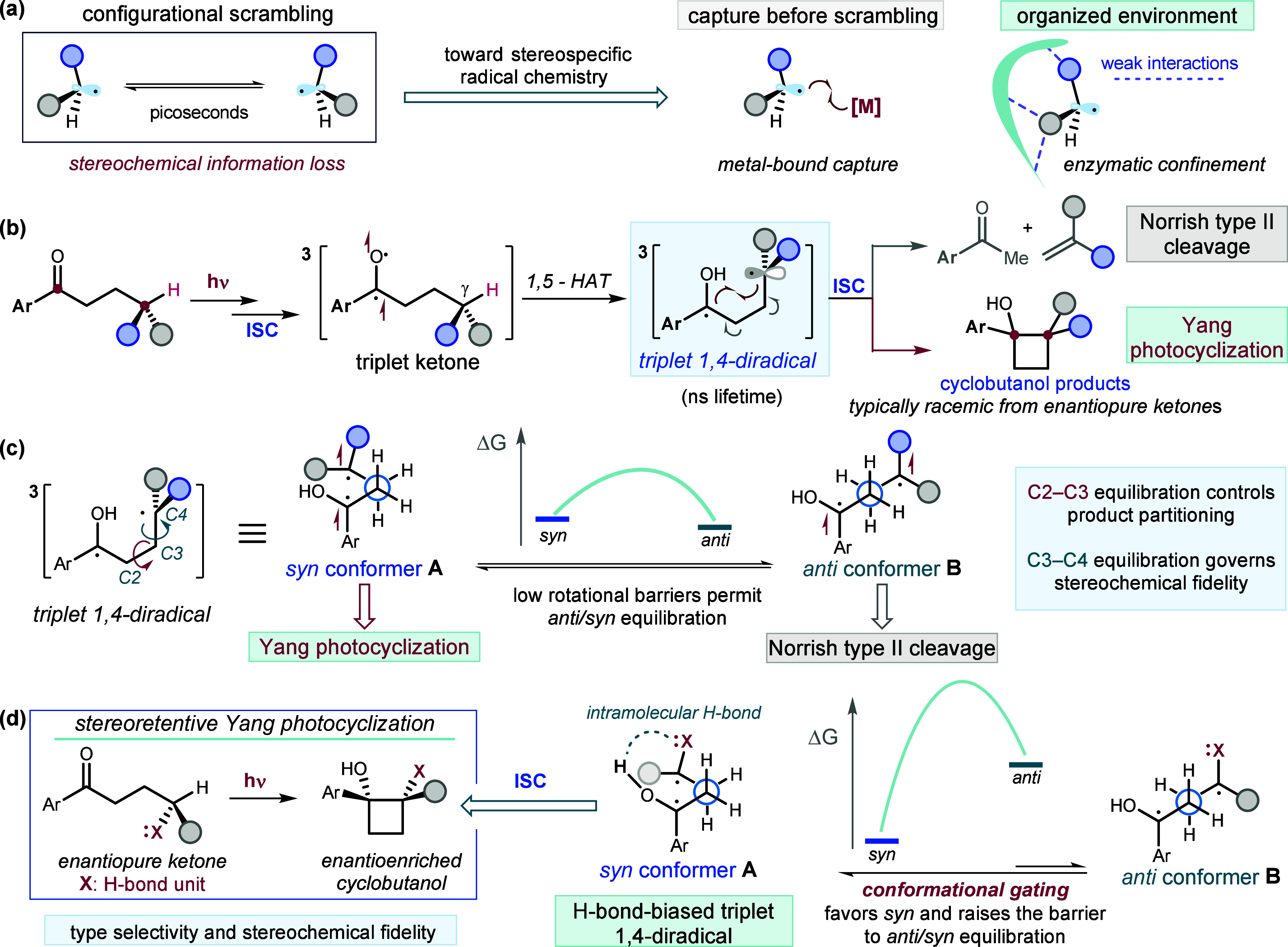
Hydrogen-bond-guided conformational control in stereoretentive Norrish–Yang photocyclization. (**a**) Open-shell intermediates undergo rapid configurational scrambling unless rapidly captured^4^ or constrained within organized environments.^5^ (**b**) Classical Norrish–Yang manifold: triplet 1,4-diradicals partition between Yang cyclization and Norrish type II cleavage. (**c**) Conformation-dependent branching: *syn* conformers **A** favor cyclization, whereas *anti* conformers **B** favor fragmentation. (**d**) Design principle: an intramolecular H-bonding unit biases the triplet 1,4-diradical toward productive *syn* conformers and disfavors pathways leading to fragmentation or stereochemical erosion.

Photochemical reactions provide a demanding test of this principle, as photoexcitation generates radicals whose evolution can be conformation-sensitive. The Norrish–Yang photocyclization^6^ offers a paradigmatic example. Following excitation and intersystem crossing (ISC), aryl alkyl ketones bearing a γ-hydrogen populate the triplet state and undergo intramolecular 1,5-hydrogen atom transfer (HAT) to form triplet-derived 1,4-diradicals,^7^ which can either cyclize to afford Yang cyclobutanol products^8a^ or fragment through Norrish type II cleavage (Figure 1b).^8b^ Both type selectivity,^9^ that is, partitioning of the 1,4-diradical between cyclization and fragmentation, and stereocontrol remain difficult to achieve. This is because the triplet 1,4-diradical persists on the nanosecond time scale,^10^ allowing conformational exchange and racemization before product formation.^2^

Classical mechanistic studies established that product partitioning is conformation-dependent.^11^ In particular, *syn* conformers of the 1,4-diradical (**A**, Figure 1c) are predisposed toward cyclization to Yang products, whereas *anti* conformers **B** preferentially evolve toward fragmentation. Earlier studies^12^ showed that the cyclization/fragmentation ratio can be tuned through substrate-induced conformational bias: varying the substitution pattern within the aliphatic moiety of aryl ketones shifts the relative stability of *syn* and *anti* triplet 1,4-diradicals, favoring different reaction channels. In specially engineered substrates, hydrogen-bonding interactions were also shown to influence this partitioning.^13^ Solid-state studies showed that crystal packing can impose specific reactive conformations and impart stereoselectivity to the Yang photocyclization.^14^ These findings establish conformational bias not only as a determinant of product partitioning, but as a potential element of stereochemical control. However, extending this level of conformational control to a general stereospecific strategy in solution-phase Norrish–Yang photochemistry has not been achieved. This unresolved problem is particularly striking because asymmetric Yang photocyclizations have remained limited to solid-state reactions^14^ or stoichiometric chiral complexing-agent approaches.^15^

We reasoned that this conformational-bias principle could be engineered directly into aromatic ketones by programming a hydrogen-bonding network into an enantiopure substrate (Figure 1d). Such a design is expected to bias the conformational landscape of the photogenerated triplet 1,4-diradical, not only tuning type selectivity but also limiting rotational equilibration and stereochemical scrambling. According to Scaiano’s conformational-memory framework,^16^ ISC from this preorganized geometry followed by rapid cyclization within the ensuing singlet manifold should retain the geometry encoded in the precursor triplet conformer.^17^ As a result, an enantiopure ketone could furnish a highly enantioenriched cyclobutanol while also controlling product partitioning. Giese reported relevant precedents for memory of chirality in the photocyclization of an oxoester under singlet-state conditions^18a^ and related peptide-derived ketones,^18b^ although in a limited number of selected substrates. A subsequent computational study supported the involvement of conformationally restricted diradicals and postulated hydrogen bonding involving the ketyl radical.^18c^ In a complementary approach, Sivaguru demonstrated axial-to-point chirality transfer in the Norrish–Yang reaction of atropchiral α-oxoamides.^18d^ Despite these precedents, a general stereospecific solution for classical triplet-state aromatic-ketone Norrish–Yang chemistry has remained elusive.

Here we show the realization of this design principle in readily available enantiopure aryl alkyl ketones bearing a suitably positioned H-bonding unit: intramolecular hydrogen-bond interactions impose conformational control on photogenerated triplet 1,4-diradicals, enabling stereoretentive Norrish–Yang photocyclization in solution while suppressing fragmentation. The process delivers highly enantioenriched cyclobutanols, including spirocyclobutane-containing amines bearing contiguous stereocenters.^19^

We started our studies by selecting chiral phenyl ketone (*S*)-**1a** as a model substrate (Figure 2a), readily accessible in enantiopure form from *N*-Boc-l-proline in 4 steps and 83% overall yield. Substrate **1a** was designed to contain an *N*-Boc group, whose carbamate oxygen was expected to enable intramolecular hydrogen bonding within the key 1,4-diradical. Irradiation of (*S*)-**1a** in a 1:1 CH_2_Cl_2_/pentane mixture with violet light (LED, λ_max_ = 370 nm) at 30 °C efficiently delivered the spirocyclobutanol product **2a** (entry 1). However, **2a** was obtained as a racemate, and the Yang cyclization showed moderate selectivity over Norrish type II cleavage (5:1 ratio of **2a** to the fragmentation product **3**). Lowering the temperature to −40 °C led to the emergence of partial enantiospecificity (e.s.) (57% e.s., **2a** formed with 77:23 enantiomeric ratio, e.r.) together with a slight improvement in product partitioning (6:1 product ratio, entry 2). Further cooling to −90 °C increased the enantiospecificity to 97% e.s. and the cyclization/fragmentation ratio to >20:1 (entry 3). This high degree of stereochemical fidelity and product partitioning was achieved without loss of efficiency, as **2a** was isolated in 86% yield and 98:2 e.r. after 3 h. Under these conditions, only a single diastereomer was observed, providing access to a spirocyclobutanol bearing two stereocenters. The absolute configuration of compound (1*S*,4*S*)-**4**, obtained upon Boc deprotection of **2a**, was determined by X-ray crystallographic analysis,^20^ establishing the stereoretentive nature of the Yang photocyclization (Figure 2b).

**Figure 2:**
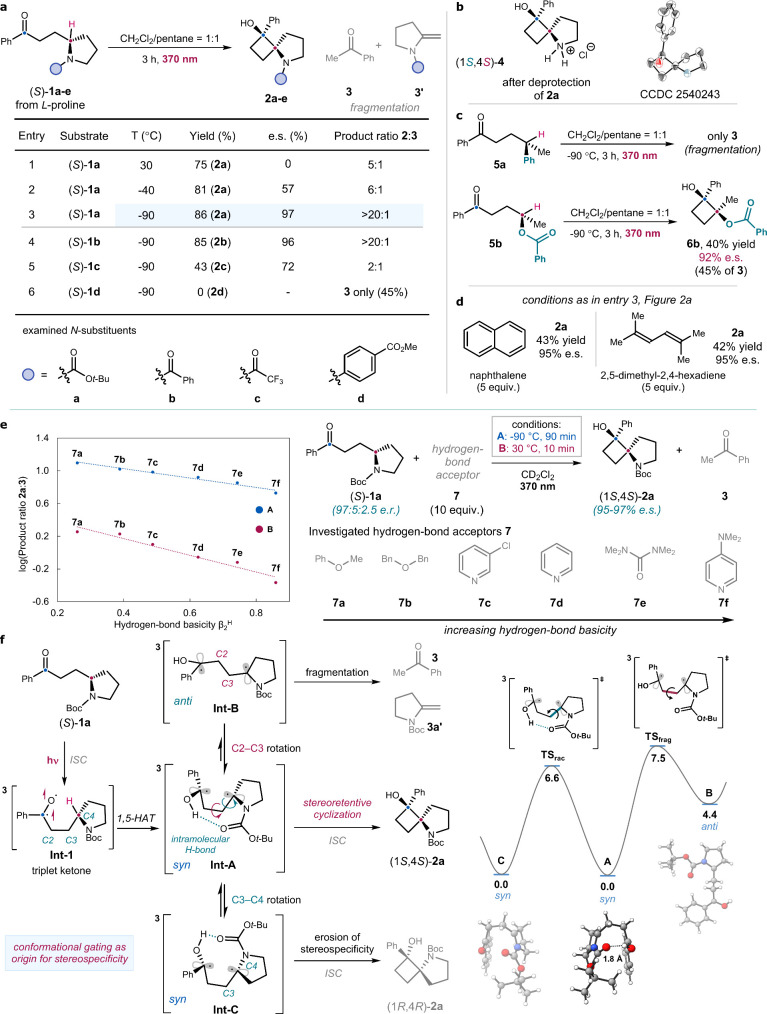
Experimental and computational studies. (**a**) Temperature and *N*-protecting-group effects on Yang cyclization/fragmentation and enantiospecificity (e.s.). (**b**) X-ray structure of **4**. (**c**) Requirement for an internal H-bond acceptor to drive a Yang cyclization pathway. (**d**) Triplet-quenching experiments. (**e**) Effect of external hydrogen-bond acceptors on product partitioning. (**f**) DFT-supported conformational landscape at the IEF-PCM­(dichloromethane)-UB3LYP-GD3BJ/6-311G­(d,p) level of theory. “e.s.” denotes enantiospecificity calculated from substrate and product enantiomeric compositions.

To probe the role of the hydrogen-bonding unit embedded in the substrate, we next examined the effect of the *N*-protecting group within the pyrrolidine scaffold of **1**. Replacing the *N*-Boc group with a *N*-benzoyl moiety (**1b**) had little effect on the reaction outcome, with both stereoretention and selective Yang cyclization maintained (entry 4, Figure 2a). By contrast, substrate **1c**, bearing a *N*-trifluoroacetyl group with reduced hydrogen-bond accepting ability,^21^ afforded the Yang product but with lower stereochemical fidelity and product selectivity (entry 5). In addition, the *N*-aryl substrate **1d**, lacking an accessible H-bonding unit, exclusively underwent Norrish fragmentation, and no cyclization product was detected (entry 6). Consistent with this picture, control substrate **5a**, selected because it most closely matches the steric demand and radical-stabilization pattern of the productive substrates **1a** and **1b** while lacking the γ hydrogen-bonding unit, underwent exclusive Norrish type II fragmentation under the optimized conditions, with no detectable Yang cyclization, even at −90 °C (Figure 2c). In contrast, installation of a benzoyl substituent at the γ-position on the same scaffold (**5b**), thus reintroducing a lone-pair-bearing unit capable of engaging in the putative intramolecular hydrogen-bonding network upon formation of the 1,4-diradical, restored Yang cyclization and did so with high stereospecificity (product **6b** obtained in 40% yield, 95:5 e.r., and 92% e.s.). Taken together, these results identify intramolecular hydrogen bonding as the key structural feature that directs both product partitioning and stereochemical fidelity, while low temperature amplifies this effect by limiting access to competing pathways.

We then further interrogated the role of hydrogen bonding by examining the effect of external hydrogen-bond acceptors on product partitioning using *N*-Boc substrate **1a** (Figure 2e). Addition of 10 equiv of acceptors **7a**–**7f** progressively reduced the Yang cyclization/fragmentation ratio (**2a**:**3**), with the magnitude of the effect correlating with the hydrogen-bond basicity of the additive. A near-linear dependence of log­(**2a**:**3**) on the Abraham^21^ hydrogen-bond basicity parameter β_2_^H^ was observed at both −90 and 30 °C, consistent with systematic perturbation of the hydrogen-bonding network that gates the reactive 1,4-diradical conformational manifold toward productive cyclization. Notably, high enantiospecificity was maintained across the additive series.

We then performed further experiments to probe the molecularity of the stereodetermining step and the nature of the reactive excited state. A nonlinear effect (NLE) analysis performed at −90 °C revealed a linear correlation between the enantiomeric purity of **1a** and product **2a**, and dilution of the reaction did not affect enantiospecificity. Together, these observations support a unimolecular pathway in which stereochemical information is preserved within a single substrate-derived intermediate. Addition of the established triplet quenchers naphthalene and 2,5-dimethyl-2,4-hexadiene (Figure 2d) significantly reduced product **2a** formation, while leaving both stereochemical fidelity and product partitioning unchanged (no fragmentation detected), indicating that the reaction proceeds through a triplet-state manifold in which conformational control is established after triplet formation.

Collectively, these results support the design principle outlined in Figure 1d, namely that an embedded intramolecular hydrogen-bonding network can impose conformational control directly within the photogenerated triplet 1,4-diradical manifold. Following photoexcitation and ISC, substrate (*S*)-**1a** forms triplet ketone **Int-1**, which undergoes 1,5-HAT to generate a triplet 1,4-diradical (Figure 2f). Within this manifold, the intramolecularly hydrogen-bonded *syn* conformer **Int-A** lies on the productive pathway to stereoretentive Yang cyclization, thereby preserving the stereochemical information encoded in the precursor triplet conformer. By contrast, rotation about the C2–C3 bond gives access to *anti* conformer **Int-B**, which is higher in energy and diverts the reaction toward Norrish fragmentation, whereas rotation about the C3–C4 bond interconverts **Int-A** with the isoenergetic alternative *syn* conformer **Int-C**, poised to furnish the opposite enantiomer of **2a** and thus erode stereospecificity. DFT calculations support this picture by identifying substantial barriers for both of these conformational exchanges (ΔG^‡^ = 7.5 kcal mol^–1^ for access to **Int-B** and 6.6 kcal mol^–1^ for interconversion to **Int-C**; Figure 2f, right panel). Under the low-temperature reaction conditions, these rotational processes become sufficiently slow that neither fragmentation nor stereochemical scrambling can compete with ISC from the preorganized triplet conformer followed by rapid cyclization within the ensuing singlet manifold. Thus, intramolecular hydrogen bonding defines the productive *syn* geometry within the 1,4-diradical, while cooling suppresses the C2–C3 and C3–C4 bond rotations that would otherwise lead to fragmentation and loss of stereochemical information. Together, these findings support a model in which hydrogen-bond-guided conformational control enables stereoretentive Norrish–Yang photocyclization in solution.

We then examined the generality of the stereoretentive Yang photocyclization (Figure 3). Enantioenriched ketone substrates **1** and **5** are readily accessible from commercially available enantiopure carboxylic acids in four steps. Across all cases examined, the photochemical cyclization consistently delivered products bearing two stereocenters as single detectable diastereomers. We first examined chiral ketones **1**, which furnish enantioenriched azaspirocyclobutanol products **2** (Figure 3a). To assess the robustness of the method, we performed the reaction of **1a** on a synthetically useful 1.5 mmol scale, affording spirocyclic product **2a** in 93% yield (418 mg) with 93% enantiospecificity.

**Figure 3:**
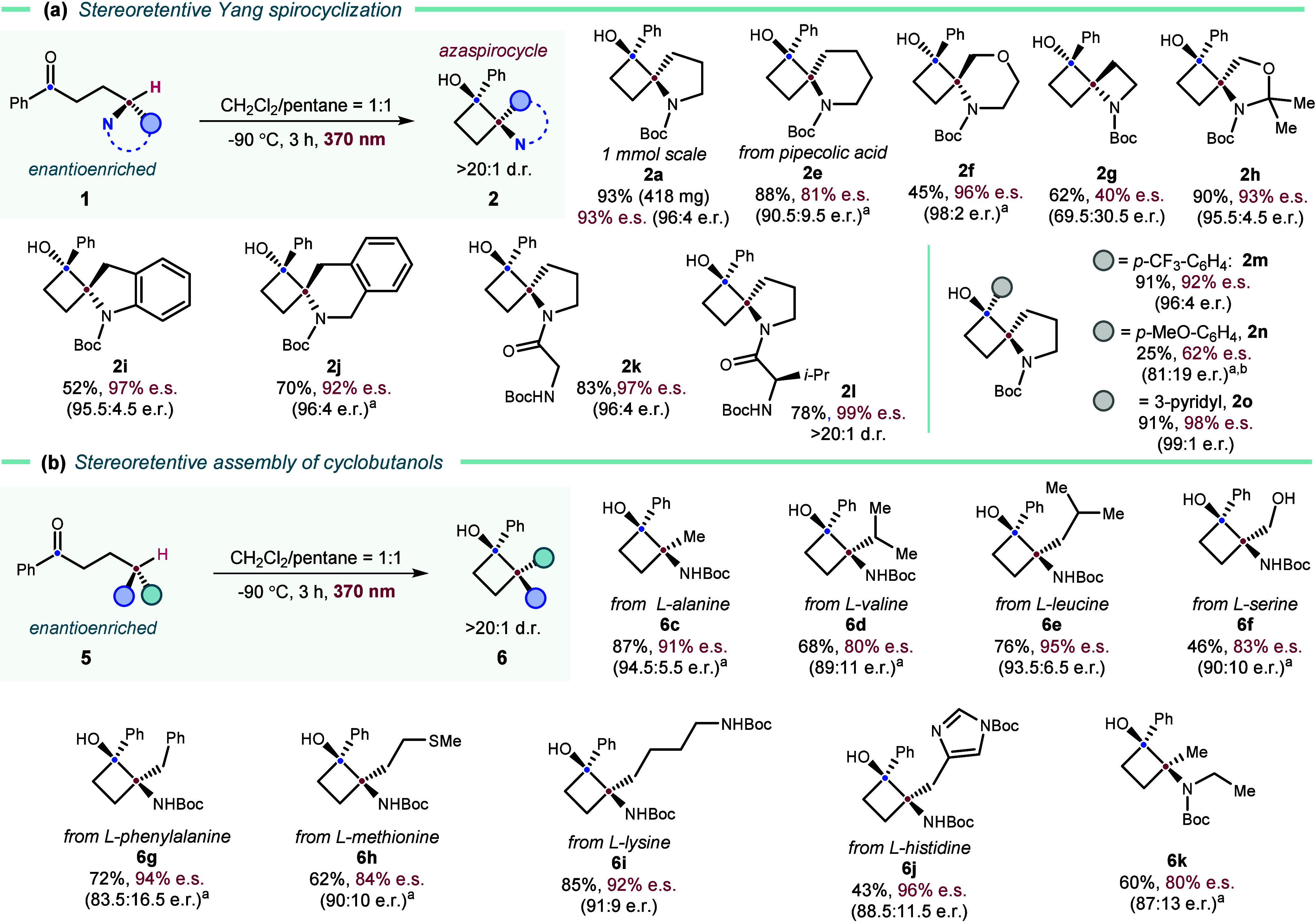
Scope of stereoretentive Yang photocyclization. (**a**) Azaspirocyclobutanols **2** from chiral ketones **1**. (**b**) Cyclobutanols **6** from amino-acid-derived ketones **5**. Yields are averages of two runs; e.s. = |ee­(product)|/|ee­(substrate)| × 100. Product e.r. values are shown in parentheses. Products were single detectable diastereomers. The absolute configurations shown reflect those of the corresponding enantioenriched amino acid precursors used in each case. ^a^CH_2_Cl_2_ as solvent. ^b^–50 °C, 12 h.

This conformational-control strategy is not limited to l-proline-derived scaffolds: piperidine, morpholine, oxazolidine, indoline, tetrahydroisoquinoline, and dipeptide-derived substrates furnished products **2e**, **2f**, and **2h**–**2l** with high enantiospecificity, whereas azetidine **2g** showed lower stereochemical fidelity. Aryl substitution showed the expected electronic dependence: *p*-CF_3_ was well tolerated (**2m**), whereas *p*-MeO **2n** required −50 °C and gave lower e.s. (62%), consistent with reduced γ-H abstraction from low-lying π,π* triplet states.^22^ The method also tolerated a 3-pyridyl-substituted aryl ketone, which furnished **2o** with high enantiospecificity. We next tested enantioenriched substrates **5** derived from linear chiral amino acids, whose greater conformational freedom could favor equilibration of the triplet 1,4-diradical intermediate (Figure 3b). Despite this potential liability, the reaction remained effective. Enantioenriched substrates derived from alanine, valine, leucine, serine, phenylalanine, methionine, lysine, and histidine delivered cyclobutanols **6c**–**6j** with high enantiospecificity and as single detectable diastereomers. Poorly reactive or unreactive substrates are collected in Figure S1; notably, the methyl ketone analogue of aryl substrate **1a** was recovered unchanged under 370 nm irradiation.

In conclusion, we provide a solution to a longstanding problem in classical photochemistry by showing that hydrogen-bond-guided conformational control can impart both type selectivity and stereochemical fidelity to the Norrish–Yang photocyclization. Intramolecular hydrogen-bond interactions embedded in chiral ketones preorganize the photogenerated triplet 1,4-diradical, suppressing conformational motions that lead to fragmentation and stereochemical erosion, thereby enabling stereoretentive Yang cyclization in solution.

## Supplementary Material



## Notes

Details of experimental procedures, full characterization data, and copies of NMR spectra (PDF)

## References

[1] Yan M., Lo J. C., Edwards J. T., Baran P. S. (2016). Radicals: reactive
intermediates with translational potential. J. Am. Chem. Soc..

[2] Johnston L. J., Ingold K. U. (1986). Kinetics of cyclopropyl radical reactions.
2. Studies on the inversion of cyclopropyl and 1-methylcyclopropyl
radicals and on the kinetics of some addition and abstraction reactions
of 1-methylcyclopropyl and 1-methoxycyclopropyl radicals. J. Am. Chem. Soc..

[3] Gloor C. S., Dénès F., Renaud P. (2016). Memory of chirality in reactions
involving monoradicals. Free Radical Research.

[4] Sun J., He J., Massaro L., Cagan D. A., Tsien J., Wang Y., Attard F. C., Smith J. E., Lee J. S., Kawamata Y., Baran P. S. (2025). Stereoretentive
radical cross-coupling. Nature.

[5] Tseliou V., Kqiku L., Berger M., Schiel F., Zhou H., Poelarends G. J., Melchiorre P. (2024). Stereospecific radical coupling with
a non-natural photodecarboxylase. Nature.

[6] Yang N. C., Yang D.-H. (1958). Photochemical reactions
of ketones in solution. J. Am. Chem. Soc..

[7] De Feyter S., Diau E. W.-G., Zewail A. H. (2000). Femtosecond
dynamics
of Norrish type-II reactions: nonconcerted hydrogen transfer and diradical
intermediacy. Angew. Chem., Int. Ed..

[8] a Wagner, P. J. ; Klán, P. Norrish type II photoelimination of ketones: cleavage of 1,4-biradicals formed by γ-hydrogen abstraction. CRC Handbook of Organic Photochemistry and Photobiology; Horspool, W. M. , Lenci, F. , Eds.; CRC Press, 2004; pp 52-1–52-31.

[9] Seebach D. (1979). Methods of
Reactivity Umpolung. Angew. Chem., Int. Ed.
Engl..

[10] Small R. D., Scaiano J. C. (1977). Photochemistry of phenyl alkyl ketones.
The lifetime of the intermediate biradicals. J. Phys. Chem..

[11] Wagner P. J. (1971). Type II photoelimination and photocyclization
of ketones. Acc. Chem. Res..

[12] Singhal N., Koner A. L., Mal P., Venugopalan P., Nau W. M., Moorthy J. N. (2005). Diastereomer-differentiating
photochemistry of β-arylbutyrophenones: Yang cyclization versus
type II elimination. J. Am. Chem. Soc..

[13] Moorthy J. N., Samanta S., Koner A. L., Saha S., Nau W. M. (2008). Intramolecular
O-H···O hydrogen-bond-mediated reversal in the partitioning
of conformationally restricted triplet 1,4-biradicals and amplification
of diastereodifferentiation in their lifetimes. J. Am. Chem. Soc..

[14] Leibovitch M., Olovsson G., Scheffer J. R., Trotter J. (1998). An investigation
of the Yang photocyclization reaction
in the solid state: asymmetric induction studies and crystal structure-reactivity
relationships. J. Am. Chem. Soc..

[15] Bach T., Aechtner T., Neumüller B. (2002). Enantioselective
Norrish–Yang
cyclization reactions of *N*-(ω-oxo-ω-phenylalkyl)-substituted
imidazolidinones in solution and in the solid state. Chem. Eur. J..

[16] Scaiano J. C. (1982). Does intersystem
crossing in triplet biradicals generate singlets with conformational
memory?. Tetrahedron.

[17] Griesbeck A. G., Mauder H., Stadtmüller S. (1994). Intersystem
crossing in triplet 1,4-biradicals:
conformational memory effects on the stereoselectivity of photocycloaddition
reactions. Acc. Chem. Res..

[18] Giese B., Wettstein P., Stähelin C., Barbosa F., Neuburger M., Zehnder M., Wessig P. (1999). Memory of chirality in photochemistry. Angew. Chem., Int. Ed..

[19] Varela M. T. (2025). Spirocyclic compounds
as innovative tools in drug discovery for medicinal
chemists. Eur. J. Med. Chem..

[20] Crystallographic data for compound **4** have been deposited with the Cambridge Crystallographic Data Centre under CCDC 2540243.

[21] Abraham M.
H., Grellier P. L., Prior D. V., Morris J. J., Taylor P. J. (1990). Hydrogen
bonding. Part 10. A scale of solute hydrogen-bond basicity using log
K values for complexation in tetrachloromethane. J. Chem. Soc., Perkin Trans..

[22] Wagner P. J., Kemppainen A. E., Schott H. (1973). Effects of ring substituents on the
type II photoreactions of phenyl ketones. How interactions between
nearby excited triplets affect chemical reactivity. J. Am. Chem. Soc..

