# Supplementary material for: Stereoretentive Norrish–Yang Photocyclization Mediated by Hydrogen Bonding
Source: J Am Chem Soc. 2026 Jun 26;148(26):27060–5. doi: 10.1021/jacs.6c09924 (PMC13352618; doi:10.1021/jacs.6c09924)

Supplementary Information for

Stereoretentive Norrish–Yang Photocyclization Mediated by Hydrogen Bonding

Enrico Sfreddo[1], Aleksa Durdevic[1], Andrea Palone[1], Tristan von Münchow[1], Nunzio Matera[1], Andrea Mazzanti[1], Paolo Melchiorre[1*]

[1]University of Bologna, Department of Industrial Chemistry '*Toso Montanari*'
via Piero Gobetti 85 – 40129 Bologna, Italy

[*]Correspondence to: p.melchiorre@unibo.it

Contents

A. General Information ... 3

B. Substrates Outside the Productive Scope ... 4

C. Synthesis and Characterization of Starting Materials ... 5

C.1 General Procedure for the Synthesis of Vinyl Aryl Ketones 9 ... 5

C.2 General Procedures for the Synthesis of Enantioenriched Substrates 1 and 5 ... 6

D. Experimental Procedures ... 34

D.1 General Procedure for the Stereoretentive Yang Photocyclization ... 34

D.2 Characterization of the Norrish-Yang Photocyclization Products ... 36

E.1 X-Ray Crystallographic Data for Compound 4 ... 61

E.2 Relative Configuration Assignment of Compound 6c ... 66

E.3 Absolute Configuration Assignment of Compound 6c ... 67

E.4 Relative Configuration Assignment of Compound 2l ... 70

F. Mechanistic Studies ... 78

F.1 Effect of Triplet Quenchers ... 78

F.2 Non-linear Effect Study ... 80

F.3 Effect of Substrate Concentration ... 82

F.4 Effect of Hydrogen-Bond Acceptors ... 84

F.5 Effect of Hydrogen-Bond Donors ... 86

F.6 Effect of the Solvent ... 87

F.7 Computational Studies ... 88

G. References ... 94

A. General Information

The NMR spectra were recorded at 400, 500 or 600 MHz for ^{1}H and 101, 126 or 151 MHz for ^{13}C. The chemical shift (δ) for ^{1}H and ^{13}C are given in ppm relative to residual signals of the solvents ($CHCl_3$ at 7.26 ppm for ^{1}H NMR and 77.06 ppm for ^{13}C NMR). Coupling constants are given in Hertz. The following abbreviations are used to indicate the multiplicity: s, singlet; d, doublet; q, quartet; m, multiplet; br, broad signal.

High resolution mass spectra (HRMS) were obtained from the Mass Facility unit on a Waters Xevo Q-Tof spectrometer with electrospray ionization (ESI). UV-vis measurements were carried out on a Cary 3500 Multicell UV-vis spectrophotometer. Enantiomeric excesses were determined by chiral HPLC (Agilent 1100 or 1200 series) or chiral SFC (Agilent 1260 Infinity II) using chiral stationary phase columns. X-ray data were acquired on a Bruker APEX-2 diffractometer.

Selected substrates were resolved by preparative chiral HPLC using a Waters 600 HPLC pump, a Rheodyne7012 injector (loop of 1 mL) and a Waters 2487 UV detector (Waters, Milford, MA, USA) with a wavelength set at 254 nm. Chiral separations were performed using either a Chiralpak AD-H column (250 × 20 mm) or a (*S*,*S*)-Whelk-O2 column (10 μm, 250 × 10 mm).

The ECD spectra were acquired in the 190–400 nm region using a JASCO J-810 spectropolarimeter in far-UV HPLC-grade acetonitrile solution. Concentration was about 1 × 10−4 M, tuned by dilution to have a maximum absorbance between 0.8 and 1 with a cell path of 0.2 cm. The spectra were obtained by the average of 6 scans at 50 nm·min−1 scan rate.

Yields refer to isolated materials of >95% purity as determined by ^{1}H NMR analysis, unless mentioned otherwise.

General Procedures. All reactions were set up under a nitrogen atmosphere using standard Schlenk techniques. Where specified, reactions were performed in a Emme 3 Vigor glovebox. Synthesis grade solvents were used as purchased and anhydrous solvents were taken from commercially available septum-sealed bottles. Chromatographic purification of products was accomplished using forced-flow chromatography (FC) on silica gel (230-400 mesh). Thin-layer chromatography (TLC) analyses were performed on Merck pre-coated TLC plates (silica gel 60 GF254, 0.25 mm), using UV light for visualization and a basic aqueous potassium permanganate ($KMnO_4$) stain followed by heating for development. Organic solutions were concentrated under reduced pressure using a Büchi rotary evaporator.

Materials. Commercial reagents and solvents were purchased from commercial suppliers at the highest available quality and used as received, unless otherwise stated

B. Substrates Outside the Productive Scope

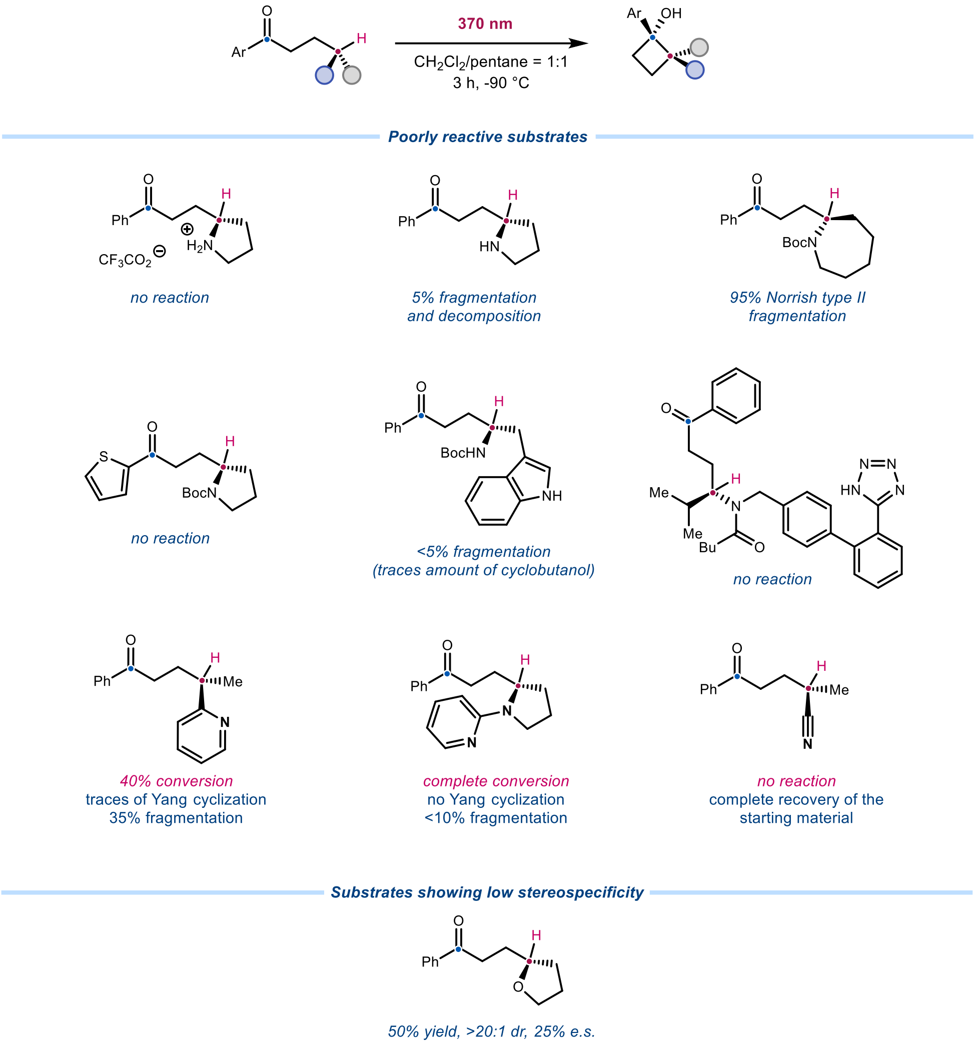

Figure S1. Unsuccessful substrates and substrates with poor stereochemical fidelity. Reactions were carried out on a 0.05 mmol scale.

C. Synthesis and Characterization of Starting Materials

C.1 General Procedure for the Synthesis of Vinyl Aryl Ketones 9

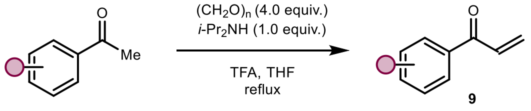

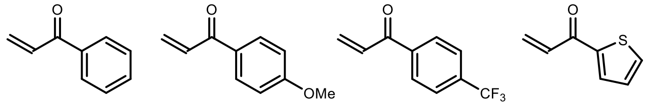

9a **9b** **9c** **9d**

To a solution of diisopropylamine (1.0 equiv) in THF was added trifluoroacetic acid (1.1 equiv.). After stirring for 5 min at room temperature, acetophenone derivative (1.0 equiv.) was added, followed by paraformaldehyde (2.0 equiv). The reaction mixture was heated to reflux and stirred for 2 h, after which an additional portion of paraformaldehyde (2.0 equiv.) was added. The reaction was then allowed to reflux overnight. After cooling to room temperature, Et_2O (50 mL) was added and the organic phase was washed with 1M HCl, saturated aqueous $NaHCO_3$, and brine. The organic layer was dried over $MgSO_4$, filtered, and concentrated under reduced pressure. The crude residue was purified by flash column chromatography (5–8% EtOAc in hexane) to afford the desired ketone **9**. The characterization data of the synthesized ketones were consistent with those reported in the literature.[1,2]

C.2 General Procedures for the Synthesis of Enantioenriched Substrates 1 and 5

General procedure **A:**

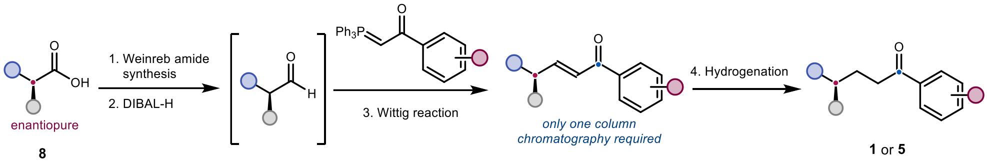

1.Weinreb amide formation

To a stirred solution of commercially available enantiopure chiral carboxylic acid **8** (1 equiv.) and *N,O*-dimethylhydroxylamine hydrochloride (1.1 equiv.) in CH_2Cl_2 were added *N*-methylmorpholine (2 equiv.) and EDC·HCl (1.2 equiv.) sequentially at room temperature. The reaction mixture was stirred overnight at room temperature and then diluted with CH_2Cl_2 (20 mL). The organic phase was washed with 1M HCl, saturated aqueous $NaHCO_3$, and brine, dried over $MgSO_4$, filtered, and concentrated under reduced pressure. The resulting amide was used directly in the next step without further purification.

2.DIBAL-H reduction

To a solution of the Weinreb amide (1 equiv.) in CH_2Cl_2 (20 mL) at -40 °C was added DIBAL-H (1.0 M in toluene, 1.5 equiv.) dropwise under stirring. The reaction mixture was stirred at -40 °C for 1 h. The reaction was quenched by pouring the mixture into 1M HCl and the resulting suspension was extracted with CH_2Cl_2. The combined organic layers were washed with brine, dried over Na_2SO_4, filtered, and concentrated under reduced pressure. The crude aldehyde was used directly in the next step without further purification.

3.Wittig olefination

A 100 mL round-bottom flask was charged with (benzoylmethylene)triphenylphosphorane (2 equiv.), $CHCl_3$ (50 mL), and the aldehyde (1 equiv.). The reaction mixture was heated to reflux in an oil bath and stirred for 16 h. After cooling to room temperature, the mixture was filtered through a silica plug. The solvent was removed under reduced pressure and the crude residue was purified by flash column chromatography on silica (5 – 50% EtOAc in hexane) to afford the desired enone. This is the only purification required for the four-step sequence.

4.Hydrogenation

A solution of the alkene in EtOAc (60 mL) containing 10% Lindlar catalyst or Pd/C was placed under an atmosphere of H_2 (1 atm) and stirred overnight. After completion of the reaction, the mixture was filtered through a pad of *Celite* and the solvent was removed under reduced pressure to afford the enantioenriched title compound. Unless otherwise specified, the ketone was used directly as a substrate. The enantiomeric purity of the product was determined by chiral HPLC analysis. In selected cases where partial racemization had occurred over the four-step sequence, the corresponding enantiopure ketone substrates were obtained by preparative chiral HPLC separation.

C.3 Synthesis and Characterization of Substrates 1 and 5

tert-Butyl (*S*)-2-(3-oxo-3-phenylpropyl)pyrrolidine-1-carboxylate (1a)

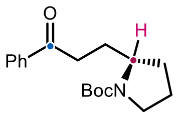

1a

Synthesized according to the general procedure **A** from enantiopure *N*-Boc-*L*-Proline (2 g, 15 mmol, 1 equiv.). The title compound was obtained as a pale-yellow oil (2.34 g, 83% yield over 4 steps). The enantiomeric ratio was determined by SFC analysis on a chiral Daicel Chiralpak IG-3 (95:5 CO_2:*i*-PrOH, 1.0 mL/min, 20 °C, λ = 210 nm: τ_{major} = 12.3 min, τ_{minor} = 18.6 min), >99.5:0.5 e.r.; $[\alpha]^D{}_{20}$ = -27.6 (c = 0.17, $CHCl_3$).

^{1}H NMR (400 MHz, $CDCl_3$) (mixture of rotamers) δ 7.99 – 7.91 (m, 2H), 7.53 (s, 1H), 7.45 (m, 2H), 3.91 (m, 1H), 3.38 (m, 3H), 2.97 (m, 2H), 2.10 – 1.75 (m, 6H), 1.67 (m, 1H), 1.43 (s, 9H).

^{13}C NMR (151 MHz, $CDCl_3$) (mixture of rotamers) δ 200.11, 199.72, 154.89, 136.98, 136.84, 133.05, 132.86, 128.60, 128.08, 79.32, 78.97, 56.77, 46.43, 46.12, 35.87, 35.40, 30.99, 30.45, 29.27, 28.50, 28.28, 23.74, 23.04.

HRMS (ESI$^+$) $C_{18}H_{25}NO_3Na$ [M+Na]$^+$: found 326.1729, required 326.1727.

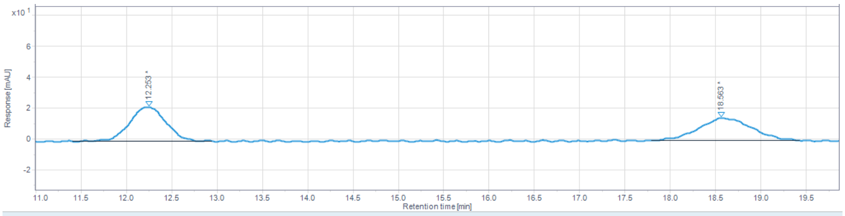

Injection Results

Peaks | Summary

#	Signal description	RT (min)	Area (mAU·s)	Area%	Height (mAU)	Height%	Amount	Concentration	Start time (min)	End time (min)
1	DAD1C,Sig=210,4 ...	12.253	617.536	51.213	22.093	60.38			11.408	12.946
2	DAD1C,Sig=210,4 ...	18.563	588.292	48.787	14.496	39.62			17.794	19.427

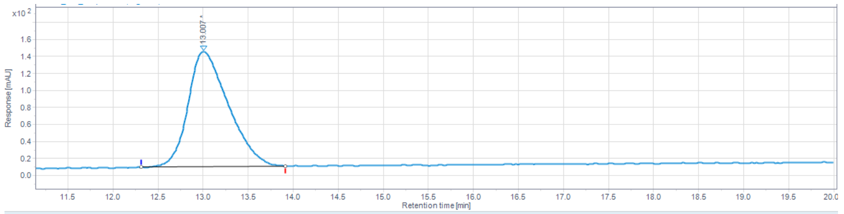

Injection Results

Peaks | Summary

#	Signal description	RT (min)	Area (mAU·s)	Area%	Height (mAU)	Height%	Amount	Concentration	Start time (min)	End time (min)
1	DAD1C,Sig=210,4 ...	13.007	4218.182	100.000	135.359	100.00			12.312	13.914

(*S*)-3-(1-Benzoylpyrrolidin-2-yl)-1-phenylpropan-1-one (1b)

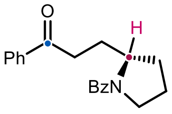

1b

Synthesized according to the general procedure **A** from enantiopure *N*-Bz-*L*-Proline (500 mg, 2.3 mmol, 1 equiv.). The title compound **1b** was obtained as a pale-yellow oil (407 mg, 58% yield over 4 steps). The enantiomeric ratio was determined by HPLC analysis on a chiral Daicel Chiralpak AD-H (90:10 hexane:*i*-PrOH, 1.0 mL/min, 30 °C, λ = 254 nm: τ_{minor} = 27.9 min, τ_{major} = 37.7 min), 98.5:1.5 e.r.; $[\alpha]^{D}_{20}$ = -77.6 (c = 0.13, $CHCl_3$).

^{1}H NMR (600 MHz, $CDCl_3$) (mixture of rotamers) δ 7.99 (d, *J* = 7.7 Hz, 2H), 7.74 (s, 1H), 7.57 – 7.51 (m, 1H), 7.42 (ddt, *J* = 39.2, 16.7, 6.7 Hz, 7H), 4.49 (p, *J* = 6.6 Hz, 1H), 3.51 (dt, *J* = 10.9, 7.2 Hz, 1H), 3.40 (dt, *J* = 11.1, 5.5 Hz, 1H), 3.22 (ddd, *J* = 15.8, 9.6, 5.7 Hz, 1H), 3.10 (ddd, *J* = 16.6, 9.7, 5.6 Hz, 1H), 2.66 (s, 1H), 2.31 (dq, *J* = 14.6, 6.1 Hz, 1H), 2.13 (dt, *J* = 14.2, 7.0 Hz, 1H), 1.99 (td, *J* = 12.3, 5.9 Hz, 2H), 1.76 (ddq, *J* = 32.3, 12.9, 7.1 Hz, 3H).

^{13}C NMR (151 MHz, $CDCl_3$) (mixture of rotamers) δ 200.09, 170.55, 137.28, 136.87, 132.98, 129.89, 128.58, 128.23, 128.15, 127.25, 56.74, 49.86, 35.90, 30.67, 29.17, 24.90.

HRMS (ESI^{+}) $C_{20}H_{21}NO_2Na$ [M+Na]$^{+}$: found 330.1467, required 330.1465.

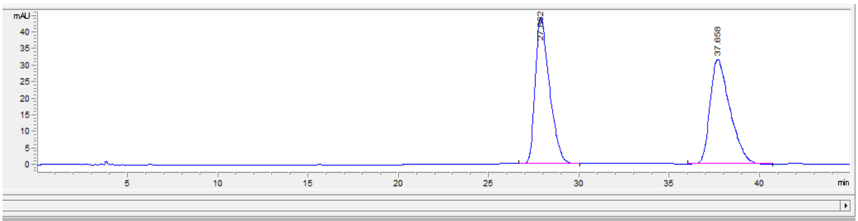

#	Time	Area	Height	Width	Area%	Symmetry
1	27.862	2407.6	43.9	0.8215	49.806	0.594
2	37.658	2426.4	31.6	1.137	50.194	0.585

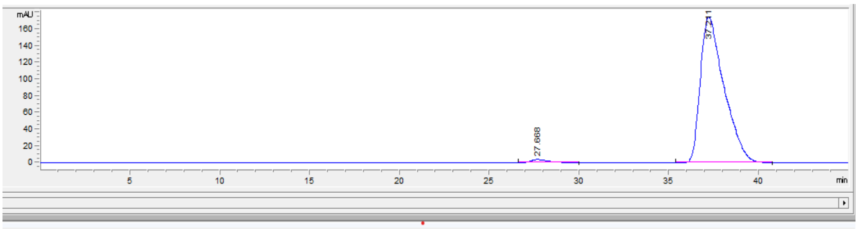

#	Time	Area	Height	Width	Area%	Symmetry
1	27.668	242.6	3.7	0.9323	1.501	0.57
2	37.211	15920	174.2	1.3421	98.499	0.516

(S)-1-phenyl-3-(1-(2,2,2-trifluoroacetyl)pyrrolidin-2-yl)propan-1-one (1c)

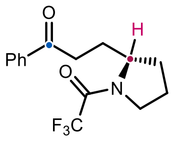

1c

Synthesized according to the general procedure **A** from enantiopure *N*-Trifluoroacetyl-*L*-Proline (450 mg, 2 mmol, 1 equiv.). The title compound **1c** was obtained as a yellow oil (149 mg, 33% yield over 4 steps). The enantiomeric ratio was determined by SFC analysis on a chiral Daicel Chiralpak IB-N3 (95:5 CO_2: *i*-PrOH, 1.0 mL/min, 20 °C, λ = 210 nm: τ_{minor} = 2.3 min, τ_{major} = 2.9 min), 99.5:0.5 e.r.;

^{1}H NMR (600 MHz, $CDCl_3$) (mixture of rotamers) δ 7.95 (dt, *J* = 8.5, 1.1 Hz, 2H), 7.59 – 7.53 (m, 1H), 7.50 – 7.44 (m, 2H), 4.34 – 4.23 (m, 1H), 3.72 – 3.64 (m, 2H), 3.11 (ddd, *J* = 17.4, 9.3, 5.8 Hz, 1H), 3.05 – 2.97 (m, 1H), 2.24 – 2.18 (m, 1H), 2.11 (dq, *J* = 11.4, 7.5 Hz, 1H), 2.01 (tdd, *J* = 11.7, 9.5, 6.2 Hz, 2H), 1.94 – 1.86 (m, 1H), 1.82 – 1.76 (m, 1H).

^{13}C NMR (151 MHz, $CDCl_3$) (mixture of rotamers) δ 199.27, 155.76, 136.69, 133.39, 133.15, 130.63, 128.74, 128.65, 128.22, 128.07, 128.00, 117.33, 58.93, 47.24, 46.46, 46.43, 46.41, 35.67, 35.38, 31.83, 30.13, 29.37, 27.81, 24.18, 20.47.

^{19}F NMR (376 MHz, $CDCl_3$) (mixture of rotamers) δ -70.57, -72.36.

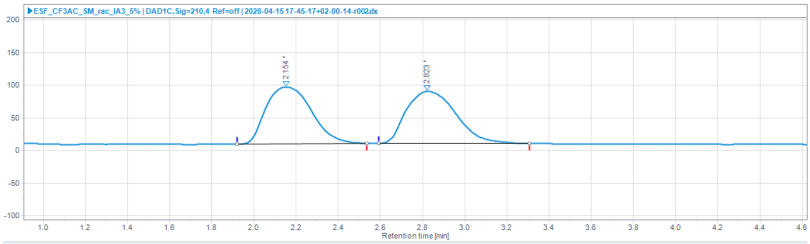

Injection Results

Peaks | Summary

#	Signal descript...	RT (min)	Area (mAU·s)	Area%	Height (mAU)	Height%	Amount	Concentration	Start time (min)	End time (min)
1	DAD1C,Sig=210,4 ...	2.154	8990.082	50.135	597.088	52.26			1.920	2.538
2	DAD1C,Sig=210,4 ...	2.823	8941.722	49.865	545.440	47.74			2.594	3.310

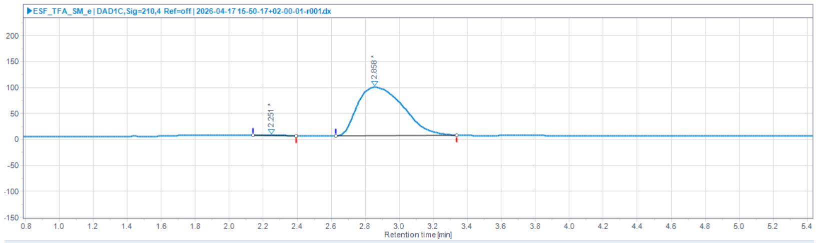

njection Results

Peaks | Summary

#	Signal descript...	RT (min)	Area (mAU·s)	Area%	Height (mAU)	Height%	Amount	Concentration	Start time (min)	End time (min)
1	DAD1C,Sig=210,4 ...	2.251	42.997	0.224	7.169	0.70			2.140	2.394
2	DAD1C,Sig=210,4 ...	2.858	19136.128	99.776	1017.408	99.30			2.626	3.338

***tert*-Butyl (+)-2-(3-oxo-3-phenylpropyl)piperidine-1-carboxylate (1e)**

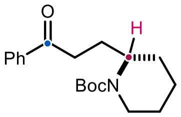

1e

Synthesized according to the general procedure **A** using enantiopure *N*-Boc-*D*-Pipecolic acid (1 g, 4.3 mmol, 1 equiv.). After the hydrogenation step, the crude mixture was purified by flash column chromatography on silica gel (20% EtOAc in hexane). Racemization was observed during the reaction sequence; consequently, the product was resolved by preparative chiral HPLC using a Chiralpak AD-H column (250 mm x 20 mm) to afford the title compound **1e** as a white solid (0.4 g, 29% yield after preparative chiral HPLC). The enantiomeric ratio was determined by HPLC analysis on a chiral Daicel Chiralpak AD-H (90:10 hexane:*i*-PrOH, 1.0 mL/min, 30 °C, λ = 254 nm: τ_{major} = 6.3 min, τ_{minor} = 8.1 min,), >99.5:0.5 e.r.; $[\alpha]^{D}_{20}$ = +27.0 (c = 0.13, CHCl3)

^{1}H NMR (600 MHz, CDCl$_3$) δ 7.97 – 7.92 (m, 2H), 7.58 – 7.52 (m, 1H), 7.45 (t, *J* = 7.8 Hz, 2H), 4.34 (s, 1H), 4.00 (s, 1H), 2.96 (dddd, 2H), 2.78 (t, *J* = 13.0 Hz, 1H), 2.31 – 2.11 (m, 1H), 1.88 – 1.78 (m, 1H), 1.72 – 1.57 (m, 6H), 1.38 (s, 9H).

^{13}C NMR (151 MHz, CDCl$_3$)) δ 199.74, 155.21, 136.96, 132.99, 128.56, 128.02, 79.23, 35.25, 29.19, 28.39, 25.65, 24.02, 19.13.

HRMS (ESI^{+}) $C_{19}H_{27}NO_3Na$ [M+Na]$^{+}$: found 340.1887, required 340.1884.

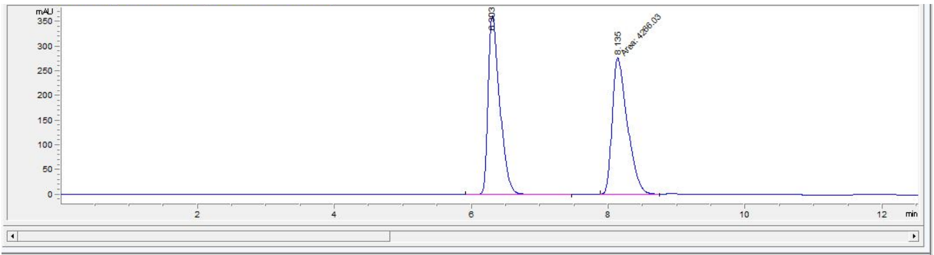

#	Time	Area	Height	Width	Area%	Symmetry
1	6.303	4243.9	361.6	0.1729	49.870	0.533
2	8.135	4266	276.7	0.257	50.130	0.567

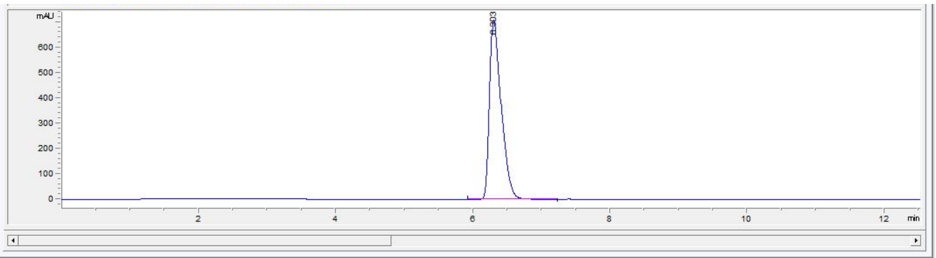

#	Time	Area	Height	Width	Area%	Symmetry
1	6.303	8417	708	0.1747	100.000	0.52

tert-Butyl (*S*)-3-(3-oxo-3-phenylpropyl)morpholine-4-carboxylate (1f)

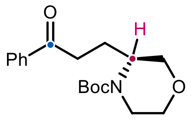

1f

Synthesized according to the general procedure **A** using enantiopure *N*-Boc-*D*-morpholine-3-carboxylic acid (0.23 g, 1.0 mmol, 1 equiv.) After the hydrogenation step, the crude mixture was purified by flash column chromatography on silica gel (20% EtOAc in hexane). Racemization was observed during the reaction sequence; consequently, the product was resolved by preparative chiral HPLC using a Chiralpak AD-H column (250 mm x 20 mm) to afford the title compound **1f** as a colorless oil (0.081 g, 35% yield after preparative chiral HPLC). The enantiomeric ratio was determined by HPLC analysis on a chiral Daicel Chiralpak AD-H (90:10 hexane:*i*-PrOH, 1.0 mL/min, 30 °C, λ = 254 nm: τ_{major} = 9.9 min, τ_{minor} = 12.4 min), >99.5:0.5 e.r.; $[\alpha]^{D}{}_{20}$ = +33.4 (c = 0.12, CHCl3).

^{1}H NMR (600 MHz, $CDCl_3$) δ 8.00 – 7.91 (m, 2H), 7.60 – 7.50 (m, 1H), 7.50 – 7.40 (m, 2H), 4.18 – 4.03 (m, 1H), 3.97 – 3.71 (m, 3H), 3.60 (dd, *J* = 11.5, 3.3 Hz, 1H), 3.45 (td, *J* = 11.8, 3.0 Hz, 1H), 3.24 – 3.10 (m, 1H), 3.03 (dt, *J* = 17.6, 7.5 Hz, 1H), 2.94 (ddd, *J* = 17.6, 7.6, 5.7 Hz, 1H), 2.40 – 2.27 (m, 1H), 2.17 – 1.95 (m, 1H), 1.34 (s, 9H).

^{13}C NMR (151 MHz, $CDCl_3$) δ 199.19, 154.90, 136.98, 133.20, 128.72, 128.16, 80.13, 69.98, 67.13, 50.89, 38.38, 34.69, 28.37, 23.11.

HRMS (ESI^{+}) $C_{18}H_{25}NO_4Na$ [M+Na]$^{+}$: found 342.1683, required 342.1676.

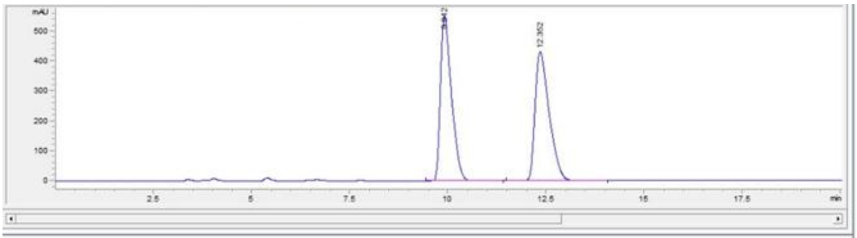

#	Time	Area	Height	Width	Area%	Symmetry
1	9.912	10657.3	551.5	0.2855	49.837	0.534
2	12.352	10727.1	431.4	0.3721	50.163	0.531

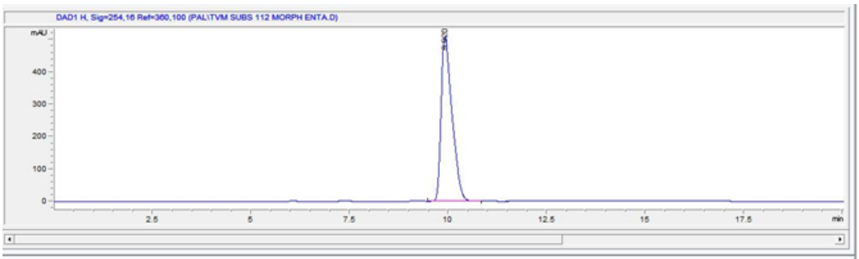

#	Time	Area	Height	Width	Area%	Symmetry
1	9.92	9844.2	508.6	0.2858	100.000	0.533

tert-Butyl (_S_)-2-(3-oxo-3-phenylpropyl)azetidine-1-carboxylate (1g)

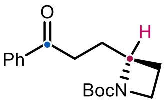

1g

Synthesized according to the general procedure **A** using enantiopure *N*-Boc-*D*-2-azetidinecarboxylic acid (1 g, 5 mmol, 1 equiv.). The title compound **1g** was obtained as a pale yellow oil (643 mg, 45% yield over 4 steps). The enantiomeric ratio was determined by SFC analysis on a chiral Daicel Chiralpak IG-3 (95:5 CO_2:*i*-PrOH, 1.0 mL/min, 20 °C, λ = 210 nm: τ_{minor} = 8.6 min, τ_{major} = 10.0 min), 98.5:1.5 e.r.; $[\alpha]^D_{20}$ = +52.8 (c = 0.08, $CHCl_3$)

^{1}H NMR (400 MHz, $CDCl_3$) δ 7.97 (dt, *J* = 7.1, 1.4 Hz, 2H), 7.59 – 7.52 (m, 1H), 7.46 (dd, *J* = 8.4, 7.0 Hz, 2H), 4.32 (dq, 1H), 3.82 (qd, *J* = 8.9, 6.4 Hz, 2H), 3.23 – 3.00 (m, 2H), 2.38 – 2.04 (m, 4H), 1.88 (ddt, *J* = 11.3, 8.9, 6.5 Hz, 1H), 1.42 (s, 9H).

^{13}C NMR (151 MHz, $CDCl_3$) δ 199.80, 156.89, 136.87, 133.01, 128.58, 128.09, 79.34, 61.62, 34.36, 30.40, 28.44, 28.42, 21.89.

HRMS (ESI$^+$) $C_{17}H_{23}NO_3Na$ [M+Na]$^+$: found 312.1574, required 312.1571.

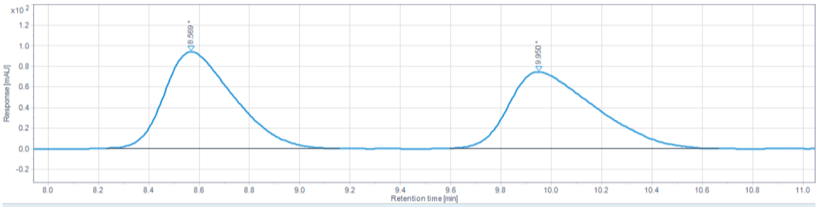

Injection Results

Peaks | Summary

#	Signal description	RT (min)	Area (mAU·s)	Area%	Height (mAU)	Height%	Amount	Concentration	Start time (min)	End time (min)
1	DAD1C,Sig=210,4 ...	8.569	1786.948	50.283	93.748	55.79			8.231	9.159
2	DAD1C,Sig=210,4 ...	9.950	1766.852	49.717	74.298	44.21			9.598	10.665

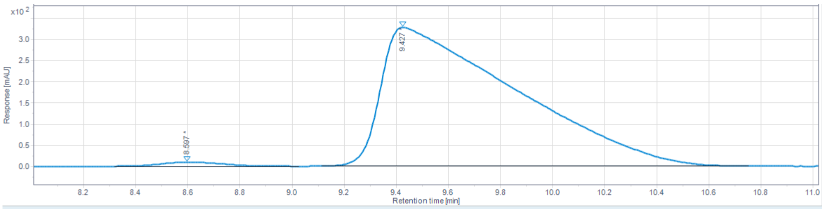

Injection Results

Peaks | Summary

#	Signal description	RT (min)	Area (mAU·s)	Area%	Height (mAU)	Height%	Amount	Concentration	Start time (min)	End time (min)
1	DAD1C,Sig=210,4 ...	8.597	164.356	1.376	9.860	2.93			8.320	9.026
2	DAD1C,Sig=210,4 ...	9.427	11779.502	98.624	326.961	97.07			9.115	10.754

tert-Butyl (R)-2,2-dimethyl-4-(3-oxo-3-phenylpropyl)oxazolidine-3-carboxylate (1h)

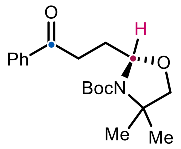

1h

Synthesized according to the general procedure **A** starting from enantiopure (*L*)-Garner's aldehyde (1.2 g, 5 mmol, 1 equiv.). The title compound **1h** was obtained as a pale yellow solid (937 mg, 54% yield over 4 steps). The enantiomeric ratio was determined by HPLC analysis on a chiral Daicel Chiralpak AD-H (90:10 hexane:*i*-PrOH, 1.0 mL/min, 30 °C, λ = 230 nm: τ_{minor} = 8.5 min, τ_{major} = 10.3 min), 99.5:0.5 e.r.; $[\alpha]^{D}_{20}$ = -45.0 (c = 0.07, $CHCl_3$)

^{1}H NMR (400 MHz, $CDCl_3$) (mixture of rotamers) δ 7.96 (d, *J* = 7.5 Hz, 2H), 7.55 (s, 1H), 7.46 (s, 2H), 4.13 – 3.91 (m, 2H), 3.79 (d, 1H), 3.02 (s, 2H), 2.14 – 1.96 (m, 2H), 1.59 (s, 3H), 1.43 (s, 12H).

^{13}C NMR (151 MHz, $CDCl_3$) (mixture of rotamers) δ 199.45, 152.55, 152.04, 136.95, 136.73, 133.17, 132.93, 128.66, 128.56, 128.07, 94.04, 93.47, 80.12, 79.80, 67.31, 56.98, 56.59, 35.41, 34.94, 28.43, 28.14, 27.68, 26.83, 24.48, 23.11.

HRMS (ESI^{+}) $C_{19}H_{27}NO_4Na$ $[M+Na]^{+}$: found 356.1836, required 356.1833.

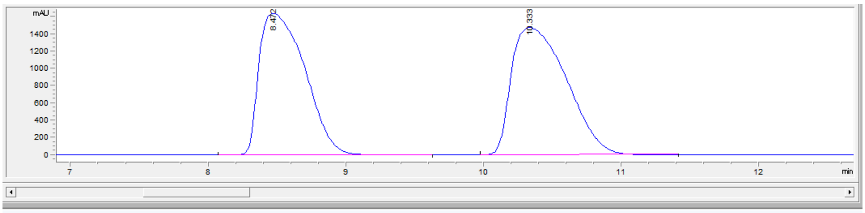

#	Time	Area	Height	Width	Area%	Symmetry
1	8.472	37662.9	1635.7	0.3866	47.920	0.436
2	10.333	40932.6	1470.4	0.4608	52.080	0.47

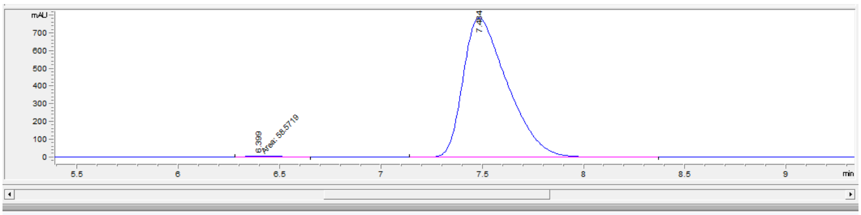

#	Time	Area	Height	Width	Area%	Symmetry
1	6.399	58.6	6.2	0.1587	0.488	0.586
2	7.484	11937.2	786.6	0.227	99.512	0.539

tert-butyl (S)-2-(3-oxo-3-phenylpropyl)indoline-1-carboxylate (1i)

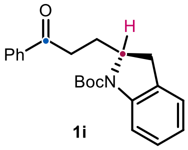

Synthesized according to the general procedure **A** starting from enantiopure (*R*)-1-(*tert*-butoxycarbonyl)indoline-2-carboxylic acid (527 mg, 2.0 mmol, 1 equiv.) The title compound **1i** was obtained as a yellow oil (302 mg, 43% yield over 4 steps). The enantiomeric ratio was determined by HPLC analysis on a chiral Daicel Chiralpak AD-H (90:10 hexane:*i*-PrOH, 1.0 mL/min, 30 °C, λ = 230 nm: τ_{major} = 7.5 min, τ_{minor} = 10.0 min), 97.0 :3.0 e.r.; $[\alpha]^{D}_{20}$ = +21.7 (c = 0.15, $CHCl_3$).

^{1}H NMR (600 MHz, $CDCl_3$) δ 7.95 – 7.83 (m, 2H), 7.54 (tt, *J* = 7.2, 1.3 Hz, 1H), 7.49 – 7.42 (m, 2H), 7.21 – 7.12 (m, 2H), 6.94 (td, *J* = 7.4, 1.1 Hz, 1H), 4.59 (s, 1H), 3.37 (dd, *J* = 16.0, 9.6 Hz, 1H), 3.09 – 2.89 (m, 2H), 2.75 (d, *J* = 16.0 Hz, 1H), 2.10 (q, *J* = 6.6 Hz, 2H), 1.53 (s, 9H).

^{13}C NMR (151 MHz, $CDCl_3$) δ 199.43, 152.58, 136.82, 133.02, 128.57, 128.05, 127.43, 122.60, 115.68, 80.97, 77.02, 60.41, 58.64, 33.86, 29.58, 28.42.

HRMS (ESI$^+$) $C_{22}H_{25}NO_3Na$ [M+Na]$^+$: found 374.1732, required 374.1727.

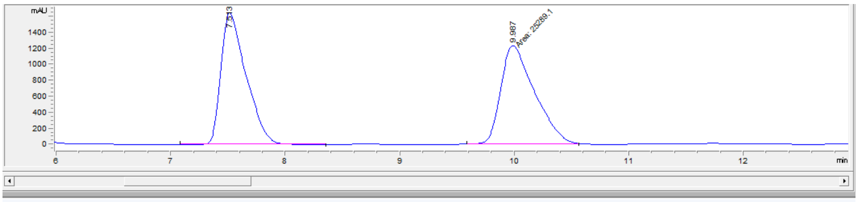

#	Time	Area	Height	Width	Area%	Symmetry
1	7.513	25024.1	1643.4	0.2217	49.737	0.508
2	9.987	25289.1	1228.1	0.3432	50.263	0.553

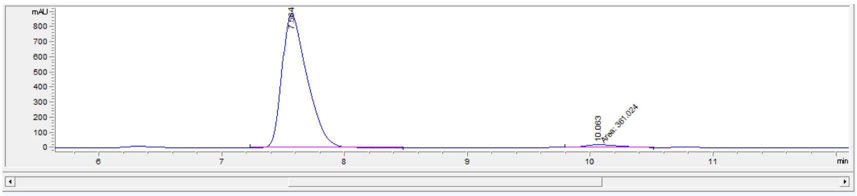

#	Time	Area	Height	Width	Area%	Symmetry
1	7.564	12511.5	883.5	0.2092	97.195	0.559
2	10.063	361	18.6	0.3236	2.805	0.65

***tert*-Butyl (3*S*)-3-(3-oxo-3-phenylpropyl)-3,4,4a,8a-tetrahydroisoquinoline-2(1H)-carboxylate (1j)**

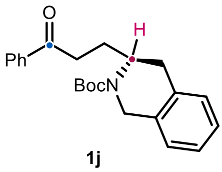

1j

Synthesized according to the general procedure **A** starting from enantiopure (3*R*)-2-(*tert*-butoxycarbonyl)-1,2,3,4,4a,8a-hexahydroisoquinoline-3-carboxylic acid (850 mg, 3.0 mmol, 1 equiv.) After the hydrogenation step, the crude mixture was purified by flash column chromatography on silica gel (10% EtOAc in hexane). Partial racemization was observed during the reaction sequence; consequently, the product was resolved by preparative chiral HPLC using a Chiralpak AD-H column (250 mm x 20 mm) to afford the title compound **1j** as a yellow oil (216 mg, 20% yield after preparative chiral HPLC). The enantiomeric ratio was determined by HPLC analysis on a chiral Daicel Chiralpak AD-H (90:10 hexane:*i*-PrOH, 1.0 mL/min, 30 °C, λ = 230 nm: τ_{major} = 9.7 min, τ_{minor} = 16.3 min), > 99.5: 0.5 e.r.; $[\alpha]^{D}_{20}$ = -49.9 (c = 0.16, $CHCl_3$).

^{1}H NMR (600 MHz, $CDCl_3$) (mixture of rotamers) δ 7.93 (d, *J* = 7.7 Hz, 2H), 7.54 (s, 1H), 7.44 (s, 2H), 7.23 – 7.04 (m, 4H), 4.87 (d, *J* = 83.7 Hz, 2H), 4.74 – 4.52 (m, 2H), 4.22 (s, 1H), 3.14 (dd, *J* = 15.9, 5.8 Hz, 1H), 3.04 (s, 1H), 2.93 (s, 1H), 2.71 (dd, *J* = 15.9, 1.9 Hz, 1H), 1.52 – 1.31 (m, 9H).

^{13}C NMR (151 MHz, $CDCl_3$) (mixture of rotamers) δ 199.67, 199.19, 155.29, 137.07, 133.26, 132.68, 129.38, 128.73, 128.71, 128.15, 126.68, 126.35, 80.03, 57.32, 49.20, 48.50, 42.99, 42.08, 35.68, 34.85, 34.04, 33.73, 28.51, 28.21, 26.34, 26.13.

HRMS (ESI$^+$) $C_{23}H_{27}NO_3Na$ [M+Na]$^+$: found 388.1887, required 388.1884.

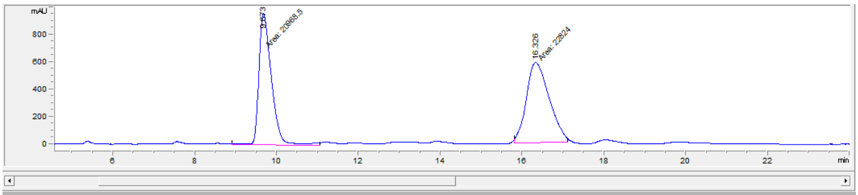

#	Time	Area	Height	Width	Area%	Symmetry
1	9.673	20968.5	962	0.3633	47.882	0.56
2	16.326	22824	586.1	0.649	52.118	0.672

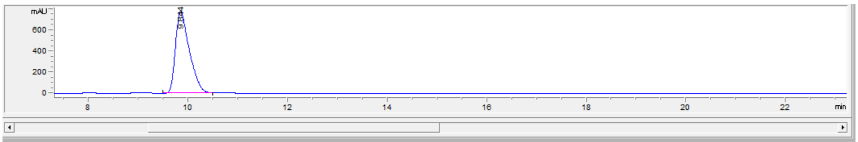

#	Time	Area	Height	Width	Area%	Symmetry
1	9.844	15159.3	779	0.289	100.000	0.551

tert-Butyl (S)-(2-oxo-2-(2-(3-oxo-3-phenylpropyl)pyrrolidin-1-yl)ethyl)carbamate (1k)

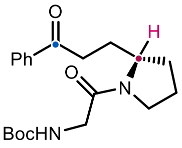

1k

Synthesized according to the general procedure **A** starting from enantiopure *N*-Boc-*L*-glycyl-*L*-proline (300 mg, 1.1 mmol, 1 equiv.). The title compound **1k** was obtained as a yellow oil (170 mg, 43% yield over 4 steps). The enantiomeric ratio was determined by HPLC analysis on a chiral Daicel Chiralpak IC-3 (80:20 hexane:i-PrOH, 2.0 mL/min, 30 °C, λ = 230 nm: τ_{minor} = 25.0 min, τ_{major} = 43.6 min), 97.5:2.5 e.r.; $[\alpha]^{D}{}_{20}$ = -8.8 (c = 0.16, $CHCl_3$).

^{1}H NMR (600 MHz, $CDCl_3$) (mixture of rotamers) δ 7.95 (tt, *J* = 6.8, 1.4 Hz, 2H), 7.61 – 7.49 (m, 1H), 7.46 (td, *J* = 7.1, 5.5 Hz, 2H), 5.46 (d, *J* = 35.6 Hz, 1H), 4.31 – 4.02 (m, 1H), 3.96 – 3.74 (m, 2H), 3.63 – 3.32 (m, 2H), 3.04 (dddd, *J* = 81.9, 17.2, 9.3, 5.8 Hz, 2H), 2.11 (dtt, *J* = 16.0, 10.8, 6.6 Hz, 2H), 2.02 – 1.89 (m, 2H), 1.88 – 1.73 (m, 2H), 1.44 (s, 9H).

^{13}C NMR (151 MHz, $CDCl_3$) (mixture of rotamers) δ 199.87, 167.47, 156.02, 136.96, 136.72, 133.46, 133.16, 128.84, 128.72, 128.22, 128.17, 79.74, 57.37, 45.78, 45.62, 43.31, 42.99, 38.76, 35.96, 35.17, 30.30, 29.83, 28.54, 28.52, 28.50, 23.91, 21.68.

HRMS (ESI^{+}) $C_{20}H_{28}N_2O_4Na$ [M+Na]$^{+}$: found 383.1945, required 383.1942.

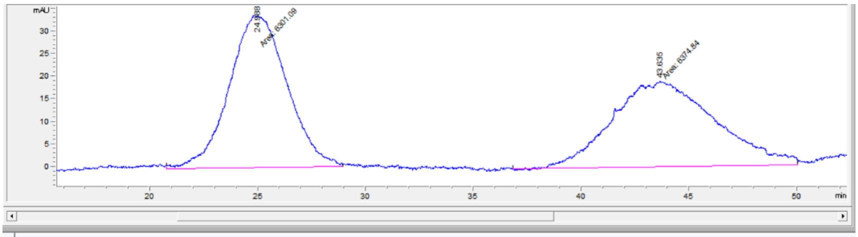

#	Time	Area	Height	Width	Area%	Symmetry
1	24.988	6301.1	33.7	3.1125	49.709	0.961
2	43.635	6374.8	19.1	5.5765	50.291	0.846

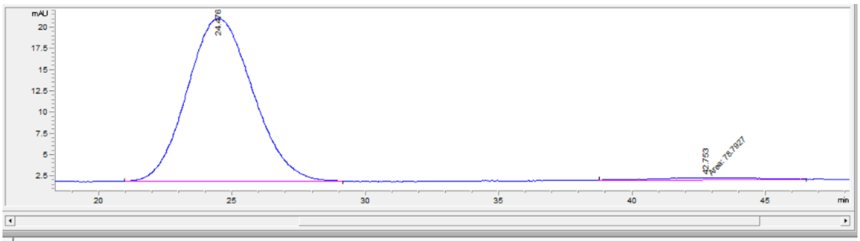

#	Time	Area	Height	Width	Area%	Symmetry
1	24.476	3281.4	19.2	2.0128	97.655	0.886
2	42.753	78.8	2.9E-1	4.5599	2.345	0.927

tert-Butyl ((_S_)-3-methyl-1-oxo-1-((S)-2-(3-oxo-3-phenylpropyl)pyrrolidin-1-yl)butan-2-yl)carbamate (1l)

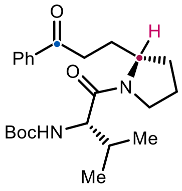

1l

Synthesized according to the general procedure **A** starting from enantiopure _N_-Boc-_L_-valyl-_L_-proline (300 mg, 1 mmol, 1 equiv.) The title compound **1l** was obtained as a clear oil (160 mg, 42% yield over 4 steps). A single detectable diastereomer was observed by ^{1}H NMR and HPLC analysis. $[\alpha]^{D}_{20}$ = -34.5 (c = 0.07, $CHCl_3$).

^{1}H NMR (600 MHz, $CDCl_3$) (mixture of rotamers) δ 8.03 – 7.92 (m, 2H), 7.55 (ddt, _J_ = 8.6, 7.0, 1.3 Hz, 1H), 7.49 – 7.42 (m, 2H), 5.27 (d, _J_ = 9.2 Hz, 1H), 4.27 (dd, _J_ = 9.2, 5.3 Hz, 2H), 3.73 – 3.65 (m, 1H), 3.59 – 3.40 (m, 2H), 3.15 – 2.95 (m, 3H), 2.13 – 2.01 (m, 3H), 2.00 – 1.91 (m, 3H), 1.82 (dddd, _J_ = 13.6, 9.7, 7.8, 5.7 Hz, 1H), 1.41 (d, _J_ = 18.1 Hz, 9H), 0.97 (dd, _J_ = 17.0, 6.7 Hz, 3H), 0.90 (d, _J_ = 6.8 Hz, 3H).

^{13}C NMR (151 MHz, $CDCl_3$) (mixture of rotamers) δ 206.96, 199.65, 171.32, 155.94, 136.85, 133.03, 128.59, 128.22, 128.05, 79.36, 60.41, 57.22, 56.90, 56.80, 46.89, 44.79, 35.95, 35.36, 32.57, 31.57, 30.94, 30.46, 29.69, 28.77, 28.51, 28.37, 28.35, 24.17, 21.48, 19.65, 19.42, 17.73, 17.27, 14.21.

HRMS (ESI^{+}) $C_{23}H_{34}N_2O_4Na$ [M+Na]$^{+}$: found 425.2417, required 425.2411.

tert-butyl (S)-2-(3-oxo-3-(pyridin-3-yl)propyl)pyrrolidine-1-carboxylate (1o)

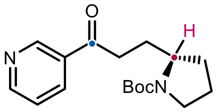

1o

Synthesized according to the general procedure **A** starting from enantiopure *N*-Boc-*L*-proline (500 mg, 2.3 mmol, 1 equiv.) The title compound **1l** was obtained as a clear oil (368 mg, 51% yield over 4 steps). The enantiomeric ratio was determined by SFC analysis on a chiral Daicel Chiralpak IB-N3 (90:10 CO_2: *i*-PrOH, 1.0 mL/min, 20 °C, λ = 210 nm: τ_{minor} = 2.3 min, τ_{major} = 2.6 min), 99.5:0.5 e.r.;

^{1}H NMR (600 MHz, $CDCl_3$) (mixture of rotamers) δ 9.15 (dd, *J* = 2.3, 0.9 Hz, 1H), 8.75 (s, 1H), 8.22 (ddd, *J* = 8.0, 2.3, 1.7 Hz, 1H), 7.40 (s, 1H), 3.92 (d, *J* = 29.6 Hz, 1H), 3.52 – 3.25 (m, 2H), 3.13 – 2.92 (m, 2H), 2.09 – 1.81 (m, 4H), 1.66 (s, 1H), 1.40 (s, 9H).

^{13}C NMR (151 MHz, $CDCl_3$) (mixture of rotamers) δ 153.39, 149.75, 135.50, 123.70, 56.68, 46.58, 36.17, 30.79, 29.10, 28.61, 23.86.

HRMS (ESI$^+$) $C_{17}H_{24}N_2O_3Na$ [M+Na]$^+$: found 327.1675, required 327.1679.

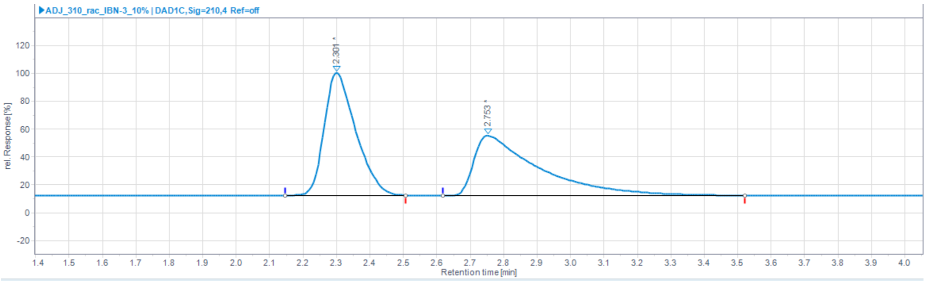

Injection Results

Peaks Summary

#	Signal descript...	RT (min)	Area (mAU·s)	Area%	Height (mAU)	Height%	Amount	Concentration	Start time (min)	End time (min)
1	DAD1C,Sig=210,4 ...	2.301	6164.602	49.699	939.934	67.06			2.148	2.508
2	DAD1C,Sig=210,4 ...	2.753	6239.286	50.301	461.647	32.94			2.619	3.523

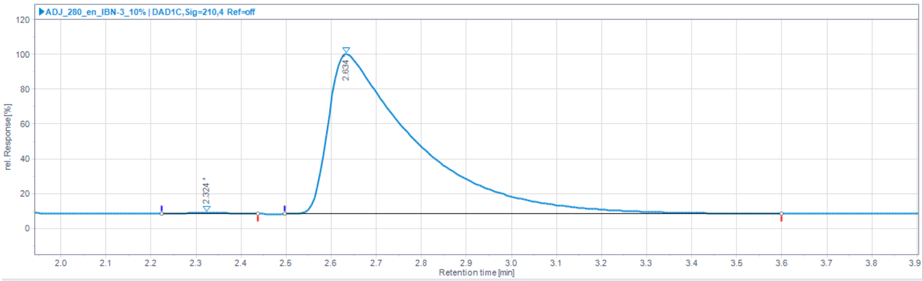

Injection Results

Peaks Summary

#	Signal descript...	RT (min)	Area (mAU·s)	Area%	Height (mAU)	Height%	Amount	Concentration	Start time (min)	End time (min)
1	DAD1C,Sig=210,4 ...	2.324	68.490	0.372	12.541	0.91			2.224	2.438
2	DAD1C,Sig=210,4 ...	2.634	18352.837	99.628	1371.173	99.09			2.497	3.600

(S)-5-oxo-5-phenylpentan-2-yl benzoate (5b)

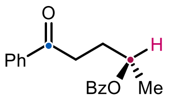

5b

Synthesized according to the general procedure **A** using enantiopure benzoyloxy-(*D*)-lactic acid (1 g, 5.5 mmol, 1 equiv.). The title compound **5b** was obtained as a pale yellow solid (533 mg, 37% yield over all 4 steps). The enantiomeric ratio was determined by HPLC analysis on a chiral Daicel Chiralpak IC-3 (95:5 hexane:*i*-PrOH, 1.0 mL/min, 30 °C, λ = 230 nm: τ_{minor} = 9.8 min, τ_{major} = 10.6 min), 99:1 e.r.; $[\alpha]^{D}{}_{20}$ = +70.5 (c = 0.07, $CHCl_3$)

^{1}H NMR (400 MHz, $CDCl_3$) δ 8.05 – 8.00 (m, 2H), 7.96 – 7.91 (m, 2H), 7.58 – 7.50 (m, 2H), 7.46 – 7.40 (m, 4H), 5.31 – 5.23 (m, 1H), 3.17 – 3.02 (m, 2H), 2.21 – 2.12 (m, 2H), 1.42 (d, *J* = 6.2 Hz, 3H).

^{13}C NMR (151 MHz, $CDCl_3$) δ 199.29, 166.18, 136.79, 133.08, 132.88, 130.56, 130.17, 129.55, 128.59, 128.48, 128.34, 128.03, 71.16, 34.63, 30.41, 20.36.

HRMS (ESI$^+$) $C_{18}H_{18}O_3Na$ [M+Na]$^+$: found 305.1147 , required 305.1149.

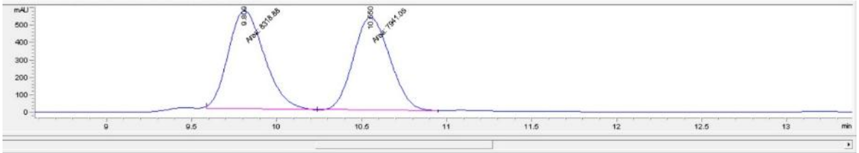

#	Time	Area	Height	Width	Area%	Symmetry
1	9.809	8318.9	562.3	0.2466	51.162	0.785
2	10.55	7941	531.2	0.2491	48.838	0.852

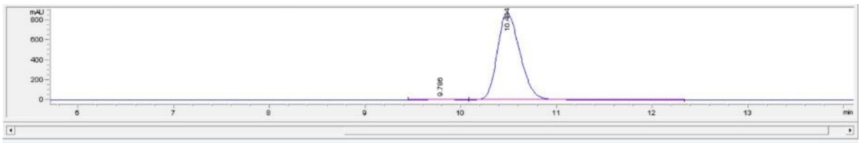

#	Time	Area	Height	Width	Area%	Symmetry
1	9.786	93.9	6.4	0.2268	0.652	0.887
2	10.484	14303.6	871	0.2556	99.348	0.736

tert-Butyl (*S*)-(5-oxo-5-phenylpentan-2-yl)carbamate (5c)

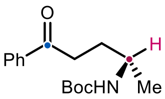

5c

Synthesized according to the general procedure **A** using enantiopure *N*-Boc-*L*-alanine (1 g, 5.3 mmol, 1 equiv.) The title compound **5c** was obtained as a white solid (1.03 g, 70% yield 4 steps). The enantiomeric ratio was determined by SFC analysis on a chiral Daicel Chiralpak IG-3 (95:5 CO_2:*i*-PrOH, 1.0 mL/min, 20 °C, λ = 210 nm: τ_{minor} = 8.8 min, τ_{major} = 13.8 min), 99:1 e.r.; $[\alpha]^{D}_{20}$ = +7.5 (c = 0.07, $CHCl_3$)

^{1}H NMR (600 MHz, $CDCl_3$) δ 7.98 – 7.93 (m, 2H), 7.58 – 7.53 (m, 1H), 7.46 (t, 2H), 4.39 (s, 1H), 3.75 (s, 1H), 3.05 (t, *J* = 7.3 Hz, 2H), 1.84 (s, 2H), 1.40 (s, 9H), 1.19 (d, *J* = 6.6 Hz, 3H).

^{13}C NMR (151 MHz, $CDCl_3$) δ 200.02, 155.54, 136.93, 133.03, 128.57, 128.05, 79.09, 65.54, 59.70, 46.52, 45.39, 35.49, 31.45, 28.44, 28.36, 21.76, 19.56.

HRMS (ESI$^+$) $C_{16}H_{23}NO_3Na$ [M+Na]$^+$: found 300.1571, required 300.1571.

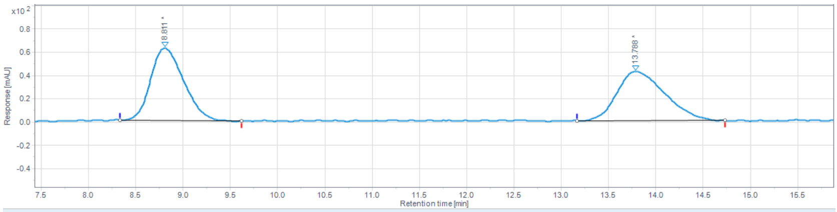

Injection Results

Peaks | Summary

#	Signal description	RT (min)	Area (mAU·s)	Area%	Height (mAU)	Height%	Amount	Concentration	Start time (min)	End time (min)
1	DAD1C,Sig=210,4 ...	8.811	1504.151	50.374	62.016	59.41			8.333	9.619
2	DAD1C,Sig=210,4 ...	13.788	1481.845	49.626	42.364	40.59			13.166	14.731

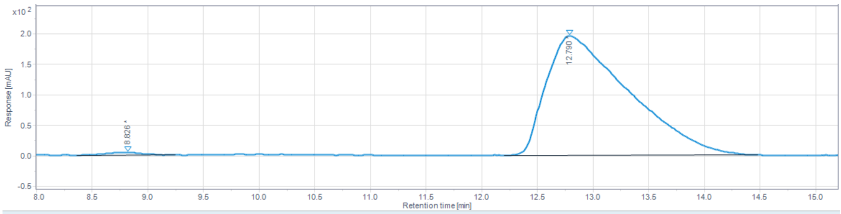

Injection Results

Peaks | Summary

#	Signal description	RT (min)	Area (mAU·s)	Area%	Height (mAU)	Height%	Amount	Concentration	Start time (min)	End time (min)
1	DAD1C,Sig=210,4 ...	8.826	93.657	0.920	4.135	2.07			8.363	9.252
2	DAD1C,Sig=210,4 ...	12.790	10083.467	99.080	196.056	97.93			12.202	14.484

tert-Butyl (*R*)-(2-methyl-6-oxo-6-phenylhexan-3-yl)carbamate (5d)

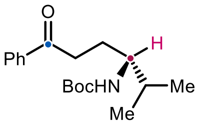

5d

Synthesized according to the general procedure **A** using enantiopure *N*-Boc-*L*-valine (850 mg, 4 mmol, 1 equiv.). The title compound **5d** was obtained as a white solid (938 mg, 78% yield over 4 steps). The enantiomeric ratio was determined by SFC analysis on a chiral Daicel Chiralpak IB-N3 (95:5 CO_2:*i*-PrOH, 1.0 mL/min, 20 °C, λ = 210 nm: τ_{minor} = 6.1 min, τ_{major} = 18.3 min), 99:1 e.r.; $[\alpha]^D_{20}$ = +3.8 (c = 0.16, $CHCl_3$).

^{1}H NMR (600 MHz, $CDCl_3$) δ 7.98 – 7.93 (m, 2H), 7.55 (t, *J* = 7.3 Hz, 1H), 7.45 (t, *J* = 7.6 Hz, 2H), 4.33 (d, 1H), 3.51 (m, 1H), 3.05 (t, *J* = 7.3 Hz, 2H), 1.94 (m, 1H), 1.81 – 1.70 (m, 2H), 0.95 (d, *J* = 6.8 Hz, 3H), 0.92 (d, *J* = 6.8 Hz, 3H).

^{13}C NMR (151 MHz, $CDCl_3$) δ 200.36, 156.12, 137.02, 132.99, 128.55, 128.34, 128.06, 78.97, 55.69, 35.77, 32.80, 30.95, 28.33, 26.72, 19.08, 17.89.

HRMS (ESI$^+$) $C_{18}H_{27}NO_3Na$ [M+Na]$^+$: found 328.1882, required 328.1884.

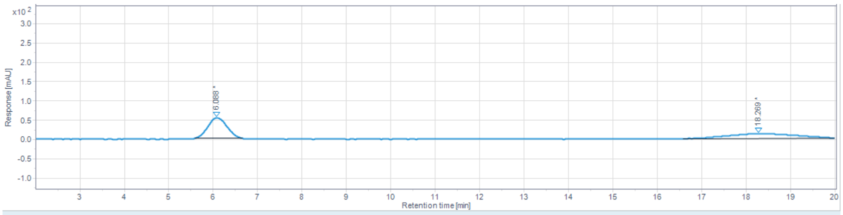

Injection Results

Peaks | Summary

#	Signal description	RT (min)	Area (mAU·s)	Area%	Height (mAU)	Height%	Amount	Concentration	Start time (min)	End time (min)
1	DAD1C,Sig=210,4 ...	6.088	1565.459	55.276	52.709	81.27			5.597	6.655
2	DAD1C,Sig=210,4 ...	18.269	1266.633	44.724	12.149	18.73			16.590	19.995

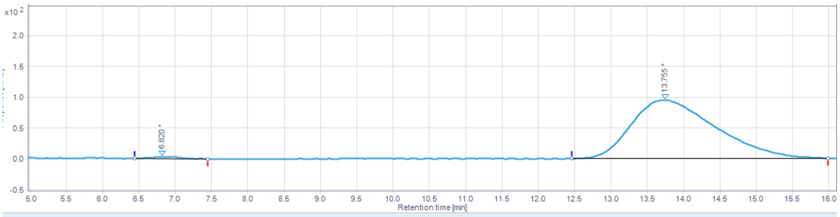

Injection Results

Peaks | Summary

#	Signal description	RT (min)	Area (mAU·s)	Area%	Height (mAU)	Height%	Amount	Concentration	Start time (min)	End time (min)
1	DAD1C,Sig=210,4 ...	6.820	91.096	1.182	3.397	3.46			6.441	7.447
2	DAD1C,Sig=210,4 ...	13.755	7615.339	98.818	94.758	96.54			12.460	15.987

***tert*-Butyl (*R*)-(6-methyl-1-oxo-1-phenylheptan-4-yl)carbamate (5e)**

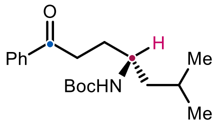

5e

Synthesized according to the general procedure **A** using enantiopure *N*-Boc-*L*-leucine (500 mg, 2.2 mmol, 1 equiv.) The title compound **5e** was obtained as a white solid (583 mg, 84% yield over 4 steps). The enantiomeric ratio was determined by SFC analysis on a chiral Daicel Chiralpak IG-3 (95:5 CO_2:*i*-PrOH, 1.0 mL/min, 20 °C, λ = 210 nm: τ_{minor} = 7.0 min, τ_{major} = 14.7 min), 96:4 e.r.; $[\alpha]^D_{20}$ = -9.0 (c = 0.07, $CHCl_3$).

^{1}H NMR (600 MHz, $CDCl_3$)δ 7.98 – 7.92 (m, 2H), 7.55 (t, *J* = 7.3 Hz, 1H), 7.45 (t, *J* = 7.6 Hz, 2H), 4.23 (d, *J* = 9.6 Hz, 1H), 3.78 – 3.63 (m, 1H), 3.06 (t, *J* = 7.4 Hz, 2H), 2.05 – 1.91 (m, 1H), 1.72 (m, 2H), 1.38 (s, 9H), 1.35 – 1.23 (m, 2H), 0.93 (d, *J* = 6.6, 6H).

^{13}C NMR (151 MHz, $CDCl_3$) δ 200.24, 155.76, 137.00, 132.96, 128.60, 128.54, 128.06, 78.96, 48.79, 45.48, 35.33, 31.59, 30.39, 28.33, 24.97, 23.02, 22.66, 22.34, 14.13.

HRMS (ESI$^+$) $C_{19}H_{29}NO_3Na$ [M+Na]$^+$: found 342.2041, required 342.2040.

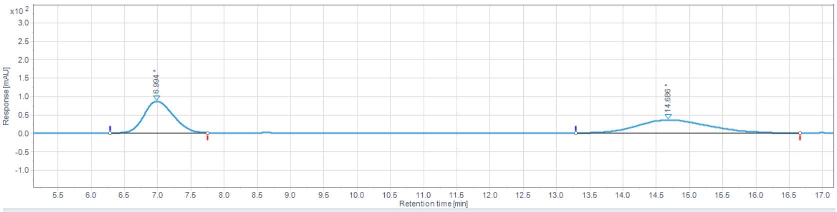

Injection Results

Peaks | Summary

#	Signal description	RT (min)	Area (mAU·s)	Area%	Height (mAU)	Height%	Amount	Concentration	Start time (min)	End time (min)
1	DAD1C,Sig=210,4 ...	6.994	2615.804	50.521	85.630	70.82			6.288	7.759
2	DAD1C,Sig=210,4 ...	14.686	2561.902	49.479	35.283	29.18			13.291	16.665

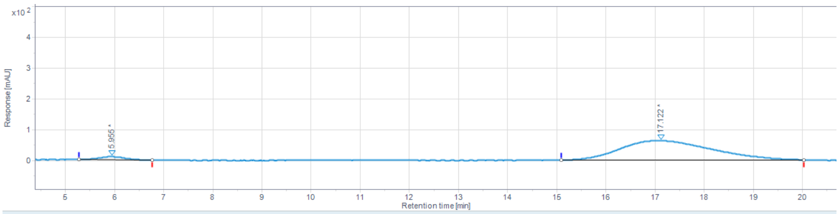

Injection Results

Peaks | Summary

#	Signal description	RT (min)	Area (mAU·s)	Area%	Height (mAU)	Height%	Amount	Concentration	Start time (min)	End time (min)
1	DAD1C,Sig=210,4 ...	5.955	357.647	4.230	10.170	13.88			5.284	6.775
2	DAD1C,Sig=210,4 ...	17.122	8096.872	95.770	63.123	86.12			15.104	20.036

tert-Butyl (_R_)-(1-hydroxy-5-oxo-5-phenylpentan-2-yl)carbamate (5f)

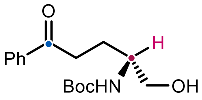

5f

Synthesized according to the general procedure **A** using enantiopure *N*-Boc-*L*-serine (600 mg, 3 mmol, 1 equiv.) After the hydrogenation step the crude mixture was purified by flash column chromatography on silica gel (15% EtOAc in hexane) to afford the title compound **5f** as a white solid (527 mg, 60% yield over 4 steps). The enantiomeric ratio was determined by HPLC analysis on a chiral Daicel Chiralpak AD-H (90:10 hexane:*i*-PrOH, 1.0 mL/min, 30 °C, λ = 230 nm: τ_{minor} = 15.4 min, τ_{major} = 19.6 min), 98:2 e.r.; $[\alpha]^{D}_{20}$ = +39.0 (c = 0.07, $CHCl_3$).

^{1}H NMR (600 MHz, $CDCl_3$) δ 8.00 – 7.91 (m, 2H), 7.57 (td, *J* = 7.3, 1.3 Hz, 1H), 7.47 (t, *J* = 7.8 Hz, 2H), 4.99 – 4.78 (m, 1H), 3.76 – 3.66 (m, 2H), 3.63 – 3.54 (m, 1H), 3.11 (td, *J* = 6.9, 2.5 Hz, 2H), 2.62 (d, *J* = 7.9 Hz, 1H), 2.08 – 1.99 (m, 1H), 1.93 (q, *J* = 7.2 Hz, 1H), 1.41 (s, 9H).

^{13}C NMR (151 MHz, $CDCl_3$) δ 200.19, 156.33, 136.70, 133.28, 128.64, 128.09, 79.61, 65.31, 52.61, 35.18, 28.33, 25.15.

HRMS (ESI^{+}) $C_{16}H_{23}NO_4Na$ [M+Na]$^{+}$: found 316.1519, required 316.1520.

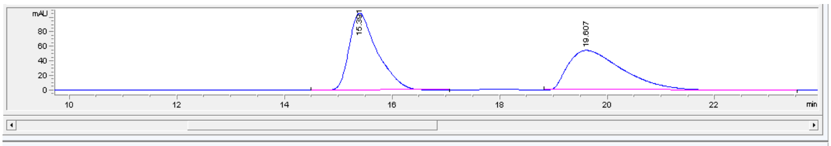

#	Time	Area	Height	Width	Area%	Symmetry
1	15.391	3797.3	105.4	0.5282	49.804	0.549
2	19.607	3827.2	54.5	1.0999	50.196	0.472

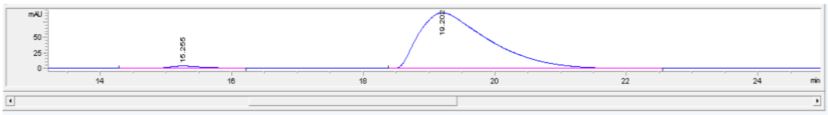

#	Time	Area	Height	Width	Area%	Symmetry
1	15.255	136.8	3.8	0.5165	2.008	0.717
2	19.202	6675.8	90.6	1.0955	97.992	0.472

tert-Butyl (_R_)-(5-oxo-1,5-diphenylpentan-2-yl)carbamate (5g)

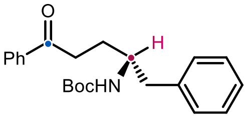

5g

Synthesized according to the general procedure **A** using enantiopure *N*-Boc-*L*-phenylalanine (800 mg, 3 mmol, 1 equiv.) The title compound **5g** was obtained as a white solid (573 mg, 54% yield over 4 steps). The enantiomeric ratio was determined by HPLC analysis on a chiral Daicel Chiralpak AD-H (90:10 hexane: *i*-PrOH, 1.0 mL/min, 30 °C, λ = 210 nm: τ_{minor} = 8.1 min, τ_{major} = 12.8 min), 85.5:14.5 e.r.; $[\alpha]^{D}_{20}$ = +14.4 (c = 0.08, $CHCl_3$).

^{1}H NMR (600 MHz, $CDCl_3$) δ 7.95 – 7.90 (m, 2H), 7.57 – 7.51 (m, 1H), 7.44 (t, 2H), 7.29 (m, , 2H), 7.22 (t, 3H), 4.39 (d, 1H), 3.90 (s, 1H), 3.04 (m, 2H), 2.88 (dd, *J* = 13.9, 5.9 Hz, 1H), 2.79 (dd, *J* = 13.3, 7.0 Hz, 1H), 1.96 (s, 1H), 1.80 (s, 1H), 1.35 (s, 9H).

^{13}C NMR (151 MHz, $CDCl_3$) δ 200.06, 155.61, 137.94, 136.91, 133.04, 129.49, 128.56, 128.42, 128.03, 126.41, 79.19, 51.75, 42.10, 35.51, 28.51, 28.31.

HRMS (ESI$^+$) $C_{22}H_{27}NO_3Na$ [M+Na]$^+$: found 376.1884, required 376.1884.

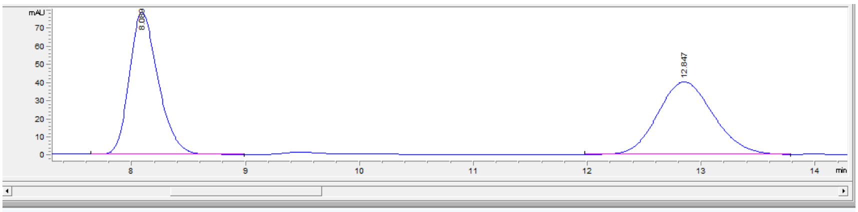

#	Time	Area	Height	Width	Area%	Symmetry
1	8.089	1369.1	78.2	0.2682	50.227	0.74
2	12.847	1356.7	40	0.53	49.773	0.869

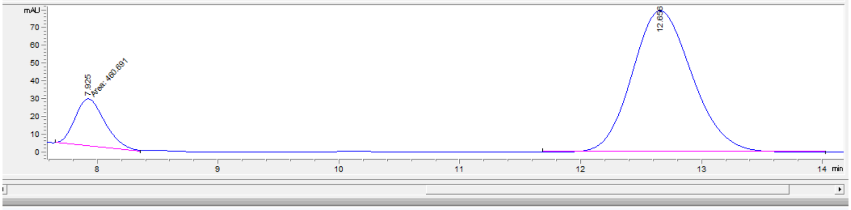

#	Time	Area	Height	Width	Area%	Symmetry
1	7.925	460.7	26.7	0.2874	14.372	0.749
2	12.656	2744.8	79.7	0.534	85.628	0.845

tert-Butyl (*R*)-(1-(methylthio)-6-oxo-6-phenylhexan-3-yl)carbamate (5h)

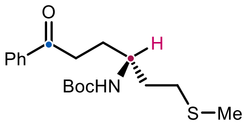

5h

Synthesized according to the general procedure **A** using enantiopure *N*-Boc-*L*-methionine (1 g, 4 mmol, 1 equiv.) The title compound **5h** was obtained as a grey solid (610 mg, 45% yield over 4 steps). The enantiomeric ratio was determined by HPLC analysis on a chiral Daicel Chiralpak IC-3 (97:3 hexane:*i*-PrOH, 1.0 mL/min, 30 °C, λ = 230 nm: τ_{major} = 52.9 min, τ_{minor} = 56.9 min), 97.5:2.5 e.r.; $[\alpha]^{D}_{20}$ = +4.8 (c = 0.08, $CHCl_3$).

^{1}H NMR (600 MHz, $CDCl_3$) δ 7.98 – 7.92 (m, 2H), 7.56 (t, *J* = 7.4 Hz, 1H), 7.46 (t, *J* = 7.6 Hz, 2H), 4.39 (d, 1H), 3.80 – 3.70 (m, 1H), 3.07 (t, *J* = 7.1 Hz, 2H), 2.60 – 2.51 (m, 2H), 2.11 (s, 3H), 2.01 – 1.93 (m, 1H), 1.84 (m, 2H), 1.72 (m, 1H), 1.38 (s, 9H).

^{13}C NMR (151 MHz, $CDCl_3$) δ 199.95, 155.78, 136.89, 133.10, 128.59, 128.05, 79.27, 50.22, 35.76, 35.32, 30.72, 29.55, 28.42, 28.31, 15.66.

HRMS (ESI$^+$) $C_{18}H_{27}NO_3SNa$ [M+Na]$^+$: found 360.1607, required 360.1604.

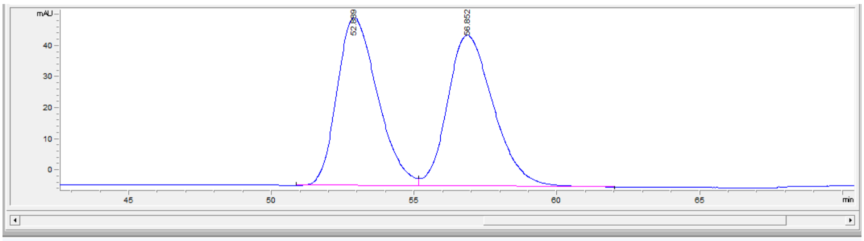

#	Time	Area	Height	Width	Area%	Symmetry
1	52.889	5340.1	54	1.5048	49.547	0.71
2	56.852	5437.8	48.7	1.6672	50.453	0.732

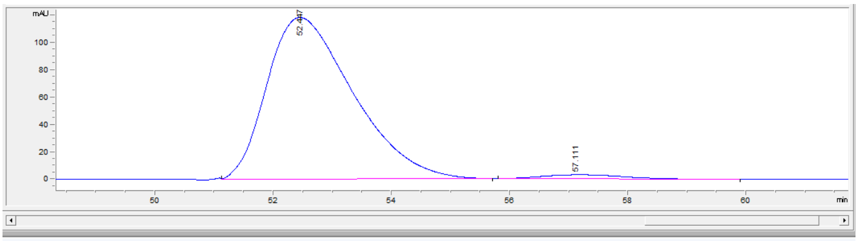

#	Time	Area	Height	Width	Area%	Symmetry
1	52.447	12059.3	118.6	1.546	97.610	0.591
2	57.111	295.3	3	1.1503	2.390	0.642

tert-Butyl (8-oxo-8-phenyloctane-1,5-diyl)(S)-dicarbamate (5i)

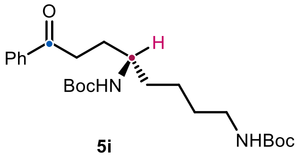

5i

Synthesized according to the general procedure **A** using enantiopure $N_{\alpha,\varepsilon}$-Bis-Boc-*L*-lysine (1 g, 2.9 mmol, 1 equiv.) The title compound **5i** was obtained as a pale yellow solid (840 mg, 67% yield over 4 steps). The enantiomeric ratio was determined by HPLC analysis on a chiral Daicel Chiralpak AD-H (90:10 hexane:*i*-PrOH, 1 mL/min, 30 °C, λ = 230 nm: τ_{minor} = 15.7 min, τ_{major} = 42.0 min), 94.5:5.5 e.r.; $[\alpha]^{D}_{20}$ = -18.0 (c = 0.07, $CHCl_3$).

^{1}H NMR (600 MHz, $CDCl_3$) δ 8.02 – 7.92 (m, 2H), 7.59 – 7.50 (m, 1H), 7.48 – 7.42 (m, 2H), 4.58 (s, 1H), 4.33 (d, *J* = 9.5 Hz, 1H), 3.70 – 3.58 (m, 1H), 3.18 – 2.98 (m, 4H), 2.02 – 1.89 (m, 1H), 1.83 – 1.72 (m, 1H), 1.40 (dd, *J* = 33.8, 6.6 Hz, 24H).

^{13}C NMR (151 MHz, $CDCl_3$) δ 200.10, 156.05, 155.94, 136.96, 133.02, 128.72, 128.57, 128.48, 128.43, 128.05, 127.99, 79.12, 79.03, 50.49, 40.35, 37.49, 35.78, 35.57, 35.39, 30.94, 29.80, 29.70, 28.44, 28.35, 28.11, 23.11, 22.97.

HRMS (ESI$^+$) $C_{24}H_{38}N_2O_5Na$ [M+Na]$^+$: found 457.2677, required 457.2673.

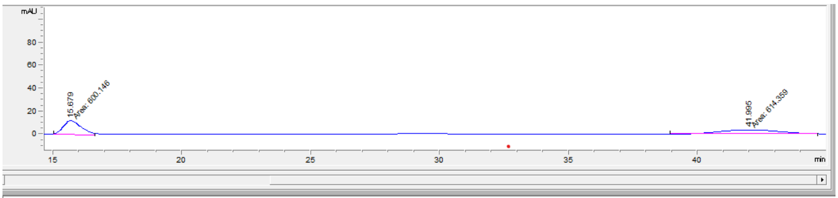

#	Time	Area	Height	Width	Area%	Symmetry
1	15.679	600.1	12.2	0.82	49.415	0.684
2	41.995	614.4	3.7	2.7433	50.585	0.98

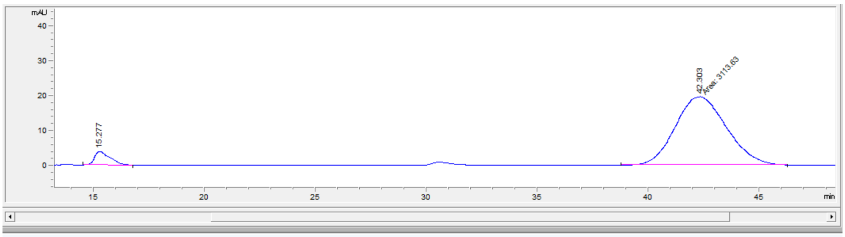

#	Time	Area	Height	Width	Area%	Symmetry
1	15.277	185.6	3.9	0.6319	5.624	0.467
2	42.303	3113.6	19.5	2.6576	94.376	0.906

tert-Butyl (_R_)-4-(2-((tert-butoxycarbonyl)amino)-5-oxo-5-phenylpentyl)-1H-imidazole-1-carboxylate (5j)

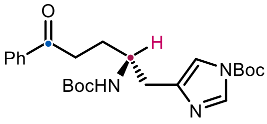

5j

Synthesized according to the general procedure **A** using enantiopure _N,N_-bis(_tert_-butoxycarbonyl)-_L_-histidine (890 mg, 2.5 mmol, 1 equiv.). The title compound **5j** as a white solid (420 mg, 38% yield over 4 steps). The enantiomeric ratio was determined by HPLC analysis on a chiral Daicel Chiralpak AD-H (80:20 hexane:_i_-PrOH, 1.0 mL/min, 30 °C, λ = 230 nm:, τ_{major} = 28.1 min, τ_{minor} = 40.2 min), 90:10 e.r.; $[\alpha]^{D}_{20}$ = +26.5 (c = 0.20, $CHCl_3$).

^{1}H NMR (600 MHz, $CDCl_3$) δ 8.00 (d, _J_ = 1.3 Hz, 1H), 7.96 – 7.90 (m, 2H), 7.53 (t, _J_ = 7.4 Hz, 1H), 7.43 (t, _J_ = 7.6 Hz, 2H), 7.17 – 7.15 (m, 1H), 5.23 (d, _J_ = 9.2 Hz, 1H), 3.97 (dq, _J_ = 10.0, 4.9 Hz, 1H), 3.07 (dt, _J_ = 8.3, 6.1 Hz, 2H), 2.83 (dd, _J_ = 14.8, 5.1 Hz, 1H), 2.79 – 2.69 (m, 1H), 1.92 (tt, _J_ = 12.6, 6.6 Hz, 1H), 1.83 – 1.76 (m, 1H), 1.60 (s, 9H), 1.38 (s, 9H).

^{13}C NMR (151 MHz, $CDCl_3$) δ 200.20, 155.92, 147.15, 140.19, 137.05, 136.84, 133.10, 128.66, 128.19, 114.60, 85.54, 79.15, 50.10, 35.66, 33.56, 29.17, 28.47, 28.03.

HRMS (ESI$^+$) $C_{24}H_{33}N_3O_5Na$ [M+Na]$^+$: found 466.2316, required 466.2313.

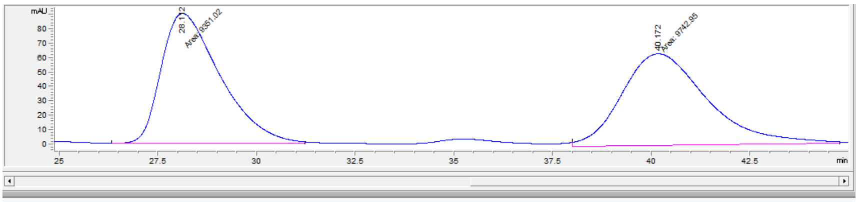

#	Time	Area	Height	Width	Area%	Symmetry
1	28.112	9351	89.9	1.7338	48.974	0.543
2	40.172	9742.9	63.8	2.5461	51.026	0.721

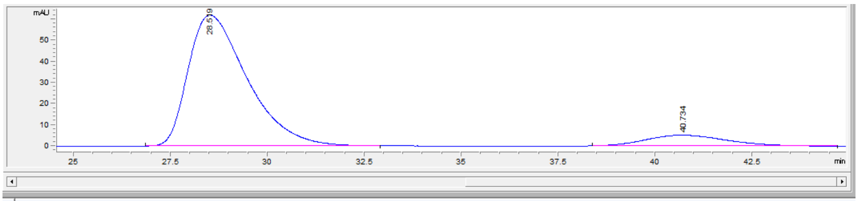

#	Time	Area	Height	Width	Area%	Symmetry
1	28.519	6550.6	62	1.5491	89.822	0.549
2	40.734	742.3	5.1	1.7122	10.178	0.841

***tert*-Butyl (*S*)-ethyl(5-oxo-5-phenylpentan-2-yl)carbamate (5k)**

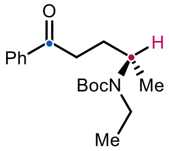

5k

Synthesized according to the general procedure **A** using enantiopure *N*-Boc-*N*-ethyl-*L*-Alanine (1 g, 4.6 mmol, 1 equiv.). The title compound **5k** was obtained as a yellow oil (590 mg, 42% yield over 4 steps). The enantiomeric ratio was determined by HPLC analysis on a chiral Daicel Chiralpak OD-H (95:5 hexane:*i*-PrOH, 1.0 mL/min, 30 °C, λ = 230 nm: τ_{minor} = 7.0 min, τ_{major} = 7.7 min), 96:4 e.r.; $[\alpha]^{D}_{20}$ = -7.2 (c = 0.13, $CHCl_3$).

^{1}H NMR (400 MHz, $CDCl_3$) δ 8.00 – 7.91 (m, 2H), 7.54 (dd, *J* = 9.0, 5.6 Hz, 1H), 7.51 – 7.39 (m, 2H), 4.13 (d, *J* = 114.5 Hz, 1H), 3.25 – 2.83 (m, 4H), 2.05 – 1.76 (m, 2H), 1.41 (s, 9H), 1.30 – 1.02 (m, 6H).

^{13}C NMR (151 MHz, $CDCl_3$) δ 200.0, 137.0, 136.9, 133.0, 128.6, 128.6, 128.0, 128.0, 79.0, 50.5, 38.4, 36.9, 35.7, 29.0, 28.5, 23.9, 20.0, 19.5, 15.8.

HRMS (ESI$^+$) $C_{18}H_{27}NO_3Na$ [M+Na]$^+$: found 328.1886, required 328.1884.

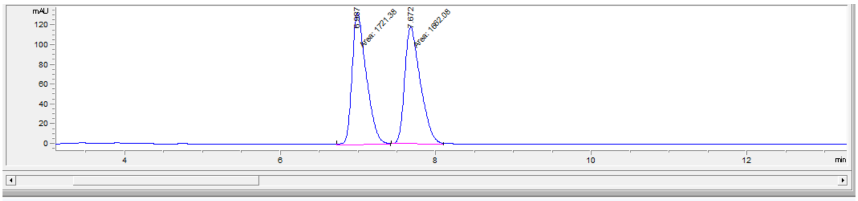

#	Time	Area	Height	Width	Area%	Symmetry
1	6.987	1721.4	133.5	0.2149	50.876	0.588
2	7.672	1662.1	118.2	0.2344	49.124	0.583

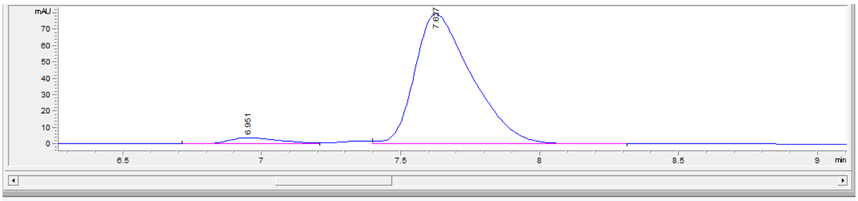

#	Time	Area	Height	Width	Area%	Symmetry
1	6.951	49	3.9	0.1864	4.151	0.555
2	7.627	1132.2	79.6	0.2099	95.849	0.569

In selected cases, enantioenriched substrates were prepared through a racemic single-step synthesis followed by preparative chiral HPLC resolution, as described in General Procedure B.

General procedure **B**:

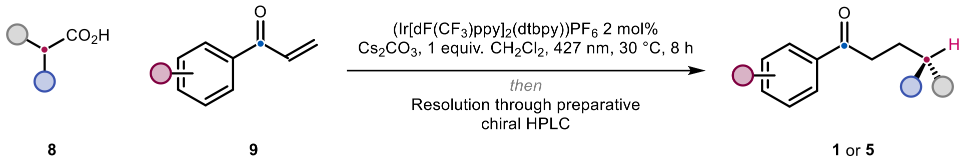

An oven-dried 4 mL vial equipped with a magnetic stir bar was charged with carboxylic acid **8** (0.10 mmol, 1.0 equiv.), (Ir[dF(CF_3)ppy]$_2$(dtbpy))PF_6 (2.3 mg, 2 mol%), and Cs_2CO_3 (32.6 mg, 0.10 mmol, 1.0 equiv.). The vial was sealed with a septum, evacuated, and backfilled with N_2 (two cycles). CH_2Cl_2 (1 mL) was then added under a nitrogen atmosphere, and the reaction mixture was degassed by bubbling N_2 for 5 min. Phenyl vinyl ketone **9** (2.0 equiv.) was added under nitrogen, and the mixture was irradiated overnight at 427 nm. Upon completion of the reaction, the solvent was removed under reduced pressure and the crude racemic ketone **1** or **5** was purified by flash column chromatography. The resulting racemate was subsequently resolved by preparative chiral HPLC to afford the individual enantiomers.

(+)-*tert*-Butyl-2-(3-oxo-3-(4-(trifluoromethyl)phenyl)propyl)pyrrolidine-1-carboxylate (1m)

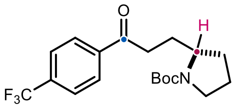

1m

Synthesized according to the general procedure **B** starting from Boc-proline (43 mg, 0.2 mmol, 1 equiv.) and vinyl ketone **9c** (80 mg, 0.4 mmol, 2 equiv.). The crude mixture was purified by flash column chromatography on silica gel (5% EtOAc in hexane) to afford the racemic title compound **1m** as a pale yellow oil. The product was resolved by preparative chiral HPLC to afford the title compound **1m** as a yellow oil using a Chiralpak AD-H column, 250 mm x 20 mm (32 mg, 43% yield after preparative chiral HPLC). The enantiomeric ratio was determined by HPLC analysis on a chiral Daicel Chiralpak AD-H (85:15 hexane:*i*-PrOH, 1.0 mL/min, 30 °C, λ = 280 nm: τ_{major} = 4.6 min, τ_{minor} = 9.5 min), > 99.5: 0.5 e.r.; $[\alpha]^{D}_{20}$ = +12.8 (c = 0.16, $CHCl_3$).

^{1}H NMR (600 MHz, $CDCl_3$) δ 7.97 – 7.93 (m, 2H), 7.91 – 7.87 (m, 2H), 7.57 (ddt, *J* = 7.8, 7.0, 1.3 Hz, 1H), 7.49 – 7.44 (m, 2H), 6.64 – 6.60 (m, 2H), 3.96 – 3.90 (m, 1H), 3.85 (s, 3H), 3.49 (ddd, *J* = 9.8, 8.1, 2.4 Hz, 1H), 3.26 (td, *J* = 9.3, 6.9 Hz, 1H), 3.07 – 3.01 (m, 2H), 2.26 – 2.18 (m, 1H), 2.15 – 2.08 (m, 1H), 2.07 – 1.98 (m, 2H), 1.89 (ddt, *J* = 11.4, 4.7, 3.4 Hz, 1H), 1.74 (dddd, *J* = 14.1, 9.9, 7.4, 6.0 Hz, 1H).

^{13}C NMR (151 MHz, $CDCl_3$) δ 199.46, 167.58, 150.37, 136.78, 133.18, 131.44, 128.68, 128.03, 116.60, 111.15, 57.70, 51.44, 48.17, 35.29, 29.96, 26.93, 23.20.

HRMS (ESI$^+$) $C_{19}H_{24}F_3NO_3Na$ [M+Na]$^+$: found 394.1600, required 394.1601.

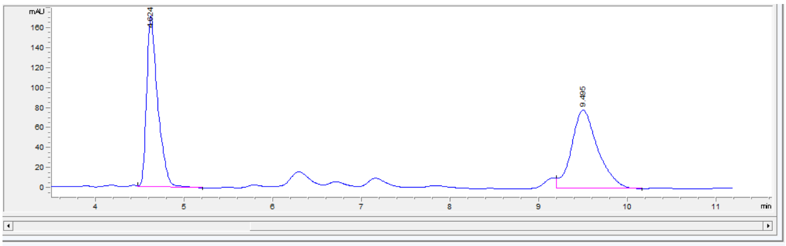

#	Time	Area	Height	Width	Area%	Symmetry
1	4.624	1499.1	170.9	0.1283	48.159	0.551
2	9.495	1613.8	78.4	0.3062	51.841	0.748

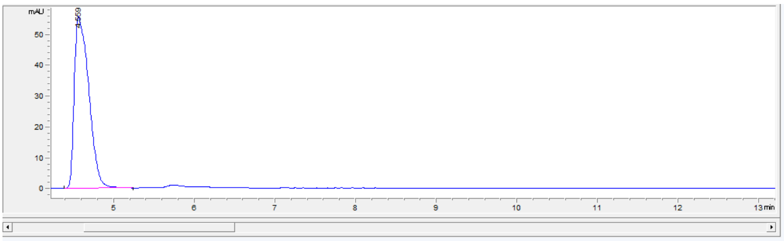

#	Time	Area	Height	Width	Area%	Symmetry
1	4.559	694.5	55.8	0.1672	100.000	0.379

(+)-*tert*-Butyl-2-(3-(4-methoxyphenyl)-3-oxopropyl)pyrrolidine-1-carboxylate (1n)

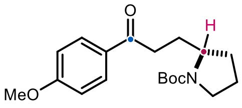

1n

Synthesized according to the general procedure **B** starting from Boc-Proline (43 mg, 0.2 mmol, 1 equiv.) and vinyl ketone **9b** (78 mg, 0.4 mmol, 2 equiv.). The crude mixture was purified by flash column chromatography on silica gel (20% EtOAc in hexane) to afford the racemic title compound **1n** as a yellow oil. The product was resolved by preparative chiral HPLC to afford the title compound **1n** as a yellow oil using a Chiralpak AD-H column, 250 mm x 20 mm (25 mg, 36% yield after preparative chiral HPLC). The enantiomeric ratio was determined by HPLC analysis on a chiral Daicel Chiralpak AD-H (85:15 hexane:*i*-PrOH, 1.0 mL/min, 30 °C, λ = 280 nm: τ_{major} = 9.0 min, τ_{minor} = 13.0 min), > 99.5: 0.5 e.r.; $[\alpha]^{D}_{20}$ = +8.5 (c = 0.75, $CHCl_3$).

^{1}H NMR (600 MHz, $CDCl_3$) (mixture of rotamers) δ 7.97 – 7.89 (m, 2H), 6.91 (dd, *J* = 8.7, 2.1 Hz, 2H), 3.85 (m+s, 4H), 3.37 (d, *J* = 56.0 Hz, 2H), 2.90 (s, 2H), 2.06 – 1.76 (m, 5H), 1.66 (s, 1H), 1.41 (s, 9H).

^{13}C NMR (151 MHz, $CDCl_3$) (mixture of rotamers) δ 163.42, 154.88, 130.35, 113.70, 79.28, 56.85, 55.47, 53.43, 46.42, 46.12, 35.56, 35.08, 30.41, 29.47, 28.52, 23.75, 23.05.

HRMS (ESI^{+}) $C_{20}H_{30}NO_4Na$ [M+Na]$^{+}$: found 371.2066, required 371.2068.

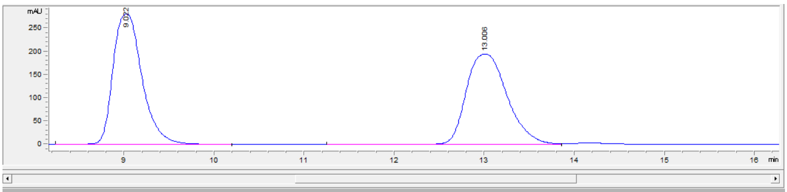

#	Time	Area	Height	Width	Area%	Symmetry
1	9.022	6287.8	280.9	0.3492	50.565	0.772
2	13.006	6147.4	194.8	0.4961	49.435	0.791

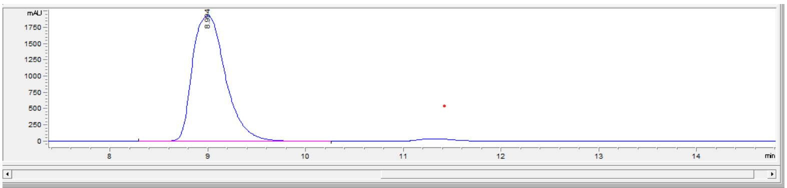

#	Time	Area	Height	Width	Area%	Symmetry
1	8.994	45342.1	1934.2	0.3735	100.000	0.775

(S)-**1,4-Diphenylpentan-1-one** (5a)

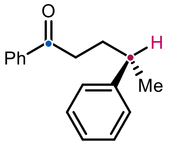

5a

Synthesized according to the general procedure **B** starting from 2-phenylpropanoic acid (0.12 g, 0.8 mmol, 1 equiv.) The crude mixture was purified by flash column chromatography on silica gel (CH_2Cl_2) to afford the title compound **5a** as a colourless oil. The product was resolved by preparative chiral HPLC using a (*S*,*S*)-Whelk-O2 10µm (250 × 10 mm) to afford the title compound **5a** (76 mg, 40% yield after preparative chiral HPLC). The enantiomeric ratio was determined by HPLC analysis on a chiral (*S*,*S*)-Whelk-O2 column (95:5 hexane:i-PrOH, 1.0 mL/min, 30 °C, λ = 254 nm: τ_{minor} = 13.3 min, τ_{major} = 16.2 min,), >99.5:0.5 e.r.; $[\alpha]^{D}_{20}$ = +36.7 (c = 0.28, $CHCl_3$). Absolute configuration was assigned based on optical rotation reported in literature.[3]

1H NMR (600 MHz, CDCl3) δ 7.91 – 7.79 (m, 2H), 7.61 – 7.47 (m, 1H), 7.45 – 7.37 (m, 2H), 7.37 – 7.27 (m, 2H), 7.23 – 7.16 (m, 3H), 2.87 (ddd, J = 16.9, 9.4, 6.3 Hz, 1H), 2.83 – 2.73 (m, 2H), 2.15 – 2.04 (m, 1H), 2.04 – 1.93 (m, 1H), 1.32 (d, J = 6.9 Hz, 3H).

13C NMR (151 MHz, CDCl3) δ 200.50, 146.69, 137.13, 133.03, 128.65, 128.15, 127.23, 126.34, 39.68, 36.86, 32.63, 22.73.

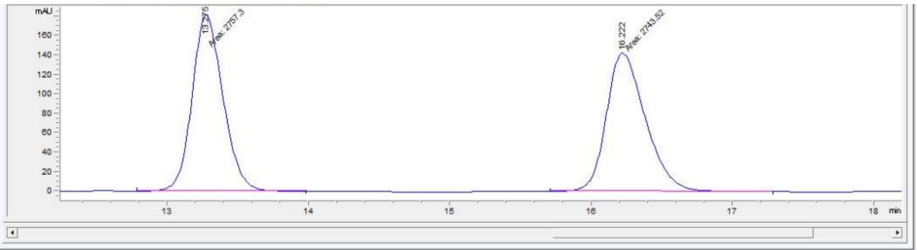

#	Time	Area	Height	Width	Area%	Symmetry
1	13.275	2757.3	180.9	0.254	50.125	0.803
2	16.222	2743.5	142.2	0.3217	49.875	0.684

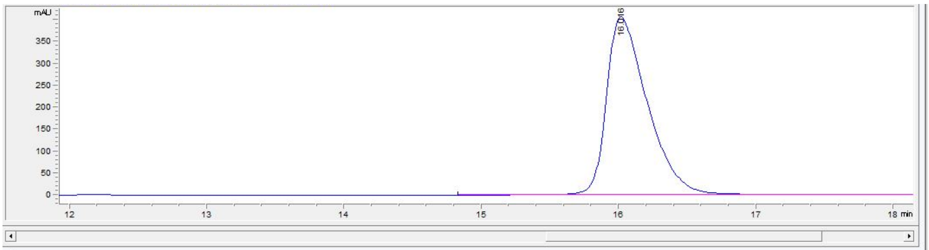

#	Time	Area	Height	Width	Area%	Symmetry
1	16.016	8524.7	406.8	0.3145	100.000	0.511

Methyl-4-(2-(3-oxo-3-phenylpropyl)pyrrolidin-1-yl)benzoate (1d)

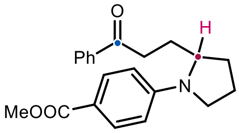

1d

Synthesized according to a modified reported literature procedure.[4] An oven-dried 4 mL vial equipped with a magnetic stir bar was charged with methyl 4-(pyrrolidin-1-yl)benzoate (0.10 mmol, 1.0 equiv), (Ir[dF(CF_3)ppy]$_2$(dtbpy))PF_6 (2.3 mg, 0.002 mmol), and quinuclidine (7 mg, 0.20 mmol, 2.0 equiv). The vial was sealed with a septum, evacuated, and backfilled with N_2 (two cycles). MeCN (1 mL) was then added under a nitrogen atmosphere, and the reaction mixture was degassed by bubbling N_2 for 5 min. Phenyl vinyl ketone **9** (22 µL, 0.20 mmol, 2.0 equiv) was added under nitrogen, and the mixture was irradiated at 427 nm overnight. Upon completion of the reaction, the solvent was removed under reduced pressure and the crude residue was purified by flash column chromatography on silica gel (10% EtOAc in hexane) to afford compound **1d** (20 mg, 60% yield).

^{1}H NMR (600 MHz, $CDCl_3$) δ 7.97 – 7.93 (m, 2H), 7.91 – 7.87 (m, 2H), 7.57 (ddt, *J* = 7.8, 7.0, 1.3 Hz, 1H), 7.49 – 7.44 (m, 2H), 6.64 – 6.60 (m, 2H), 3.96 – 3.90 (m, 1H), 3.85 (s, 3H), 3.49 (ddd, *J* = 9.8, 8.1, 2.4 Hz, 1H), 3.26 (td, *J* = 9.3, 6.9 Hz, 1H), 3.07 – 3.01 (m, 2H), 2.26 – 2.18 (m, 1H), 2.15 – 2.08 (m, 1H), 2.07 – 1.98 (m, 2H), 1.89 (ddt, *J* = 11.4, 4.7, 3.4 Hz, 1H), 1.74 (dddd, *J* = 14.1, 9.9, 7.4, 6.0 Hz, 1H).

^{13}C NMR (151 MHz, $CDCl_3$) δ 199.46, 167.58, 150.37, 136.78, 133.18, 131.44, 128.68, 128.03, 116.60, 111.15, 57.70, 51.44, 48.17, 35.29, 29.96, 26.93, 23.20.

HRMS (ESI$^+$) $C_{21}H_{23}NO_3Na$ [M+Na]$^+$: found 360.1578, required 360.1571.

D. Experimental Procedures

D.1 General Procedure for the Stereoretentive Yang Photocyclization

<u>General procedure **C:**</u>

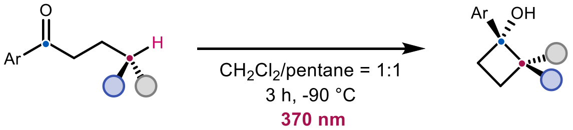

1 or **5** **2** or **6**

An oven-dried 8 mL vial equipped with a magnetic stir bar was charged with the enantioenriched ketone substrate **1** or **5** (0.05 mmol) and sealed with a septum. The solvent mixture (CH_2Cl_2/pentane = 1:1 (v/v), 1 mL) was then added under a nitrogen atmosphere and the resulting solution was degassed by bubbling N_2 with stirring. The vial was subsequently placed in an EtOH/liquid nitrogen cooling bath maintained at − 90 °C and irradiated with a 370 nm Kessil lamp (100% intensity). After 3 h, the light source was turned off and the reaction mixture was allowed to warm to room temperature. The solvent was removed under reduced pressure and the crude residue was purified by flash column chromatography (EtOAc in hexane) to afford the desired product **2** or **6**. Unless otherwise stated, reported yields and stereochemical data represent the average of two independent experiments for each substrate. In all cases, products were obtained as single detectable diastereomers.

For all product-ratio determinations reported in this study, reaction-channel selectivity was determined by ^{1}H NMR analysis of the crude reaction mixtures, using the diagnostic signals of cyclization product **2** and acetophenone **3**. Under the standard reaction conditions, compound **3′** was not detected and was therefore not included in the quantitative analysis, likely owing to its instability under the reaction conditions.

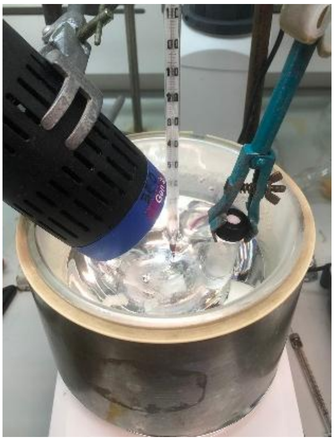

Figure S2. Reaction set-up using a Kessil lamp at 370 nm.

Scale-up Procedure for the Stereoretentive Yang Photocyclization

General procedure **D**:

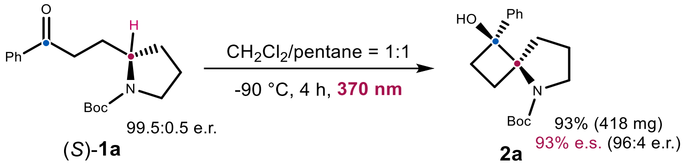

An oven-dried 100 mL round bottom flask equipped with a magnetic stir bar was charged with the enantioenriched ketone (*S*)-**1a** and sealed with a septum. A solvent mixture of CH_2Cl_2/pentane (1:1 v/v, 30 mL) was then added under a nitrogen atmosphere and the resulting solution was degassed by bubbling N_2 with stirring. The flask was subsequently placed in an EtOH/liquid nitrogen cooling bath maintained at − 90 °C and irradiated with a 370 nm Kessil lamp (100% intensity). After 4h, the light source was turned off and the reaction mixture was allowed to warm to room temperature. The solvent was removed under reduced pressure and the crude residue was purified by flash column chromatography (7% EtOAc in hexane) to afford **2a**.

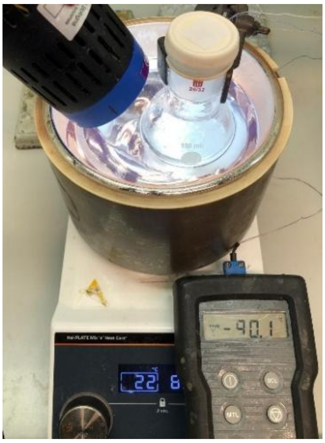

Figure S3. Reaction set-up for the 1.5 mmol-scale stereoretentive Yang photocyclization using a 370 nm Kessil lamp.

D.2 Characterization of the Norrish-Yang Photocyclization Products

***tert*-Butyl (1*S*,4*S*)-1-hydroxy-1-phenyl-5-azaspiro[3.4]octane-5-carboxylate (2a)**

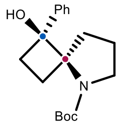

2a

Synthesized according to the general procedure **C** using enantioenriched ketone (*S*)-**1a** (15 mg, 0.05 mmol, >99.5:0.5 e.r). The crude mixture was purified by flash column chromatography on silica gel (7% EtOAc in hexane) to afford the title compound **2a** as a clear oil (13 mg, 86% yield). The enantiomeric ratio was determined by SFC analysis on a chiral Daicel Chiralpak IG-3 (95:5 CO_2:*i*-PrOH, 1.0 mL/min, 20 °C, λ = 210 nm:, τ_{major} = 5.138 min, τ_{minor} = 6.1 min), 98:2 e.r.; $[\alpha]^D_{20}$ = +47.3 (c = 0.15, $CHCl_3$). An enantiospecificity (e.s.) of 97% was determined for the reaction. A single detectable diastereomer was observed by ^{1}H NMR and HPLC analysis.

The reaction was performed also on a larger scale following general procedure **D** using **1a** (450 mg, 1.5 mmol, >99.5:0.5 e.r). After 4 h of irradiation, complete conversion was observed. After evaporation of the solvent the crude reaction mixture was purified by flash column chromatography on silica gel (7% EtOAc in hexane) to afford product **2a** (418 mg, 93% yield, 93% e.s., 96:4 e.r.).

^{1}H NMR (600 MHz, $CDCl_3$) δ 7.48 – 7.44 (m, 2H), 7.35 – 7.30 (m, 2H), 7.26 – 7.22 (m, 1H), 6.34 (bs, 1H), 3.52 (m, 1H), 3.40 – 3.23 (m, 2H), 2.67 (dt, *J* = 12.5, 9.2 Hz, 1H), 2.15 (ddd, *J* = 12.5, 10.8, 4.2 Hz, 1H), 1.88 – 1.77 (m, 2H), 1.53 (s, 10H), 1.33 (ddd, *J* = 12.2, 6.1, 3.3 Hz, 1H), 1.05 – 0.95 (m, 1H).

^{13}C NMR (151 MHz, $CDCl_3$) δ 140.95, 126.67, 126.08, 125.47, 85.47, 79.69, 70.64, 47.99, 36.93, 28.69, 28.29, 27.51, 26.57, 20.07, 13.10, -1.02.

HRMS (ESI$^+$) $C_{18}H_{25}NO_3$ $[M+H]^+$: found 304.1903, required 304.1908.

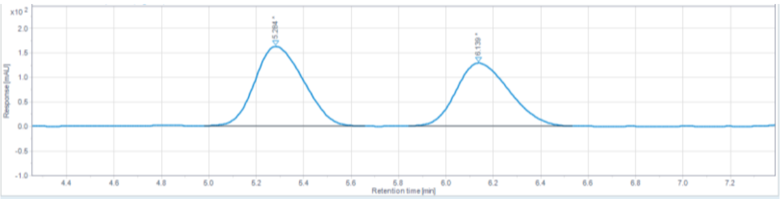

Injection Results

Peaks | Summary

#	Signal description	RT (min)	Area (mAU·s)	Area%	Height (mAU)	Height%	Amount	Concentration	Start time (min)	End time (min)
1	DAD1C,Sig=210,4 ...	5.284	2309.413	53.633	162.431	55.82			4.982	5.660
2	DAD1C,Sig=210,4 ...	6.139	1996.573	46.367	128.567	44.18			5.843	6.534

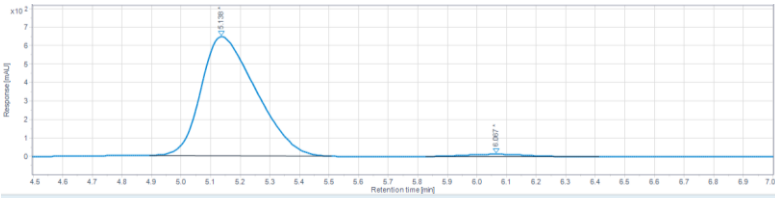

Injection Results

Peaks | Summary

#	Signal description	RT (min)	Area (mAU·s)	Area%	Height (mAU)	Height%	Amount	Concentration	Start time (min)	End time (min)
1	DAD1C,Sig=210,4 ...	5.138	8320.809	97.853	645.924	98.14			4.895	5.510
2	DAD1C,Sig=210,4 ...	6.067	182.583	2.147	12.245	1.86			5.827	6.414

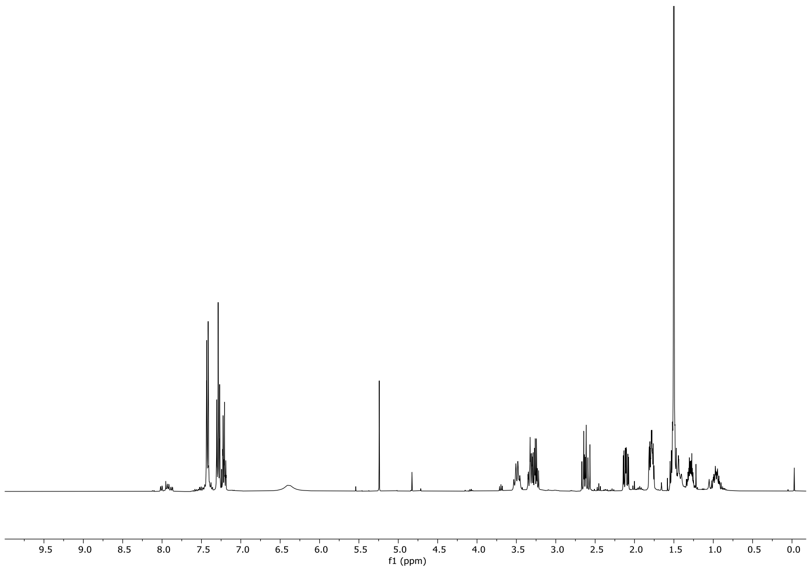

Figure S4. ^{1}HNMR spectrum of the crude reaction mixture from the 1.5 mmol scale-up.

((1*S*,4*S*)-1-Hydroxy-1-phenyl-5-azaspiro[3.4]octan-5-yl)(phenyl)methanone (2b)

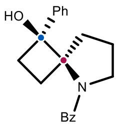

2b

Synthesized according to the general procedure **C** from enantioenriched ketone (*S*)-**1b** (15 mg, 0.05 mmol, 98.5:1.5 e.r.). The crude mixture was purified by flash column chromatography on silica gel (10% EtOAc in hexane) to afford the title compound **2b** as a clear oil (13 mg, 85% yield). The enantiomeric ratio was determined by HPLC analysis on a chiral Daicel Chiralpak AD-H column (85:15 hexane:*i*-PrOH, 1.0 mL/min, 30 °C, λ = 254 nm:, τ_{major} = 23.5 min, τ_{minor} = 37.3 min), 96.5:3.5 e.r.; $[\alpha]^{D}_{20}$ = +44.0 (c = 0.05, $CHCl_3$). An enantiospecificity (e.s.) of 96% was determined for the reaction. A single detectable diastereomer was observed by ^{1}H NMR and HPLC analysis.

^{1}H NMR (600 MHz, $CDCl_3$) δ 7.52 (ddd, *J* = 7.3, 3.6, 1.4 Hz, 4H), 7.45 – 7.41 (m, 3H), 7.36 (dd, *J* = 8.4, 7.0 Hz, 2H), 7.30 – 7.25 (m, 1H), 3.74 – 3.59 (m, 1H), 3.41 (ddd, *J* = 10.4, 8.5, 6.8 Hz, 1H), 3.27 (ddd, *J* = 10.9, 7.5, 4.2 Hz, 1H), 2.78 (ddd, *J* = 12.7, 9.4, 8.1 Hz, 1H), 2.47 (ddd, *J* = 12.7, 11.0, 4.8 Hz, 1H), 1.95 (ddd, *J* = 11.8, 9.4, 4.8 Hz, 1H), 1.81 (dt, *J* = 12.9, 5.4 Hz, 1H), 1.63 (s, 2H), 1.40 (dddd, *J* = 12.8, 11.0, 6.8, 3.6 Hz, 1H).

^{13}C NMR (151 MHz, $CDCl_3$) δ 173.92, 142.14, 137.75, 129.88, 128.47, 127.87, 127.25, 126.53, 126.47, 86.57, 74.39, 52.85, 38.06, 30.94, 30.59, 27.19, 22.24.

HRMS (ESI$^+$) $C_{20}H_{21}NO_2Na$ $[M+Na]^+$: found 330.1472, required 330.1465 .

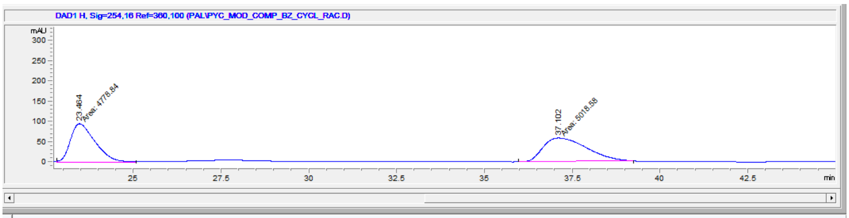

#	Time	Area	Height	Width	Area%	Symmetry
1	23.464	4778.8	95.8	0.8317	48.777	0.561
2	37.102	5018.6	57.9	1.4435	51.223	0.538

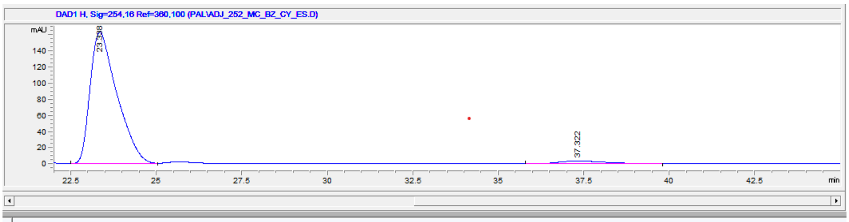

#	Time	Area	Height	Width	Area%	Symmetry
1	23.338	8955	163.2	0.8161	96.553	0.526
2	37.322	319.7	3.5	1.3681	3.447	0.64

2,2,2-trifluoro-1-((1S,4S)-1-hydroxy-1-phenyl-5-azaspiro[3.4]octan-5-yl)ethan-1-one (2c)

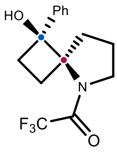

2c

Synthesized according to the general procedure **C** from enantioenriched ketone (*S*)-**1c** (15 mg, 0.05 mmol, 99:5:0.5 e.r.). The crude mixture was purified by flash column chromatography on silica gel (15% EtOAc in hexane) to afford the title compound **2c** as a clear oil (7 mg, 43% yield). The enantiomeric ratio was determined by HPLC analysis on a chiral Daicel Chiralpak IC-3 column (95:5 hexane:*i*-PrOH, 1.0 mL/min, 30 °C, λ = 234 nm:, τ_{major} = 7.3 min, τ_{minor} = 13.2 min), 86:14 e.r.;. An enantiospecificity (e.s.) of 73% was determined for the reaction. A single detectable diastereomer was observed by ^{1}H NMR and HPLC analysis.

^{1}H NMR (600 MHz, CDCl$_3$) δ 7.50 – 7.44 (m, 2H), 7.40 – 7.33 (m, 2H), 7.33 – 7.27 (m, 1H), 4.83 (s, 1H), 3.71 (t, *J* = 8.9 Hz, 1H), 3.66 – 3.56 (m, 1H), 3.50 (ddd, *J* = 12.6, 11.3, 7.7 Hz, 1H), 2.81 (ddd, *J* = 13.1, 9.6, 7.7 Hz, 1H), 2.46 (ddd, *J* = 13.2, 11.3, 5.5 Hz, 1H), 1.95 (ddd, *J* = 12.5, 9.6, 5.5 Hz, 1H), 1.83 (ddd, *J* = 10.9, 5.3, 2.1 Hz, 1H), 1.62 – 1.55 (m, 1H), 1.49 (tt, *J* = 6.3, 3.1 Hz, 1H), 1.25 – 1.12 (m, 1H).

^{13}C NMR (151 MHz, CDCl$_3$) δ 159.37 (q), 141.13, 128.18, 127.80, 126.38, 115.70 (q), 86.06, 76.41, 49.11, 49.09, 49.06, 49.03, 36.91, 31.29, 26.22, 22.09.

^{19}F NMR (376 MHz, CDCl$_3$) δ -72.06.

HRMS (ESI$^+$) $C_{15}H_{16}NO_2F_3Na$ [M+Na]$^+$: found 322.1023, required 322.1025.

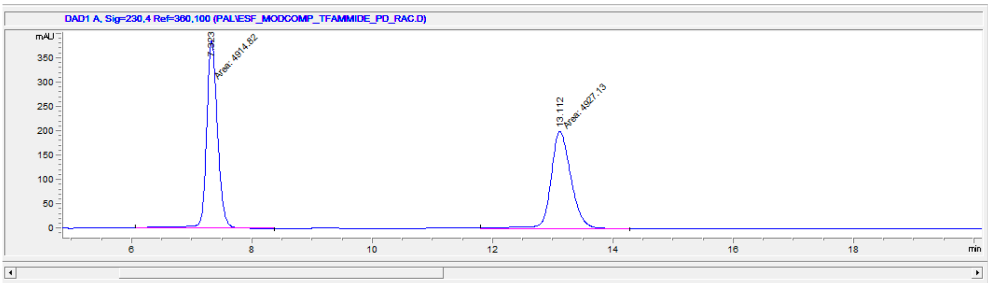

#	Time	Area	Height	Width	Area%	Symmetry
1	7.323	4914.8	385.2	0.2127	49.937	0.843
2	13.112	4927.1	201.5	0.4076	50.063	0.817

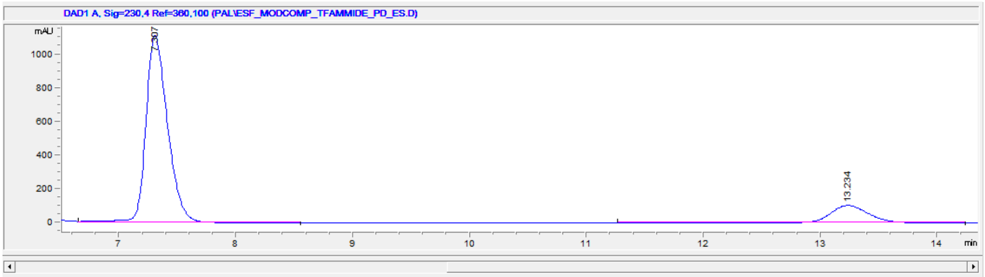

#	Time	Area	Height	Width	Area%	Symmetry
1	7.307	14476.4	1111.4	0.198	85.868	0.7
2	13.234	2382.5	103.4	0.3547	14.132	0.866

(-) *tert*-Butyl-1-hydroxy-1-phenyl-5-azaspiro[3.5]nonane-5-carboxylate (2e)

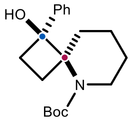

2e

Synthesized according to the general procedure **C** from enantioenriched ketone (+)-**1e** (16 mg, 0.05 mmol, >99.5:0.5 e.r.). The crude mixture was purified by flash column chromatography on silica gel (10% EtOAc in hexane) to afford the title compound **2e** as a clear oil (14 mg, 88% yield). The enantiomeric ratio was determined by HPLC analysis on a chiral Daicel Chiralpak AD-H (95:5 hexane:*i*-PrOH, 1.0 mL/min, 30 °C, λ = 230 nm:, τ_{major} = 8.5 min, τ_{minor} = 13.5 min), 90.5:9.5 e.r.; $[\alpha]^{D}_{20}$ = –17.3 (c = 0.21, $CHCl_3$). An enantiospecificity (e.s.) of 81% was determined for the reaction. A single detectable diastereomer was observed by ^{1}H NMR and HPLC analysis.

^{1}H NMR (400 MHz, $CDCl_3$) δ 7.65 – 7.59 (m, 2H), 7.37 – 7.29 (m, 2H), 7.26 – 7.22 (m, 1H), 5.49 (bs, 1H), 3.41 (m, 1H), 3.15 (m, 1H), 2.70 – 2.57 (m, 1H), 2.51 – 2.38 (m, 1H), 2.16 – 2.03 (m, 2H), 1.68 (m, 1H), 1.60 – 1.10 (m, 13H), 1.16 (m, 1H).

^{13}C NMR (151 MHz, $CDCl_3$) δ 156.40, 143.16, 127.82, 127.35, 126.96, 81.36, 80.44, 67.51, 43.60, 32.09, 31.93, 29.71, 29.09, 28.44, 28.41, 28.14, 24.59, 20.28, 1.02.

HRMS (ESI$^+$) $C_{19}H_{27}NO_3Na$ [M+Na]$^+$: found 340.1887, required 340.1884.

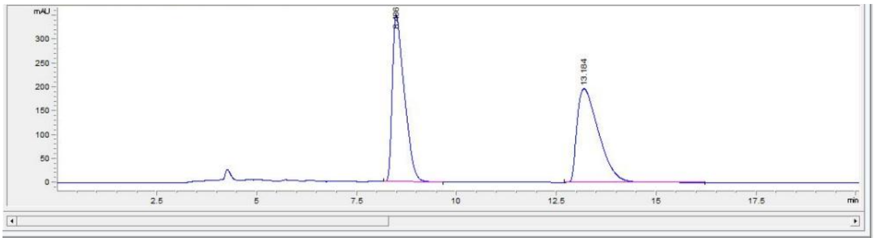

#	Time	Area	Height	Width	Area%	Symmetry
1	8.486	7338.5	348.5	0.3117	49.894	0.458
2	13.184	7369.8	196.3	0.5821	50.106	0.434

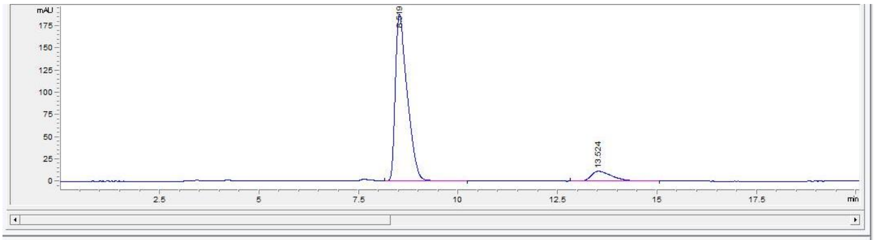

#	Time	Area	Height	Width	Area%	Symmetry
1	8.519	3901.9	187.8	0.3064	90.545	0.474
2	13.524	407.4	11.2	0.5217	9.455	0.513

***tert*-Butyl (1*S*,4*R*)-1-hydroxy-1-phenyl-8-oxa-5-azaspiro[3.5]nonane-5-carboxylate (2f)**

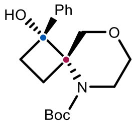

2f

Synthesized according to the general procedure **C** from enantioenriched ketone (*S*)-**1f** (16 mg, 0.05 mmol, >99.5:0.5 e.r.). The crude mixture was purified by flash column chromatography on silica gel (20% EtOAc in hexane) to afford the title compound **2f** as a clear oil (7.2 mg, 45% yield). The enantiomeric ratio was determined by HPLC analysis on a chiral Daicel Chiralpak AD-H (95:5 hexane:*i*-PrOH, 1.0 mL/min, 30 °C, λ = 230 nm:, τ_{major} = 10.0 min, τ_{minor} = 13.9 min), 98.0:2.0 e.r.; $[\alpha]^{D}_{20}$ = –30.3 (c = 0.18, $CHCl_3$). An enantiospecificity (e.s.) of 96% was determined for the reaction. A single detectable diastereomer was observed by ^{1}H NMR and HPLC analysis.

^{1}H NMR (600 MHz, $CDCl_3$) δ 7.60 – 7.48 (m, 2H), 7.38 – 7.30 (m, 2H), 7.29 – 7.21 (m, 1H), 5.67 (s, 1H), 3.66 – 3.55 (m, 2H), 3.54 (dd, *J* = 12.0, 1.6 Hz, 1H), 3.48 – 3.39 (m, 2H), 3.39 – 3.31 (m, 1H), 2.79 (dtd, *J* = 11.5, 9.8, 1.5 Hz, 1H), 2.58 – 2.42 (m, 1H), 2.28 – 2.16 (m, 2H), 1.51 (s, 9H).

^{13}C NMR (151 MHz, $CDCl_3$) δ 156.32, 142.42, 128.09, 127.44, 127.04, 81.53, 81.44, 71.63, 66.49, 66.40, 43.88, 29.03, 28.56, 26.24.

HRMS (ESI$^+$) $C_{18}H_{25}NO_4Na$ [M+Na]$^+$: found 342.1680, required 342.1676.

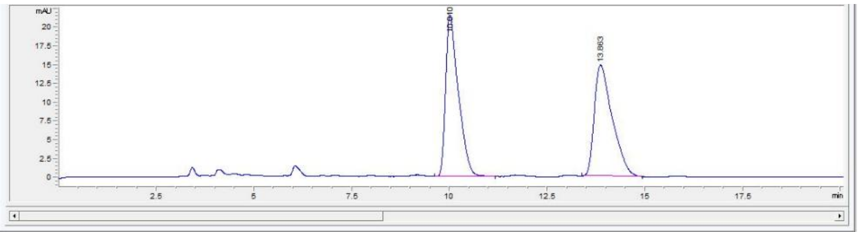

#	Time	Area	Height	Width	Area%	Symmetry
1	10.01	489.1	21.5	0.3291	50.688	0.492
2	13.863	475.8	14.8	0.471	49.312	0.511

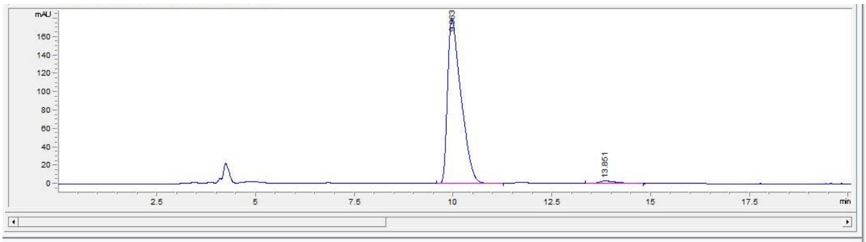

#	Time	Area	Height	Width	Area%	Symmetry
1	9.963	4284.5	179.9	0.3498	97.935	0.451
2	13.851	90.3	2.8	0.4416	2.065	0.535

***tert*-Butyl (4*R*,5*S*)-5-hydroxy-5-phenyl-1-azaspiro[3.3]heptane-1-carboxylate (2g)**

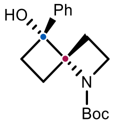

2g

Synthesized according to the general procedure **C** from enantioenriched ketone (*S*)-**1g** (15 mg, 0.05 mmol, 98.5:1.5 e.r.). The crude mixture was purified by flash column chromatography on silica gel (15% EtOAc in hexane) to afford the title compound **2g** as a clear oil (9 mg, 62% yield). The enantiomeric ratio was determined by HPLC analysis on a chiral Daicel Chiralpak AD-H (95:5 hexane:*i*-PrOH, 1.0 mL/min, 30 °C, λ = 230 nm:, τ_{major} = 5.5 min, τ_{minor} = 6.4 min), 69.5:30.5 e.r.; $[\alpha]^{D}_{20}$ = -6.0 (c = 0.10, $CHCl_3$). An enantiospecificity (e.s.) of 40% was determined for the reaction. A single detectable diastereomer was observed by ^{1}H NMR and HPLC analysis.

^{1}H NMR (400 MHz, $CDCl_3$) δ 7.45 (d, *J* = 7.4 Hz, 2H), 7.37 (t, *J* = 7.6 Hz, 2H), 7.28 (d, *J* = 7.1 Hz, 1H), 5.76 (bs, 1H), 3.74 (m, 1H), 3.52 (m, 1H), 3.00 (m, 1H), 2.51 (m, 1H), 2.39 (m, 1H), 2.16 – 2.00 (m, 1H), 1.73 (q, 2H), 1.50 (s, 9H).

^{13}C NMR (151 MHz, $CDCl_3$) δ 157.13, 142.38, 128.20, 127.04, 125.49, 82.93, 80.69, 44.79, 31.18, 29.71, 28.86, 28.46, 26.67, 1.03.

HRMS (ESI$^+$) $C_{17}H_{23}NNaO_3$ [M+Na]$^+$: found 312.1575, required 312.1571.

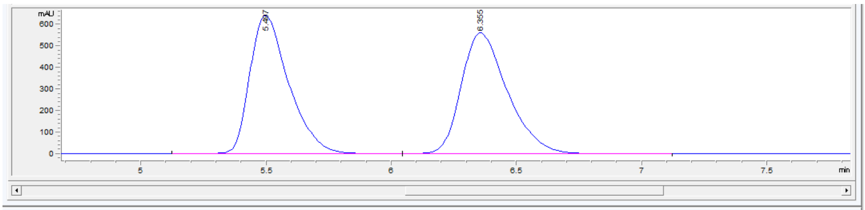

#	Time	Area	Height	Width	Area%	Symmetry
1	5.497	6937.6	641.3	0.1642	49.093	0.642
2	6.355	7194	561.1	0.1956	50.907	0.679

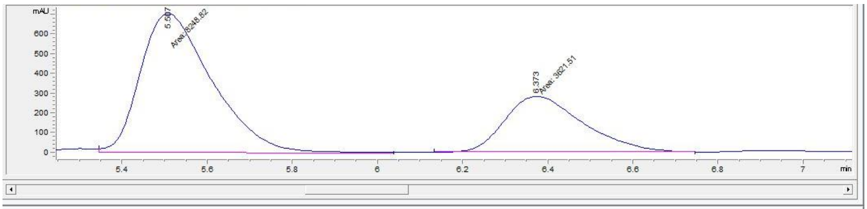

#	Time	Area	Height	Width	Area%	Symmetry
1	5.507	8248.8	704.6	0.1951	69.491	0.606
2	6.373	3621.5	283.8	0.2127	30.509	0.674

tert-Butyl (1*S*,4*R*)-1-hydroxy-6,6-dimethyl-1-phenyl-7-oxa-5-azaspiro[3.4]octane-5-carboxylate (2h)

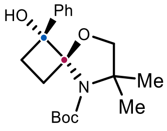

2h

Synthesized according to the general procedure **C** from enantioenriched ketone (*R*)-**1h** (17 mg, 0.05 mmol, 99.5:0.5 e.r.). The crude mixture was purified by flash column chromatography on silica gel (7% EtOAc in hexane) to afford the title compound **2h** as a clear oil (15 mg, 90% yield). The enantiomeric ratio was determined by SFC analysis on a chiral Daicel Chiralpak IG-3 (95:5 CO_2:*i*-PrOH, 1.0 mL/min, 20 °C, λ = 210 nm:, τ_{major} = 2.3 min, τ_{minor} = 3.1 min), 96:4 e.r.; $[\alpha]^{D}_{20}$ = +82.5 (c = 0.07, $CHCl_3$). An enantiospecificity (e.s.) of 93% was determined for the reaction. A single detectable diastereomer was observed by ^{1}H NMR and HPLC analysis.

^{1}H NMR (600 MHz, $CDCl_3$) δ 7.48 – 7.44 (m, 2H), 7.35 (t, 2H), 7.29 – 7.26 (m, 1H), 5.82 (bs, 1H), 3.65 (d, *J* = 9.4 Hz, 1H), 3.51 (d, *J* = 9.4 Hz, 1H), 3.38 – 3.22 (m, 1H), 2.66 (ddd, *J* = 13.0, 9.7, 7.9 Hz, 1H), 2.31 (ddd, *J* = 13.0, 11.4, 5.5 Hz, 1H), 1.84 (ddd, *J* = 12.4, 9.7, 5.5 Hz, 1H), 1.59 (s, 3H), 1.57 (s, 9H), 1.45 (s, 3H).

^{13}C NMR (151 MHz, $CDCl_3$) δ 141.48, 127.97, 127.47, 126.11, 95.39, 85.67, 81.83, 72.04, 70.80, 30.31, 28.47, 26.67, 24.53, 24.15.

HRMS (ESI^+) $C_{19}H_{27}NO_4Na$ $[M+Na]^+$: found 356.1836, required 356.1833.

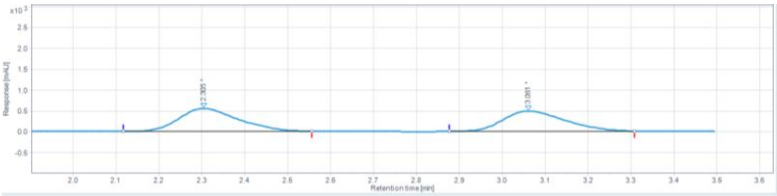

Injection Results

Peaks | Summary

#	Signal description	RT (min)	Area (mAU·s)	Area%	Height (mAU)	Height%	Amount	Concentration	Start time (min)	End time (min)
1	DAD1C,Sig=210,4 ...	2.305	5318.245	51.994	547.930	53.00			2.117	2.556
2	DAD1C,Sig=210,4 ...	3.061	4910.252	48.006	485.852	47.00			2.876	3.308

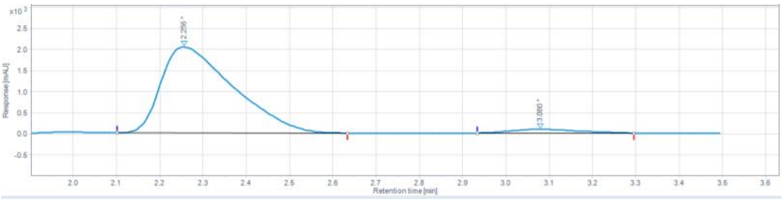

Injection Results

Peaks | Summary

#	Signal description	RT (min)	Area (mAU·s)	Area%	Height (mAU)	Height%	Amount	Concentration	Start time (min)	End time (min)
1	DAD1C,Sig=210,4 ...	2.256	23652.915	96.113	2043.535	95.86			2.102	2.634
2	DAD1C,Sig=210,4 ...	3.080	956.481	3.887	88.227	4.14			2.934	3.296

***tert*-Butyl (1*S*,2*R*)-2-hydroxy-2-phenylspiro[cyclobutane-1,2'-indoline]-1'-carboxylate (2i)**

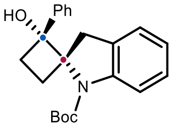

2i

Synthesized according to the general procedure **B** from enantioenriched ketone (S)-**1i** (17.6 mg, 0.05 mmol, 97:3 e.r.). The crude mixture was purified by flash column chromatography on silica gel (10% EtOAc in hexane) to afford the title compound **2i** as a clear oil (9.2 mg, 52% yield). The enantiomeric ratio was determined by HPLC analysis on a chiral Daicel Chiralpak AD-H (95:10 hexane:*i*-PrOH, 1.0 mL/min, 30 °C, λ = 230 nm:, τ_{major} = 5.8 min, τ_{minor} = 6.3 min), 95.5:4.5 e.r.; $[\alpha]^{D}_{20}$ = -6.0 (c = 0.35, $CHCl_3$). An enantiospecificity (e.s.) of 97% was determined for the reaction. A single detectable diastereomer was observed by ^{1}H NMR and HPLC analysis.

^{1}H NMR (600 MHz, $CDCl_3$) δ 7.49 (d, J = 8.2 Hz, 1H), 7.21 – 7.05 (m, 6H), 6.84 (td, J = 7.4, 1.1 Hz, 1H), 6.73 (d, J = 7.4 Hz, 1H), 5.84 (s, 1H), 3.85 (ddd, J = 11.9, 10.8, 9.3 Hz, 1H), 3.02 (d, J = 16.4 Hz, 1H), 2.93 (d, J = 16.4 Hz, 1H), 2.75 (dt, J = 12.6, 9.4 Hz, 1H), 2.32 (ddd, J = 12.6, 10.9, 3.9 Hz, 1H), 2.01 (ddd, J = 11.9, 9.4, 3.9 Hz, 1H), 1.67 (s, 9H).

^{13}C NMR (151 MHz, $CDCl_3$) δ 155.87, 142.99, 142.42, 129.28, 128.80, 128.18, 127.95, 127.56, 127.25, 126.33, 124.13, 123.06, 115.78, 86.91, 83.26, 77.16, 74.05, 42.09, 29.23, 29.15, 28.67.

HRMS (ESI$^+$) $C_{22}H_{25}NO_3Na$ [M+Na]$^+$: found 374.1734, required 374.1732.

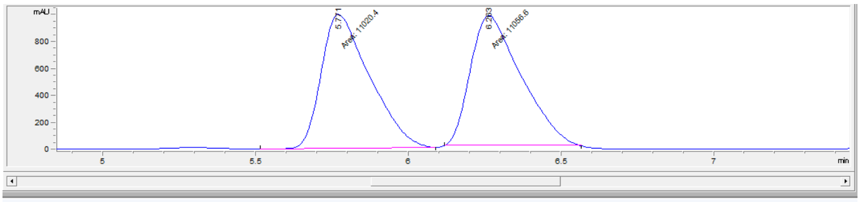

#	Time	Area	Height	Width	Area%	Symmetry
1	5.771	11020.4	997.3	0.1842	49.918	0.549
2	6.263	11056.6	961.9	0.1916	50.082	0.558

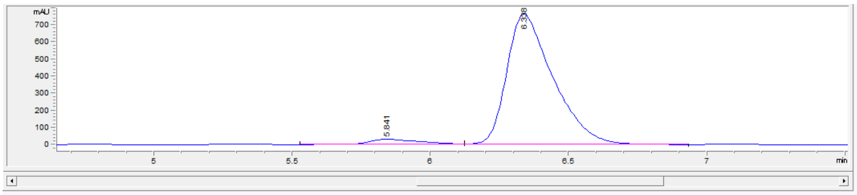

#	Time	Area	Height	Width	Area%	Symmetry
1	5.841	411.6	31.1	0.1826	4.422	0.529
2	6.338	8897.2	762.6	0.1701	95.578	0.558

tert-Butyl (1S,2R)-2-hydroxy-2-phenyl-1',4'-dihydro-2'H-spiro[cyclobutane-1,3'-isoquinoline]-2'-carboxylate (2j)

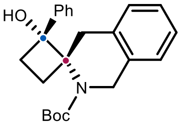

2j

Synthesized according to the general procedure **B** from enantioenriched ketone (S)-**1j** (18.3 mg, 0.05 mmol, > 99.5: 0.5 er). The crude mixture was purified by flash column chromatography on silica gel (10% EtOAc in Hexane) to afford the title compound **2j** as a clear oil (12.8 mg, 70% yield). The enantiomeric ratio was determined by HPLC analysis on a chiral Daicel Chiralpak AD-H (90:10 hexane:*i*-PrOH, 1.0 mL/min, 30 °C, λ = 230 nm:, τ_{major} = 13.9 min, τ_{minor} = 15.6 min), 96:4 e.r.; $[\alpha]^{D}_{20}$ = -2.6 (c = 0.28, $CHCl_3$). An enantiospecificity (e.s.) of 92% was determined for the reaction. A single detectable diastereomer was observed by ^{1}H NMR and HPLC analysis.

^{1}H NMR (600 MHz, $CDCl_3$) δ 7.51 – 7.39 (m, 2H), 7.24 – 7.11 (m, 4H), 7.12 – 6.91 (m, 4H), 6.67 (d, *J* = 7.5 Hz, 1H), 5.88 – 5.56 (m, 1H), 4.71 – 4.60 (m, 1H), 4.48 (d, *J* = 16.3 Hz, 1H), 3.07 (dddd, *J* = 11.8, 10.7, 9.5, 1.2 Hz, 1H), 2.88 (d, *J* = 15.9 Hz, 1H), 2.77 – 2.64 (m, 2H), 2.25 – 2.17 (m, 1H), 1.86 (ddd, *J* = 12.0, 9.3, 3.3 Hz, 1H), 1.50 (s, 9H).

^{13}C NMR (151 MHz, $CDCl_3$) δ 155.87, 142.99, 142.42, 129.28, 128.80, 128.18, 127.95, 127.56, 127.25, 126.33, 124.13, 123.06, 115.78, 86.91, 83.26, 77.16, 74.05, 42.09, 29.23, 29.15, 28.67.

HRMS (ESI$^+$) $C_{23}H_{27}NO_3Na$ [M+Na]$^+$: found 388.1888, required 388.1884.

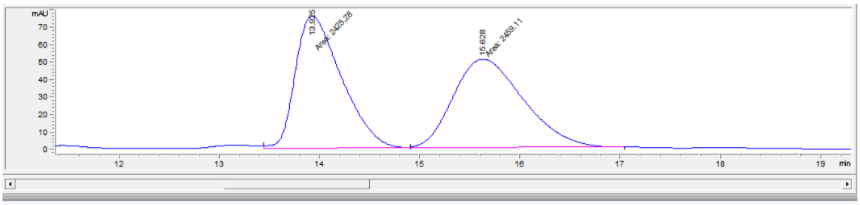

#	Time	Area	Height	Width	Area%	Symmetry
1	13.925	2425.3	75.8	0.5331	49.654	0
2	15.628	2459.1	50.7	0.8089	50.346	0.718

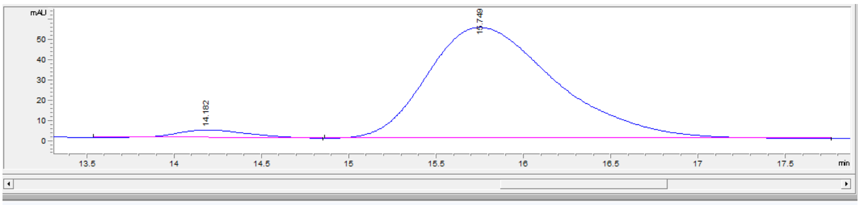

#	Time	Area	Height	Width	Area%	Symmetry
1	14.182	115.4	4	0.4415	3.971	0.742
2	15.749	2790	54.8	0.7905	96.029	0.655

tert-Butyl (2-((1S,4S)-1-hydroxy-1-phenyl-5-azaspiro[3.4]octan-5-yl)-2-oxoethyl)carbamate (2k)

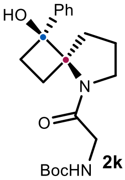

2k

Synthesized according to the general procedure **B** from enantioenriched ketone (S)-**1k** (18.0 mg, 0.05 mmol, 97.5:2.5 er). The crude mixture was purified by flash column chromatography on silica gel (50% EtOAc in hexane) to afford the title compound **2k** as a clear oil (14.9 mg, 83% yield). The enantiomeric ratio was determined by SFC analysis on a chiral Daicel Chiralpak IB-N3 (90:10 CO_2:*i*-PrOH, 1.0 mL/min, 20 °C, λ = 230 nm:, τ_{minor} = 6.0 min, τ_{major} = 9.2 min), 96:4 e.r.; $[\alpha]^D_{20}$ = +12.4 (c = 0.34, $CHCl_3$). An enantiospecificity (e.s.) of 97% was determined for the reaction. A single detectable diastereomer was observed by ^{1}H NMR and HPLC analysis.

^{1}H NMR (600 MHz, $CDCl_3$) δ 7.40 – 7.35 (m, 2H), 7.27 (dd, *J* = 8.4, 6.9 Hz, 2H), 7.23 – 7.20 (m, 1H), 5.92 (s, 1H), 5.44 (s, 2H), 4.03 – 3.84 (m, 3H), 3.54 (td, *J* = 11.3, 8.6 Hz, 1H), 3.41 – 3.17 (m, 3H), 2.67 (dt, *J* = 12.7, 9.1 Hz, 1H), 2.22 (ddd, *J* = 12.6, 10.9, 4.4 Hz, 2H), 1.79 (tdd, *J* = 13.9, 7.9, 3.5 Hz, 3H), 1.41 (s, 9H).

^{13}C NMR (151 MHz, $CDCl_3$) δ 171.44, 155.96, 141.46, 128.04, 127.59, 126.52, 86.62, 79.97, 74.64, 48.24, 44.14, 37.39, 30.06, 29.85, 28.51, 27.13, 21.86.

HRMS (ESI$^+$) $C_{20}H_{28}N_2O_4Na$ [M+Na]$^+$: found 383.1945, required 383.1942.

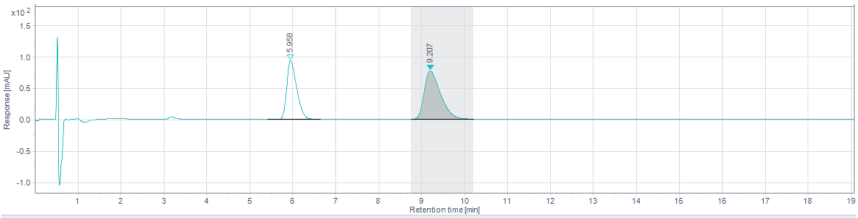

Injection Results

Peaks | Summary

#	Signal description	RT (min)	Area (mAU·s)	Area%	Height (mAU)	Height%	Amount	Concentration	Start time (min)	End time (min)
1	DAD1D,Sig=230,4 ...	5.958	1501.558	42.288	93.510	54.68			5.428	6.651
2	DAD1D,Sig=230,4 ...	9.207	2049.214	57.712	77.517	45.32			8.767	10.218

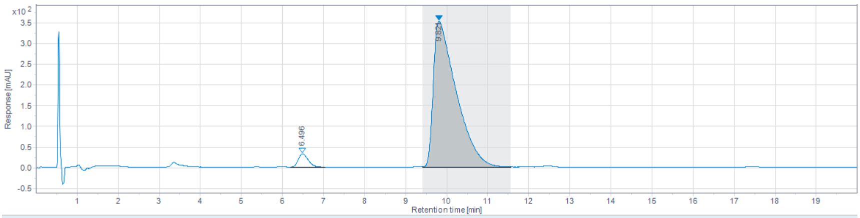

Injection Results

Peaks | Summary

#	Signal description	RT (min)	Area (mAU·s)	Area%	Height (mAU)	Height%	Amount	Concentration	Start time (min)	End time (min)
1	DAD1D,Sig=230,4 ...	6.496	543.296	3.833	32.244	8.40			6.206	7.053
2	DAD1D,Sig=230,4 ...	9.821	13632.429	96.167	351.596	91.60			9.418	11.576

tert-Butyl (2-((1S,4S)-1-hydroxy-1-phenyl-5-azaspiro[3.4]octan-5-yl)-2-oxoethyl)carbamate (2l)

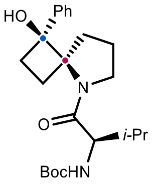

2l

Synthesized according to the general procedure **C** from enantioenriched ketone (*S*,*S*)-**1l** (20 mg, 0.05 mmol, >20:1 d.r.). The crude mixture was purified by flash column chromatography on silica gel (50% EtOAc in hexane) to afford the title compound **2l** as a clear oil (15.6 mg, 78% yield). A single diastereomer of **2l** was detected by NMR and HPLC analysis, which indicated an enantiospecificity (e.s.) of 99%. $[\alpha]^{D}_{20}$ = +62.4 (c = 0.07, $CHCl_3$).

^{1}H NMR (600 MHz, $CDCl_3$) δ 7.50 – 7.44 (m, 2H), 7.33 (dd, *J* = 8.4, 7.0 Hz, 2H), 7.26 (s, 1H), 6.42 (s, 1H), 5.26 (d, *J* = 9.4 Hz, 1H), 4.37 (dd, *J* = 9.4, 6.6 Hz, 1H), 3.80 (t, *J* = 8.9 Hz, 1H), 3.47 (dtd, *J* = 26.8, 10.7, 7.5 Hz, 2H), 2.72 (ddd, *J* = 12.7, 9.4, 8.3 Hz, 1H), 2.30 (ddd, *J* = 12.7, 10.9, 4.6 Hz, 1H), 2.02 – 1.96 (m, 1H), 1.88 (ddd, *J* = 12.8, 6.3, 2.2 Hz, 1H), 1.82 (ddd, *J* = 11.8, 9.4, 4.6 Hz, 1H), 1.53 (dt, *J* = 12.6, 6.3 Hz, 2H), 1.49 (s, 9H), 1.45 – 1.38 (m, 1H), 1.11 (dtdd, *J* = 18.6, 12.4, 6.0, 3.9 Hz, 1H), 0.98 (dd, *J* = 13.4, 6.7 Hz, 6H).

^{13}C NMR (151 MHz, $CDCl_3$) δ 175.50, 156.03, 141.66, 127.80, 127.31, 126.55, 86.35, 79.77, 74.41, 57.87, 49.69, 37.52, 31.53, 30.13, 28.38, 26.48, 21.52, 19.44, 17.66.

HRMS (ESI^{+}) $C_{23}H_{34}N_2O_4Na$ [M+Na^{+}]: found 425.2408, required 425.2411.

(-)-*tert*-Butyl-1-hydroxy-1-(4-(trifluoromethyl)phenyl)-5-azaspiro[3.4]octane-5-carboxylate (2m)

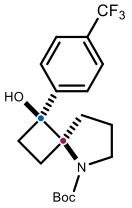

2m

Synthesized according to the general procedure **C** from enantioenriched (+)-**1m** (19 mg, 0.05 mmol, >99.5:0.5 e.r.). The crude mixture was purified by flash column chromatography on silica gel (3% EtOAc in hexane) to afford the title compound **2m** as a clear oil (17.3 mg, 91% yield). The enantiomeric ratio was determined by HPLC analysis on a chiral Daicel Chiralpak IC-3 (97:3 hexane:*i*-PrOH, 1.0 mL/min, 30 °C, λ = 254 nm:, τ_{major} = 4.6 min, τ_{minor} = 5.1 min), 96:4 e.r.; $[\alpha]^{D}_{20}$ = -73.3 (c = 0.32, $CHCl_3$). An enantiospecificity (e.s.) of 92% was determined for the reaction. A single detectable diastereomer was observed by ^{1}H NMR and HPLC analysis.

^{1}H NMR (600 MHz, $CDCl_3$) δ 7.59 (s, 4H), 6.53 (s, 1H), 3.53 (q, *J* = 10.7 Hz, 1H), 3.39 – 3.26 (m, 2H), 2.66 (dt, *J* = 12.7, 9.2 Hz, 1H), 2.25 – 2.11 (m, 1H), 1.85 (ddd, *J* = 11.8, 9.4, 4.2 Hz, 1H), 1.77 (ddd, *J* = 13.0, 6.4, 2.9 Hz, 1H), 1.61 – 1.57 (m, 1H), 1.54 (d, *J* = 8.5 Hz, 9H), 1.36 (dtt, *J* = 12.4, 6.4, 2.8 Hz, 1H), 0.98 – 0.91 (m, 1H).

^{13}C NMR (151 MHz, $CDCl_3$) ^{13}C NMR (151 MHz, $CDCl_3$) δ 146.01, 129.40, 129.18, 126.90, 125.18, 124.73, 124.70, 124.68, 124.65, 86.10, 81.01, 71.68, 49.00, 37.87, 29.48, 28.50, 27.71, 21.15.

^{19}F NMR (376 MHz, $CDCl_3$) δ -62.40.

HRMS (ESI$^+$) $C_{19}H_{24}F_3NO_3Na$ [M+Na]$^+$: found 394.1599, required 394.1601.

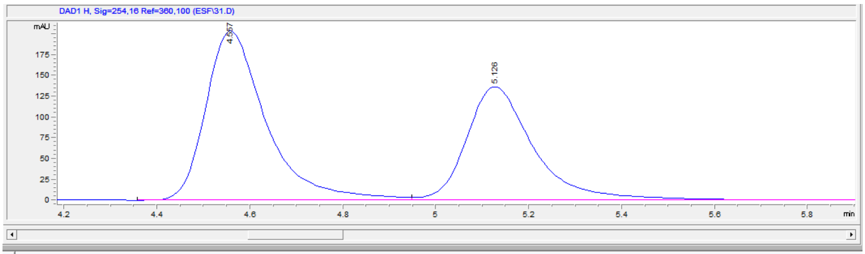

#	Time	Area	Height	Width	Area%	Symmetry
1	4.557	1838	203.8	0.1352	57.087	0.629
2	5.126	1381.6	136.6	0.152	42.913	0.662

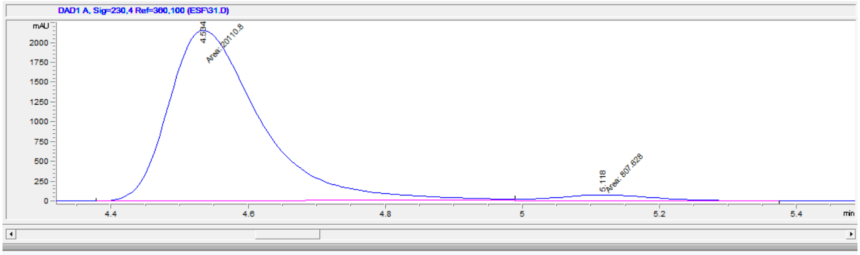

#	Time	Area	Height	Width	Area%	Symmetry
1	4.534	20110.8	2155.4	0.1555	96.139	0.587
2	5.118	807.6	79.7	0.1689	3.861	0.862

(-)-*tert*-Butyl-1-hydroxy-1-(4-methoxyphenyl)-5-azaspiro[3.4]octane-5-carboxylate (2n)

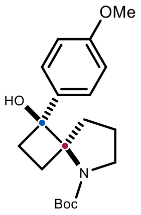

2n

Synthesized according to a modified general procedure **C** starting from enantioenriched (+)-**1n** (19 mg, 0.05 mmol, >99.5:0.5 e.r.). The reaction was conducted in CH_2Cl_2 and irradiated at −50 °C using a chiller system, as no conversion was observed in 3 h at −90 °C. After 16 h of irradiation, the solvent was removed under reduced pressure and the crude residue was purified by flash column chromatography on silica gel (15% EtOAc in hexane) to afford compound **2n** as a clear oil (4.8 mg, 25% yield). The enantiomeric ratio was determined by HPLC analysis on a chiral Daicel Chiralpak IC-3 (90:10 hexane:*i*-PrOH, 1.0 mL/min, 30 °C, λ = 254 nm:, τ_{minor} = 15.9 min, τ_{major} = 17.8 min), 81:19 e.r.; $[\alpha]^{D}_{20}$ = -35.0 (c = 0.12, $CHCl_3$). An enantiospecificity (e.s.) of 62% was determined for the reaction. A single detectable diastereomer was observed by ^{1}H NMR and HPLC analysis.

^{1}H NMR (600 MHz, $CDCl_3$) δ 7.59 (s, 4H), 6.53 (s, 1H), 3.53 (q, *J* = 10.7 Hz, 1H), 3.39 – 3.26 (m, 2H), 2.66 (dt, *J* = 12.7, 9.2 Hz, 1H), 2.25 – 2.11 (m, 1H), 1.85 (ddd, *J* = 11.8, 9.4, 4.2 Hz, 1H), 1.77 (ddd, *J* = 13.0, 6.4, 2.9 Hz, 1H), 1.61 – 1.57 (m, 1H), 1.54 (d, *J* = 8.5 Hz, 9H), 1.36 (dtt, *J* = 12.4, 6.4, 2.8 Hz, 1H), 0.98 – 0.91 (m, 1H).

^{13}C NMR (151 MHz, $CDCl_3$) ^{13}C NMR (151 MHz, $CDCl_3$) δ 146.01, 129.40, 129.18, 126.90, 125.18, 124.73, 124.70, 124.68, 124.65, 86.10, 81.01, 71.68, 49.00, 37.87, 29.48, 28.50, 27.71, 21.15.

^{19}F NMR (376 MHz, $CDCl_3$) δ -62.40.

HRMS (ESI$^+$) $C_{19}H_{27}NO_4Na$ [M+Na]$^+$: found 356. 1830, required 356.1833.

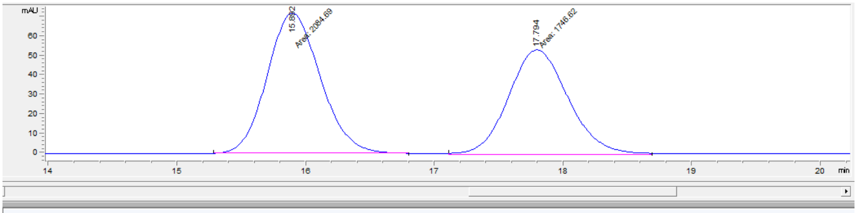

#	Time	Area	Height	Width	Area%	Symmetry
1	15.892	2084.7	72.2	0.4811	54.412	0.864
2	17.794	1746.6	53.8	0.5412	45.588	0.88

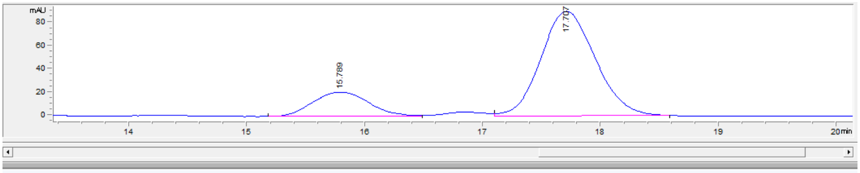

#	Time	Area	Height	Width	Area%	Symmetry
1	15.789	698.6	20.9	0.4015	19.284	0.903
2	17.707	2924.1	90.4	0.5036	80.716	0.835

tert-butyl (1S,4S)-1-hydroxy-1-(pyridin-3-yl)-5-azaspiro[3.4]octane-5-carboxylate (2o)

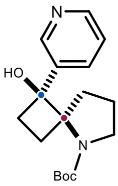

2o

Synthesized according to general procedure **C** starting from enantioenriched (+)-**1o** (16 mg, 0.05 mmol). The crude mixture was purified by flash column chromatography on silica gel (20% EtOAc in hexane) to afford the title compound **2o** as a clear oil (13.7 mg, 91% yield). The enantiomeric ratio was determined by SFC analysis on a chiral Daicel Chiralpak IB-N3 (97:3 hexane:CO_2, 1.0 mL/min, 20 °C, λ = 230 nm:, τ_{minor} = 7.6 min, τ_{major} = 8.1 min), 99:1 e.r.; An enantiospecificity (e.s.) of 98% was determined for the reaction. A single detectable diastereomer was observed by ^{1}H NMR and HPLC analysis.

^{1}H NMR (600 MHz, $CDCl_3$) δ 8.77 – 8.69 (m, 1H), 8.51 (dd, *J* = 4.9, 1.6 Hz, 1H), 7.79 (ddd, *J* = 8.0, 2.3, 1.7 Hz, 1H), 7.26 (s, 1H), 6.58 (s, 1H), 3.56 (q, *J* = 10.6 Hz, 1H), 3.36 (ddd, *J* = 10.7, 8.2, 2.5 Hz, 1H), 3.30 (td, *J* = 10.4, 6.7 Hz, 1H), 2.71 – 2.62 (m, 1H), 2.21 – 2.13 (m, 1H), 1.86 (ddd, *J* = 11.8, 9.4, 4.0 Hz, 1H), 1.79 (ddd, *J* = 13.0, 6.4, 2.8 Hz, 1H), 1.65 – 1.62 (m, 1H), 1.53 (s, 7H), 1.37 (dddd, *J* = 12.8, 6.6, 3.9, 2.7 Hz, 1H), 0.97 – 0.89 (m, 1H).

^{13}C NMR (151 MHz, $CDCl_3$) δ 148.66, 148.43, 137.38, 134.33, 122.84, 85.36, 81.21, 71.66, 49.11, 38.02, 29.85, 29.17, 28.63, 27.90, 21.17.

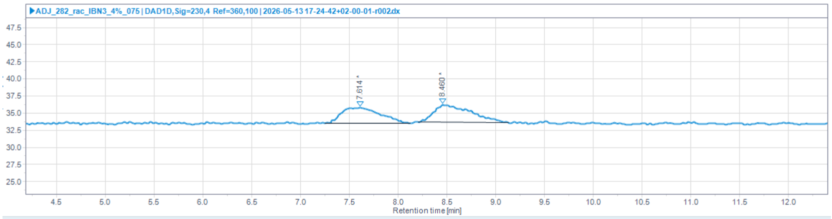

UV Spectrum

Injection Results

Peaks | Summary

#	Signal descript...	RT (min)	Area (mAU·s)	Area%	Height (mAU)	Height%	Amount	Concentration	Start time (min)	End time (min)
1	DAD1D,Sig=230,4 ...	7.614	140.864	48.444	4.982	47.24			7.250	8.123
2	DAD1D,Sig=230,4 ...	8.460	149.912	51.556	5.564	52.76			8.208	9.137

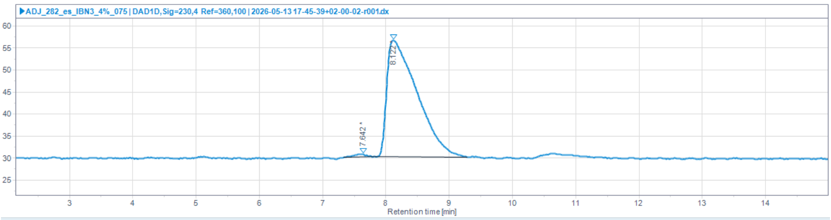

UV Spectrum

Injection Results

Peaks | Summary

#	Signal descript...	RT (min)	Area (mAU·s)	Area%	Height (mAU)	Height%	Amount	Concentration	Start time (min)	End time (min)
1	DAD1D,Sig=230,4 ...	7.642	20.606	0.997	1.419	2.25			7.335	7.838
2	DAD1D,Sig=230,4 ...	8.122	2045.564	99.003	61.614	97.75			7.838	9.290

tert-Butyl ((1S,2S)-2-hydroxy-1-methyl-2-phenylcyclobutyl)carbamate (6b)

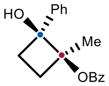

6b

Synthesized according to the general procedure **C** from enantioenriched ketone (*S*)-**5b** (14 mg, 0.05 mmol, 99:1 e.r.). The crude mixture was purified by flash column chromatography on silica gel (15% EtOAc in hexane) to afford the title compound **6b** as a clear oil (5.6 mg, 40 % yield). The enantiomeric ratio was determined by HPLC analysis on a chiral AD-H (95:5 hexane:*i*-PrOH, 1.0 mL/min, 30 °C, λ = 230 nm:, τ_{major} = 16.4 min, τ_{minor} = 33.2 min), 95:5 e.r.; $[\alpha]^{D}_{20}$ = -11.4 (c = 0.16, $CHCl_3$). An enantiospecificity (e.s.) of 92% was determined for the reaction. A single detectable diastereomer was observed by ^{1}H NMR and HPLC analysis.

^{1}H NMR (600 MHz, $CDCl_3$) δ 8.06 (dt, *J* = 7.2, 1.4 Hz, 2H), 7.63 – 7.54 (m, 1H), 7.47 (t, *J* = 7.7 Hz, 2H), 7.44 – 7.36 (m, 4H), 7.35 – 7.27 (m, 1H), 3.22 (s, 1H), 2.82 (q, *J* = 10.1 Hz, 1H), 2.51 (dt, *J* = 11.4, 8.8 Hz, 1H), 2.41 (ddd, *J* = 11.7, 8.4, 3.0 Hz, 1H), 2.21 (ddd, *J* = 11.7, 10.1, 3.1 Hz, 1H), 1.33 (s, 3H).

^{13}C NMR (151 MHz, $CDCl_3$) δ 165.23, 141.54, 133.17, 133.16, 130.72, 129.50, 129.41, 128.55, 128.53, 128.44, 128.21, 127.85, 127.80, 126.23, 126.20, 83.46, 81.82, 32.22, 27.38, 23.34, 23.00, 21.53, 14.13, 1.03.

HRMS (ESI^{+}) $C_{18}H_{18}NO_3Na$ [M+Na]$^{+}$: found 305.1154 , required 305.1149.

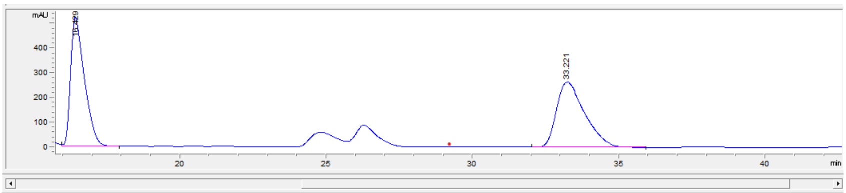

#	Time	Area	Height	Width	Area%	Symmetry
1	16.429	17658.8	522.3	0.5104	50.212	0.523
2	33.221	17509.9	263.2	1.0001	49.788	0.561

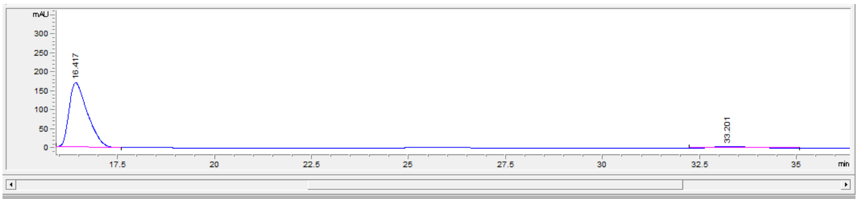

#	Time	Area	Height	Width	Area%	Symmetry
1	16.417	5528.8	168.9	0.49	94.955	0.536
2	33.201	293.8	4.6	0.9123	5.045	0.576

tert-Butyl ((1_S_,2_S_)-2-hydroxy-1-methyl-2-phenylcyclobutyl)carbamate (6c)

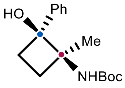

6c

Synthesized according to the general procedure **C** from enantioenriched ketone (*S*)-**5c** (15.5 mg, 0.05 mmol, 99:1 e.r.) using CH_2Cl_2 as solvent. The crude mixture was purified by flash column chromatography on silica gel (15% EtOAc in hexane) to afford the title compound **6c** as a clear oil (13.4 mg, 87% yield). The enantiomeric ratio was determined by SFC analysis on a chiral IC-3 (95:5 CO_2:*i*-PrOH, 1.0 mL/min, 20 °C, λ = 210 nm:, τ_{major} = 4.9 min, τ_{minor} = 6.8 min), 94.5:5.5 e.r.; $[\alpha]^D_{20}$ = +12.4 (c = 0.25, $CHCl_3$). An enantiospecificity (e.s.) of 91% was determined for the reaction. A single detectable diastereomer was observed by ^{1}H NMR and HPLC analysis.

^{1}H NMR (600 MHz, $CDCl_3$) δ 7.42 – 7.38 (m, 2H), 7.36 (d, *J* = 8.5, 2H), 7.30 – 7.27 (m, 1H), 5.37 (s, 1H), 3.68 (bs, 1H), 2.68 (m, 2H), 2.19 – 2.07 (m, 1H), 1.98 – 1.90 (m, 1H), 1.48 (s, 9H), 1.03 (s, 3H).

^{13}C NMR (151 MHz, $CDCl_3$) δ 156.22, 142.08, 128.23, 127.64, 126.25, 79.74, 59.92, 29.70, 28.47, 28.44, 23.97.

HRMS (ESI$^+$) $C_{16}H_{23}NO_3Na$ [M+Na]$^+$: found 300.1574 , required 300.1571.

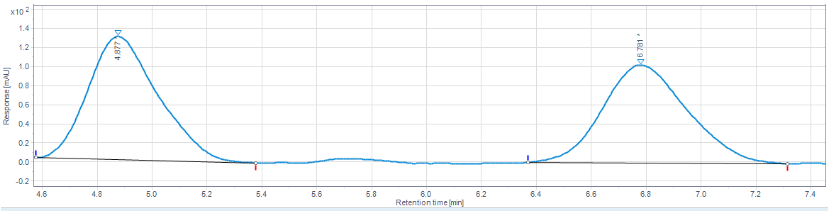

Injection Results

Peaks | Summary

#	Signal description	RT (min)	Area (mAU·s)	Area%	Height (mAU)	Height%	Amount	Concentration	Start time (min)	End time (min)
1	DAD1C,Sig=210,4 ...	4.877	2277.074	50.805	128.787	55.63			4.578	5.379
2	DAD1C,Sig=210,4 ...	6.781	2204.887	49.195	102.700	44.37			6.370	7.315

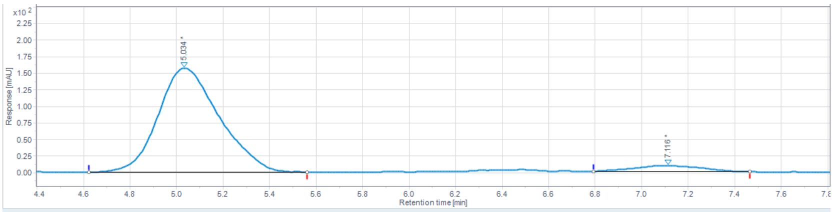

Injection Results

Peaks | Summary

#	Signal description	RT (min)	Area (mAU·s)	Area%	Height (mAU)	Height%	Amount	Concentration	Start time (min)	End time (min)
1	DAD1C,Sig=210,4 ...	5.034	2879.793	94.526	157.097	94.82			4.623	5.561
2	DAD1C,Sig=210,4 ...	7.116	166.779	5.474	8.581	5.18			6.792	7.465

tert-Butyl ((1*R*,2*S*)-2-hydroxy-1-isopropyl-2-phenylcyclobutyl)carbamate (6d)

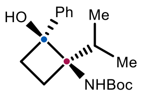

6d

Synthesized according to the general procedure **C** from enantioenriched ketone (*R*)-**5d** (15.3 mg, 0.05 mmol, 99:1 e.r.) using CH_2Cl_2 as solvent. The crude mixture was purified by flash column chromatography on silica gel (10-15% EtOAc in hexane) to afford the title compound **6d** as a clear oil (10.4 mg, 68% yield). The enantiomeric ratio was determined by SFC analysis on a chiral Daicel Chiralpak IB-N3 (95:5 CO_2:*i*-PrOH, 1.0 mL/min, 20 °C, λ = 210 nm:, τ_{minor} = 3.1 min, τ_{major} = 4.4 min), 89:11 e.r.; $[\alpha]^{D}_{20}$ = +20.7 (c = 0.21, $CHCl_3$). An enantiospecificity (e.s.) of 80% was determined for the reaction. A single detectable diastereomer was observed by ^{1}H NMR and HPLC analysis.

^{1}H NMR (400 MHz, $CDCl_3$) δ 7.60 – 7.53 (m, 2H), 7.35 (m, 2H), 7.29 – 7.26 (m, 1H), 5.02 (s, 1H), 3.86 (bs, 1H), 2.80 (ddd, *J* = 12.5, 10.6, 5.0 Hz, 1H), 2.58 (ddd, *J* = 12.4, 9.3, 5.0 Hz, 1H), 2.38 (ddd, *J* = 12.4, 10.6, 8.5 Hz, 1H), 1.92 (dt, *J* = 12.5, 8.9 Hz, 1H), 1.60 (m, 1H), 1.49 (s, 9H), 0.76 (d, *J* = 6.9 Hz, 3H), 0.43 (d, *J* = 6.6 Hz, 3H).

^{13}C NMR (151 MHz, $CDCl_3$) δ 156.87, 142.86, 127.90, 127.19, 126.42, 80.83, 79.65, 68.80, 33.48, 32.88, 29.71, 28.43, 28.38, 28.29, 28.20, 28.07, 24.84, 18.14, 17.70, 17.36, 16.47, 16.07, 14.13, 12.28, 1.78, 1.05, 1.02.

HRMS (ESI$^+$) $C_{18}H_{27}NO_3Na$ [M+Na]$^+$: found 328.1884 required 328.1884.

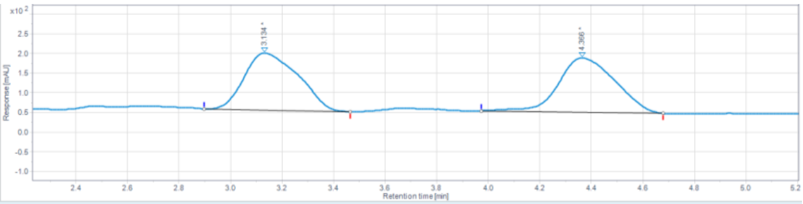

Injection Results

Peaks | Summary

#	Signal description	RT (min)	Area (mAU·s)	Area%	Height (mAU)	Height%	Amount	Concentration	Start time (min)	End time (min)
1	DAD1C,Sig=210,4 ...	3.134	2102.945	50.103	144.877	51.16			2.897	3.464
2	DAD1C,Sig=210,4 ...	4.366	2094.330	49.897	138.299	48.84			3.973	4.679

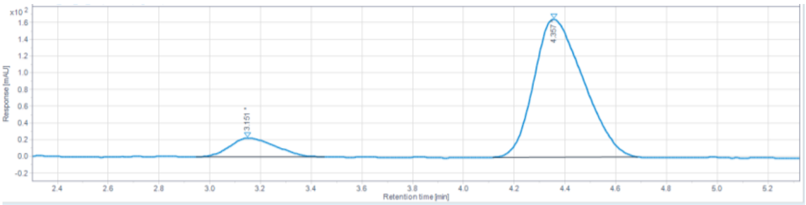

Injection Results

Peaks | Summary

#	Signal description	RT (min)	Area (mAU·s)	Area%	Height (mAU)	Height%	Amount	Concentration	Start time (min)	End time (min)
1	DAD1C,Sig=210,4 ...	3.151	281.141	11.165	22.574	12.03			2.948	3.454
2	DAD1C,Sig=210,4 ...	4.357	2236.940	88.835	165.057	87.97			4.117	4.689

tert-Butyl ((1*R*,2*S*)-2-hydroxy-1-isobutyl-2-phenylcyclobutyl) carbamate (6e)

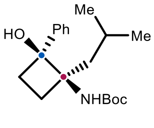

6e

Synthesized according to the general procedure **C** from enantioenriched (*R*)-**5e** (16 mg, 0.05 mmol, 96:4 e.r.). The crude mixture was purified by flash column chromatography on silica gel (15% EtOAc in Hexane) to afford the title compound **6e** as a clear oil (12.2 mg, 76% yield). The enantiomeric ratio was determined by HPLC analysis on a chiral Daicel Chiralpak IG-3 (95:5 CO_2:*i*-PrOH, 1.0 mL/min, 20 °C, λ = 210 nm:, τ_{major} = 6.5 min, τ_{minor} = 9.3 min), 93.5:6.5 e.r.; $[\alpha]^{D}_{20}$ = +20.8 (c = 0.13, $CHCl_3$). An enantiospecificity (e.s.) of 95% was determined for the reaction. A single detectable diastereomer was observed by ^{1}H NMR and HPLC analysis.

^{1}H NMR (400 MHz, $CDCl_3$) δ 7.51 – 7.45 (m, 2H), 7.38 – 7.32 (m, 2H), 7.24 (m, 1H), 5.01 (s, 1H), 4.12 (bs, 1H), 2.64 (ddd, *J* = 10.9, 9.1, 4.4 Hz, 1H), 2.37 – 2.18 (m, 2H), 1.97 (ddd, *J* = 11.9, 9.1, 7.3 Hz, 1H), 1.46 (s, 10H), 0.98 – 0.80 (m, 2H), 0.76 (d, *J* = 6.5 Hz, 3H), 0.66 (d, *J* = 6.5 Hz, 3H).

^{13}C NMR (151 MHz, $CDCl_3$) δ 156.82, 143.78, 142.53, 128.02, 127.36, 127.30, 127.20, 127.16, 127.06, 126.91, 126.39, 81.20, 80.00, 78.85, 64.97, 64.46, 42.94, 30.51, 29.71, 28.40, 25.37, 25.14, 24.97, 24.76, 24.10, 23.61, 14.13, 1.05, 1.02.

HRMS (ESI$^+$) $C_{18}H_{27}NO_3Na$ $[M+Na]^+$: found 342.2038, required 342.2040.

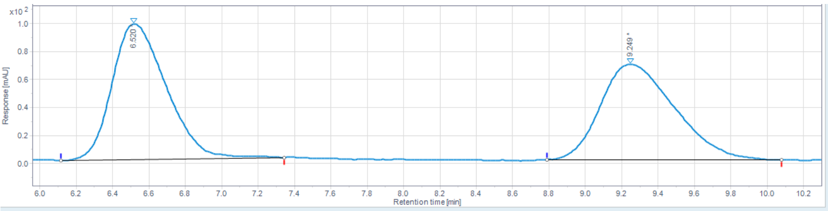

Injection Results

Peaks | Summary

#	Signal description	RT (min)	Area (mAU·s)	Area%	Height (mAU)	Height%	Amount	Concentration	Start time (min)	End time (min)
1	DAD1C,Sig=210,4 ...	6.520	2002.529	50.973	96.811	58.67			6.116	7.343
2	DAD1C,Sig=210,4 ...	9.249	1926.071	49.027	68.196	41.33			8.788	10.078

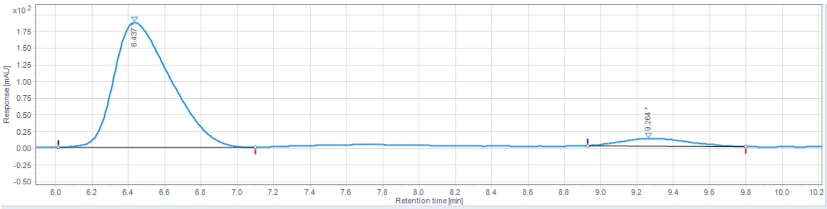

Injection Results

Peaks | Summary

#	Signal description	RT (min)	Area (mAU·s)	Area%	Height (mAU)	Height%	Amount	Concentration	Start time (min)	End time (min)
1	DAD1C,Sig=210,4 ...	6.437	4015.978	93.378	186.389	94.24			6.017	7.101
2	DAD1C,Sig=210,4 ...	9.264	284.790	6.622	11.395	5.76			8.929	9.802

tert-Butyl ((1_R_,2_S_)-2-hydroxy-1-(hydroxymethyl)-2-phenylcyclobutyl)carbamate (6f)

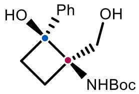

6f

Synthesized according to the general procedure **C** from enantioenriched ketone (_R_)-**5f** (14.7 mg, 0.05 mmol, 98:2 e.r.) using CH_2Cl_2 as solvent. The crude mixture was purified by flash column chromatography on silica gel (15% EtOAc in hexane) to afford the title compound **6f** as a clear oil (6.8 mg, 46% yield). The enantiomeric ratio was determined by HPLC analysis on a chiral Daicel Chiralpak AD-H (95:5 hexane:_i_-PrOH, 1.0 mL/min, 30 °C, λ = 230 nm:, τ_{major} = 19.7 min, τ_{minor} = 24 min), 90:10 e.r.; $[\alpha]^{D}_{20}$ = +36.0 (c = 0.18, $CHCl_3$). An enantiospecificity (e.s.) of 83% was determined for the reaction. A single detectable diastereomer was observed by ^{1}H NMR and HPLC analysis.

^{1}H NMR (600 MHz, $CDCl_3$) δ 7.42 – 7.27 (m, 4H), 6.05 (s, 1H), 4.39 (s, 1H), 3.48 – 3.42 (m, 1H), 3.28 – 3.18 (m, 2H), 2.71 (dt, _J_ = 12.6, 9.2 Hz, 1H), 2.50 (dddd, _J_ = 11.6, 10.3, 9.1, 1.2 Hz, 1H), 2.34 (ddd, _J_ = 11.8, 9.3, 3.8 Hz, 1H), 2.15 – 2.07 (m, 1H), 1.46 (d, _J_ = 3.4 Hz, 9H).

^{13}C NMR (151 MHz, $CDCl_3$) δ 157.37, 140.75, 128.58, 128.35, 126.03, 81.18, 80.42, 80.35, 79.72, 67.19, 66.25, 62.86, 53.43, 31.94, 29.71, 29.37, 28.38, 28.33, 28.24, 28.22, 25.59, 22.70, 21.00, 14.13, 1.79, 1.05, 1.03.

HRMS (ESI$^+$) $C_{16}H_{23}NO_4Na$ [M+Na]$^+$: found 316.1522, required 316.1519.

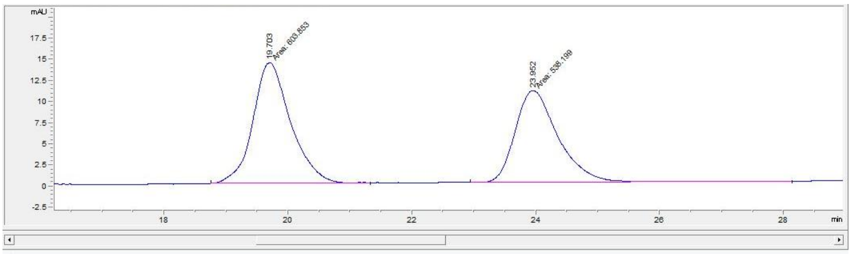

#	Time	Area	Height	Width	Area%	Symmetry
1	19.703	603.9	14.3	0.7053	52.874	0.78
2	23.952	538.2	10.9	0.8235	47.126	0.655

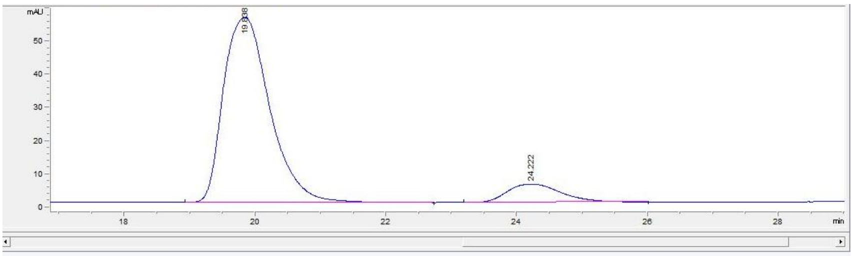

#	Time	Area	Height	Width	Area%	Symmetry
1	19.838	2750.6	56	0.7709	89.773	0.771
2	24.222	313.4	5.5	0.8355	10.227	0.771

***tert*-Butyl ((1R,2S)-1-benzyl-2-hydroxy-2-phenylcyclobutyl) carbamate (6g)**

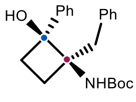

6g

Synthesized according to the general procedure **C** from enantioenriched ketone (*S*)-**5g** (18 mg, 0.05 mmol, 85.5:14.5 e.r.). The crude mixture was purified by flash column chromatography on silica gel (10-15% EtOAc in Hexane) to afford the title compound **6g** as a clear oil (13 mg, 72% yield). The enantiomeric ratio was determined by SFC analysis on a chiral Daicel Chiralpak IC-3 (85:5 CO_2:*i*-PrOH, 1.0 mL/min, 20 °C, λ = 210 nm:, τ_{major} = 8.5 min, τ_{minor} = 15.1 min), 83.5:16.5 e.r.; $[\alpha]^D{}_{20}$ = +10.6 (c = 0.08, $CHCl_3$). An enantiospecificity (e.s.) of 94% was determined for the reaction. A single detectable diastereomer was observed by ^{1}H NMR and HPLC analysis.

^{1}H NMR (400 MHz, $CDCl_3$) δ 7.64 – 7.57 (m, 2H), 7.40 (t, *J* = 7.7 Hz, 2H), 7.33 – 7.27 (m, 1H), 7.21 – 7.10 (m, 3H), 6.95 – 6.90 (m, 2H), 4.74 (s, 1H), 4.53 (bs, 1H), 3.00 (d, *J* = 13.9 Hz, 1H), 2.73 (ddd, *J* = 12.4, 9.1, 4.9 Hz, 1H), 2.42 – 2.30 (m, 1H), 2.19 – 2.06 (m, 2H), 1.84 (td, *J* = 11.5, 4.8 Hz, 1H), 1.48 (s, 9H).

^{13}C NMR (151 MHz, $CDCl_3$) δ 157.22, 142.63, 137.54, 129.82, 129.34, 128.27, 128.02, 127.81, 127.65, 127.43, 126.47, 126.21, 80.74, 80.24, 65.19, 40.12, 29.71, 29.53, 28.40, 27.37.

HRMS (ESI$^+$) $C_{19}H_{27}NO_4Na$ [M+Na]$^+$: found 376.1887, required 376.1884.

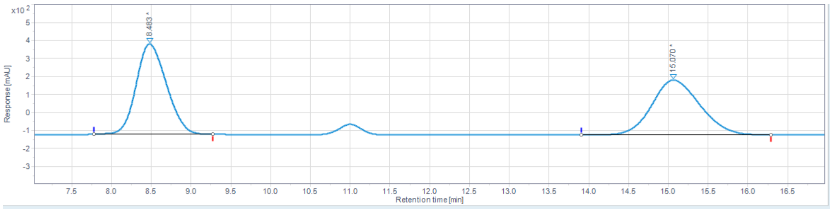

Injection Results

Peaks Summary

#	Signal description	RT (min)	Area (mAU·s)	Area%	Height (mAU)	Height%	Amount	Concentration	Start time (min)	End time (min)
1	DAD1C,Sig=210,4 ...	8.483	13222.514	50.824	501.861	62.19			7.778	9.273
2	DAD1C,Sig=210,4 ...	15.070	12793.518	49.176	305.107	37.81			13.906	16.290

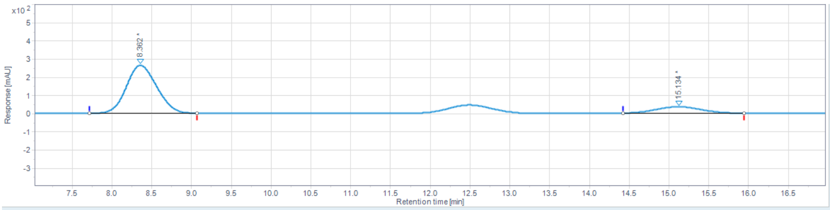

Injection Results

Peaks Summary

#	Signal description	RT (min)	Area (mAU·s)	Area%	Height (mAU)	Height%	Amount	Concentration	Start time (min)	End time (min)
1	DAD1C,Sig=210,4 ...	8.362	7023.685	83.492	264.171	88.29			7.716	9.068
2	DAD1C,Sig=210,4 ...	15.134	1388.695	16.508	35.036	11.71			14.422	15.943

tert-Butyl ((1_R_,2_S_)-2-hydroxy-1-(2-(methylthio)ethyl)-2-phenylcyclobutyl)carbamate (6h)

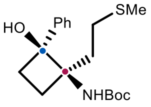

6h

Synthesized according to the general procedure **C** from enantioenriched ketone (_R_)-**5h** (17 mg, 0.05 mmol, 97.5:2.5 e.r.). The crude mixture was purified by flash column chromatography on silica gel (20-30% EtOAc in hexane) to afford the title compound **6h** as a clear oil (10.5 mg, 62% yield). The enantiomeric ratio was determined by HPLC analysis on a chiral Daicel Chiralpak AD-H (95:5 hexane:i-PrOH, 1.0 mL/min, 30 °C, λ = 230 nm:, τ_{major} = 8.7 min, τ_{minor} = 10.4 min), 90:10 e.r.; $[\alpha]^{D}_{20}$ = +34.0 (c = 0.18, $CHCl_3$). An enantiospecificity (e.s.) of 84% was determined for the reaction. A single detectable diastereomer was observed by ^{1}H NMR and HPLC analysis.

^{1}H NMR (600 MHz, $CDCl_3$) δ 7.50 – 7.47 (m, 2H), 7.39 – 7.35 (m, 2H), 7.31 – 7.27 (m, 1H), 5.31 (s, 1H), 3.56 (s, 1H), 2.68 (ddd, _J_ = 12.5, 9.2, 6.5 Hz, 1H), 2.39 (dd, _J_ = 11.3, 6.7 Hz, 1H), 2.26 (ddd, _J_ = 13.1, 10.9, 5.3 Hz, 2H), 2.12 – 2.05 (m, 1H), 1.97 (ddd, _J_ = 12.1, 9.2, 6.6 Hz, 1H), 1.86 (s, 3H), 1.76 (dt, _J_ = 10.8, 4.7 Hz, 1H), 1.65 – 1.58 (m, 1H), 1.48 (s, 9H).

^{13}C NMR (151 MHz, $CDCl_3$) δ 141.83, 128.31, 127.73, 126.27, 63.71, 53.43, 34.61, 34.14, 31.94, 29.81, 29.71, 28.59, 28.41, 28.35, 22.71, 22.35, 15.42, 14.13, 14.07, 1.03.

HRMS (ESI$^+$) $C_{18}H_{27}NO_3SNa$ [M+Na]$^+$: found 360.1607, required 360.1604.

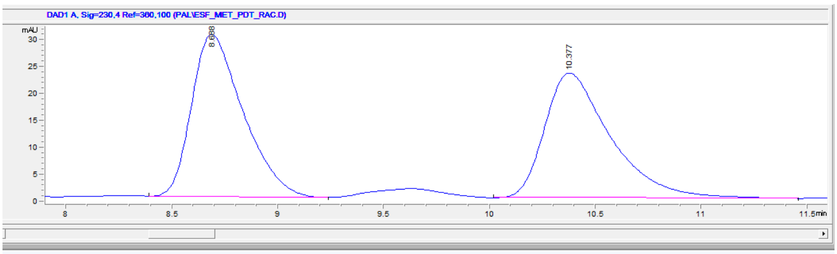

#	Time	Area	Height	Width	Area%	Symmetry
1	8.688	514.2	30.3	0.254	50.611	0.617
2	10.377	501.8	23.2	0.3203	49.389	0.566

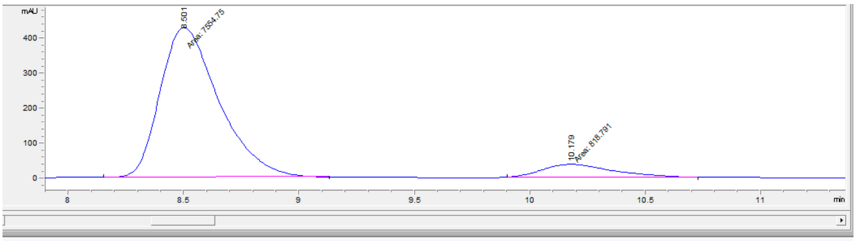

#	Time	Area	Height	Width	Area%	Symmetry
1	8.501	7554.7	427.9	0.2942	90.222	0.668
2	10.179	818.8	37.4	0.3646	9.778	0.641

tert-Butyl ((1S,2S)-1-(4-((tert-butoxycarbonyl)amino)butyl)-2-hydroxy-2-phenylcyclobutyl)carbamate (6i)

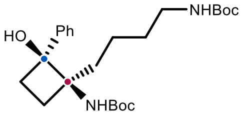

6i

Synthesized according to the general procedure **C** from enantioenriched ketone *(S)*-**5i** (17 mg, 0.05 mmol, 94.5:5.5 e.r.). The crude mixture was purified by flash column chromatography on silica gel (15-20% EtOAc in hexane) to afford the title compound **6i** as a clear oil (14.5 mg, 85% yield). The enantiomeric ratio was determined by HPLC analysis on a chiral Daicel Chiralpak AD-H (90:10 hexane:i-PrOH, 1.0 mL/min, 30 °C, λ = 230 nm:, τ_{minor} = 7.3 min, τ_{major}= 10.5 min), 91:9 e.r.; $[\alpha]^{D}_{20}$ = +2.1 (c = 0.29, $CHCl_3$). An enantiospecificity (e.s.) of 92% was determined for the reaction. A single detectable diastereomer was observed by ^{1}H NMR and HPLC analysis.

^{1}H NMR (600 MHz, $CDCl_3$) δ 7.50 – 7.44 (m, 2H), 7.36 (dd, *J* = 8.4, 7.0 Hz, 2H), 7.31 – 7.27 (m, 1H), 5.14 (s, 1H), 4.37 (s, 1H), 3.75 (s, 1H), 3.24 – 3.07 (m, 1H), 2.93 (d, *J* = 7.9 Hz, 2H), 2.66 (ddd, *J* = 12.5, 9.2, 6.9 Hz, 1H), 2.49 – 2.39 (m, 1H), 2.20 (td, *J* = 10.1, 4.9 Hz, 1H), 1.91 (ddd, *J* = 12.0, 9.2, 6.2 Hz, 1H), 1.50 – 1.39 (m, 18H), 1.32 – 1.21 (m, 4H), 1.15 (dd, *J* = 12.9, 7.4 Hz, 2H).

^{13}C NMR (151 MHz, $CDCl_3$) δ 156.37, 155.95, 142.05, 128.12, 127.52, 126.37, 79.87, 78.97, 63.92, 40.07, 34.45, 30.94, 29.93, 29.43, 28.43, 28.24, 20.65.

HRMS (ESI$^+$) $C_{24}H_{38}N_2O_5Na$ [M+Na]$^+$: found 457.2670, required 457.2673.

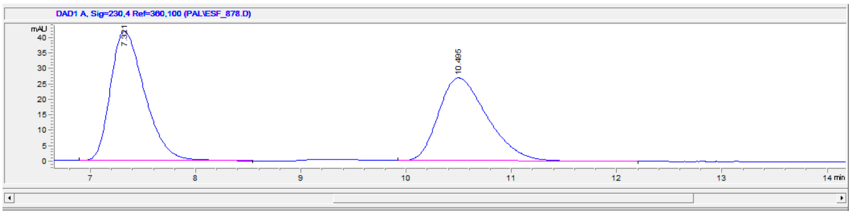

#	Time	Area	Height	Width	Area%	Symmetry
1	7.321	945.2	41.8	0.3479	51.948	0.646
2	10.495	874.3	26.9	0.4992	48.052	0.656

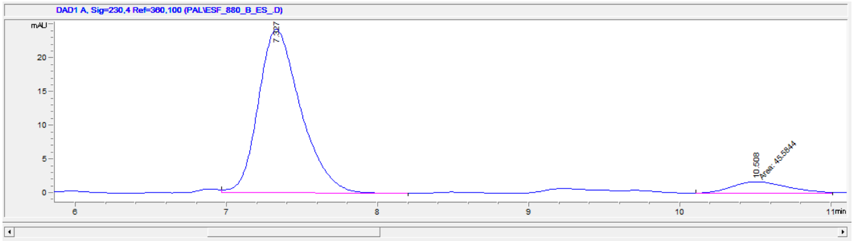

#	Time	Area	Height	Width	Area%	Symmetry
1	7.327	474.1	24.3	0.2938	91.229	0.698
2	10.508	45.6	1.7	0.4366	8.771	0.781

tert-Butyl 4-(((1R,2S)-1-((tert-butoxycarbonyl)amino)-2-hydroxy-2-phenylcyclobutyl)methyl)-1H-imidazole-1-carboxylate (6j)

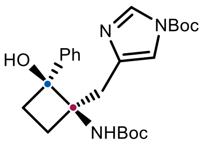

6j

Synthesized according to the general procedure **C** from enantioenriched ketone *(R)*-**5j** (22.2 mg, 0.05 mmol, 90.0:10.0 e.r). The crude mixture was purified by flash column chromatography on silica gel (50% EtOAc in hexane) to afford the title compound **6j** as a clear oil (9.5 mg, 43% yield). The enantiomeric ratio was determined by HPLC analysis on a chiral Daicel Chiralpak IC-3 (80:20 hexane:i-PrOH, 2.0 mL/min, 30 °C, λ = 254 nm:, τ_{major} = 25.8 min, τ_{minor} = 31.1 min), 88.5:11.5 e.r.; $[\alpha]^{D}_{20}$ = +4.5 (c = 0.27, $CHCl_3$). An enantiospecificity (e.s.) of 96% was determined for the reaction. A single detectable diastereomer was observed by ^{1}H NMR and HPLC analysis.

^{1}H NMR (600 MHz, $CDCl_3$) δ 7.93 (d, *J* = 1.3 Hz, 1H), 7.53 – 7.49 (m, 2H), 7.34 – 7.30 (m, 2H), 7.26 – 7.23 (m, 1H), 6.73 – 6.65 (m, 1H), 5.92 (s, 1H), 5.39 – 5.31 (m, 1H), 2.78 – 2.71 (m, 1H), 2.70 (d, *J* = 8.8 Hz, 1H), 2.58 (d, *J* = 14.9 Hz, 1H), 2.40 (d, *J* = 14.9 Hz, 1H), 2.28 (ddd, *J* = 11.3, 9.7, 6.0 Hz, 1H), 2.13 – 2.03 (m, 1H), 1.47 (s, 9H).

^{13}C NMR (151 MHz, $CDCl_3$) δ 157.57, 154.07, 147.07, 142.32, 139.24, 136.35, 128.05, 127.47, 126.75, 114.73, 85.46, 80.18, 64.01, 33.99, 28.55, 28.03.

HRMS (ESI^+) $C_{24}H_{33}N_3O_5Na$ $[M+Na]^+$: found 466.2317, required 466.2318.

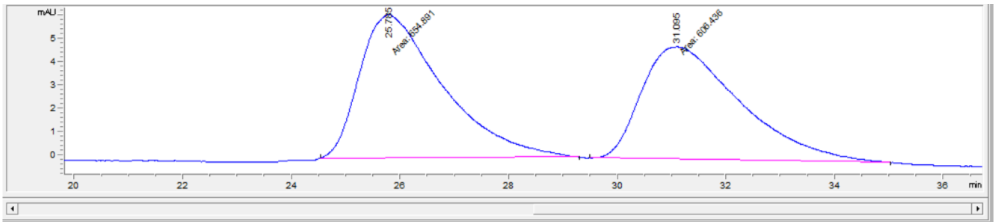

#	Time	Area	Height	Width	Area%	Symmetry
1	25.785	654.9	6.2	1.774	51.921	0.526
2	31.095	606.4	4.8	2.0918	48.079	0.547

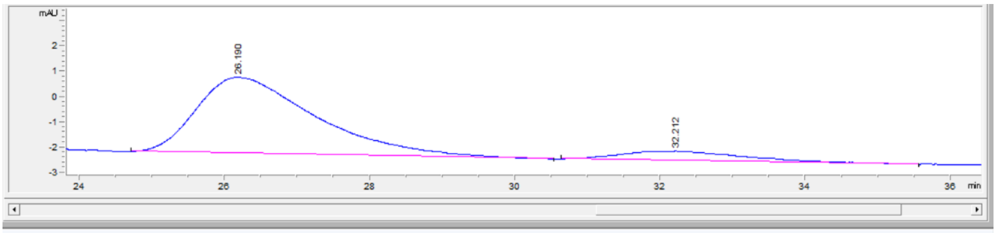

#	Time	Area	Height	Width	Area%	Symmetry
1	26.19	346.5	3	1.4148	88.653	0.505
2	32.212	44.3	3.7E-1	1.3996	11.347	0.713

tert-butyl ethyl((1S,2S)-2-hydroxy-1-methyl-2-phenylcyclobutyl)carbamate (6k)

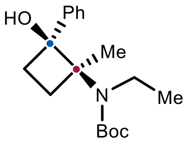

6k

Synthesized according to the general procedure **C** from enantioenriched ketone *(R)*-**5k** (15.3 mg, 0.05 mmol, 96:4 e.r). The crude mixture was purified by flash column chromatography on silica gel (30% EtOAc in hexane) to afford the title compound **6k** as a clear oil (9.2 mg, 60% yield). The enantiomeric ratio was determined by HPLC analysis on a chiral Daicel Chiralpak OD-H (95:5 hexane:i-PrOH, 1.0 mL/min, 30 °C, λ = 230 nm:, τ_{minor} = 7.1 min, τ_{major} = 10.6 min), 87:13 e.r.; $[\alpha]^{D}_{20}$ = -2.5 (c = 0.24, $CHCl_3$). An enantiospecificity (e.s.) of 80% was determined for the reaction. A single detectable diastereomer was observed by ^{1}H NMR and HPLC analysis.

^{1}H NMR (600 MHz, $CDCl_3$) δ 7.63 – 7.52 (m, 2H), 7.35 (t, *J* = 7.7 Hz, 2H), 7.27 (s, 1H), 4.06 (s, 1H), 3.07 (ddq, *J* = 28.8, 14.6, 7.3 Hz, 2H), 2.68 (q, *J* = 10.0 Hz, 1H), 2.47 (dt, *J* = 12.0, 8.9 Hz, 1H), 2.00 (dddd, *J* = 33.6, 11.9, 9.2, 3.6 Hz, 2H), 1.42 (s, 9H), 1.27 – 1.19 (m, 6H).

^{13}C NMR (151 MHz, $CDCl_3$) δ 156.73, 142.92, 127.89, 127.27, 127.11, 81.19, 80.17, 67.36, 40.08, 31.10, 29.46, 28.55, 22.72, 15.55.

HRMS (ESI$^+$) $C_{18}H_{27}NNaO_3$ [M+Na]$^+$: found 328.1889, required 328.1884.

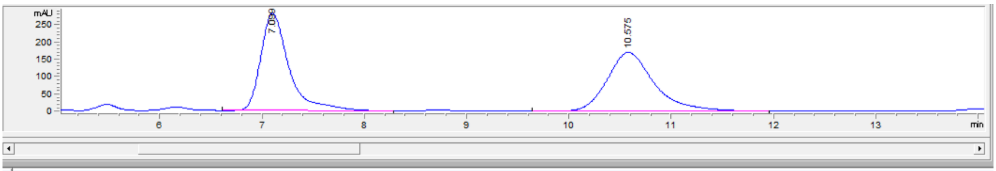

#	Time	Area	Height	Width	Area%	Symmetry
1	7.099	5394.9	281.2	0.2878	49.900	0.66
2	10.575	5416.5	169.5	0.4767	50.100	0.77

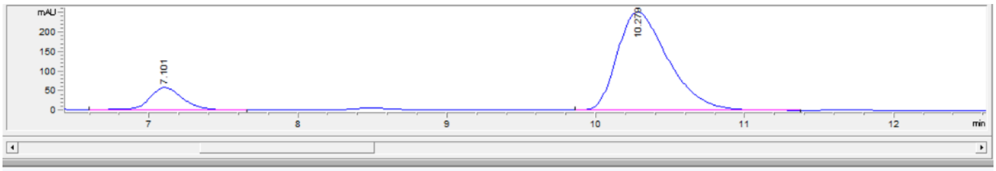

#	Time	Area	Height	Width	Area%	Symmetry
1	7.101	903.8	57.5	0.2395	13.155	0.852
2	10.279	5966.8	250.9	0.3655	86.845	0.631

E.1 X-Ray Crystallographic Data for Compound 4

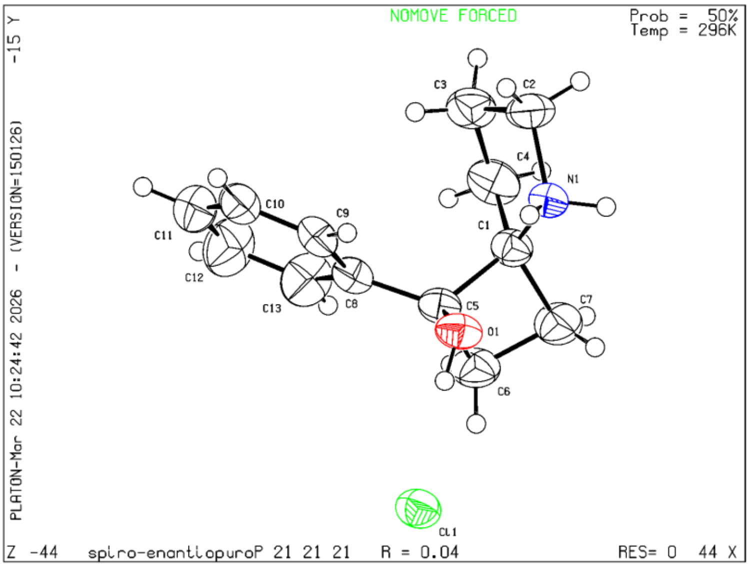

A specimen of compound **4** ($C_{13}H_{18}ClNO$), obtained upon Boc deprotection of product **2a** (98:2 e.r.), was used for the X-ray crystallographic analysis. The X-ray intensity data were measured (λ = 0.71073 Å). The total exposure time was 20.33 hours. The frames were integrated with the Bruker SAINT software package using a narrow-frame algorithm. The integration of the data using an orthorhombic unit cell yielded a total of 18357 reflections to a maximum θ angle of 26.00° (0.81 Å resolution), of which 2525 were independent (average redundancy 7.270, completeness = 99.8%, R_{int} = 4.99%, R_{sig} = 2.52%) and 2417 (95.72%) were greater than $2\sigma(F^2)$. The final cell constants of a = 7.4936(5) Å, b = 12.8569(9) Å, c =13.3317(10) Å, volume = 1284.44(16) $Å^3$, are based upon the refinement of the XYZ-centroids of 7901 reflections above 20 σ(I) with 4.401° < 2θ < 56.30°. Data were corrected for absorption effects using the Multi-Scan method (SADABS). The ratio of minimum to maximum apparent transmission was 0.771.

The structure was solved and refined using the Bruker SHELXTL Software Package, using the space group P 21 21 21, with Z = 4 for the formula unit, $C_{13}H_{18}ClNO$. The final anisotropic full-matrix least-squares refinement on F^2 with 157 variables converged at R1 =3.91%, for the observed data and wR2 = 9.88% for all data. The goodness-of-fit was 1.112. The largest peak in the final difference electron density synthesis was 0.189 $e^-/Å^3$ and the largest hole was -0.176 $e^-/Å^3$ with an RMS deviation of 0.038 $e^-/Å^3$. On the basis of the final model, the calculated density was 1.240 g/cm^3 and F(000), 512 e^-. Flack parameter for S,S, absolute configuration was determined as -0.03 (3) using 953 quotients [(I+)-(I-)]/[(I+)+(I-)] (Parsons, Flack and Wagner, Acta Cryst. B69 (2013) 249-259).

The CIF file, including structure factors, has been deposited within the CCDC service, deposition Number *2540243*.

Table S1. Sample and crystal data for compound (*S,S*)-**4.**

**Identification code**	spirocyclo4	
**Chemical formula**	$C_{13}H_{18}ClNO$	
**Formula weight**	239.73 g/mol	
**Temperature**	296(2) K	
**Wavelength**	0.71073 Å	
**Crystal system**	orthorhombic	
**Space group**	P 21 21 21	
**Unit cell dimensions**	a = 7.4936(5) Å	α = 90°
	b = 12.8569(9) Å	β = 90°
	c = 13.3317(10) Å	γ = 90°
**Volume**	1284.44(16) $Å^3$	
**Z**	4	
**Density (calculated)**	1.240 g/cm^3	
**Absorption coefficient**	0.277 mm^{-1}	
**F(000)**	512	

Table S2. Data collection and structure refinement for compound (*S,S*)-**4.**

**Theta range for data collection**	2.20 to 26.00°	
**Index ranges**	-9<=h<=9, -15<=k<=15, -16<=l<=16	
**Reflections collected**	18357	
**Independent reflections**	2525 [R(int) = 0.0499]	
**Coverage of independent reflections**	99.8%	
**Absorption correction**	Multi-Scan	
**Structure solution technique**	direct methods	
**Structure solution program**	SHELXT 2014/5 (Sheldrick, 2014)	
**Refinement method**	Full-matrix least-squares on F^2	
**Refinement program**	SHELXL-2017/1 (Sheldrick, 2017)	
**Function minimized**	$\Sigma w(F_o^2 - F_c^2)^2$	
**Data / restraints / parameters**	2525 / 0 / 157	
**Goodness-of-fit on F^2**	1.112	
**Final R indices**	2417 data; I>2σ(I)	R1 = 0.0391, wR2 = 0.0977
	all data	R1 = 0.0408, wR2 = 0.0988
**Weighting scheme**	$w=1/[\sigma^2(F_o^2)+(0.0467P)^2+0.2732P]$ where $P=(F_o^2+2F_c^2)/3$	
**Absolute structure parameter**	-0.03(3)	

**Largest diff. peak and hole**	0.189 and -0.176 eÅ^{-3}
**R.M.S. deviation from mean**	0.038 eÅ^{-3}

Table S3. Atomic coordinates and equivalent isotropic atomic displacement parameters (Å^2) for compound (*S*,*S*)-**4.**

U(eq) is defined as one third of the trace of the orthogonalized U_{ij} tensor.

	**x/a**	**y/b**	**z/c**	**U(eq)**
Cl1	0.16206(10)	0.23418(6)	0.33492(6)	0.0519(2)
O1	0.5067(3)	0.32412(16)	0.41879(16)	0.0463(5)
N1	0.8556(4)	0.34904(19)	0.4485(2)	0.0419(5)
C5	0.5604(4)	0.4216(2)	0.3779(2)	0.0402(6)
C9	0.4439(4)	0.5000(2)	0.5368(2)	0.0474(7)
C8	0.4764(4)	0.5106(2)	0.4350(2)	0.0419(6)
C1	0.7686(4)	0.4145(2)	0.3689(2)	0.0431(6)
C2	0.9662(5)	0.4177(3)	0.5146(3)	0.0562(8)
C6	0.5564(5)	0.4159(3)	0.2620(2)	0.0571(9)
C10	0.3772(4)	0.5820(3)	0.5926(3)	0.0571(8)
C4	0.8722(5)	0.5155(3)	0.3741(3)	0.0606(9)
C13	0.4387(6)	0.6058(3)	0.3918(3)	0.0673(10)
C3	0.9193(5)	0.5264(2)	0.4835(3)	0.0605(9)
C7	0.7433(5)	0.3661(3)	0.2646(2)	0.0626(9)
C11	0.3411(5)	0.6756(3)	0.5483(3)	0.0695(10)
C12	0.3716(7)	0.6869(3)	0.4480(3)	0.0814(13)

Table S4. Bond lengths (Å) for compound (*S*,*S*)-**4.**

O1-C5	1.425(3)	O1-H1O	0.84(4)
N1-C2	1.497(4)	N1-C1	1.504(4)
N1-H1N	0.95(4)	N1-H2N	0.81(4)
C5-C8	1.511(4)	C5-C6	1.548(4)
C5-C1	1.568(4)	C9-C10	1.384(5)
C9-C8	1.386(4)	C9-H9	0.93
C8-C13	1.382(4)	C1-C4	1.514(4)
C1-C7	1.536(4)	C2-C3	1.500(5)
C2-H2A	0.97	C2-H2B	0.97
C6-C7	1.540(5)	C6-H6A	0.97
C6-H6B	0.97	C10-C11	1.367(5)
C10-H10	0.93	C4-C3	1.506(5)
C4-H4A	0.97	C4-H4B	0.97

C13-C12	1.378(5)	C13-H13	0.93
C3-H3A	0.97	C3-H3B	0.97
C7-H7A	0.97	C7-H7B	0.97
C11-C12	1.364(6)	C11-H11	0.93
C12-H12	0.93		

Table S5. Bond angles (°) for compound **(S,S)-4.**

C5-O1-H1O	108.(3)	C2-N1-C1	109.0(2)
C2-N1-H1N	108.(3)	C1-N1-H1N	112.(3)
C2-N1-H2N	109.(3)	C1-N1-H2N	112.(3)
H1N-N1-H2N	107.(4)	O1-C5-C8	110.9(2)
O1-C5-C6	109.5(3)	C8-C5-C6	122.0(3)
O1-C5-C1	105.0(2)	C8-C5-C1	119.8(3)
C6-C5-C1	86.5(2)	C10-C9-C8	121.0(3)
C10-C9-H9	119.5	C8-C9-H9	119.5
C13-C8-C9	117.3(3)	C13-C8-C5	123.1(3)
C9-C8-C5	119.4(3)	N1-C1-C4	103.0(2)
N1-C1-C7	117.8(3)	C4-C1-C7	116.9(3)
N1-C1-C5	114.2(2)	C4-C1-C5	117.2(3)
C7-C1-C5	88.3(2)	N1-C2-C3	104.9(3)
N1-C2-H2A	110.8	C3-C2-H2A	110.8
N1-C2-H2B	110.8	C3-C2-H2B	110.8
H2A-C2-H2B	108.8	C7-C6-C5	88.8(2)
C7-C6-H6A	113.8	C5-C6-H6A	113.8
C7-C6-H6B	113.8	C5-C6-H6B	113.8
H6A-C6-H6B	111.1	C11-C10-C9	120.6(3)
C11-C10-H10	119.7	C9-C10-H10	119.7
C3-C4-C1	104.2(3)	C3-C4-H4A	110.9
C1-C4-H4A	110.9	C3-C4-H4B	110.9
C1-C4-H4B	110.9	H4A-C4-H4B	108.9
C12-C13-C8	121.2(3)	C12-C13-H13	119.4
C8-C13-H13	119.4	C2-C3-C4	103.6(3)
C2-C3-H3A	111.0	C4-C3-H3A	111.0
C2-C3-H3B	111.0	C4-C3-H3B	111.0
H3A-C3-H3B	109.0	C1-C7-C6	87.9(3)
C1-C7-H7A	114.0	C6-C7-H7A	114.0
C1-C7-H7B	114.0	C6-C7-H7B	114.0
H7A-C7-H7B	111.2	C12-C11-C10	119.0(4)
C12-C11-H11	120.5	C10-C11-H11	120.5

C11-C12-C13	120.9(4)	C11-C12-H12	119.6
C13-C12-H12	119.6		

Table S6. Anisotropic atomic displacement parameters (Å^2) for compound **(S,S)-4.**
The anisotropic atomic displacement factor exponent takes the form: $-2\pi^2[h^2 a^{*2} U_{11} + ... + 2 h k a^* b^* U_{12}]$

	U_{11}	U_{22}	U_{33}	U_{23}	U_{13}	U_{12}
Cl1	0.0428(4)	0.0646(4)	0.0482(4)	-0.0017(3)	-0.0009(3)	-0.0070(3)
O1	0.0454(12)	0.0438(11)	0.0495(12)	0.0064(9)	-0.0110(10)	-0.0100(9)
N1	0.0358(12)	0.0447(13)	0.0453(13)	-0.0032(11)	-0.0051(11)	-0.0029(11)
C5	0.0410(14)	0.0445(15)	0.0350(13)	0.0058(12)	-0.0053(11)	-0.0055(12)
C9	0.0352(14)	0.0547(17)	0.0522(17)	0.0050(14)	-0.0049(13)	0.0056(13)
C8	0.0366(14)	0.0434(14)	0.0457(15)	0.0057(12)	-0.0062(12)	-0.0023(11)
C1	0.0408(14)	0.0503(15)	0.0383(14)	0.0028(12)	-0.0013(12)	-0.0029(13)
C2	0.0561(19)	0.0578(18)	0.0549(18)	-0.0090(15)	-0.0152(16)	-0.0093(15)
C6	0.060(2)	0.075(2)	0.0363(15)	0.0016(15)	-0.0111(14)	-0.0013(18)
C10	0.0404(16)	0.075(2)	0.0555(18)	-0.0101(16)	-0.0032(14)	0.0056(15)
C4	0.0514(19)	0.064(2)	0.067(2)	0.0141(17)	0.0017(15)	-0.0170(16)
C13	0.091(3)	0.0517(19)	0.059(2)	0.0135(16)	-0.0021(19)	0.0044(19)
C3	0.0537(19)	0.0479(17)	0.080(2)	-0.0028(17)	-0.0094(18)	-0.0139(15)
C7	0.059(2)	0.090(3)	0.0383(16)	-0.0094(16)	-0.0009(15)	0.000(2)
C11	0.063(2)	0.058(2)	0.087(3)	-0.0171(19)	-0.006(2)	0.0074(19)
C12	0.109(4)	0.0466(18)	0.088(3)	0.0056(19)	-0.007(3)	0.016(2)

Table S7. Hydrogen atomic coordinates and isotropic atomic displacement parameters (Å^2) for compound **(S,S)-4.**

	x/a	y/b	z/c	U(eq)
H9	0.4673	0.4368	0.5681	0.057
H2A	1.0924	0.4044	0.5046	0.067
H2B	0.9374	0.4063	0.5847	0.067
H6A	0.4643	0.3705	0.2356	0.069
H6B	0.5532	0.4834	0.2294	0.069
H10	0.3568	0.5734	0.6609	0.069
H4A	0.9789	0.5120	0.3331	0.073
H4B	0.7995	0.5735	0.3518	0.073
H13	0.4591	0.6154	0.3236	0.081
H3A	1.0199	0.5730	0.4924	0.073
H3B	0.8187	0.5525	0.5217	0.073
H7A	0.7420	0.2906	0.2646	0.075
H7B	0.8244	0.3929	0.2141	0.075

H11	0.2964	0.7306	0.5859	0.083
H12	0.3467	0.7501	0.4171	0.098
H1O	0.410(5)	0.307(3)	0.391(3)	0.063(11)
H1N	0.931(6)	0.297(3)	0.421(3)	0.074(12)
H2N	0.782(6)	0.319(3)	0.483(3)	0.070(13)

E.2 Relative Configuration Assignment of Compound 6c

The relative configuration of the two stereocenters of product **6c** was assigned by NOE analysis, using the DPFGSE-NOE 1D sequence.[5] Both the ^{1}H NMR and NOE spectra were recorded at -10 °C to minimize conformational exchange of the Boc moiety. No additional conformers were observed at -10 °C relative to the room-temperature NMR spectra. This is consistent with the presence of a strong hydrogen bond between the OH group and the Boc carbonyl, which may bias the conformational equilibrium toward a single dominant conformer. Upon saturation of the methyl signal at 1.01 ppm (Figure S6), strong NOEs were observed for the NH signal and for the *ortho* hydrogens of the C1 phenyl ring. Additional NOEs were observed for the two hydrogens on cyclobutane ring lying on the same side of the methyl group. These observations indicate that the methyl group is *syn* to the phenyl ring on C_1. Accordingly, the relative configuration was assigned as *R**,*R**.

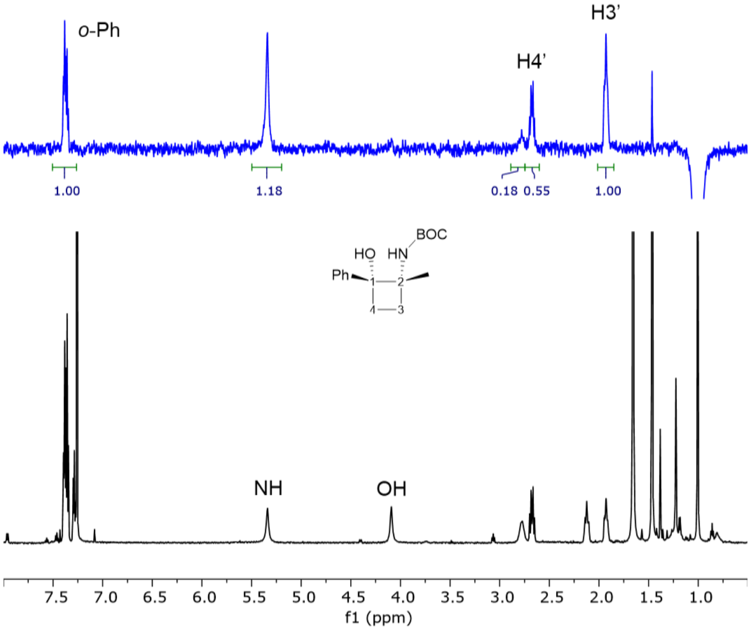

Figure S6. DPFGSE-NOE of compound **6c** (600 MHz in $CDCl_3$ at -10°C.). Bottom: ^{1}H control spectrum. Top: NOE spectrum with saturation of methyl at 1.01 ppm (50 Hz-wide R-SNOB shaped pulse). Key integrations, relative to the NOE intensity of $H_{3'}$, are reported.

A DFT conformational search was consistent with a highly restricted conformational space for **6c**, supporting the NOE-based stereochemical assignment. Taking the NMR-based relative configuration as reference, the conformational space of compound **6c** was first explored using CREST (version 2.12)[6] at the GFN2-xTB[7] level, with acetonitrile included as an implicit solvent through the ALPB model.[8] The resulting ensemble was clustered by principal component analysis of the dihedral angles. Only one conformer was found within a 2 kcal mol^{-1} energy window, indicating a highly restricted conformational space for **6c**. A second conformer, differing in the orientation of the Boc group, was located 2.42 kcal mol^{-1} higher in energy. The relevant structures were then fully optimized with Gaussian 16[9] at the IEFPCM-B3LYP-GD3BJ/6-311G(d,p) level, using acetonitrile as the implicit solvent. Frequency analysis confirmed that all optimized structures were true minima. Thermochemical corrections and Boltzmann populations were obtained using the mRRHO[10] approach (cut-off = 100 cm−1, 298 K, 1 M, 1 bar). To benchmark the computational protocol, the same conformational analysis was carried out on compound **2a**, the Boc precursor of derivative **4**, whose absolute configuration was established by X-ray diffraction (see Section E.1). In contrast to **6c**, two conformers of **2a**, arising from different pyrrolidine-ring conformations, were found within 2 kcal mol^{-1}. Table S8 summarizes the relative energies and Boltzmann populations of the conformers of **6c** and **2a**.

Table S8. Relative energies and Boltzmann populations of the conformers of **6c** and **2a**, calculated at the IEFPCM(acetonitrile)-B3LYP-GD3BJ/6-311G(d,p) level of theory. ΔH° and ΔG° values are given in kcal mol^{-1}.

Conf.	H°	d-G° ($100\ cm^{-1}$)	ΔH°	d-ΔG° ($100\ cm^{-}$)	Pop % (on d-ΔG°)
**6c-c1**	-903.941177	-904.007585	0.00	0.00	99
**6c-c2**	-903.937433	-904.003713	2.35	2.42	1
**2a-c1**	-981.346869	-981.414906	0.00	0.00	95
**2a-c2**	-981.343924	-981.412095	1.85	1.76	5

E.3 Absolute Configuration Assignment of Compound 6c

Given the presence of UV chromophores, the absolute configuration of product **6c** was determined by TD-DFT simulation of the electronic circular dichroism (ECD) spectrum.[11] Because the absolute configuration of **2a** had been established unambiguously by X-ray analysis of derivative **4**, the following analysis was performed on both compounds to assess the reliability of the computational approach. The UV and ECD spectra of **6c** and **2a** were recorded on a sample with 94.5:5.5 e.r and 98:2 e.r., respectively, using a JASCO J-810 spectropolarimeter. Data were acquired with a 0.1 cm path-length cell in the 185–400 nm range, using hypergrade LC-MS acetonitrile (Merck), at a concentration of ≈ $1\cdot10^{-4}$ M, in order to obtain a maximum absorbance between 0.9 and 1.2 in the UV/vis spectrum. The spectra represent averages of 16 scans acquired at a scan rate of 50 $nm\cdot min^{-1}$ (Figure S7).

The ECD spectrum of **6c** shows two positive bands: a weak one centered at ≈210 nm and a stronger one at 197 nm. The spectrum of **2a** shows two strong positive bands at 224 and 206 nm, followed by a negative band at 190 nm. Below 185 nm, the ECD spectra are unreliable due to the strong absorbance of acetonitrile. From the comparison of the two UV spectra, it appears that the spectrum of **6c** is slightly blue-shifted with respect to **2a**.

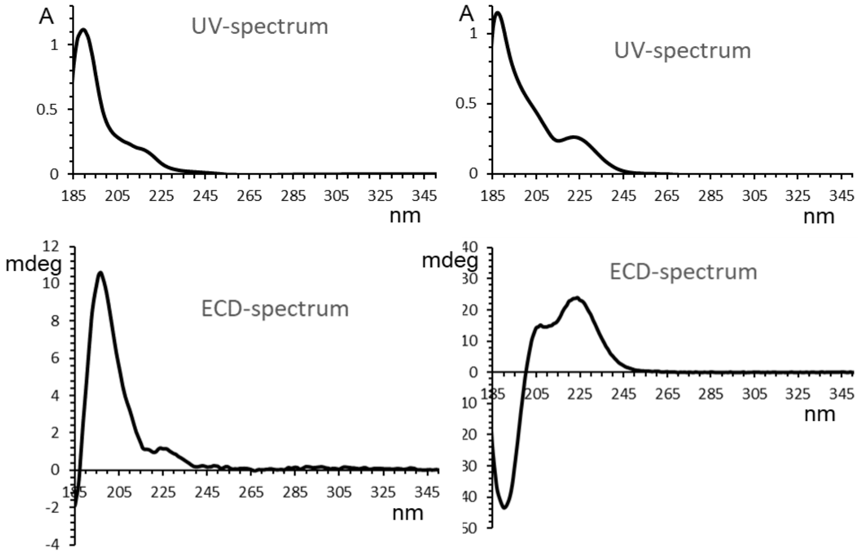

Figure S7. UV and ECD spectra of **6c** (left) and **2a** (right) in acetonitrile (≈ $1 \cdot 10^{-4}$ M, 0.1 path length).

The TD-DFT simulations of the UV and ECD spectra of **6c** and **2a** were performed using the geometries of the conformations reported in Table S8. To ensure methodological redundancy, calculations were carried out with the ωB97X-D functional, which includes empirical dispersion, and with CAM-B3LYP, which incorporates long-range corrections. All TD-DFT calculations employed the 6-311++G(2d,p) basis set, known to provide good performance at a reasonable computational cost.[12] The solvent (acetonitrile) was included using the IEF-PCM formalism. For each conformation, 50 transitions were computed to cover the 3.0–8.0 eV energy range (≈ 413–155 nm). Each transition was convoluted with a Gaussian function (0.25 eV half-width) to obtain the simulated spectra.

The (1*S*,4*S*) absolute configuration was used in all calculations. The UV/ECD spectra of the two conformations of **2a** were weighted and summed using Boltzmann populations at 298 K, based on the relative energies reported in Table S8 (corrected ΔG° energies). The resulting simulated spectra were then converted into the wavelength (nm) scale, and compared with the experimental spectra.

In both cases (Figures S8-S9) the agreement with the experimental spectra was very good and reliably supports assignment of the (*S*,*S*) absolute configuration to **6c**. The successful simulation of the known compound **2a** further supports the reliability of the computational approach.

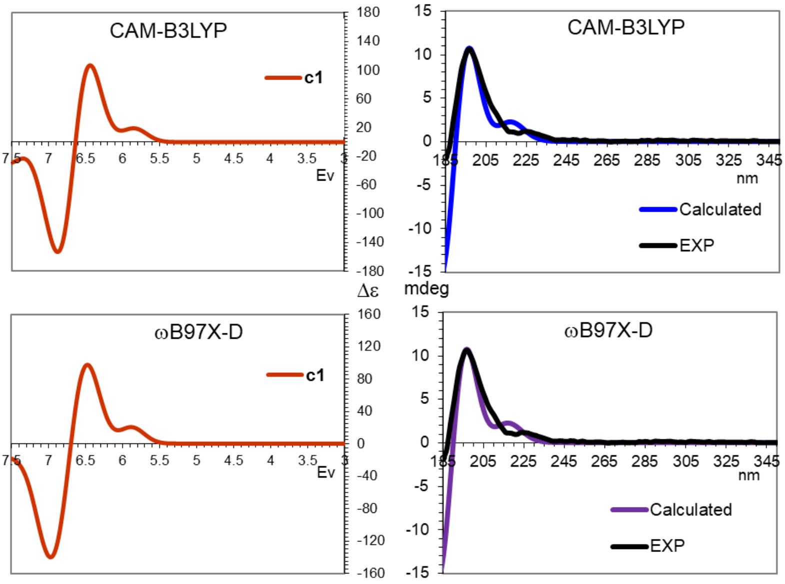

Figure S8. Left: TD-DFT simulation of the ECD spectrum of compound **6c** at the TD-DFT CAM-B3LYP/6-311++G(2d,p)//B3LYP-D3BJ/6-311G(d,p) level and the ωB97X-D/6-311++G(2d,p)//B3LYP-D3BJ/6-311G(d,p) level. Right: comparison between the Boltzmann-averaged simulated spectra and the experimental ones. The simulated spectra were both red-shifted by 0.16 eV and vertically scaled by 0.11 to match the experimental ones.

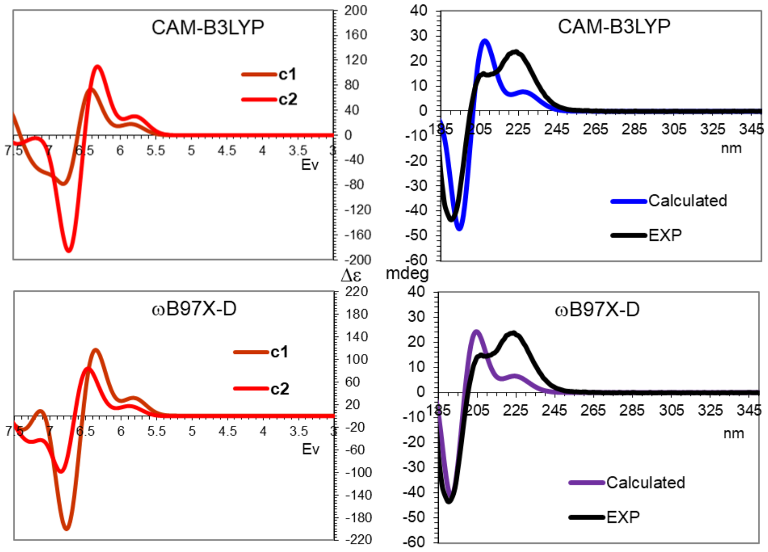

Figure S9. Left: TD-DFT simulation of the ECD spectrum of compound **2a** at the TD-DFT CAM-B3LYP/6-311++G(2d,p)//B3LYP-D3BJ/6-311G(d,p) level and ωB97X-D/6-311++G(2d,p)//B3LYP-D3BJ/6-311G(d,p). Right: comparison between the Boltzmann-averaged simulated spectra and the experimental one. The simulated spectra were both red-shifted by 0.33 eV and vertically scaled by 0.26 to match the experimental ones.

E.4 Relative Configuration Assignment of Compound 2l

The relative configuration of the three stereocenters of compound **2l** was assigned by NOE analysis, using the DPFGSE-NOE 1D sequence.[5] All the NMR spectra were recorded at -10°C to completely freeze any conformational rearrangement of the Boc moiety on valine. Indeed, an additional pattern of signals (8%) was observed at -10°C, due to the two populated Boc conformers (Figure S10). Bi-dimensional NMR spectra (gHSQC, gCOSY, gHMBC) allowed full assignment of all the carbons and hydrogens of **2l**, as shown in Table S9.

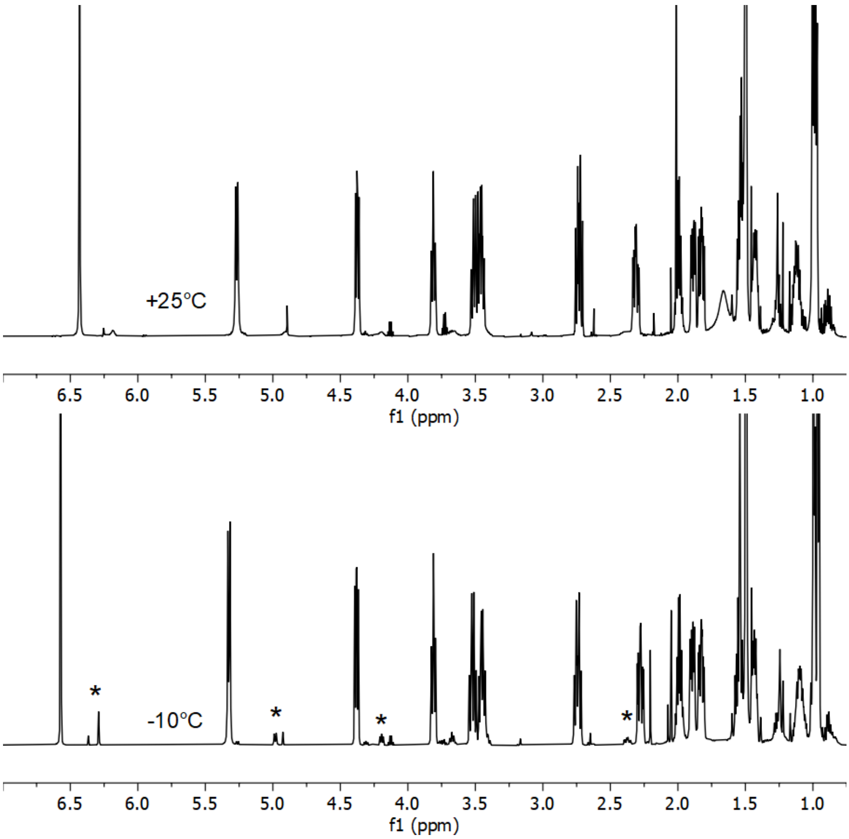

Figure S10. Top: ^{1}H NMR spectrum of **2l** at 600 MHz in $CDCl_3$ at +25°C. Bottom: ^{1}H NMR spectrum recorded at -10°C, showing a second set of signals due to a valine-Boc conformer.

Table S9. Atom numbering and full assignment of the NMR signals of **2l**

Label	^{13}C	^{1}H	notes
1	86.6	-	HMBC from *o*-Ph
2	29.9	2.74 and 2.27	COSY with 3.52 and 1.82
3	26.3	3.52 and 1.82	COSY with 2.74 and 2.27
4	74.22	-	HMBC from H2 and H3
5	21.5	1.89 and 1.53	HMBC with C4, COSY with H6’ and H6”
6	37.5	1.43 and 1.09	HMBC with C4,<br>COSY with H5’,H5”,H7’, H7”
7	49.6	3.81 and 3.45	HMBC with C4, COSY with H6’ and H6”
8	57.6	4.38	dd, J=9.4, 6.4
9	31.5	1.99	
10+11	17.6 + 19.5	0.95 and 0.97	
t-Bu (Me)	28.3	1.49	
t-Bu(C_q)	79.8	-	
o-Ph	126.5	7.47	d, 2H
m-Ph	127.8	7.35	t, 2H
p-Ph	127.4	7.28	t, 1H
CO (N)	175	-	HMBC from H8
CO (NH)	156	-	
OH	-	6.57	
NH	-	5.32	d, J=9.4 Hz with H8

J-coupling and NOE analysis:

From the ^{1}H NMR spectrum, *J* analysis provides useful information about the conformational preferences of the valine branch. The NH at 5.32 ppm is coupled with H8 with *J*=9.4 Hz. Such a large value implies a dihedral angle close to the *anti*-orientation of H8 and NH. On the other hand, H8 is coupled with H9 with *J*=6.4 Hz, a value typical of a *gauche* disposition. Both these observations help to define the conformation of the conformationally free valine branch.

Several NOE effects were obtained at -10°C by means of the DPFGSE-NOE sequence, using a mixing time of 0.75 s, corresponding to the average T1 measured at -10°C.

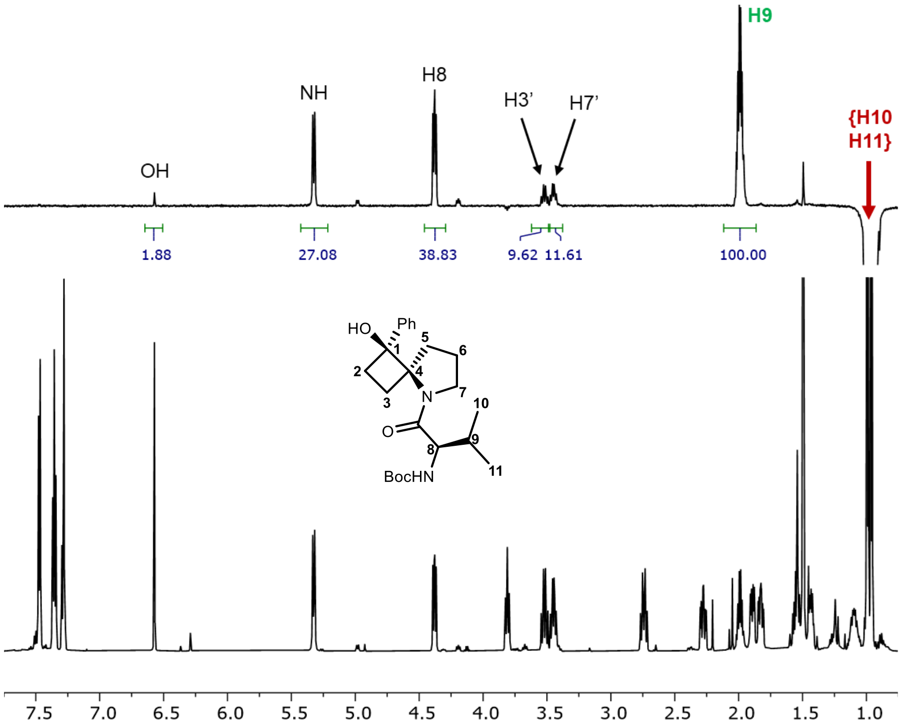

Figure S11. DPFGSE-NOE of compound **2l** (600 MHz in $CDCl_3$ at -10°C.). Bottom: ^{1}H control spectrum. Top: NOE spectrum with saturation of the two methyl groups of the isopropyl group, shown at 0.95-0.97 ppm (50 Hz-wide R-SNOB shaped pulse). Key integrations, relative to the NOE intensity of H_9, are reported.

On saturation of the two methyls of the isopropyl group, strong NOE effects were observed on H9, H8 and NH. They act as "control" NOEs to estimate the distance with the remaining hydrogens. Substantial NOE effects were observed on H3' and H7', and a weak effect was observed on the OH. The NOEs observed on H3' and H7' place the isopropyl group above the plane of the pyrrolidine, and on the same side of the CH_2 in position 3 on the cyclobutane.

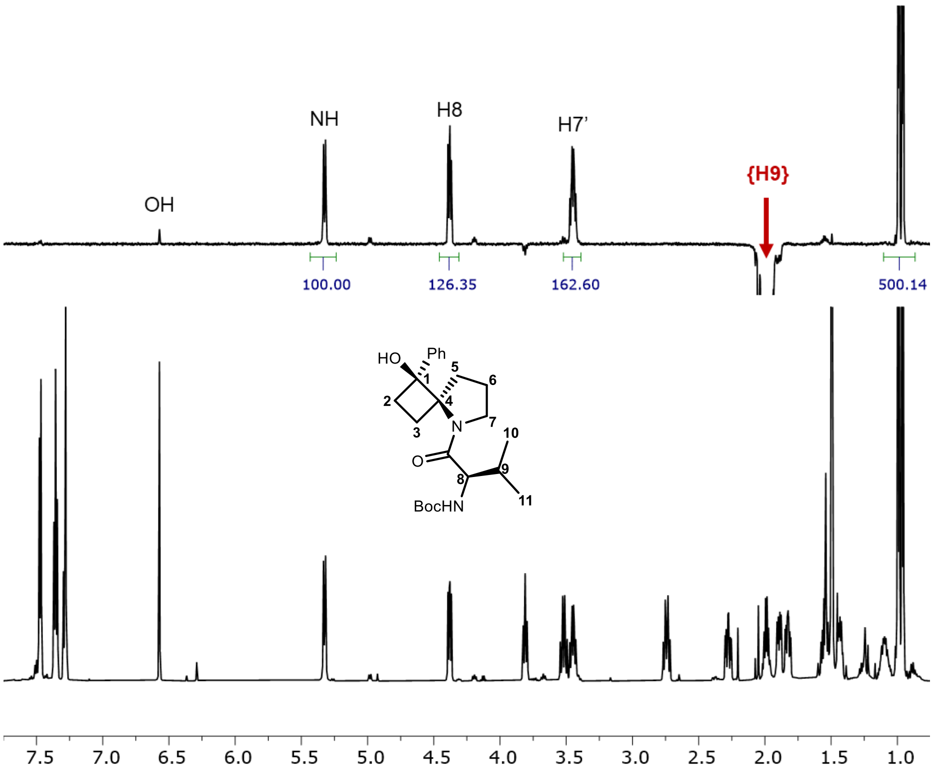

Figure S12. DPFGSE-NOE of compound **2l** (600 MHz in $CDCl_3$ at -10°C.). Bottom: ^1H control spectrum. Top: NOE spectrum with saturation of the H9 signal at 1.99 ppm (50 Hz-wide R-SNOB shaped pulse). Key integrations, relative to the NOE intensity of NH, are reported.

On saturation of H9, in addition to the strong control NOEs on the isopropyl methyl groups, NH and H8, a strong NOE was again observed on H7', but no effect was detected on H3'. The relative intensity of the NOEs on NH and H8 suggests that the H9-H8 and H-9 distances are very similar, while the absence of NOE on H3' places H8 in a position close to H7' and far from H3'.

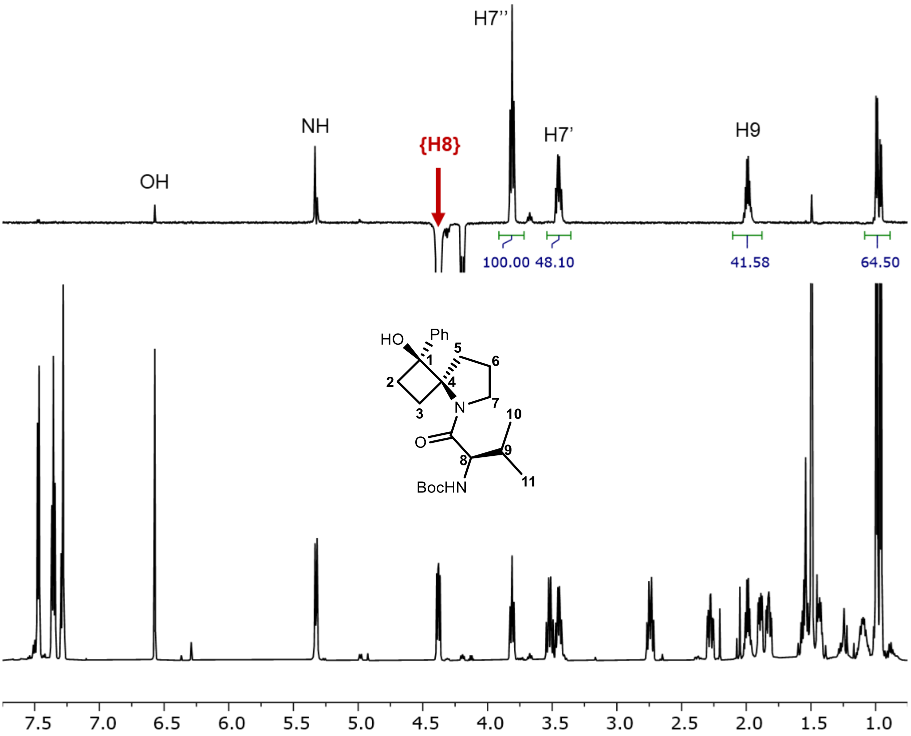

Figure S13. DPFGSE-NOE of compound **2l** (600 MHz in $CDCl_3$ at -10°C.). Bottom: ^1H control spectrum. Top: NOE spectrum with saturation of the H8 signal at 4.38 ppm (50 Hz-wide R-SNOB shaped pulse). Key integrations, relative to the NOE intensity of H7', are reported.

On saturation of H8, control NOEs were observed on the two methyls of the isopropyl group, with roughly the same intensity, and on H9. The latter is equivalent in intensity to that observed on H7', and *weaker* than the NOE on H7'' (i.e. on the opposite site of the pyrrolidine ring with respect to H7'). This means that H8 must be closer to H7'', while the H8-H7' and H8-H9 distances must be very similar. These constraints impose an anti-orientation of H8 with respect to the amidic oxygen (i.e., the one engaged with the OH group of the cyclobutane. In the optimized structure (see below), the H8-H7" distance is 2.16 Å, while the H8-H7' is 2.36 Å, yielding a theoretical NOE ratio of 1.75, vs the observed ratio of 2.08. In the optimized structure, the H8-H9 distance is 2.42 Å, again in agreement with the observed NOE on H9 vs the NOE observed on H8.

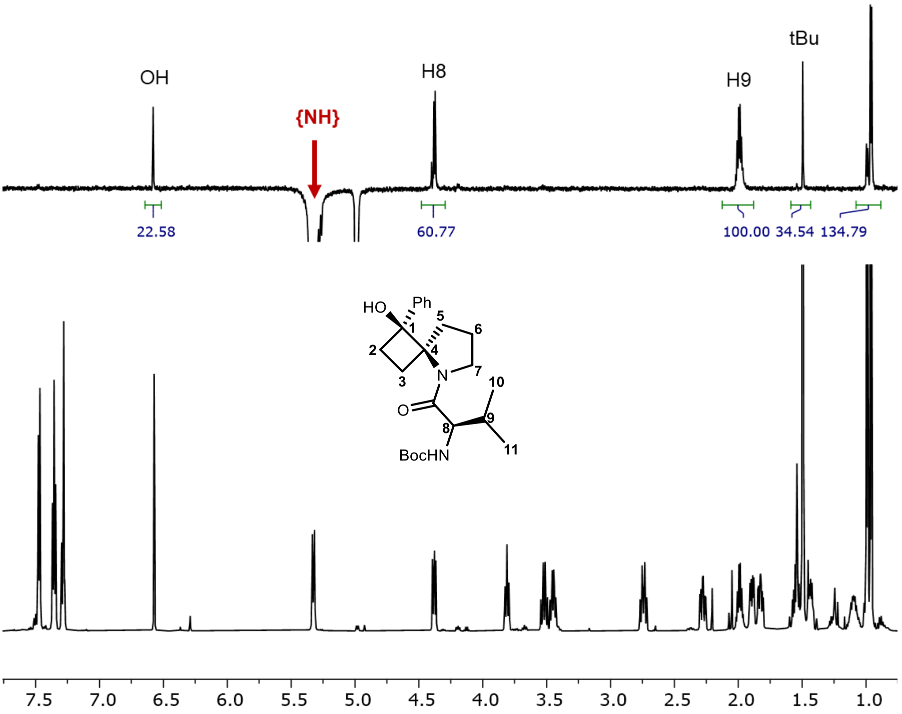

Figure S14. DPFGSE-NOE of compound **2l** (600 MHz in $CDCl_3$ at -10°C.). Bottom: ^{1}H control spectrum. Top: NOE spectrum with saturation of the NH signal at 5.32 ppm (50 Hz-wide R-SNOB shaped pulse). Key integrations, relative to the NOE intensity of H9, are reported.

As expected, on saturation of the NH, control NOEs were observed on H8 and H9. However, as a key indication of the conformational preferences of the isopropyl group, the NOE effect on the two methyls are quite different, the ratio being approximately 4:1. This implies that in the preferred conformation, only one methyl is close to the NH. Because of the *anti* conformation of the carbamate, a very weak NOE is observed also on the carbonyl group.

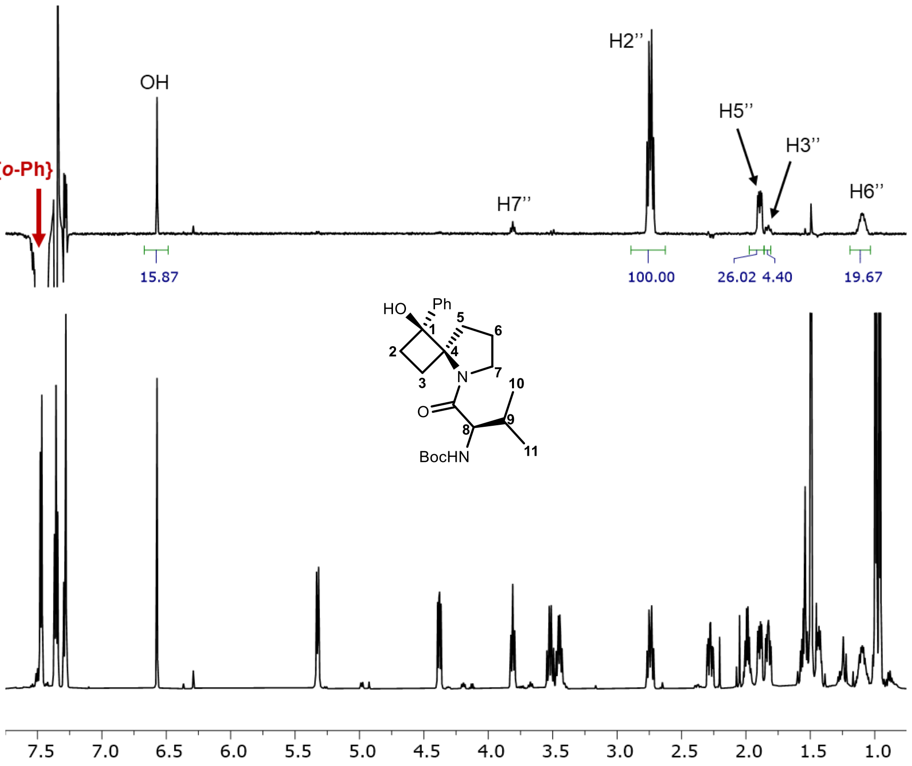

Figure S15. DPFGSE-NOE of compound **2l** (600 MHz in $CDCl_3$ at -10°C.). Bottom: ^1H control spectrum. Top: NOE spectrum with saturation of the *ortho* hydrogens of the phenyl ring at 7.48 ppm (50 Hz-wide R-SNOB shaped pulse). Key integrations, relative to the NOE intensity of H2', are reported.

When both the ortho-hydrogens of the phenyl were saturated very strong NOEs were observed on H2'' H3", and H5'. The first two NOEs allow unambiguous assignment of the hydrogens that lie on the same side of the cyclobutane ring with respect to the phenyl, and confirm the NOE observed on H3' in Figure S11. No NOE effects were observed in any part of the valine signals, thus confirming that the phenyl is on the opposite side of the cyclobutane with respect to the valine moiety. NOE were also observed on the pyrrolidine hydrogens H5" and H6", on the same side of the pyrrolidine with respect to H7" (see NOE in Figure S14).

Key findings by NOE can be summarized as follows:

1) NOEs clearly show that H8 must be close to H7' and H7". This implies an *anti*-arrangement with the amidic carbonyl.
2) The isopropyl moiety must be within the NOE range with respect to H7' on the pyrrolidine and H3' on the cyclobutanol.
3) The NH of protected valine is *anti* to H8 because of the very large J-coupling.

Taken together, these NOE and J-coupling constraints are consistent only with the **S,S,S** diastereoisomer of **2l**. In particular, the observed NOE contacts of the valine isopropyl group with H7' and H3', together with the *anti* relationship between NH and H8 inferred from *J*-coupling analysis, are satisfied only when the cyclobutanol moiety has *S,S* configuration and the valine residue has *S* configuration. The alternative diastereoisomer, shown in Figure S16 for comparison, does not reproduce the observed spatial proximities.

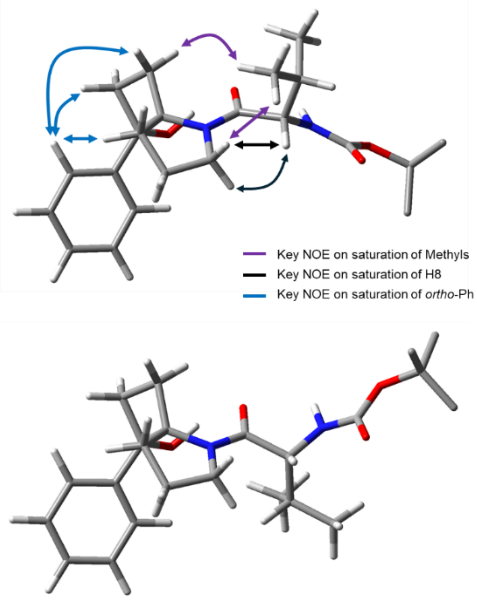

Figure S16. Optimized structures for two diastereoisomers of **2l** (B3LYP/6-31G(d,p) level of theory). Top: the *S,S,S* diastereoisomer, with the relevant experimental NOE constraints shown as arrows. Bottom: the *R*-valine, *S,S* diastereoisomer shown for comparison (negative control).

F. Mechanistic Studies

In all these studies, product partitioning ratios (**2a**:**3**) were determined by ^{1}H NMR analysis of the crude reaction mixtures; under the standard reaction conditions, **3'** was not detected and was therefore not included in the quantitative analysis, likely owing to instability under the reaction conditions.

F.1 Effect of Triplet Quenchers

To investigate the involvement of triplet excited states in the reaction mechanism and their influence on product partitioning and stereochemical fidelity, the effect of triplet quenchers as additives was examined.

Table S10. Effect of triplet quenchers on the efficiency of the reaction and on product selectivity and stereospecificity.

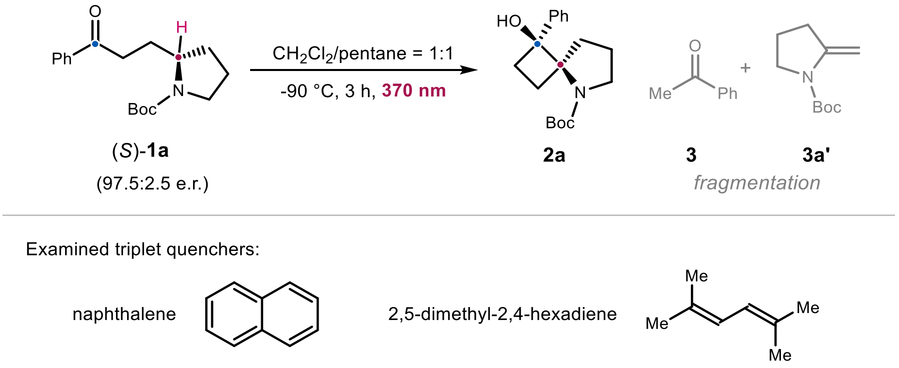

Entry	Condition	Yield **2a** (%)	e.r. **2a**	% e.s.	Product ratio **2a**:**3**
1	No additive	86	95:5	95	>20:1
2	Naphthalene (0.5 equiv.)	65	95:5	95	>20:1
3	Naphthalene (5.0 equiv.)	43	95:5	95	>20:1
4	2,5-Dimethyl-2,4-hexadiene (5.0 equiv.)	42	95:5	95	>20:1

The reaction mixtures were prepared in a nitrogen-filled glovebox using anhydrous and degassed solvents. An 8 mL oven-dried vial equipped with a magnetic stir bar was charged with ketone substrate (*S*)-**1a** (5.0 mg, 0.017 mmol, 97.5:2.5 e.r.), dichloromethane (165 µL),

pentane (165 μL), and the indicated additive (0.5 or 5.0 equiv.). The vial was sealed with an airtight screw cap and removed from the glovebox. The reaction vessel was then placed in a EtOH/liquid nitrogen cooling bath maintained at −90 °C and irradiated with stirring (450 rpm) using a Kessil lamp (λ = 370 nm) for 90 min reaction time. The reaction temperature was continuously monitored throughout the irradiation period and maintained at −90 °C by addition of liquid nitrogen as required. Upon completion, the lamp was turned off and the reaction mixture was allowed to warm to room temperature. The solvent was carefully removed under reduced pressure at room temperature. The crude reaction mixture was analyzed by ^{1}H NMR spectroscopy to determine conversion and product distribution. The singlet corresponding to the methyl group of **3** (δ 2.60 ppm) was used to quantify fragmentation, while the signal at δ 2.67 ppm corresponding to product **2a** was used to determine product formation. Compound **3a′** was not detected under the photochemical conditions, likely due to competing degradation pathways. The enantiomeric excess was determined by chiral SFC analysis.

The reduced conversion observed in the presence of triplet quenchers supports the involvement of triplet excited states in the productive pathway (Table S10). The unchanged product partitioning and high stereochemical fidelity suggest that the selectivity-determining step is established after triplet excitation, within the reactive triplet-state manifold.

F.2 Non-linear Effect Study

To investigate whether the stereochemical outcome of the reaction arises from intermolecular interactions between substrate molecules, a nonlinear effect (NLE) study was performed. In such experiments, deviations from a linear correlation between the enantiomeric excess of the substrate and that of the product may indicate the involvement of higher-order or aggregated species in the stereodetermining step.

Table S11. Effect of the enantiomeric excess of substrate **1a** on the enantiomeric excess of the product **2a**.

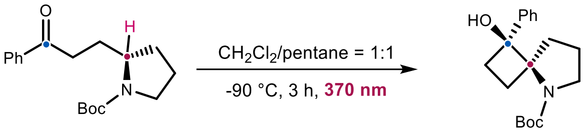

Entry	% e.e. Substrate **1a**	% e.e. Product **2a**
1	8	8
2	20	20
3	32	31
4	43	43
5	58	56
6	68	66
7	84	80
8	95	90

The reaction mixtures were prepared in a nitrogen-filled glovebox using anhydrous and degassed solvents. Stock solutions of racemic ketone substrate **1a** and substrate (*S*)-**1a** with 97.5:2.5 e.r. were first prepared (50 mM) in dichloromethane/pentane (1:1, v/v). The reaction solutions with varying % e.e. of substrate **1a** were then obtained by combining appropriate portions of these stock solutions to a total volume of 330 µL. The enantiomeric excess of **1a** of each resulting solution was quantified prior to the reaction by chiral SFC analysis.

An 8 mL oven-dried vial equipped with a magnetic stir bar was charged with the prepared solution with defined enantiomeric excess of **1a**, sealed with an airtight screw cap, and removed from the glovebox. The reaction vessel was placed in an EtOH/liquid nitrogen cooling bath maintained at −90 °C and irradiated with stirring (450 rpm) using a Kessil lamp (λ = 370 nm) for 90 min. The reaction temperature was continuously monitored and maintained at −90 °C by addition of liquid nitrogen as required. Upon completion, the lamp

was turned off and the reaction mixture was allowed to warm to room temperature. The solvent was carefully removed under reduced pressure at room temperature. The enantiomeric excess of the product **2a** was determined by chiral SFC analysis.

A linear correlation was observed between the enantiomeric excess of the substrate **1a** and that of the product **2a** (Figure S17), indicating that the stereochemical outcome of the reaction arises from a single-molecule process rather than intermolecular interactions or aggregation. This observation supports the view that the stereodetermining step involves only one substrate molecule and that stereochemical information is transferred intramolecularly during the photochemical step.

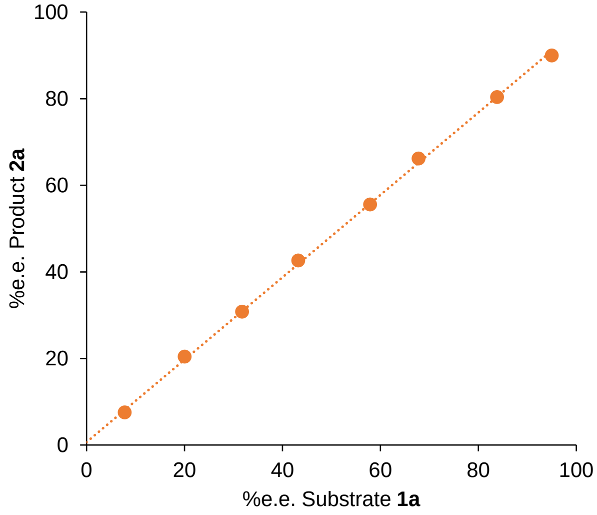

Figure S17. Linear relationship of the enantiomeric excess of substrate **1a** and the enantiomeric excess of the product **2a** formed in the reaction.

F.3 Effect of Substrate Concentration

To examine whether a potential intermolecular process contributes to the stereospecificity of the reaction, the reaction was conducted at different substrate concentrations and the stereochemical outcome was assessed.

Table S12. Effect of substrate concentration on the efficiency of the reaction as well as on product selectivity and enantiospecificity.

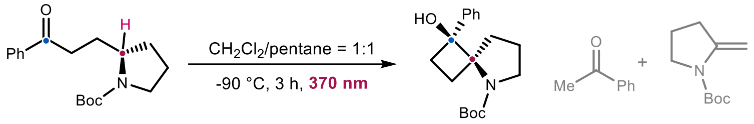

(*S*)-**1a** (97.5:2.5 e.r.) → **2a** + **3** + **3a'** (*fragmentation*)

Entry	Substrate concentration (mM)	Yield **2a** (%)	e.r. **2a**	% e.s.	Product ratio **2a**:**3**
**1**	50	86	95:5	95	>20:1
**2**	17	88	94:6	93	>20:1
**3**	10	87	94:6	93	>20:1

The reaction mixtures were prepared in a nitrogen-filled glovebox using anhydrous and degassed solvents. An 8 mL oven-dried vial equipped with a magnetic stir bar was charged with ketone substrate **1a** (5.0 mg, 0.017 mmol, 97.5:2.5 e.r.), and the volumes of dichloromethane and pentane were adjusted to achieve the desired substrate concentration. The vial was sealed with an airtight screw cap and removed from the glovebox. The reaction vessel was placed in an EtOH/liquid nitrogen cooling bath maintained at −90 °C and irradiated with stirring (450 rpm) using a Kessil lamp (λ = 370 nm) for 90 min. The reaction temperature was continuously monitored throughout the irradiation period and maintained at −90 °C by addition of liquid nitrogen as required. Upon completion, the lamp was turned off and the reaction mixture was allowed to warm to room temperature. The solvent was carefully removed under reduced pressure at room temperature. The crude reaction mixture was analyzed by ^{1}H NMR spectroscopy to determine conversion and product distribution. The singlet corresponding to the methyl group of compound **3** (δ 2.60 ppm) was used to quantify fragmentation, while the signal at δ 2.67 ppm corresponding to product **2a** was used to determine product formation. Compound **3′** was not detected under the photochemical conditions, likely due to competing degradation pathways. The enantiomeric excess was determined by chiral SFC analysis.

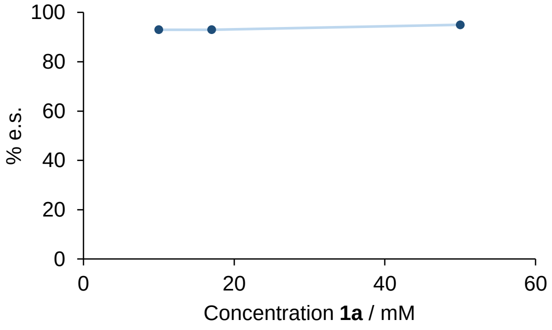

Figure S18. Effect of substrate concentration on enantiospecificity.

No significant dependence of enantiospecificity, product ratio, or reaction efficiency on substrate concentration was observed (Figure S18). This result is consistent with intermolecular interactions between substrate molecules not playing a significant role in the stereodetermining step and supports a mechanism in which the stereochemical outcome is governed by an intramolecular process.

F.4 Effect of Hydrogen-Bond Acceptors

To investigate the influence of hydrogen-bonding interactions on product partitioning, hydrogen-bond acceptor additives (10 equiv.) were evaluated.

Table S13. Effect of hydrogen-bond acceptor additives on product partitioning.[13]

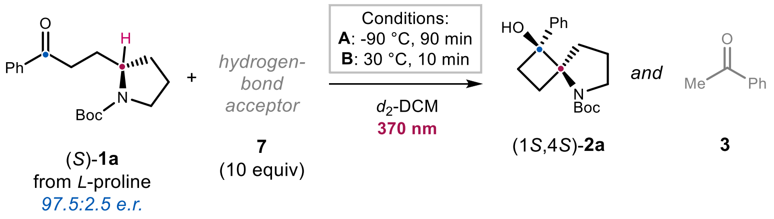

Investigated hydrogen-bond acceptors **7**:

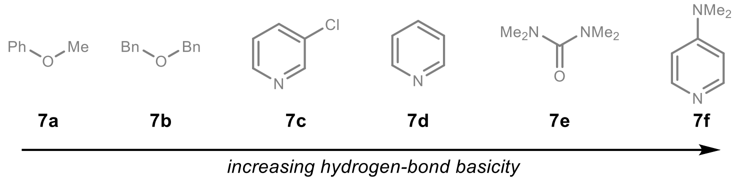

			Condition **A**			Condition **B**
Entry	Additive	β_2^{H}[40]	Product ratio **2a**:**3**	e.r. **2a**	e.s. %	Product ratio **2a**:**3**
1	No Additive	-	13.04	95.0:5.0	95%	2.28
2	**7a**	0.260	12.50	95.0:5.0	95%	1.80
3	**7b**	0.388	10.50	95.5:4.5	96%	1.69
4	**7c**	0.488	9.67	96.0:4.0	97%	1.26
5	**7d**	0.625	8.33	96.5:3.5	97%	0.88
6	**7e**	0.743	7.14	95.5:4.5	96%	0.76
7	**7f**	0.859	5.36	96.0:4.0	97%	0.43

An 8 mL oven-dried vial equipped with a magnetic stir bar was charged with ketone substrate **1a** (5.0 mg, 0.017 mmol, 97.5:2.5 e.r.) in deuterated dichloromethane (250 µL) and hydrogen-bond acceptor additive **7** (10 equiv). The vial was sealed with an airtight screw cap and irradiated with stirring (450 rpm) using a Kessil lamp (λ = 370 nm) under the conditions specified below. Reaction temperatures were maintained and monitored throughout the irradiation period. Upon completion, the lamp was turned off and the reaction mixture was allowed to warm to room temperature. An aliquot of the reaction mixture was removed and diluted with $CDCl_3$ for analysis by ^1H NMR spectroscopy to determine the product ratio. The singlet corresponding to the methyl group of **3** (δ 2.60 ppm) was used to quantify fragmentation, while the signal at δ 2.67 ppm corresponding to product **2a** was used to determine product formation. Compound **3′** was not detected under the photochemical conditions, likely due to competing degradation pathways.

Condition A: The sealed reaction vessel was placed in an EtOH/liquid nitrogen cooling bath maintained at −90 °C and irradiated for 90 min.

Condition B: The sealed reaction vessel was maintained at 30 °C and irradiated for 10 min.

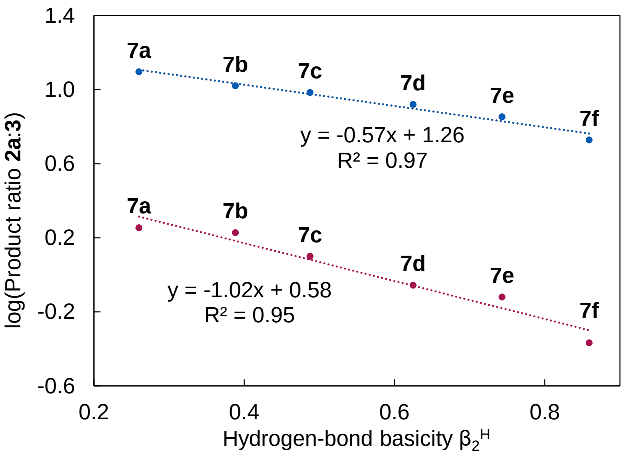

Figure S19. Linear relationship of β_2^H of hydrogen-bond acceptor additives on the product partitioning **2a**:**3** in the reaction.

Logarithmic plotting of the product ratios (**2a**:**3**) revealed a linear correlation with increasing hydrogen-bond basicity parameter β_2^H of the additives **7** (Figure S19). This result is consistent with hydrogen-bonding interactions systematically influencing product partitioning, likely through differential stabilization of the competing pathways.

F.5 Effect of Hydrogen-Bond Donors

To probe the effect of external hydrogen-bond donors on type selectivity and enantiospecificity, methanol was added in increasing volume fractions under the optimized reaction conditions. The methanol volume fraction is defined as $\varphi_{MeOH} = V_{MeOH}/V_{total}$, with $V_{total} = 1.0$ mL.

The reaction mixtures were prepared using anhydrous and degassed solvents. An 8 mL oven-dried vial equipped with a magnetic stir bar was charged with ketone substrate (*S*)-**1a** (5.0 mg, 0.017 mmol, 95% e.e.) and the appropriate solvent mixture. For $\varphi_{MeOH} = 0$, CH_2Cl_2/pentane was used as a 1:1 mixture, with a total solvent volume of 1.0 mL. For $\varphi_{MeOH} = 0.25$, methanol (0.25 mL) was added and the remaining solvent volume (0.75 mL) consisted of CH_2Cl_2/pentane in a 1:1 ratio. For $\varphi_{MeOH} = 1.0$, methanol was used as the sole solvent. The vial was sealed with an airtight screw cap fitted with a septum and removed from the glovebox. When required, degassed methanol was added via syringe through the septum. The reaction vessel was placed in a liquid nitrogen/EtOH cooling bath maintained at −90 °C and irradiated with stirring (450 rpm) using a Kessil lamp (λ_{max} = 370 nm) for 90 min. The reaction temperature was continuously monitored and maintained at −90 °C by addition of liquid nitrogen as required. Upon completion, the lamp was turned off and the reaction mixture was allowed to warm to room temperature. The solvent was removed under reduced pressure at room temperature. The crude mixture was analyzed by ^{1}H NMR spectroscopy to determine conversion and product ratio. The enantiomeric excess of product **2a** was determined by chiral SFC analysis.

Table S14. Effect of methanol on yield, enantiospecificity, and type selectivity.

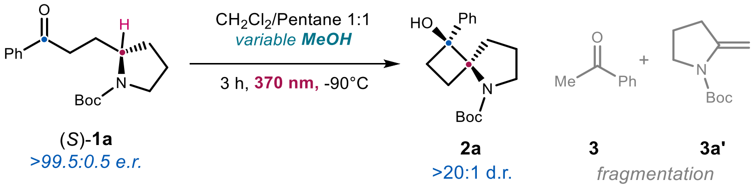

**Entry**	MeOH volume fraction $\varphi_{MeOH} = V_{MeOH}/V_{total}$	Yield **2a** (%)	e.e. **2a** (%)	e.s. (%)	Product ratio **2a**:**3**
**1**	0	86	90	95	>20:1
**2**	0.25	56	85	89	5.9:1
**3**	1.0	29	83	87	0.7:1

Total solvent volume = 1.0 mL. For entries containing CH_2Cl_2/pentane, CH_2Cl_2:pentane = 1:1.

F.6 Effect of the Reaction Medium

To investigate the influence of the reaction medium on product partitioning and enantiospecificity, different solvent mixtures were evaluated. The reactions were performed according to General Procedure C, varying the solvent mixture and temperature.

Table S15. Effect of the reaction medium on the stereoretentive Norrish–Yang photocyclization of **1a**.

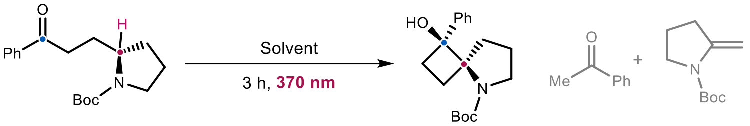

**Entry**	Solvent	T °C	Yield **2a** (%)	e.s. (%)	Product ratio **2a**:**3**
**1**	CH_2Cl_2/pentane 1:1	-90	86	95	>20:1
**2**	MeCN/pentane 1:1	-90	63	93	3.5:1
**3**	$CHCl_3$/pentane 1:1	-90	78	94	10:1
**4**	Frozen benzene	0	72	21	3:1

F.7 Computational Studies

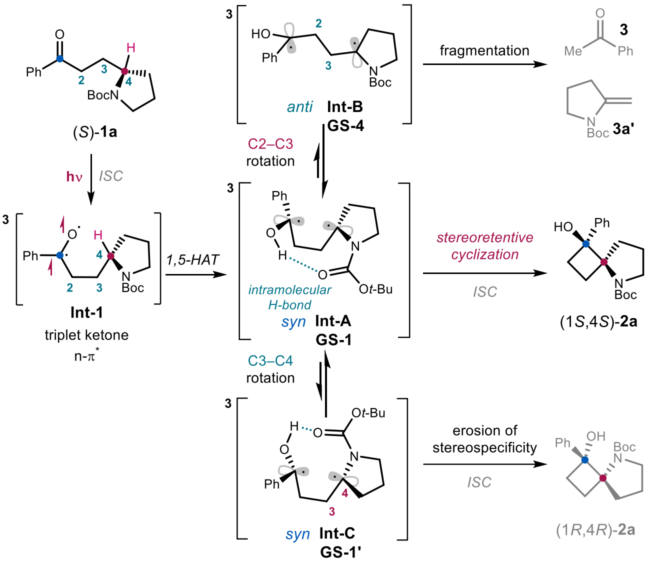

Figure S20. Simplified conformational pathways considered in the computational analysis of the triplet 1,4-diradical manifold derived from (*S*)-**1a**.

It is well established that, for aryl ketone substrates, the Yang photocyclization and/or Norrish type-II fragmentation occur after formation of a triplet 1,4-diradical intermediate **Int-A** generated by 1,5-HAT from the initial triplet ketone **Int-1**. The lifetime of such triplet 1,4-diradicals before intersystem crossing is typically 50–200 ns.[14,15] Under conditions of restricted conformational mobility, such as in viscous media or rigid systems, this timescale can extend into the microsecond range.[16]

A DFT investigation was carried out to analyze the available conformational pathways to achieve either stereoretentive cyclization, or non-stereospecific cyclization/fragmentation. Starting from the optimized ground-state geometry (**GS**) of the spirocyclobutanol **2a**, the 1,4-diradical starting guess geometry was generated by elongation of the C1-C4 bond to 2.0Å, followed by optimization in the triplet state with DFT at the UB3LYP-GD3BJ/6-311G(d,p) level of theory,[17,18] including the solvent dichloromethane by means of the IEF-PCM model[19] (hereafter indicated as IEFPCM[DCM]). Frequency analysis confirmed that the resulting geometry (**GS-1**) was indeed a ground state (no imaginary frequency), and the wavefunction was successfully checked for stability (Gaussian 16 keyword "stable"). The geometry of **GS-1** is strongly influenced by the intramolecular hydrogen bonding (1.81 Å), which keeps the Boc moiety fixed in a single conformation (confirmed by NMR, see Section E.2)

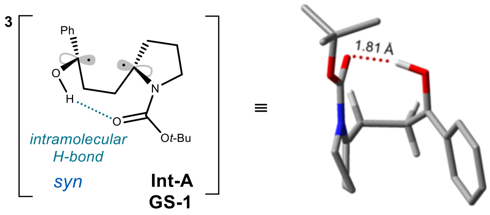

Figure S21. DFT optimized structure of the 1,4 triplet diradical after the HAT process. Optimization at the IEF-PCM[DCM]-UB3LYP-GD3BJ/6-311G(d,p) level of theory.

C2-C3 rotation pathway

Starting from **GS-1** geometry, the C2-C3 rotation was modelled by means of a relaxed rotational scan driven on the C1-C2-C3-C4 dihedral, with geometry optimization at each step (Figure S22), at the IEFPCM[DCM]-UB3LYP-GD3BJ/6-311G(d,p) level of theory. Guess geometries for stationary points (**GS** and **TS**) were extracted at the minima and maxima of the scan curve, and full optimizations were performed at the IEFPCM[DCM]-UB3LYP-GD3BJ/6-311G(d,p) level.

Frequency analysis confirmed that the optimized geometries were ground states **GS-2**, **GS-3**, **GS-4**, and **GS-5** (no imaginary frequencies) and transition states (one imaginary frequency; **TS-1**, **TS-2**, **TS-3**, and **TS-4**). The free Gibbs energy was evaluated with Goodvibes software[20] at 183 K, 1 atm, and 1M concentration by using Grimme's qRRHO approximation[21] with a threshold of 100 cm^{-1}. A summary of key geometric parameters and relative energies is reported in Table S16. The optimized ground-state structures are reported in Figure S23.

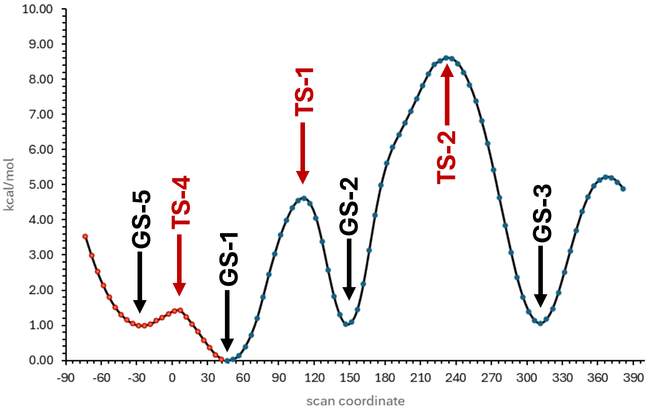

Figure S22. Rotational scan around C2-C3, starting from **GS-1** by 70 increments of +5° (blue dots) and by 25 increments of -5° (red dots). For each point the C1-C2-C3-C4 dihedral was frozen and the optimizations were run at the IEFPCM[DCM]-UB3LYP-GD3BJ/6-311G(d,p) level of theory. NOTE: for a better visualization of the pathway, the scan coordinate is presented from left to right starting from the geometry of the enantiomer of **GS-1** (thus C1-C2-C3-C4 dihedral = +46.6°), and by exceeding the 180° of the dihedral definition. Table S16 reports the correct dihedral values of the optimized stationary points.

Table S16. Summary of geometric parameters and relative energies for C2-C3 rotation. Geometries optimized at the IEFPCM[DCM]-UB3LYP-GD3BJ/6-311G(d,p) level.

	C2-C3 dihedral	H-bond dist. Å	H° a.u.	ΔH° kcal/mol	qh-G(T) (a.u.)[a]	ΔG(T) kcal/mol	Boltz. 183K	Boltz. 298K
**GS-1**	-46.6	1.81	-981.307100	**0.00**	-981.342046	**0.00**	0.762	0.580
**TS-1**	-110.6	1.66	-981.300575	4.09	-981.335297	4.23		
**GS-2**	-148.3	1.73	-981.305432	1.05	-981.341003	0.65	0.128	0.190
**TS-2**	127.1	4.6	-981.294507	7.90	-981.329706	7.74		
**GS-3**	49.1	5.49	-981.305358	1.09	-981.340489	0.98	0.051	0.110
**TS-3**[b]	-179.4	4.23	-981.294827	7.70	-981.33016	7.46		
**GS-4**	176.4	5.54	-981.298895	5.15	-981.335117	4.35	0.000	0.000
**TS-4**[c]	4.7	1.76	-981.305403	1.06	-981.339695	1.48		
**GS-5**[c,d]	26.5	1.76	-981.305379	1.08	-981.340568	0.93	0.059	0.120

[a] ZPE-corrected Free Gibbs Energies were evaluated at 183K (-90°C), 1 atm and 1M concentration with Grimme's qRRHO approximation and frequency threshold set to 100 cm^{-1}. [b]**TS-3** connects **GS-2** to **GS-4** by rotation of the C3-C2-C1-O dihedral. [c] From **GS-1** by clockwise rotation. [d] **GS-5** is connected to **GS-3** by 180° rotation of the C1-C2 bond.

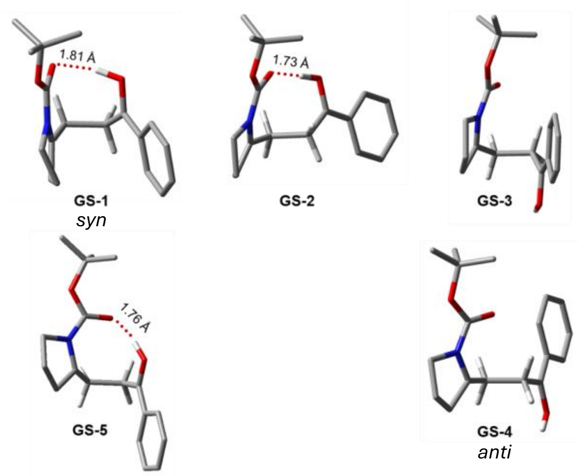

Figure S23. DFT optimized structures of **GS-1**, **GS-2**, **GS-3**, **GS-4**, and **GS-5**. Optimizations at the IEF-PCM[DCM]-UB3LYP-GD3BJ/6-311G(d,p) level of theory. All the hydrogens, except the OH and those on C2-C3, were omitted for clarity.

The first transition state **TS-1** connects **GS-1** to **GS-2**, with a C2-C3 dihedral of -110.6° and an energy barrier of 4.23 kcal/mol energy. **GS-2** and **GS-3** are then connected by a transition state **TS-2** with activation energy higher than 7.5 kcal/mol (127.1° dihedral), with a conformational half-life time ≈ 200 μs (≈ 4000 s^{-1} rate constant) at 183K. **GS-3** can be thus considered inaccessible within the intersystem crossing timescale (τ_{ISC} ≈ 50-200 ns).

From **GS-2**, the *anti*-conformation **GS-4** can be accessed by rotation around the C3-C2-C1-O dihedral angle. During this process, the H-bond is lost and the **GS-4** conformation is indeed an *anti*-disposition, with C1-C2-C3-C4 dihedral angle of 176°. The energy of **TS-3** connecting

GS-2 and **GS-4** (*anti*-periplanar, ϕ = 176 °) is again higher than 7.0 kcal/mol. At 183K, the rate constant for this energy barrier is ≈ 4000 s^{-1}, corresponding to a half-life time of ≈ 200 μs, while at 298K the values are $1.5 \cdot 10^7$ s^{-1} and ≈ 40-50 ns, respectively. Lacking the H-bond stabilization contribution, **GS-4** is quite high in energy, so its population should be negligible. Therefore, at 183K, the long lifetime of **GS-1** and the high energies of **TS-3** and **GS-4** strongly bias the reaction pathway toward Yang photocyclization.

However, it is worth noting that the intermediate **GS-2** is accessible *via* **TS-1** even at -90°C, given the low $\Delta G^{\neq}$ value (Table S16): a transition state of ≈ 4.2 kcal/mol implies a rate constant ≈ $3 \cdot 10^7$ s^{-1} at 183 K (-90 °C), corresponding to a half-life time of ≈ 20-30 ns, whereas at 298K the same rotational rate constant is ≈ $5 \cdot 10^9$ s^{-1}, corresponding to a lifetime in the 100 ps scale. **GS-2** has a dihedral angle of -148° (anticlinal[22]) and could still allow for Norrish Type-II cleavage. This alternative pathway could explain why in *d_2-DCM* used for mechanistic investigation (see Figure 2 in the main text) we observed trace amounts of fragmentation products even at -90 °C (13.5:1, in favor of Yang cyclization). The dipole moments calculated for **GS-1** and **GS-2** are very different (4.04 and 8.29 D, respectively), and dichloromethane is known to largely increase its polarity on lowering the temperature,[23] reaching values up to 15 at -80°C. Thus, in pure dichloromethane, **GS-2** could contribute to some fragmentation. Under the optimized reaction conditions, in which a lower-polarity mixture is used (pentane/CH_2Cl_2), this fragmentation pathway is less efficient, (i.e., > 20:1 in favor of Yang cyclization, see Figure 2 main text). DFT optimization of **GS-1** and **GS-2** including pentane instead of DCM, showed that the energy difference rises from 0.65 to 1.17 kcal/mol, thus implying a 4% population of **GS-2** at 183 K in pentane.

From **GS-1**, by driving the dihedral in the opposite direction, an alternative pathway leads to a different ground state through a transition state where the C4-C3-C2-C1 dihedral is ≈ 0° (**TS-4** in Figure S22). In the optimized geometry of **TS-4** the H-bond is still effective, and the activation energy is therefore very low ($\Delta G^{\neq}$ =1.48 kcal/mol). When the ground state structure corresponding to C4-C3-C2-C1 = 26° (**GS-5**) was optimized, its energy was found to be 0.93 kcal/mol higher than that of **GS-1** (note: **GS-5** geometry is different with respect to **GS-3** because of the different C1-C2-C3-C4 dihedral; **GS-5** converts into **GS-3** by 180° rotation around C1-C2 bond). However, **GS-5** also biases the reaction toward Yang cyclization.

C3-C4 rotation pathway

In the case of C3-C4 rotation, the rotational pathway allows conversion of the syn-**GS-1** conformation into a syn-enantiomeric conformation (**GS-1'**) by 180° rotation of the N-C4-C3-C2 dihedral. In principle, this interconversion can happen by two TSs in which the CH_2 on C3 crosses the pyrrolidine plane close to the CH_2 (**TS-5**) or to the nitrogen of the pyrrolidine ring (**TS-6**). Again, the rotational pathway was generated by a relaxed scan around the N-C4-C3-C2 dihedral angle, and the guess geometries for stationary points were extracted from the energy minima and maxima. Table S17 reports the geometrical parameters and relative energies of the fully optimized geometries; Figure S24 reports the optimized 3D structures of the TSs.

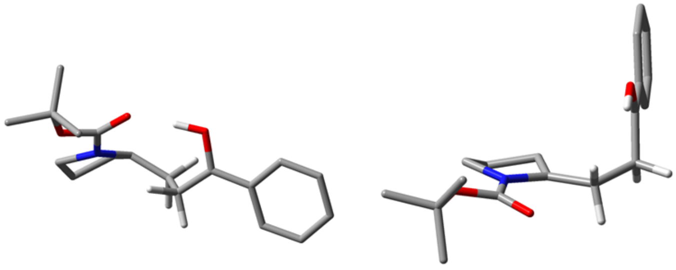

Figure S24. DFT optimized structure of the racemization transition states **TS-5** and **TS-6**. Optimization at the IEF-PCM[DCM]-UB3LYP-GD3BJ/6-311G(d,p) level of theory. All the hydrogens, except the OH and those on C2-C3 were omitted for clarity.

Table S17. Summary of geometric parameters and relative energies for C3-C4 rotation. Geometries optimized at the IEFPCM[DCM]-UB3LYP-GD3BJ/6-311G(d,p) level.

	N-C4-C3-C2 dihedral.	H-bond dist. Å	H° a.u.	ΔH° kcal/mol	qh-G(T) a.u.[a]	ΔG(T) kcal/mol	Boltz. 183K
**GS-1**	86.9	1.81	-981.3071	0.00	-981.342046	0.00	0.967
**TS-5**	-14.9	1.66	-981.296639	6.56	-981.331510	6.61	
**GS-1'**	-90.6	1.78	-981.304559	1.59	-981.340087	1.23[b]	0.033
**TS-6**	-160.8	4.24	-981.295707	7.15	-981.331054	6.90	

[a] ZPE-corrected Free Gibbs Energies were evaluated at 183K, 1 atm and 1M concentration with Grimme's qRRHO and threshold set to 100 cm^{-1}. [b] The energy of **GS-1'** is not exactly identical to that of **GS-1** because of a different conformation of the pyrrolidine ring. Fast ring inversion drives this conformation to the enantiomer of **GS-1**.

In both **TS-5** and **TS-6**, the activation energy is higher than 6.6 kcal/mol, a value that implies a half-life time of ≈10-20 μs at 183 K. Thus, also in this case, at low temperature the conformational rearrangement is slow with respect to the lifetime of the triplet diradical (τ_{ISC}

≈ 50-200 ns). By contrast, at 298 K, the half-life time of **GS-1** becomes ≈10 ns, and the conformational rearrangement is therefore fast on the timescale of the triplet-diradical lifetime, consistent with the experimentally observed complete loss of stereochemical fidelity at room temperature. At the intermediate temperature of 233 K (-40 °C; see Figure 2, main text), the half-life time of conformer interconversion (≈ 200 ns considering a 6.6 kcal/mol barrier) becomes comparable to that of the triplet diradical, in line with the experimentally observed partial stereoretention (77:23 e.r., 55% e.s.). At 183 K (-90 °C), by contrast, conformational rearrangement is much slower than triplet decay, consistent with the high stereochemical fidelity observed experimentally (97.5:2.5 e.r., 96% e.s.). Figure S25 summarizes the whole conformational pathway.

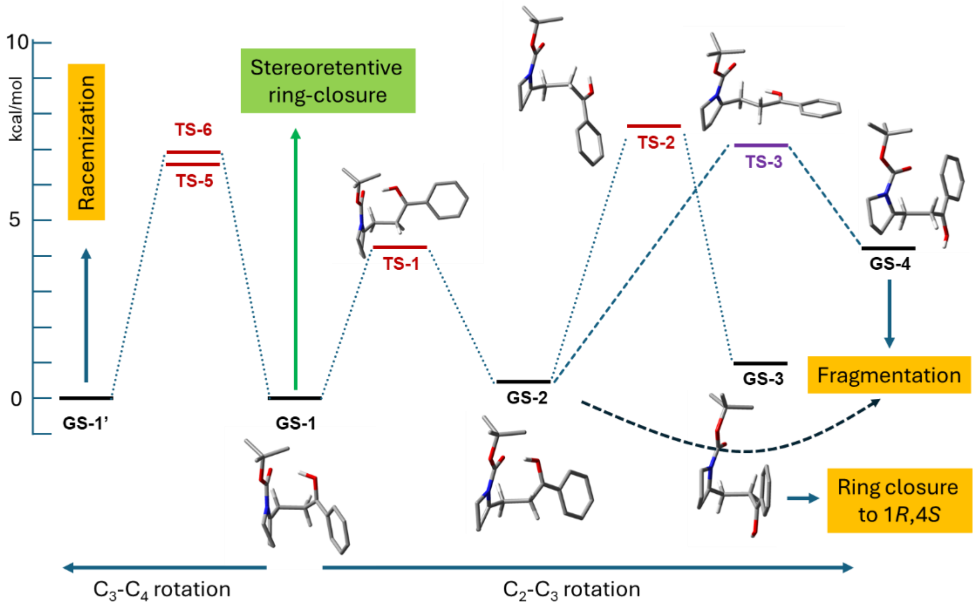

Figure S25. Conformational pathways for 1,4-diradical evolution. Full optimization of GSs and TSs at the IEFPCM[DCM]-UB3LYP-GD3BJ/6-311G(d,p) level.

Considering how the rotational rate constants (k_{ROT}) of the investigated dihedrals (C1-C2, C2-C3 and C3-C4) increase with temperature, other possible pathways become competitive, thus leading to erosion of stereochemical fidelity and reaction-channel selectivity.

Given the calculated conformational landscape, hydrogen bonding in **Int-A** (**GS-1**) biases both stereochemical fidelity in the Yang photocyclization and reaction-channel selectivity, in line with the experimental findings described in the manuscript.

G. References

(1) Li, Y.-Q.; Wang, H.-J.; Huang, Z.-Z. Morita–Baylis–Hillman reaction of α,β-unsaturated ketones with allylic acetates by the combination of transition-metal catalysis and organomediation. *J. Org. Chem.* **2016**, *81*, 4429–4433.

(2) Zhang, Y.; Liu, B.; Wu, X.; Li, R.; Ning, X.; Liu, Y.; Liu, Z.; Ge, Z.; Li, R.; Yin, Y. New pyridin-3-ylmethyl carbamodithioic esters activate pyruvate kinase M2 and potential anticancer lead compounds. *Bioorg. Med. Chem.* **2015**, *23*, 4815–4823.

(3) Huang, R.-Z.; Lau, K. K.; Li, Z.; Liu, T.-L.; Zhao, Y. Rhodium-catalyzed enantioconvergent isomerization of homoallylic and bishomoallylic secondary alcohols. *J. Am. Chem. Soc.* **2018**, *140*, 14647–14654.

(4) Lam, N.Y.S.; Dhankhar, J.; Lahdenpera, A.S.K.; Phipps, R.J. Catalytic Enantioselective Hydrogen Atom Abstraction Enables the Asymmetric Oxidation of Meso Diols. *J. Am. Chem. Soc.* **2024**, *146*, 33302–33308.

(5) Stott, K.; Stonehouse, J.; Keeler, J.; Hwang, T. L.; Shaka, A. J. Excitation sculpting in high-resolution nuclear magnetic resonance spectroscopy: Application to selective NOE experiments. *J. Am. Chem. Soc.* **1995**, *117*, 4199–4200 .

(6) Pracht, P. *et al.* CREST—A program for the exploration of low-energy molecular chemical space. *J. Chem. Phys.* **2024**, *160*, 114110.

(7) Bannwarth, C.; Ehlert, S.; Grimme, S. GFN2-xTB—An accurate and broadly parametrized self-consistent tight-binding quantum chemical method with multipole electrostatics and density-dependent dispersion contributions. *J. Chem. Theory Comput.* **2019**, *15*, 1652–1671.

(8) Ehlert, S.; Stahn, M.; Spicher, S.; Grimme, S. Robust and efficient implicit solvation model for fast semiempirical methods. *J. Chem. Theory Comput.* **2021**, *17*, 4250–4261 (2021).

(9) Frisch, M. J. *et al.* Gaussian 16, Revision A.03 (Gaussian, Inc., 2016).

(10) Pracht, P.; Grimme, S. Calculation of absolute molecular entropies and heat capacities made simple. *Chem. Sci.* **2021**, *12*, 6551–6568.

(11) Pescitelli, G.; Bruhn, T. Good computational practice in the assignment of absolute configurations by TDDFT calculations of ECD spectra. *Chirality* **2016**, *28*, 466–474.

(12) Pecorari, D.; Giuliani, E.; Mazzanti, A.; Stagni, S.; Fiorini, V.; Vigarani, G.; Zinna, F.; Pescitelli, G.; Mancinelli, M. Synthesis and stereodynamic and emission properties of dissymmetric bis-aryl carbazole boranes and identification of a CPL-active B–C atropisomeric compound. *J. Org. Chem.* **2023**, *88*, 871–881.

(13) Abraham, H.M.; Grellier, L.; Prior, D.V.; Morris, J.J.: Taylor, P.J. *J. Chem. Soc., Perkin Trans.* **1990**, *2*, 521-529.

(14) Goez, M.; Zubarev, V. Efficient photoionization of a Norrish II Diradical *Angew. Chem. Int. Ed*. **2006**, *45*, 2135-2138.

(15) Moorthy, J. N.; Koner, A.L.; Samanta, S.; Singhal, N.; Nau, W. M.; Weiss, R. G. Diastereomeric discrimination in the lifetimes of Norrish Type II triplet 1,4-biradicals and stereocontrolled partitioning of their reactivity (Yang Cyclization versus Type II fragmentation) *Chem. Eur. J.* **2006**, *12*, 8744 – 8749.

(16) Zimmt, M. B.; Doubleday, C.; Turro, N.J. The rate-determining step for decay of triplet biradicals: intersystem crossing vs. chain dynamics. *J. Am. Chem. Soc.* **1986**, *108*, 3618-3620.

(17) Stephens, P. J.; Devlin, F. J.; Chablowski, C. F.; Frisch, M. J. Ab Initio Calculation of Vibrational Absorption and Circular Dichroism Spectra Using Density Functional Force Fields. *J. Phys. Chem.* **1994**, *98*, 11623.

(18) Grimme, S.; Antony, J.; Ehrlich, S.; Krieg, H. A consistent and accurate ab initio parameterization of density functional dispersion correction (DFT-D) for the 94 elements H-Pu. *J. Chem. Phys.* **2010**, *132* ,154104.

(19) Tomasi, J.: Mennucci, B.; Cammi, R. Quantum mechanical continuum solvation models, *Chem. Rev.* **2005**, *105*, 2999-3093.

(20) Luchini, G.; Alegre-Requena, J.V.; Funes-Ardoiz, I.; Paton, R.S. GoodVibes: automated thermochemistry for heterogeneous computational chemistry data. *F1000Research* **2020**, *9*, 291.

(21) Grimme, S. Supramolecular Binding Thermodynamics by Dispersion-Corrected Density Functional Theory. *Chem. Eur. J.* **2012**, *18*, 9955-9964.

(22) Testa, B. *Principles of Organic Stereochemistry* (Marcel Dekker, New York, **1979**).

(23) Maryott, A. A. & Smith, E. R. *Tables of Dielectric Constants of Pure Liquids* (National Bureau of Standards Circular 514, Washington, DC, **1951**).

H. NMR Spectra

^{1}H NMR of **1a** (600 MHz, $CDCl_3$) – mixture of rotamers

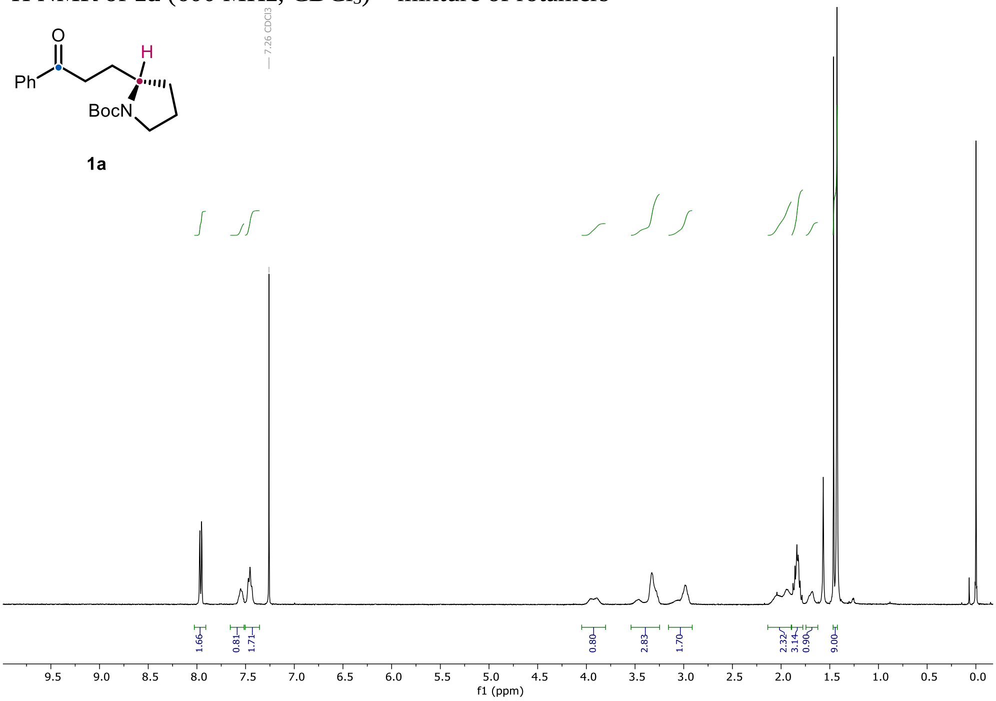

^{13}C NMR of **1a** (151 MHz, $CDCl_3$) – mixture of rotamers

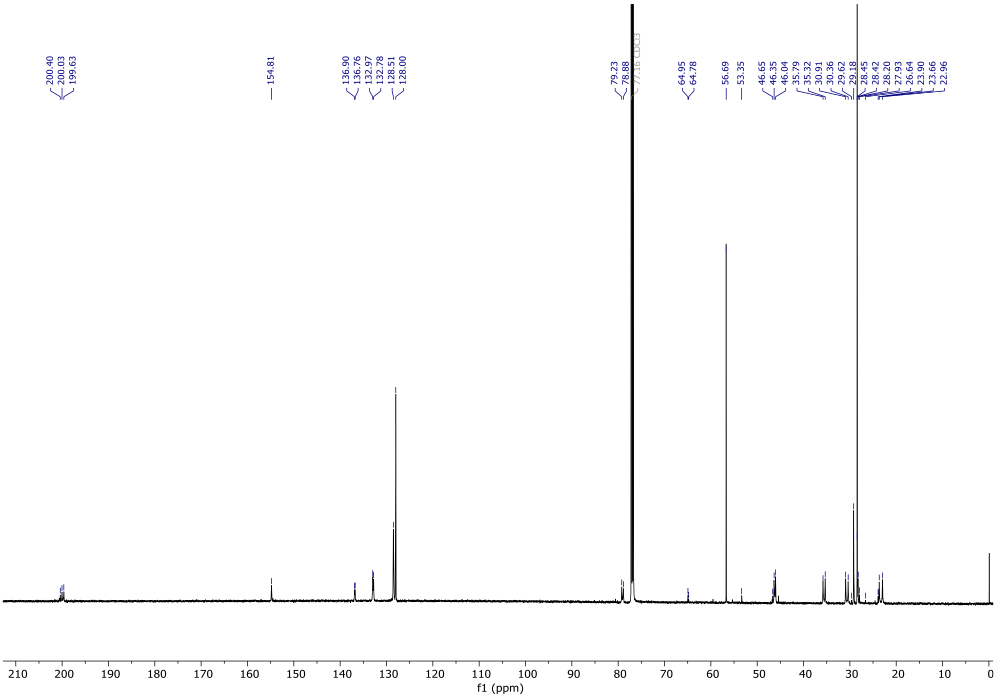

^{1}H NMR of **1b** (600 MHz, $CDCl_3$) – mixture of rotamers

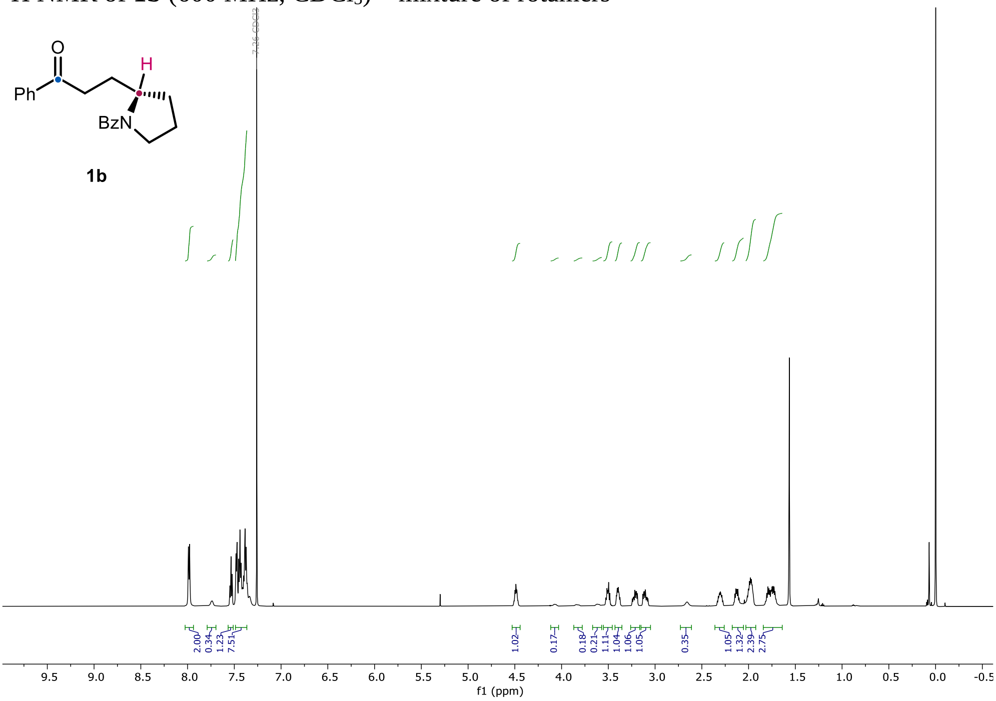

^{13}C NMR of **1b** (151 MHz, $CDCl_3$) – mixture of rotamers

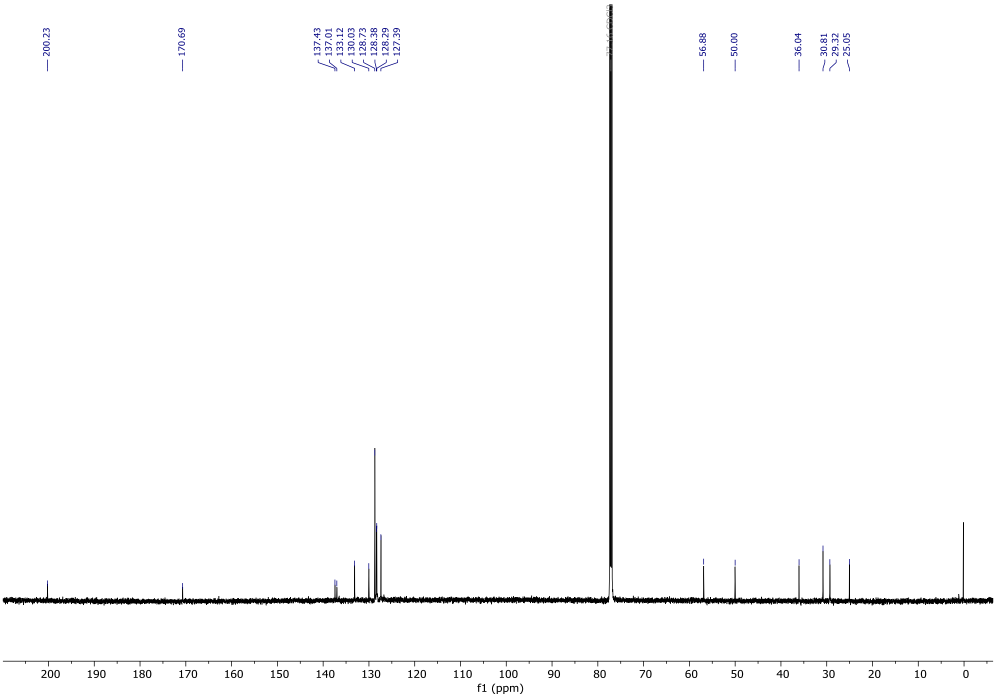

^{1}H NMR of **1c** (600 MHz, $CDCl_3$) – mixture of rotamers

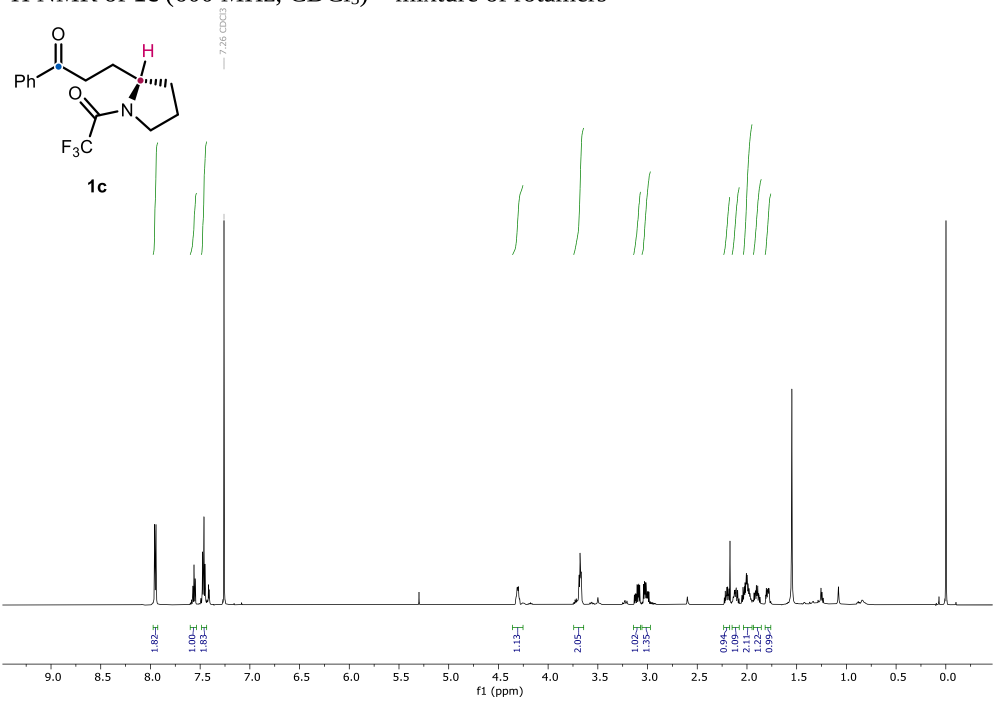

^{13}C NMR of **1c** (151 MHz, $CDCl_3$) – mixture of rotamers

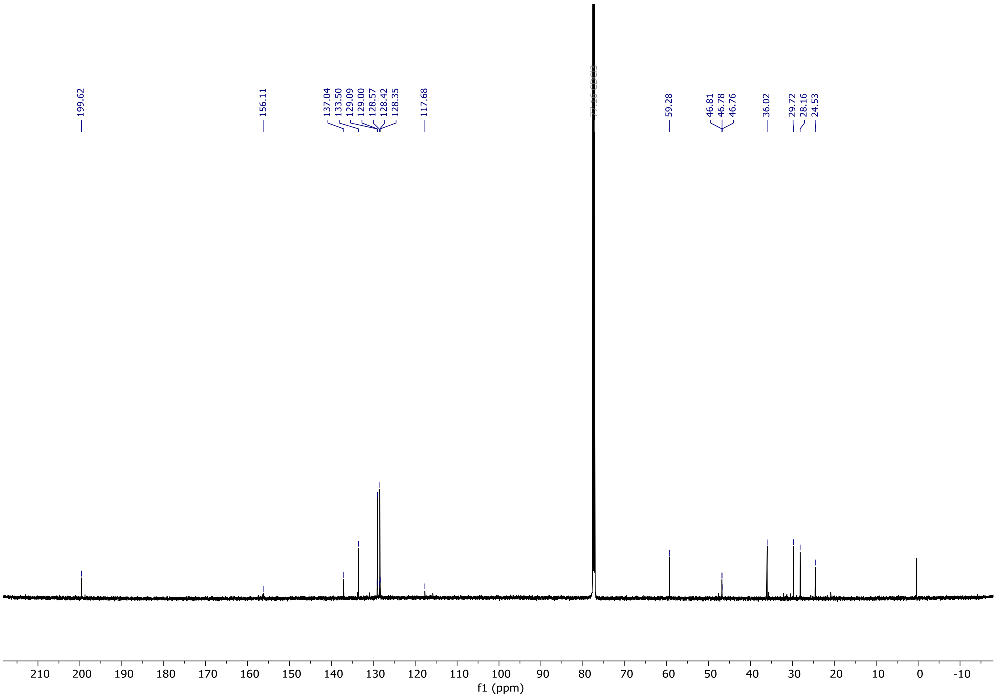

^{19}F NMR of **1c** (376 MHz, $CDCl_3$) – mixture of rotamers

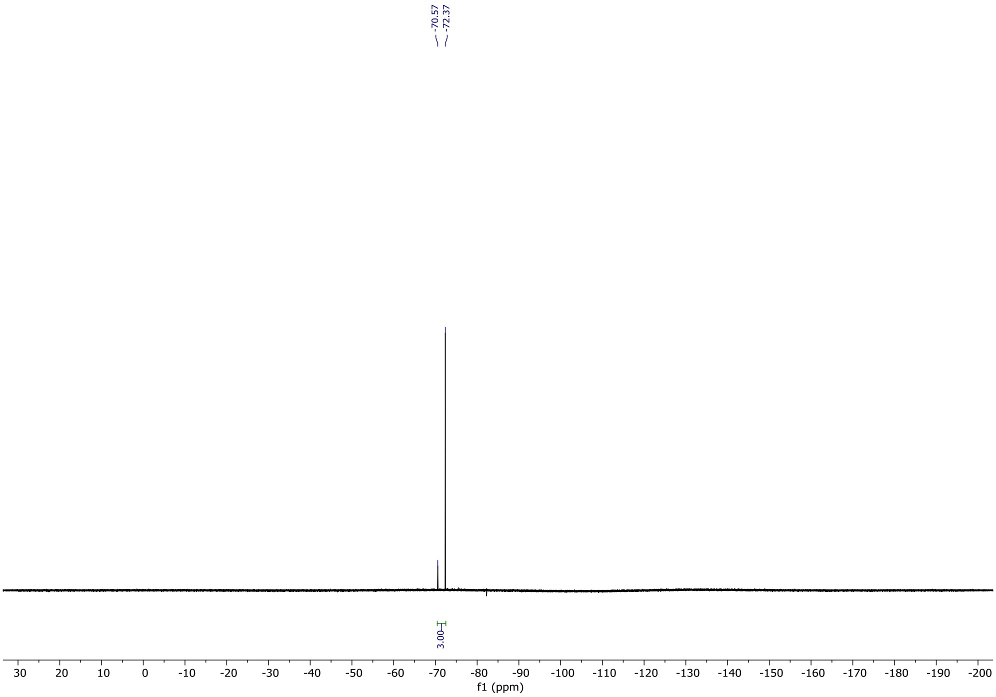

^{1}H NMR of **1d** (600 MHz, $CDCl_3$)

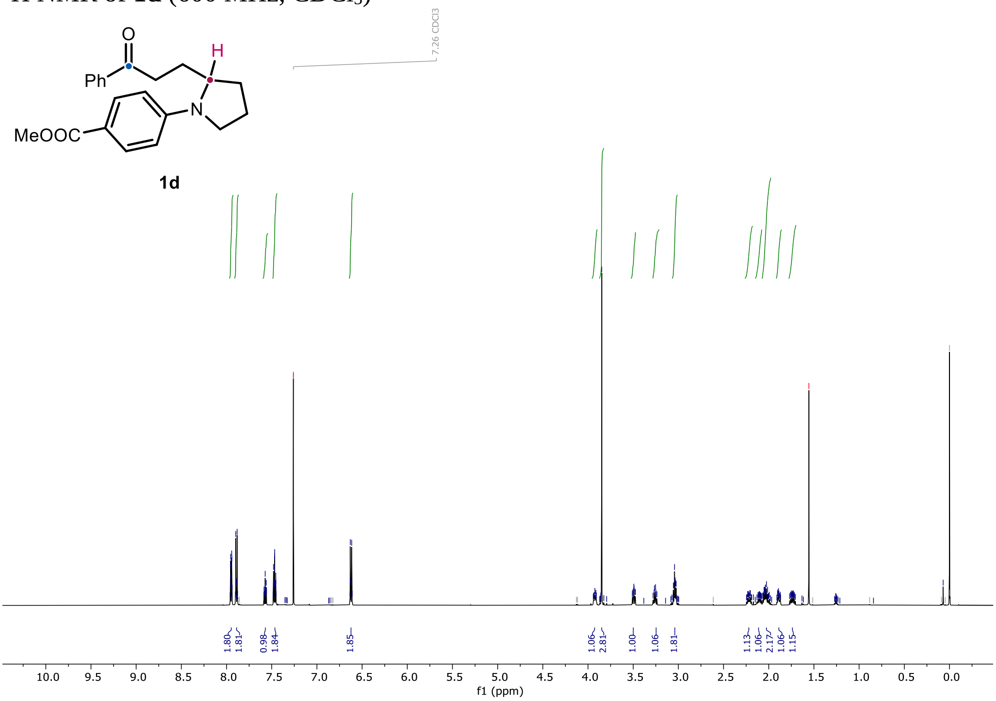

^{13}C NMR of **1d** (151 MHz, $CDCl_3$)

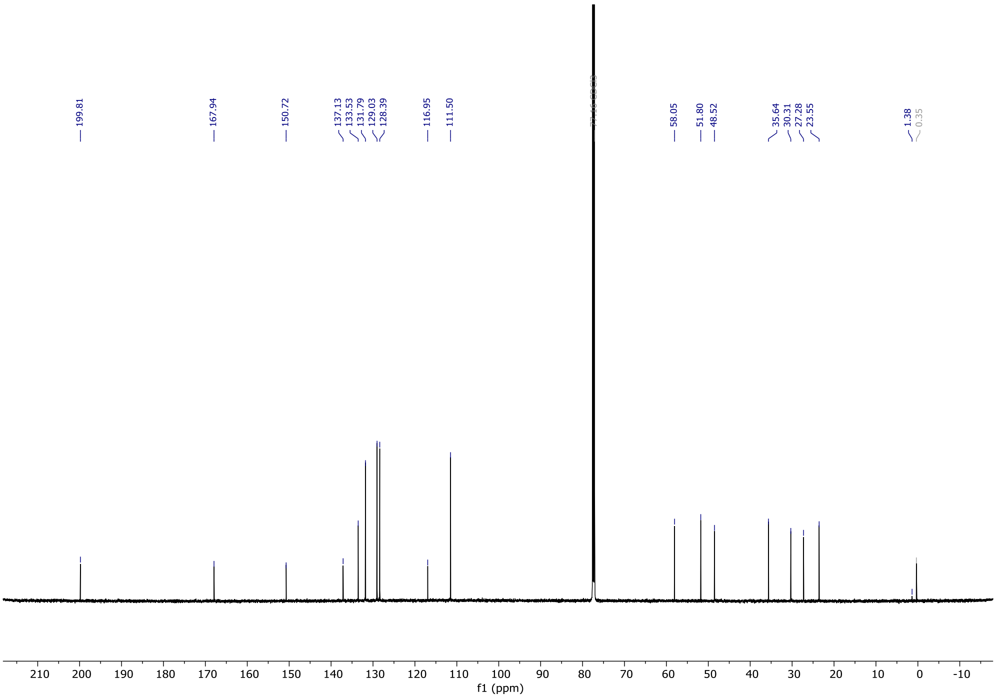

^{1}H NMR of **1e** (600 MHz, $CDCl_3$)

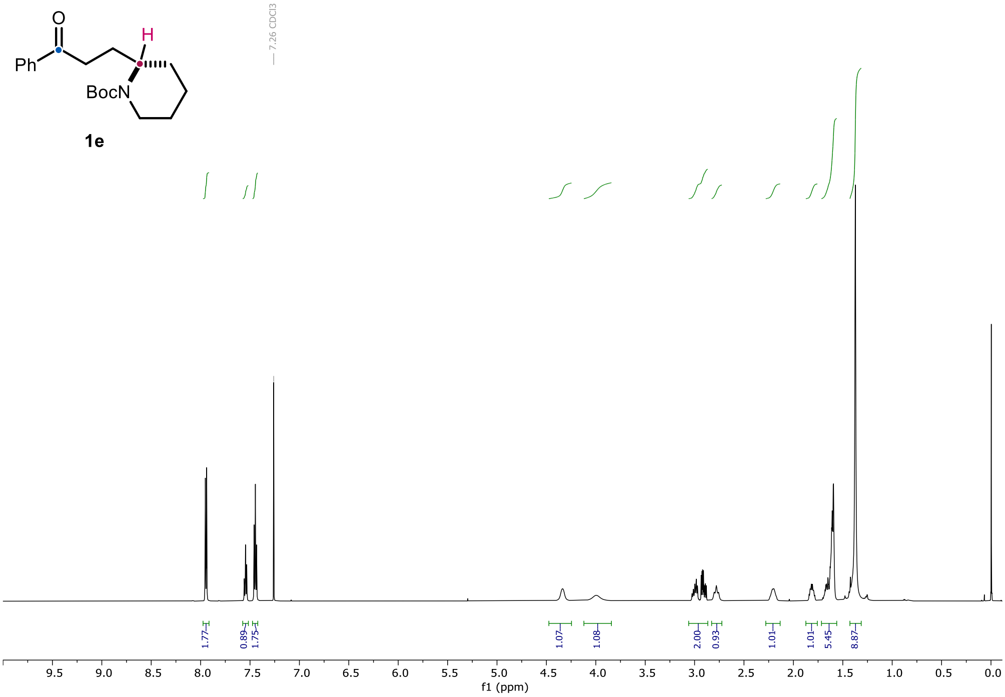

^{13}C NMR of **1e** (151 MHz, $CDCl_3$)

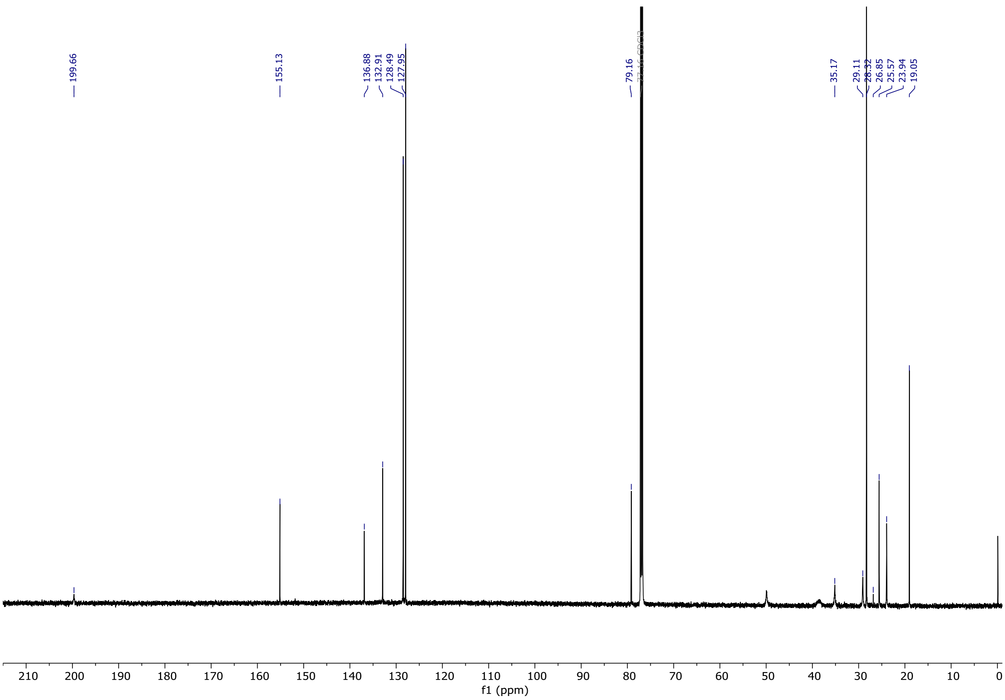

^{1}H NMR of **1f** (600 MHz, $CDCl_3$)

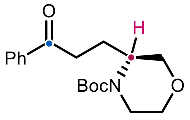

1f

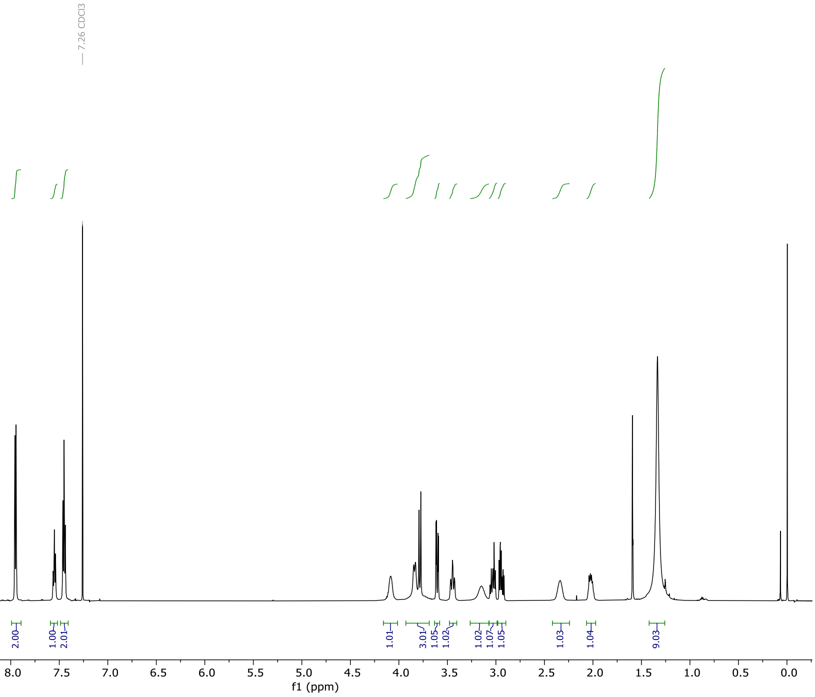

^{13}C NMR of **1f** (151 MHz, $CDCl_3$)

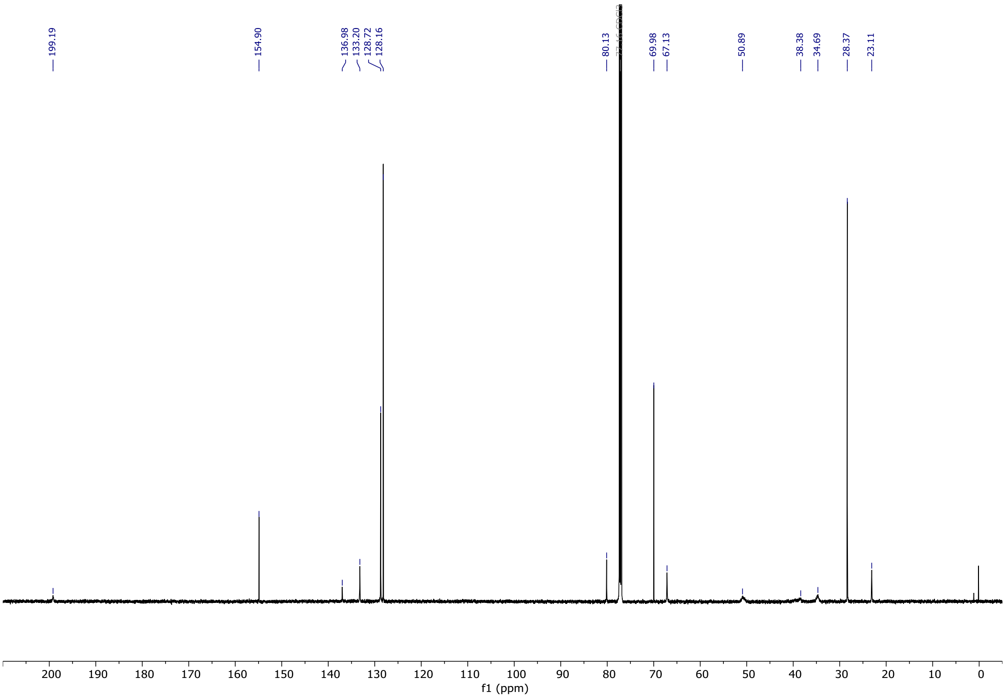

^{1}H NMR of **1g** (600 MHz, $CDCl_3$)

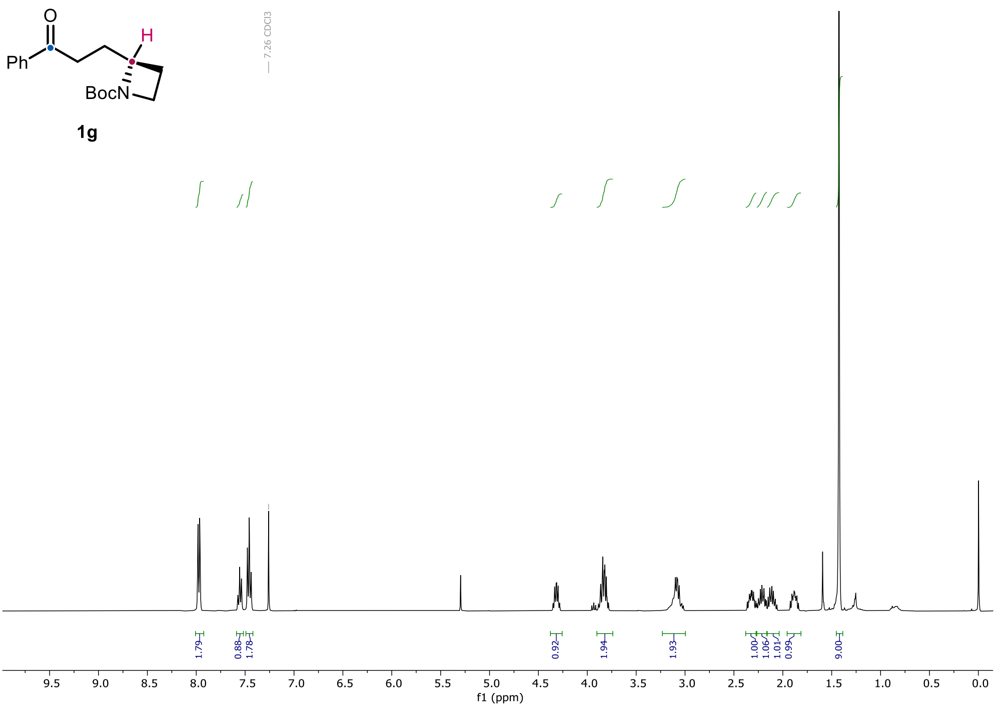

^{13}C NMR of **1g** (151 MHz, $CDCl_3$)

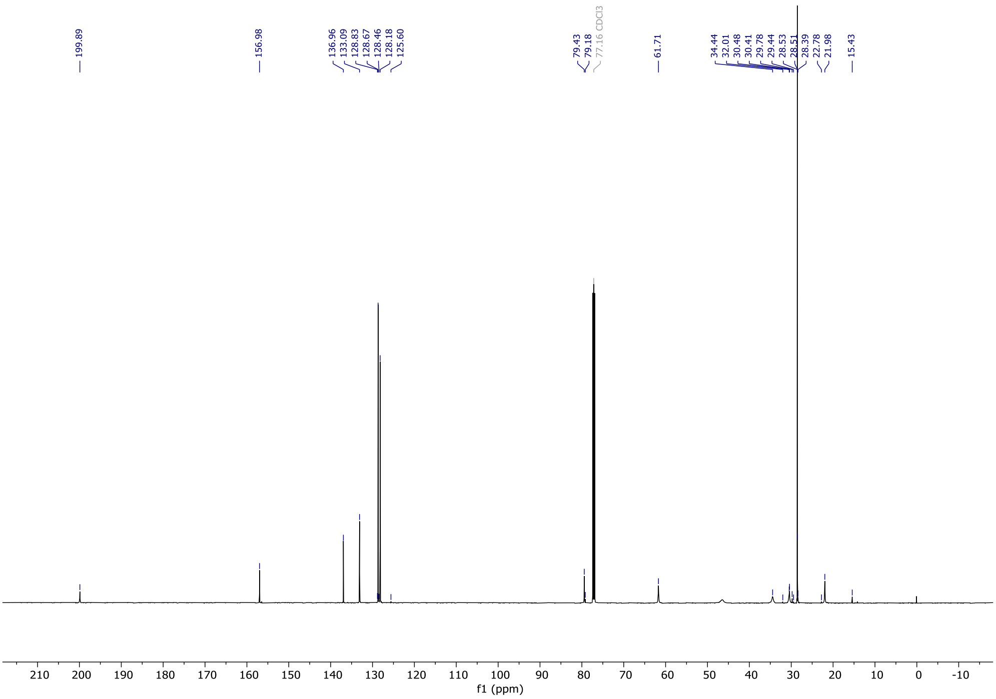

^{1}H NMR of **1h** (600 MHz, $CDCl_3$) – mixture of rotamers

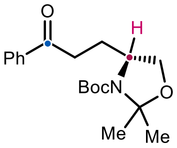

1h

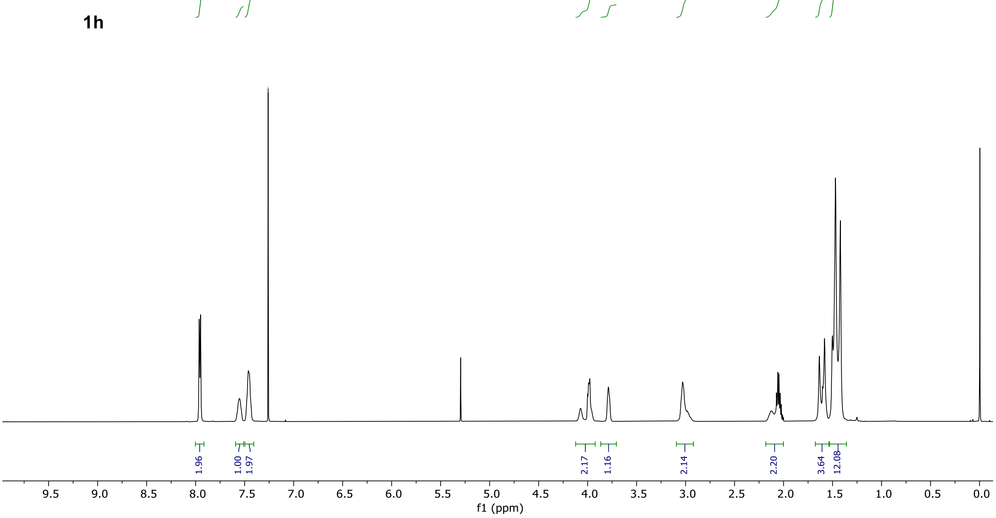

^{13}C NMR of **1h** (151 MHz, $CDCl_3$) – mixture of rotamers

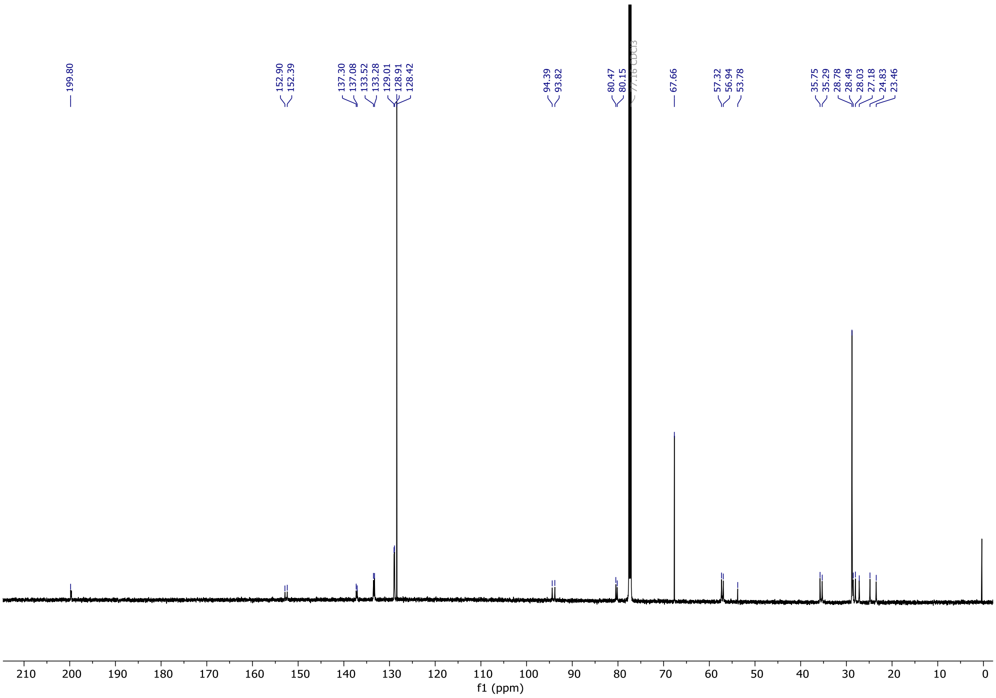

^{1}H NMR of **1i**(600 MHz, $CDCl_3$)

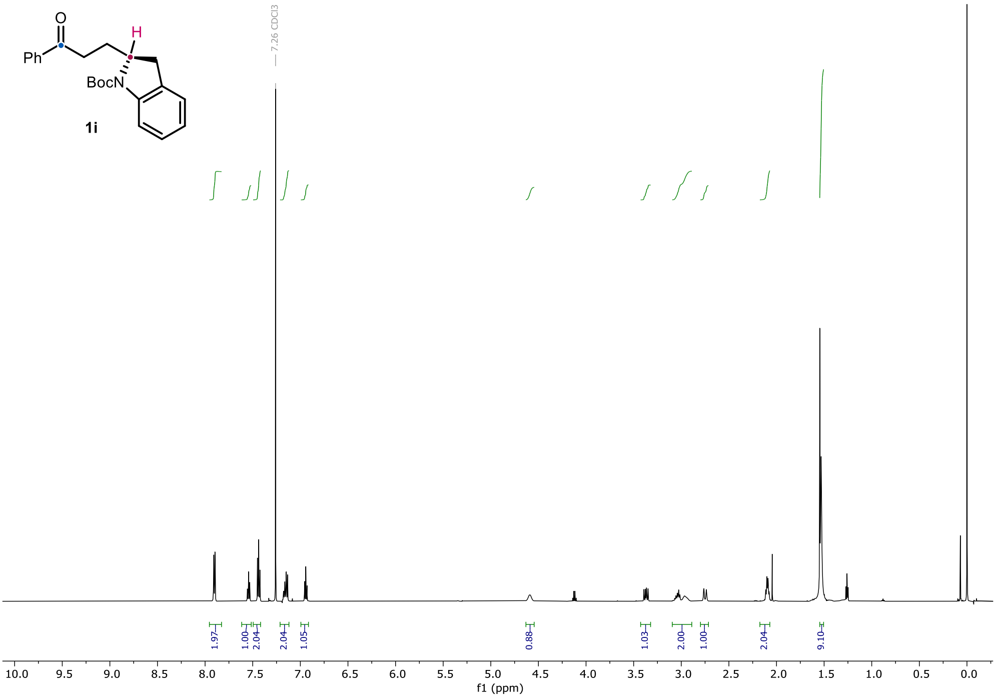

^{13}C NMR of **1i** (151 MHz, $CDCl_3$)

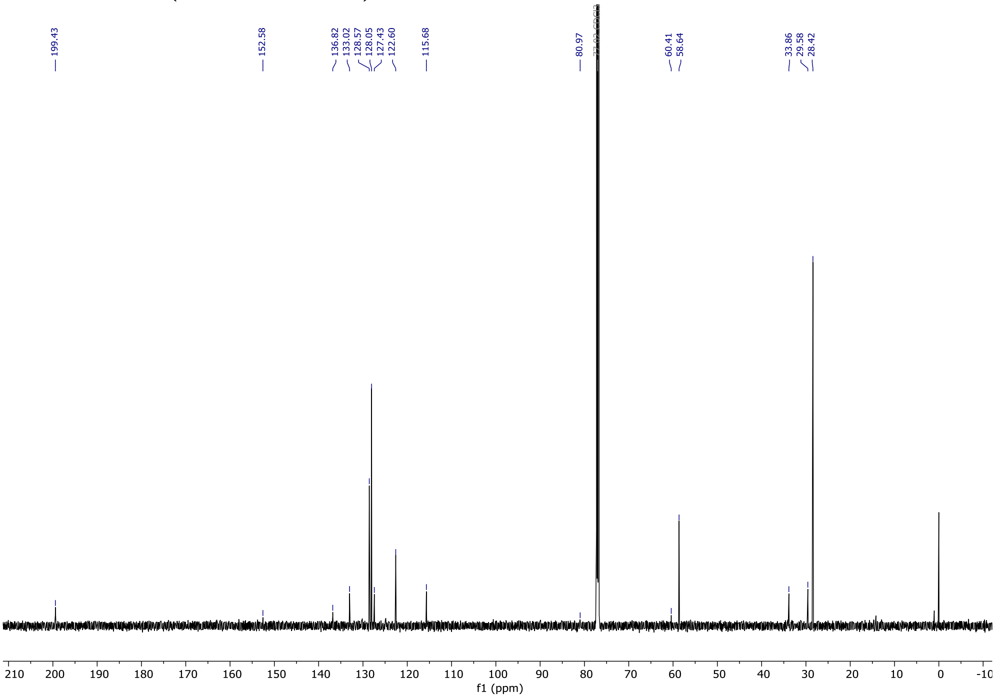

^{1}H NMR of **1j** (600 MHz, $CDCl_3$)– mixture of rotamers

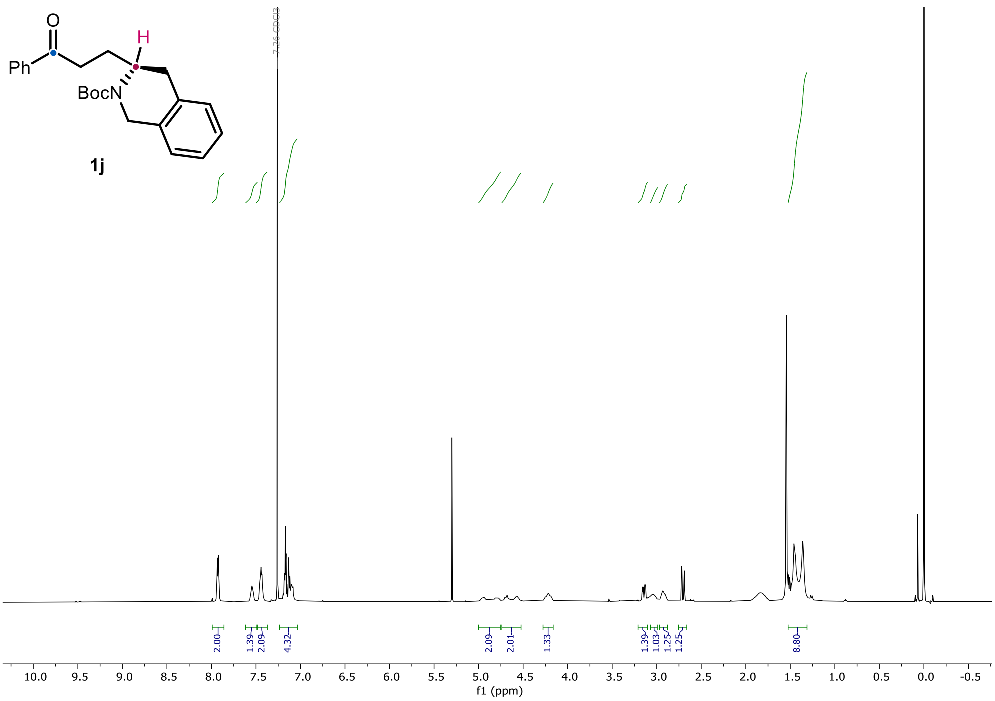

^{13}C NMR of **1j** (151 MHz, $CDCl_3$) – mixture of rotamers

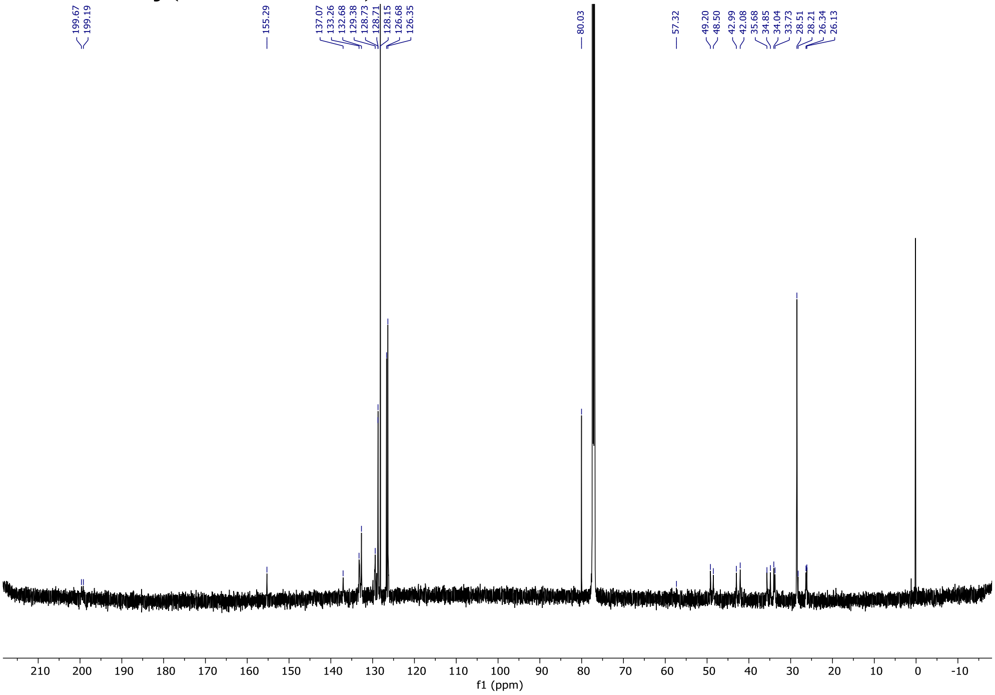

^{1}H NMR (600 MHz, $CDCl_3$) of **1k** – mixture of rotamers

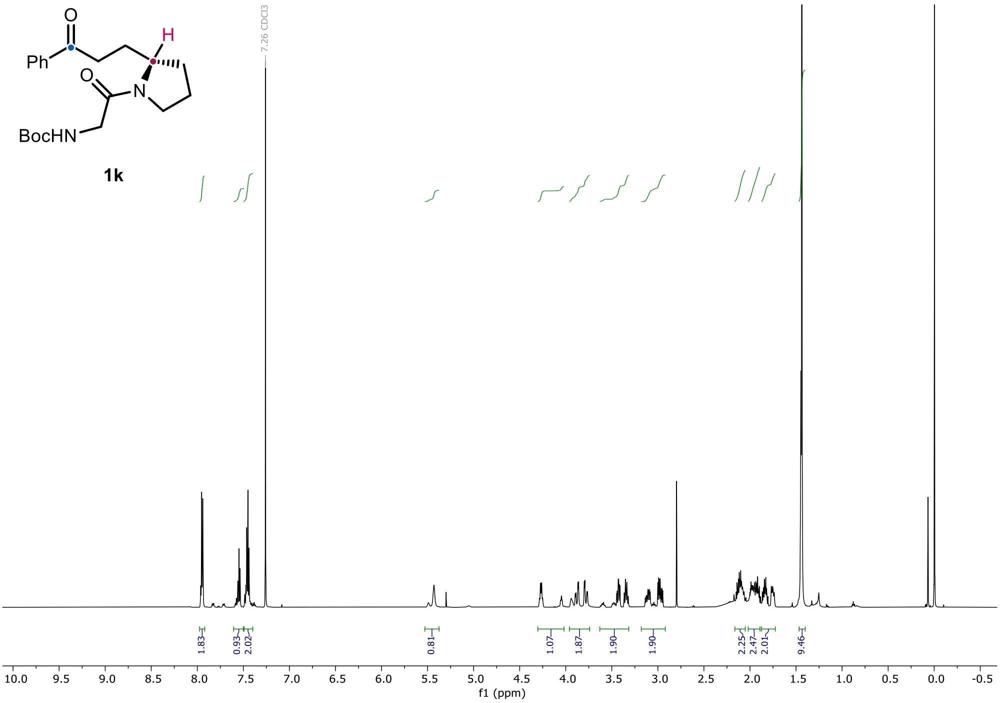

^{13}C NMR (151 MHz, $CDCl_3$) of **1k** – mixture of rotamers

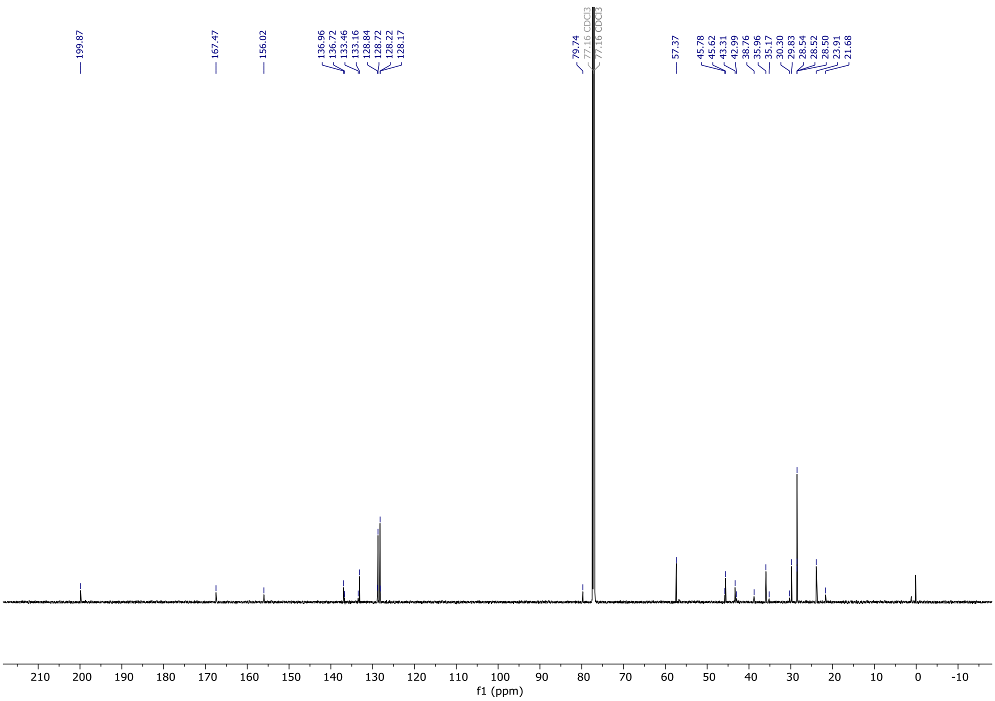

^{1}H NMR of **5a** (600 MHz, $CDCl_3$)

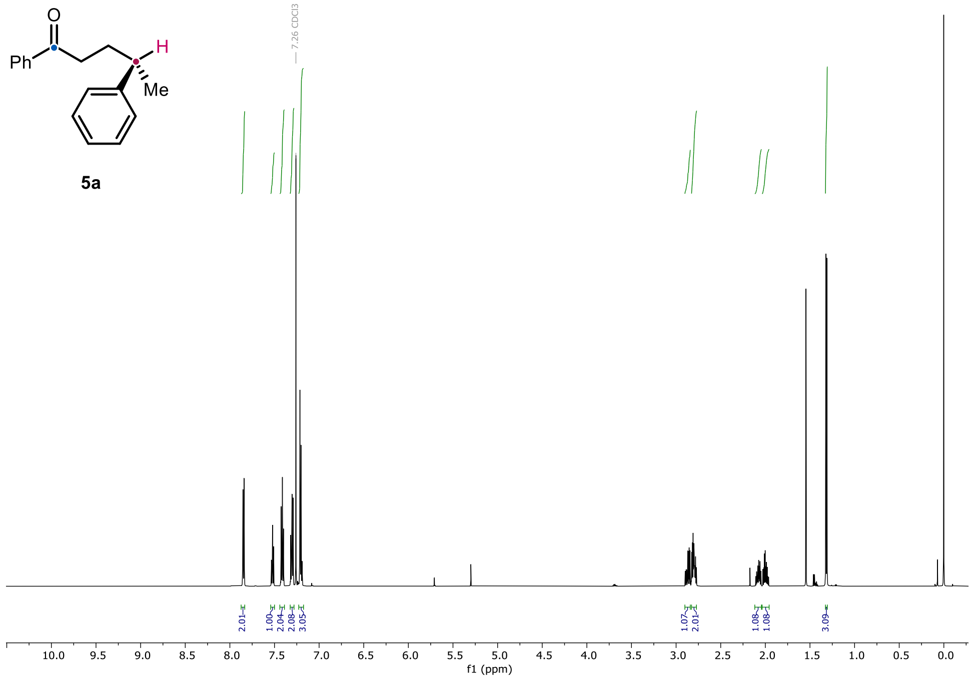

^{13}C NMR of **5a** (151 MHz, $CDCl_3$)

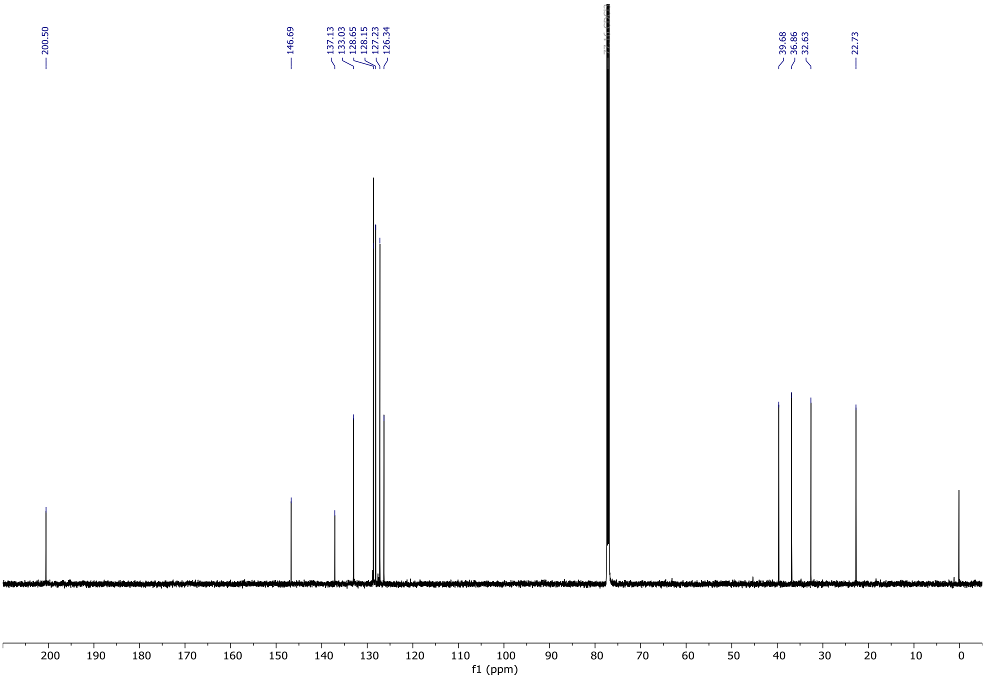

^{1}H NMR of **5b** (600 MHz, $CDCl_3$)

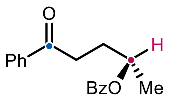

5b

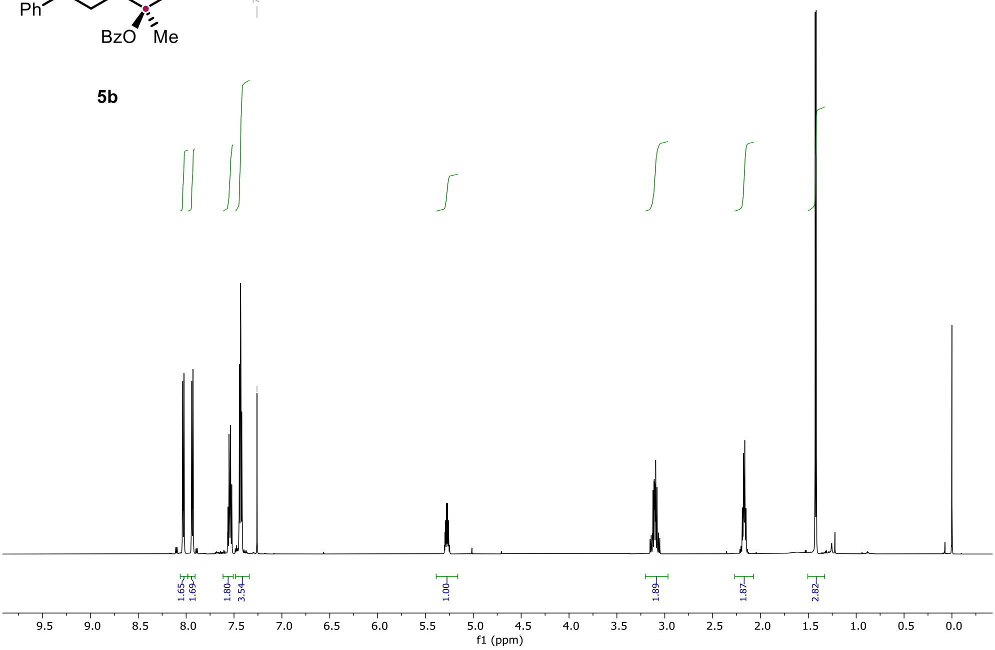

^{13}C NMR of **5b** (151 MHz, $CDCl_3$)

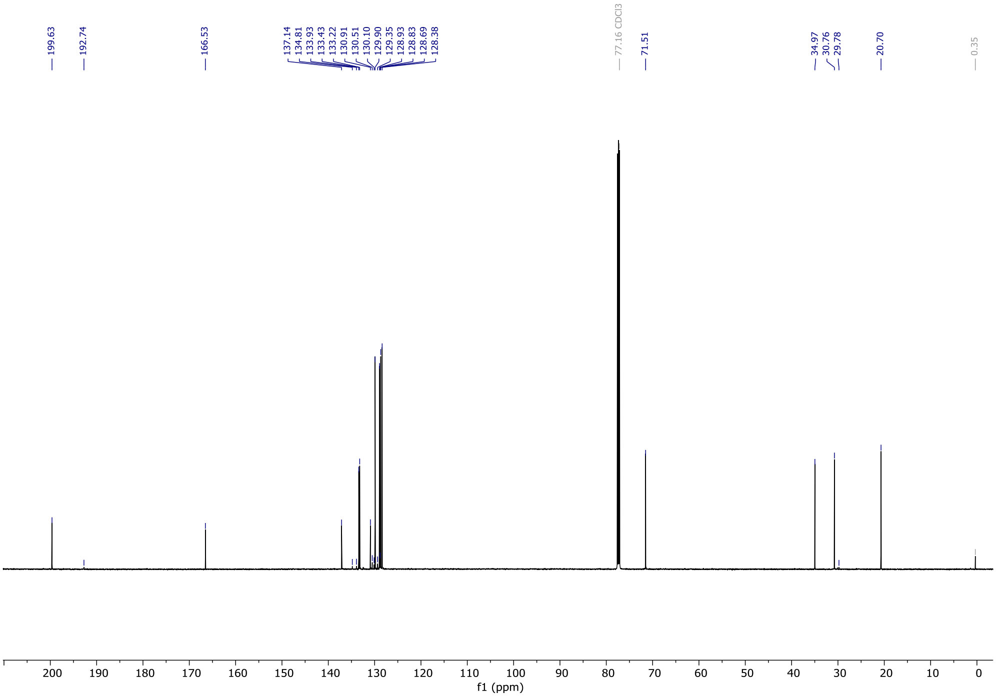

^{1}H NMR of **5c** (600 MHz, $CDCl_3$)

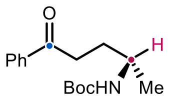

5c

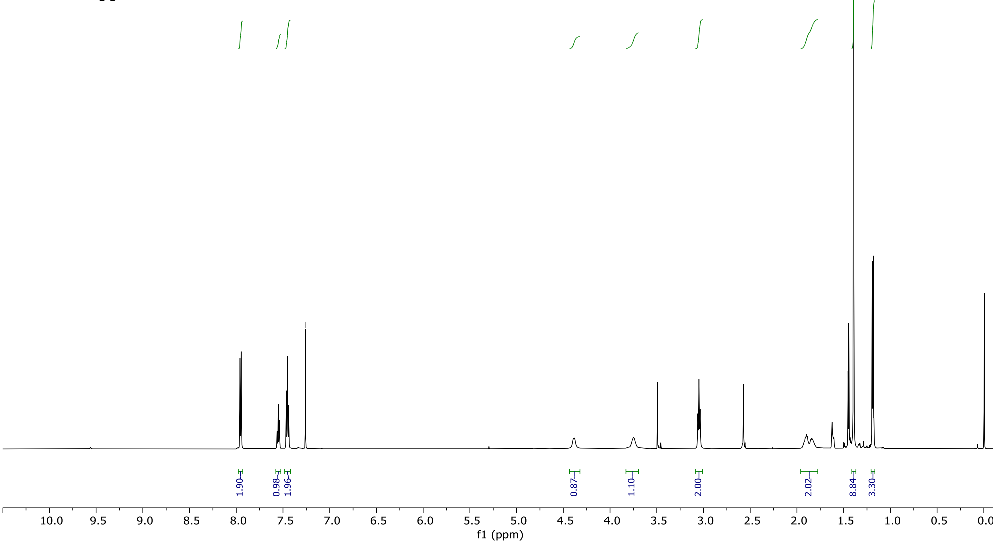

^{13}C NMR of **5c** (151 MHz, $CDCl_3$)

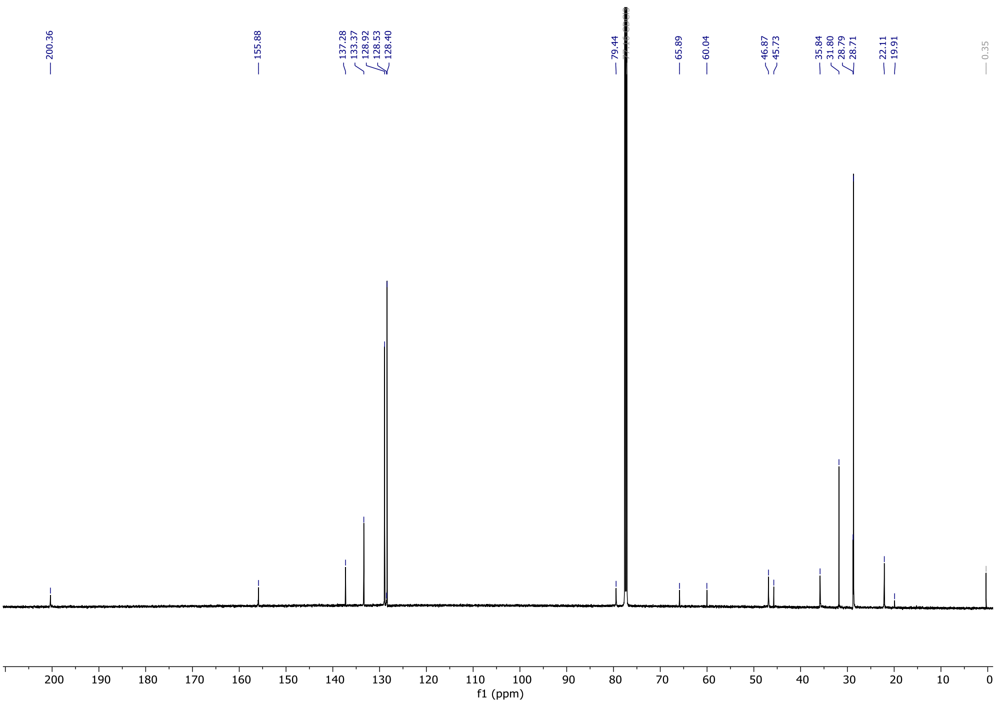

^{1}H NMR of **5d** (600 MHz, $CDCl_3$)

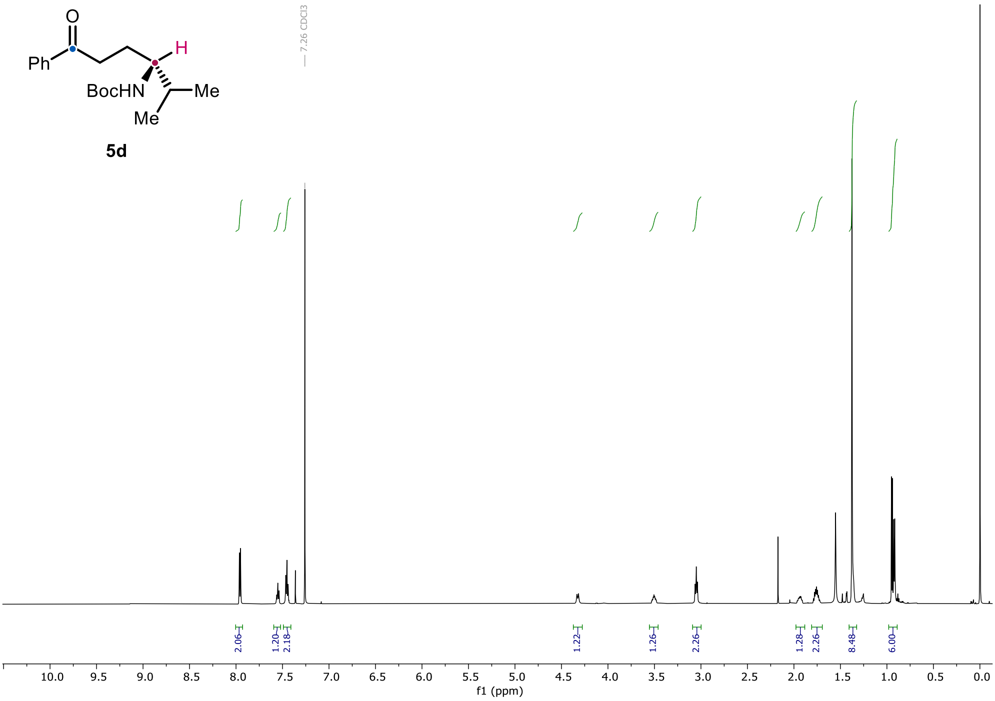

^{13}C NMR of **5d** (151 MHz, $CDCl_3$)

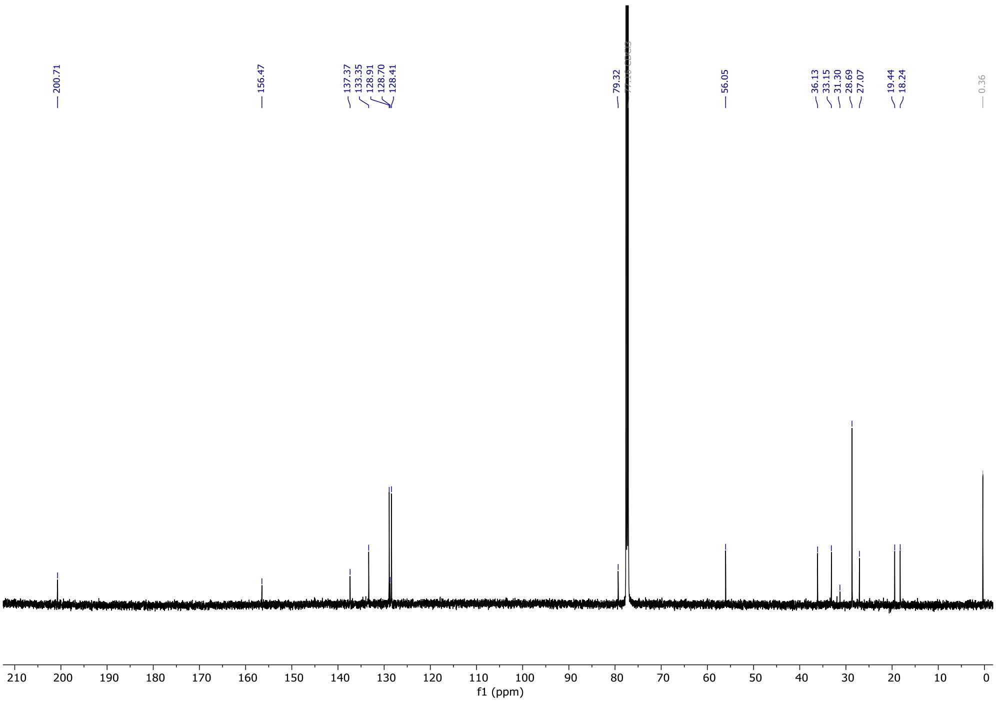

^{1}H NMR of **5e** (600 MHz, $CDCl_3$)

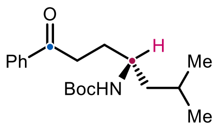

5e

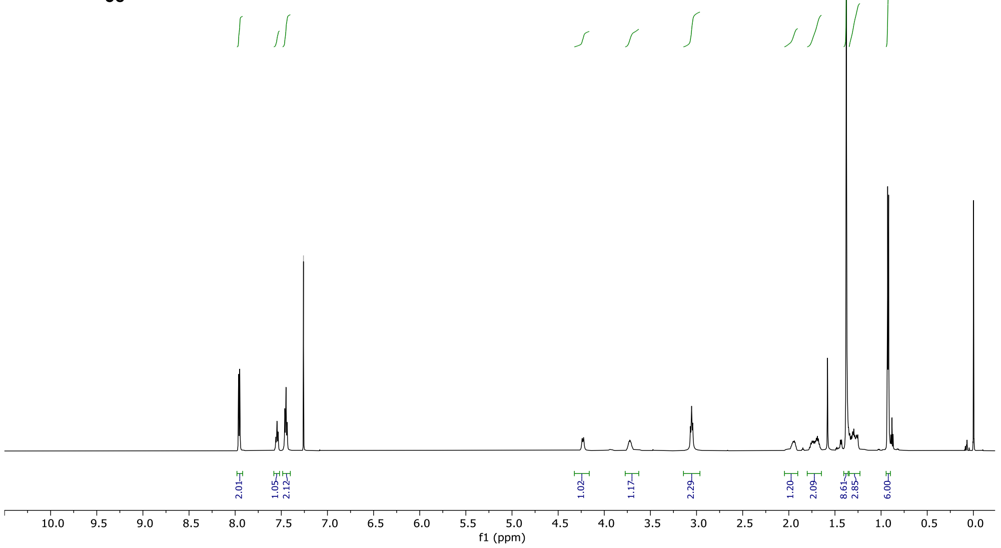

^{13}C NMR of **5e** (151 MHz, $CDCl_3$)

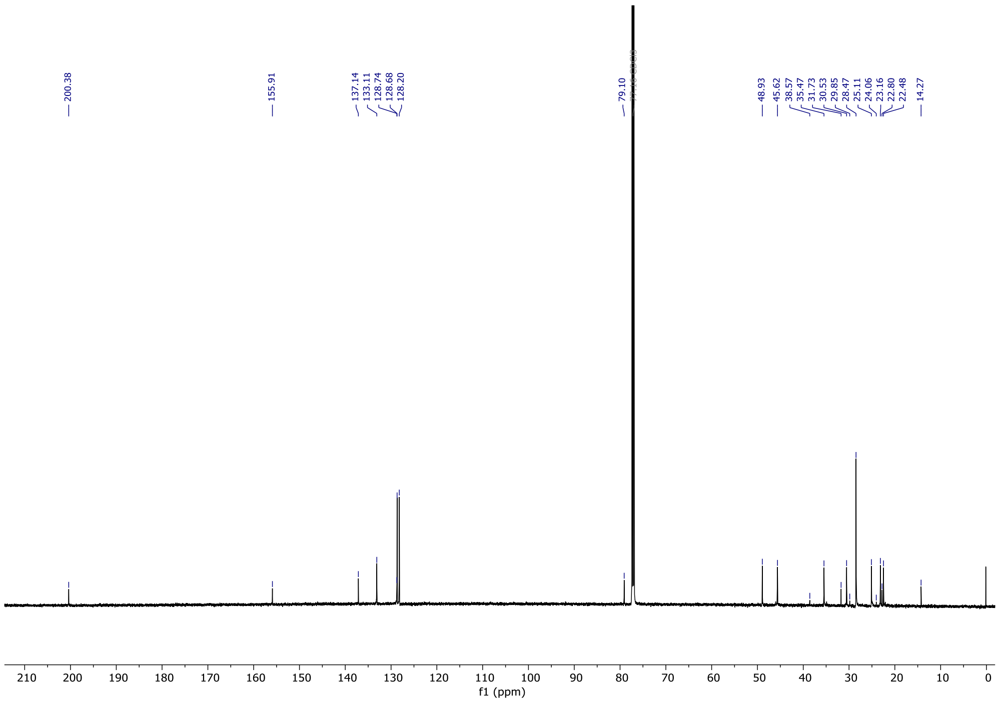

^{1}H NMR of **5f** (600 MHz, $CDCl_3$)

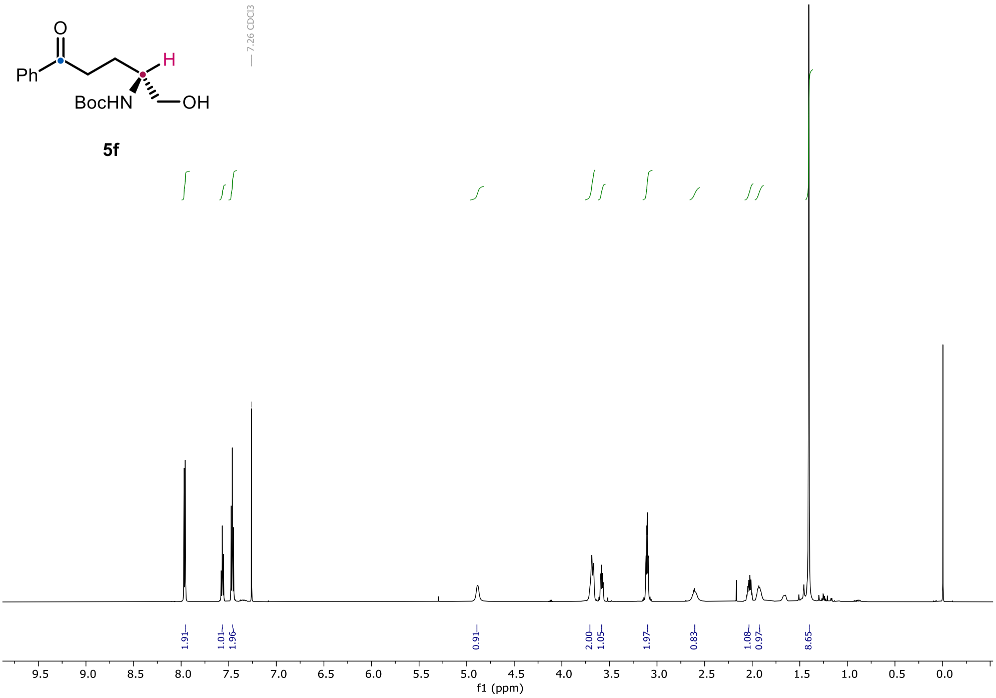

^{13}C NMR of **5f** (151 MHz, $CDCl_3$)

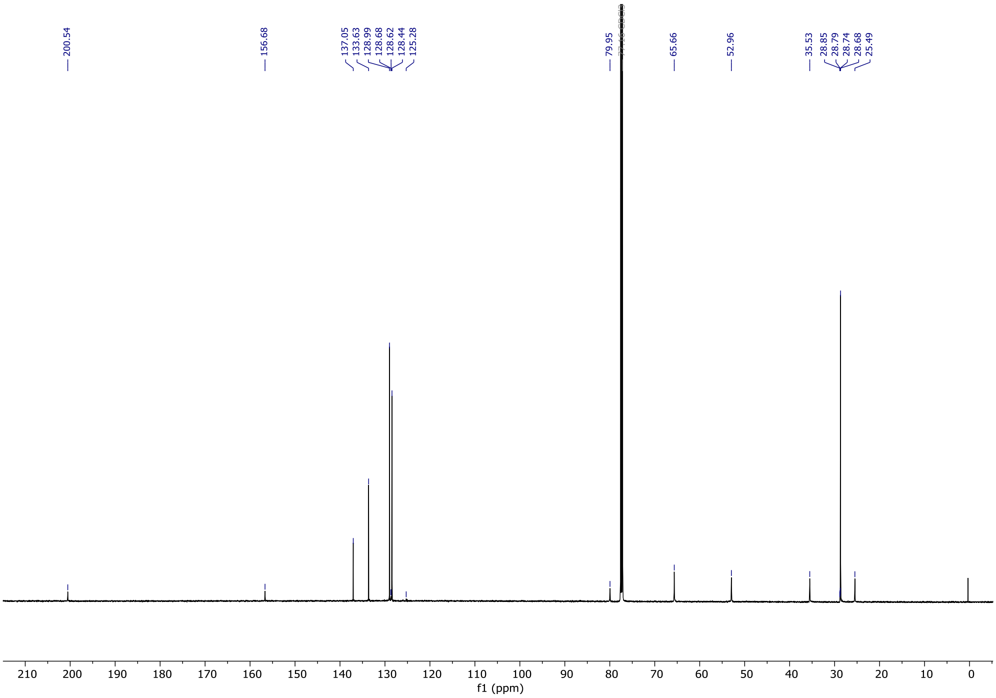

^{1}H NMR of **5g** (600 MHz, $CDCl_3$)

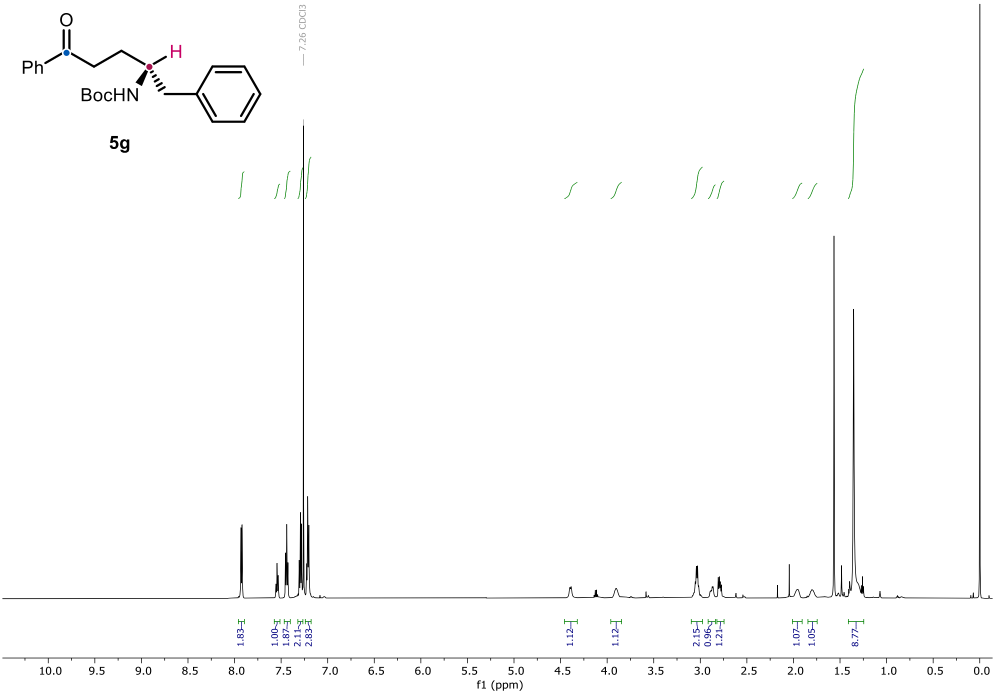

^{13}C NMR of **5g** (151 MHz, $CDCl_3$)

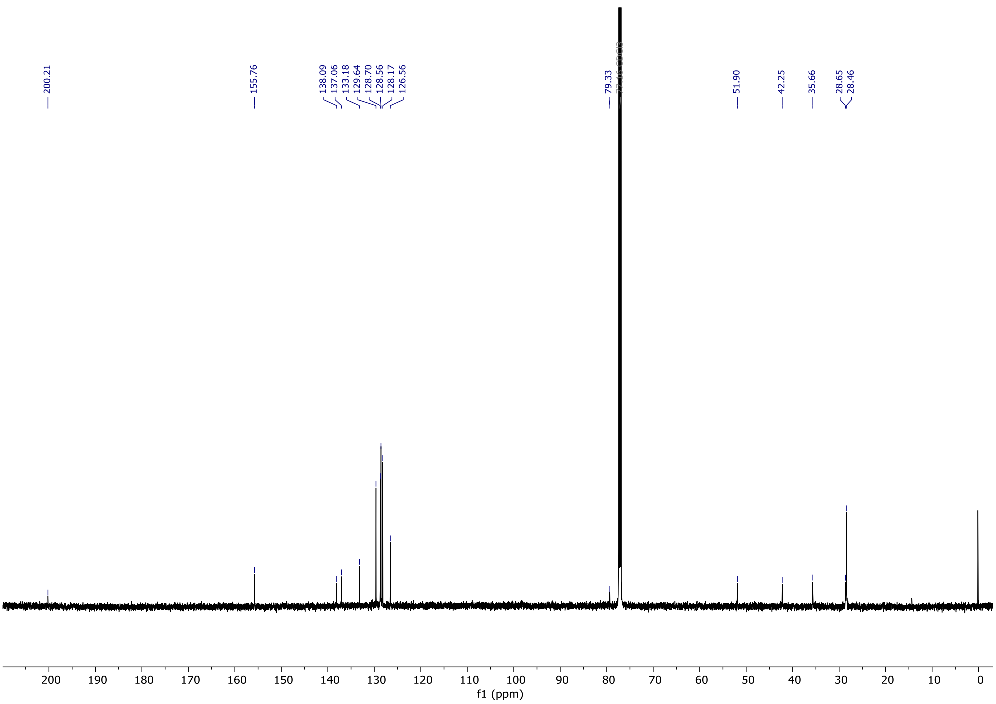

^{1}H NMR of **5h** (600 MHz, $CDCl_3$)

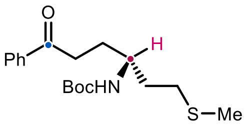

5h

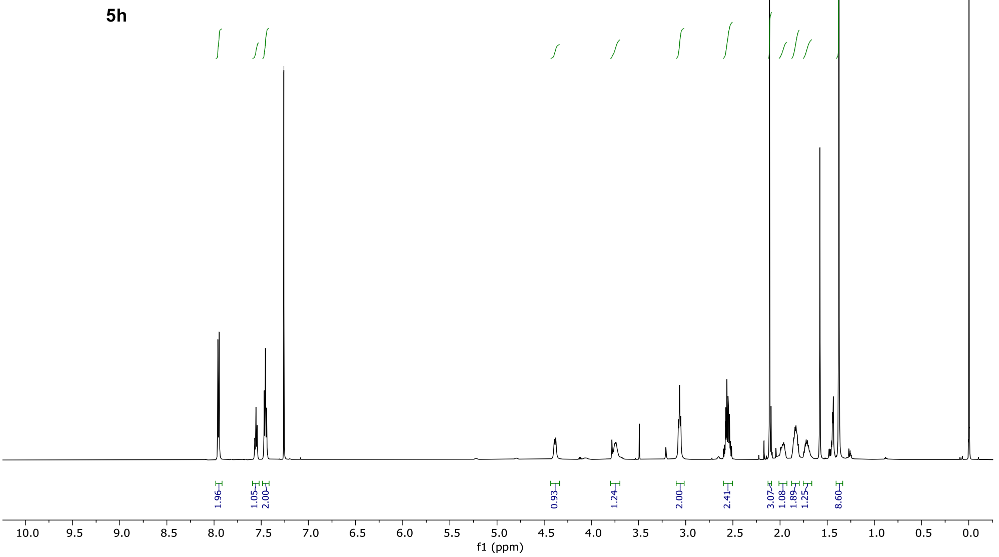

^{13}C NMR of **5h** (151 MHz, $CDCl_3$)

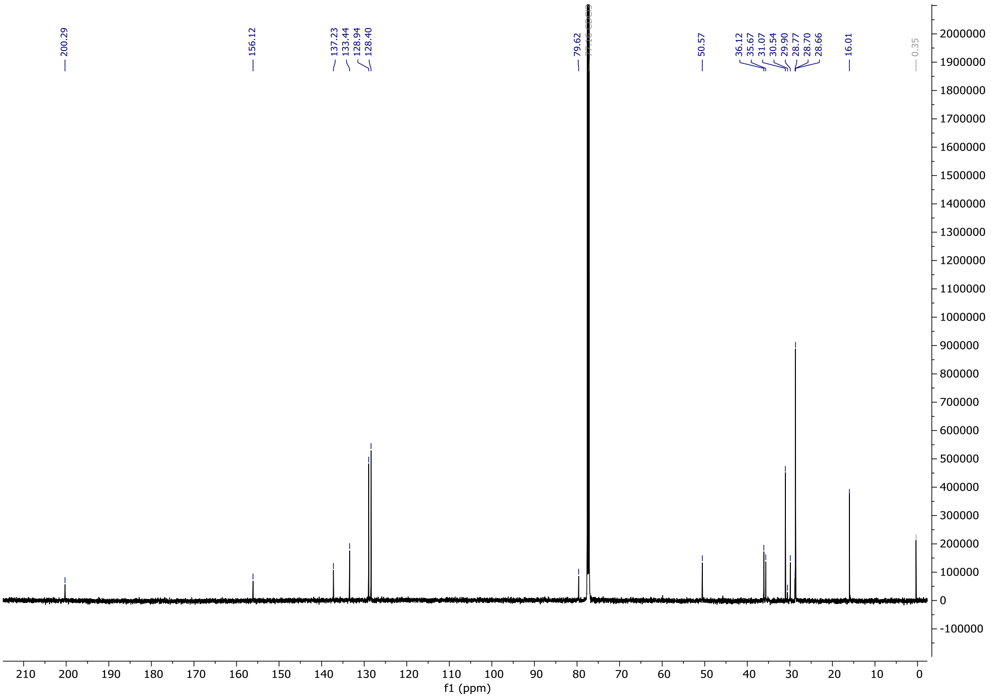

^{1}H NMR of **5i** (600 MHz, $CDCl_3$) – mixture of rotamers

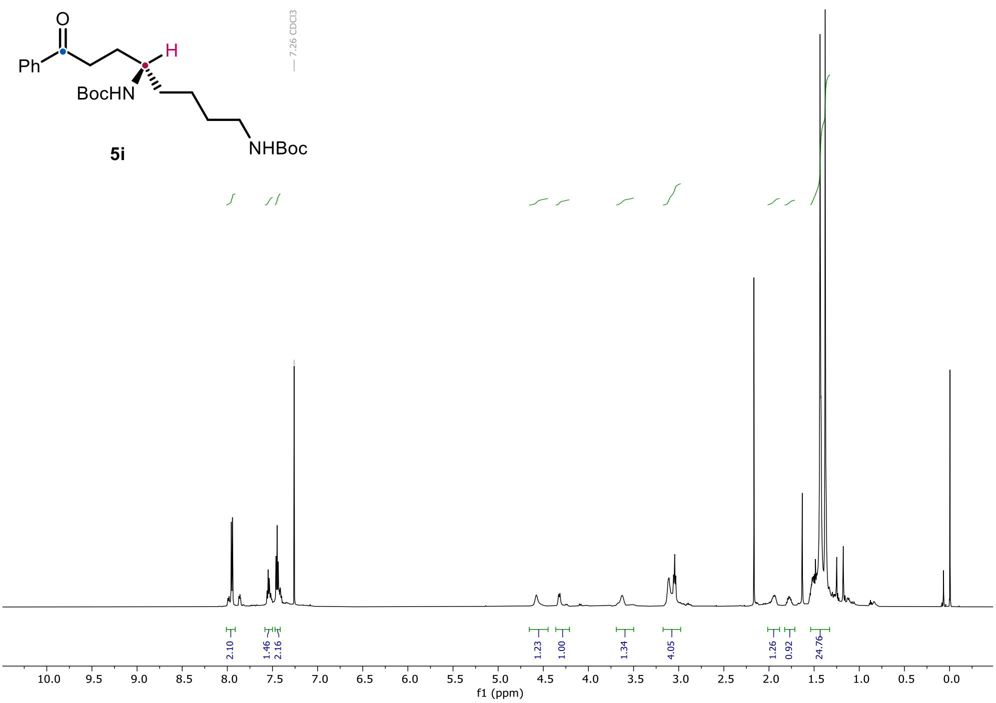

^{13}C NMR of **5i** (151 MHz, $CDCl_3$) – mixture of rotamers

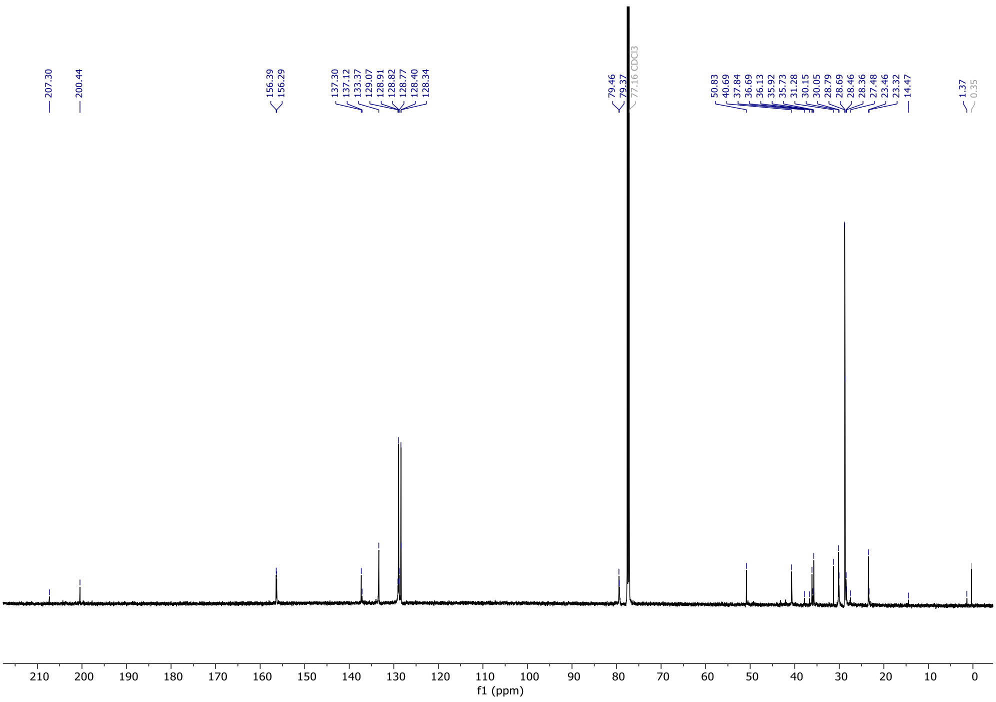

^{1}H NMR of **5j** (600 MHz, $CDCl_3$)

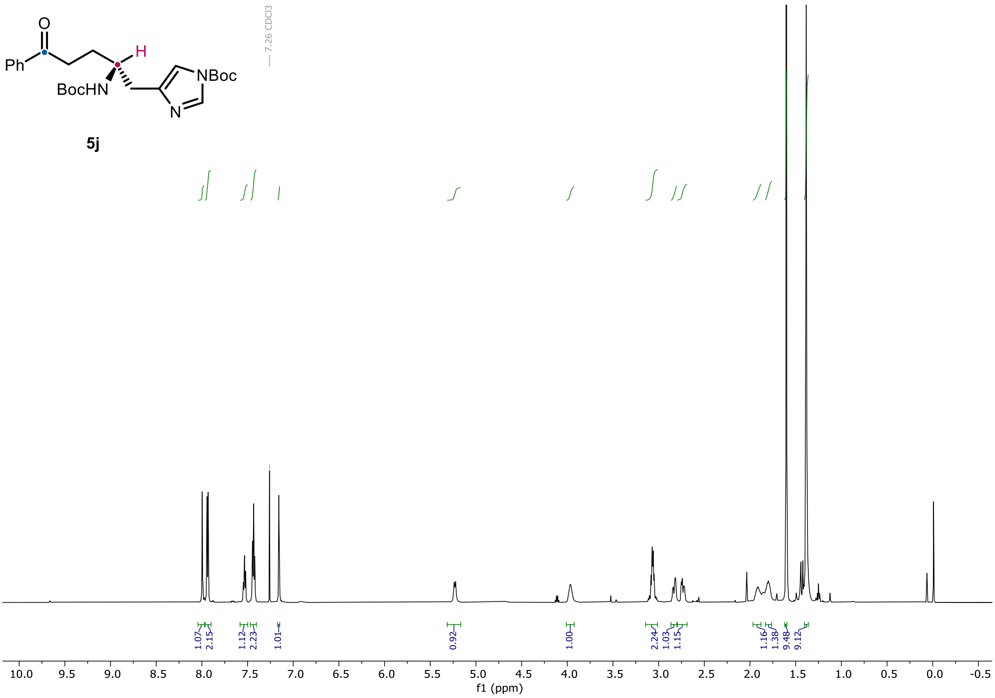

^{13}C NMR of **5j** (151 MHz, $CDCl_3$)

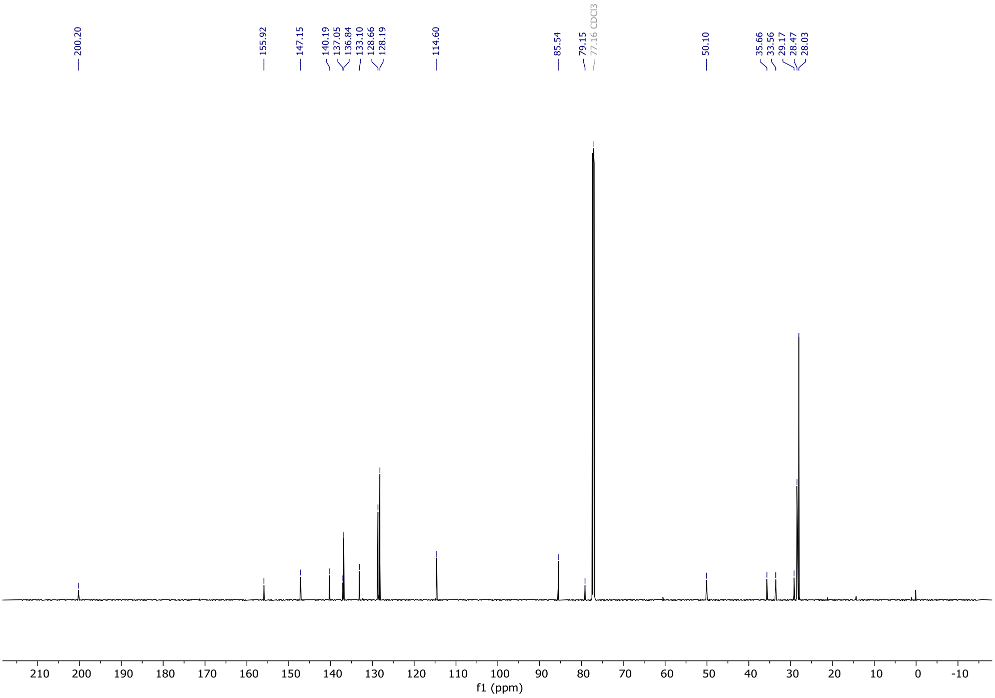

^{1}H NMR of **5k** (600 MHz, $CDCl_3$) – mixture of rotamers

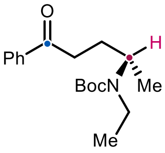

5k

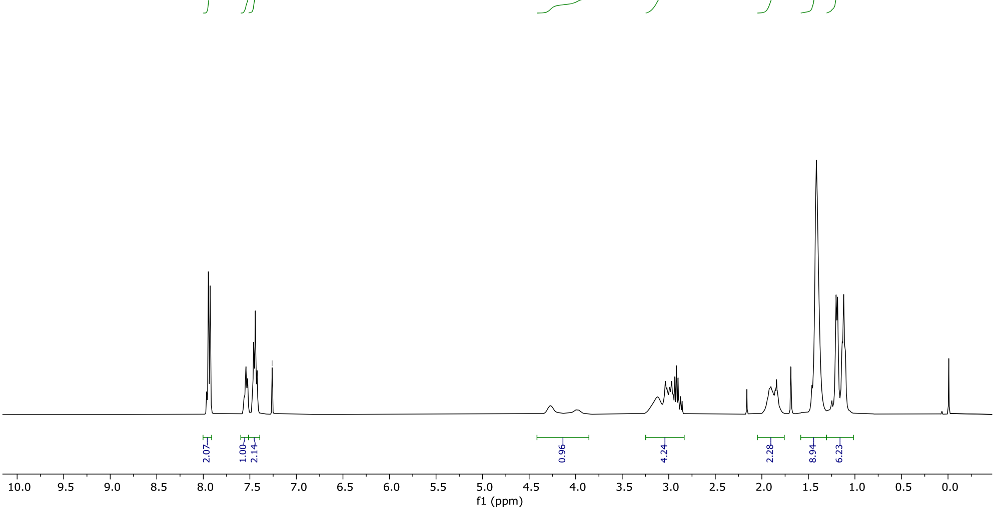

^{13}C NMR of **5k** (151 MHz, $CDCl_3$)

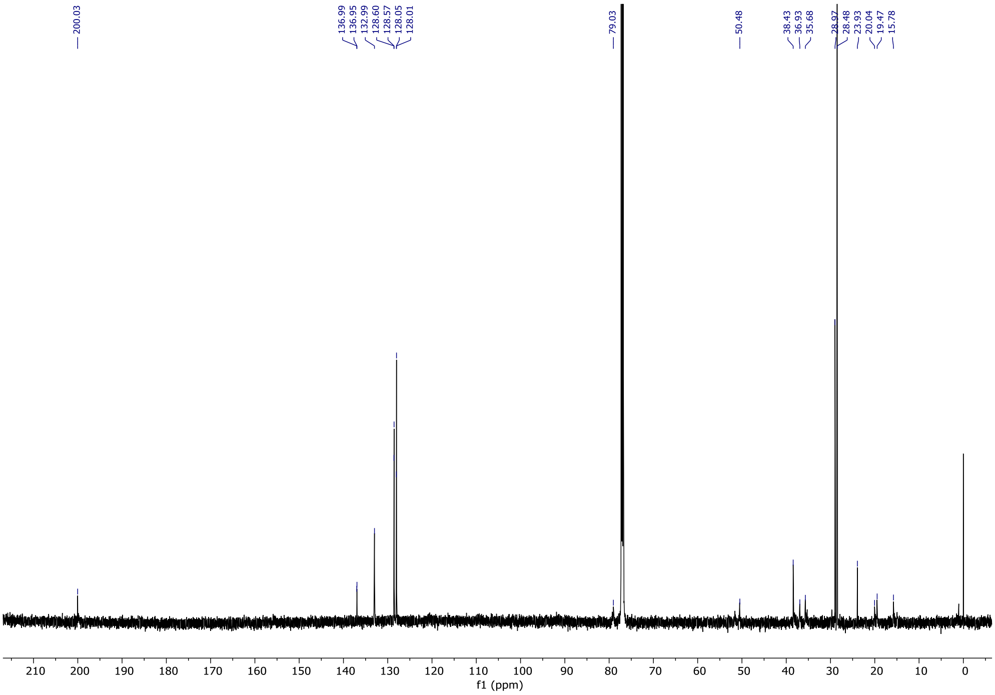

^{1}H NMR of **1m** (600 MHz, $CDCl_3$) – mixture of rotamers

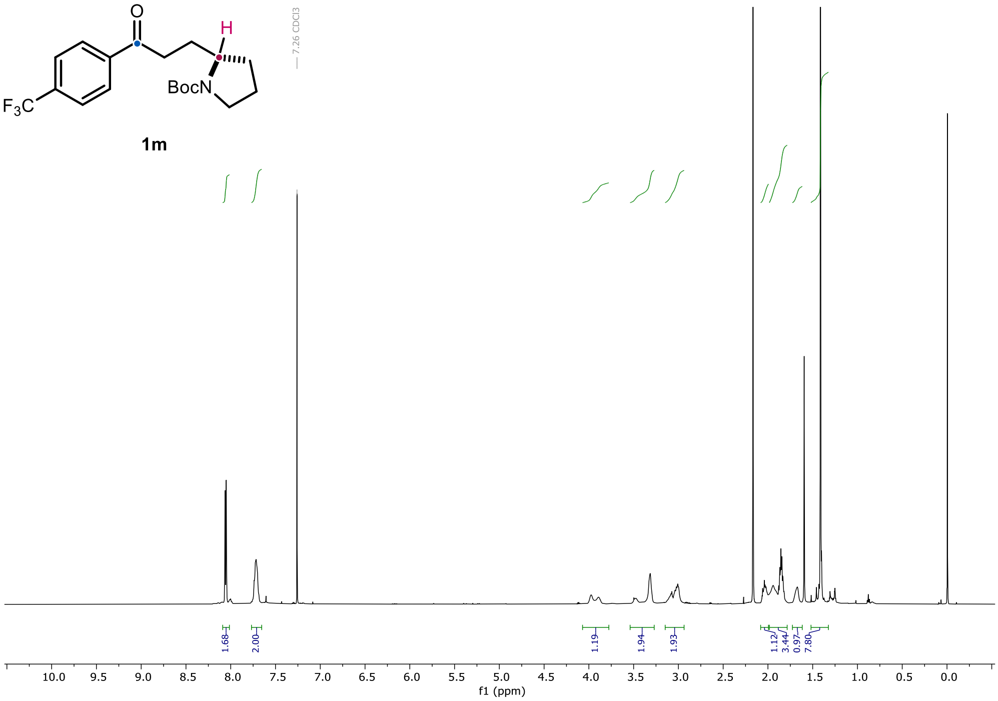

^{13}C NMR of **1m** (151 MHz, $CDCl_3$) – mixture of rotamers

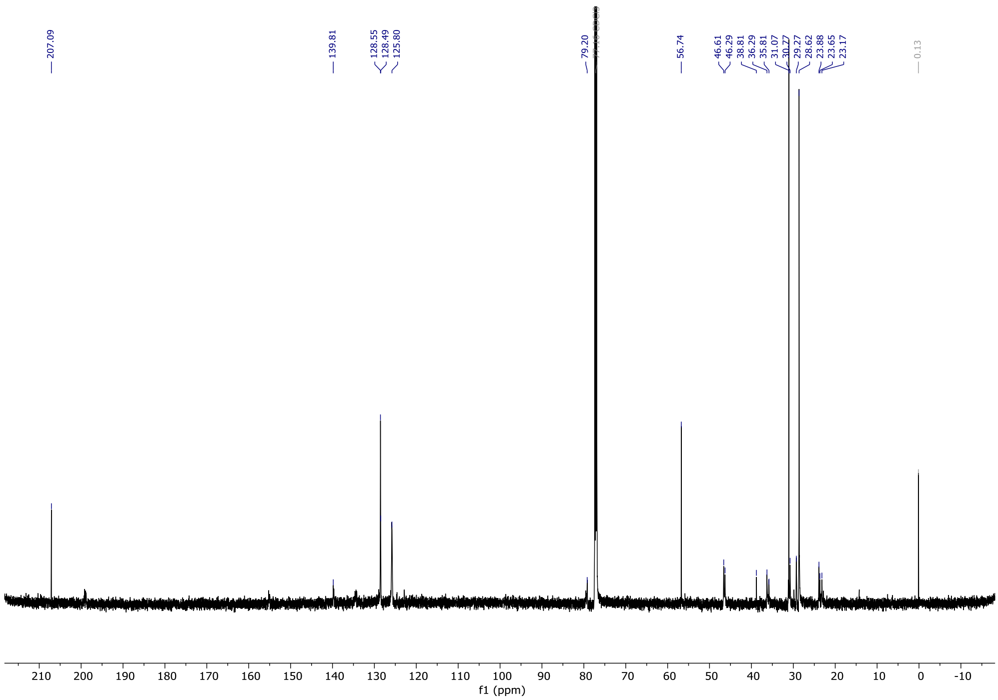

^{19}F NMR of **1m** (376 MHz, $CDCl_3$) – mixture of rotamers

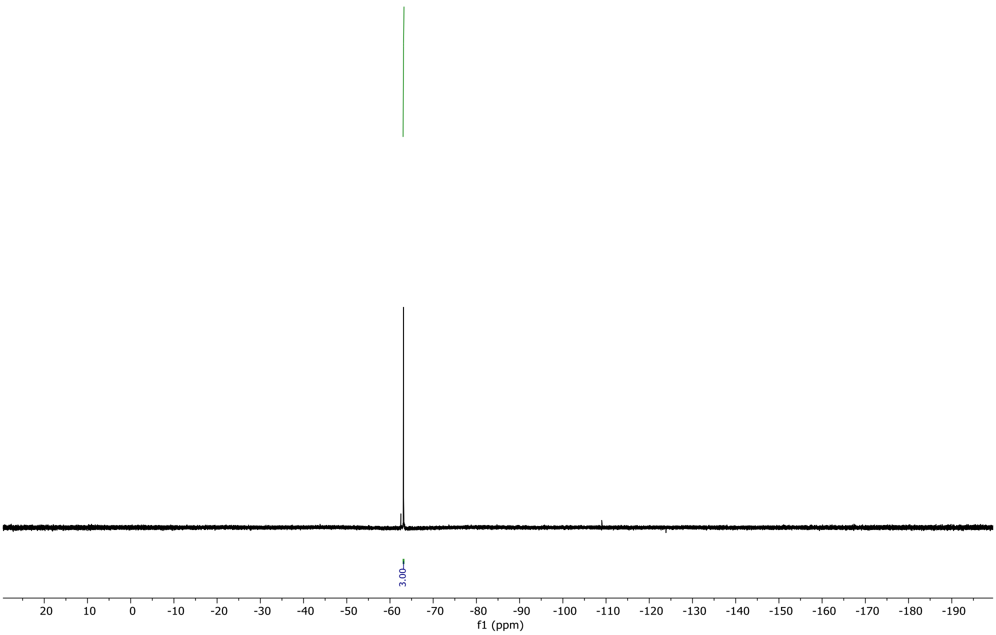

^{1}H NMR of **1n** (600 MHz, $CDCl_3$) – mixture of rotamers

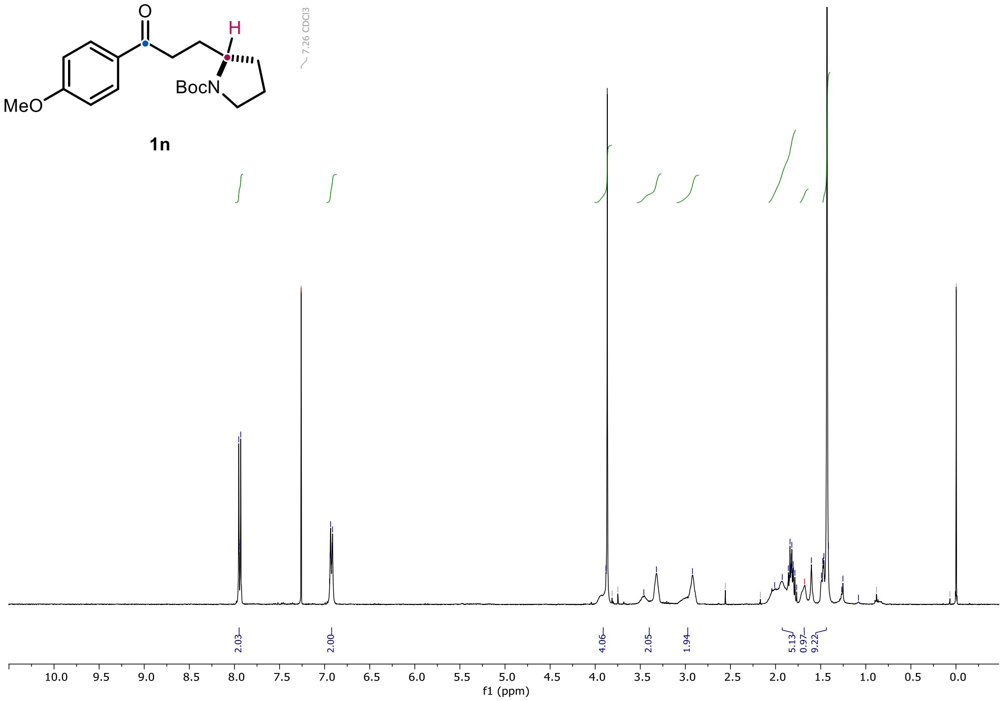

^{13}C NMR of **1n** (151 MHz, $CDCl_3$) – mixture of rotamers

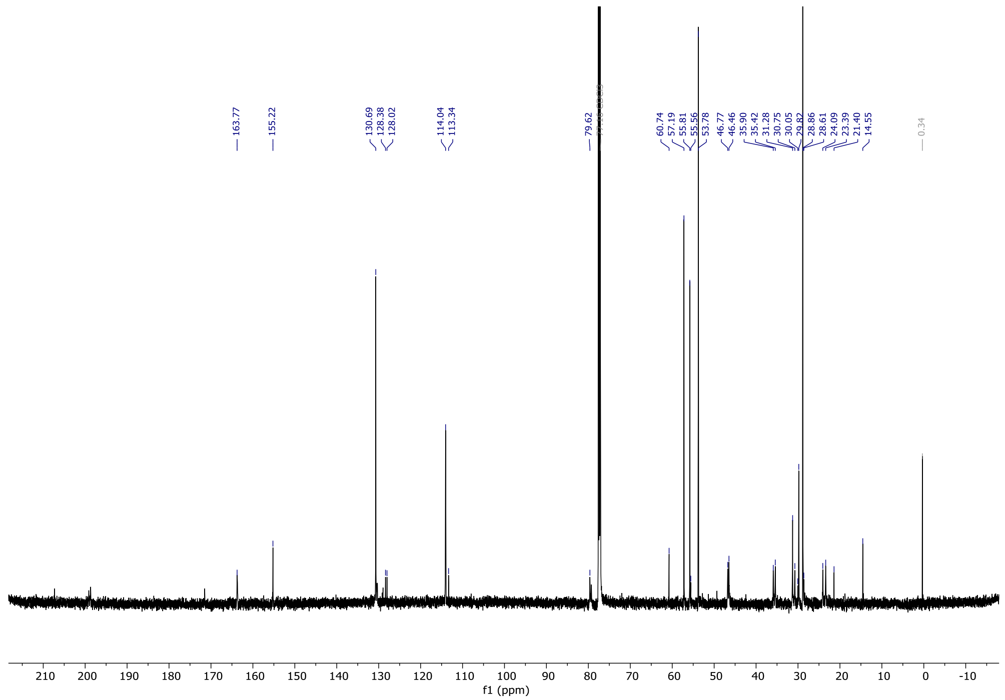

^{1}H NMR of **1o** (600 MHz, $CDCl_3$) – mixture of rotamers

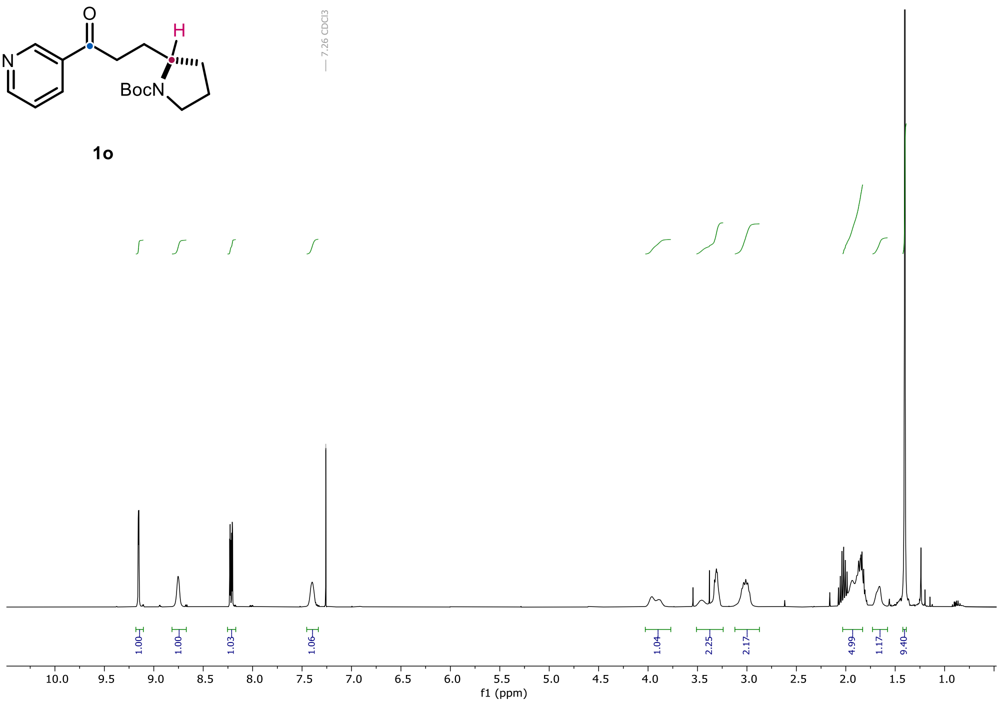

^{13}C NMR of **1o** (151 MHz, $CDCl_3$) – mixture of rotamers

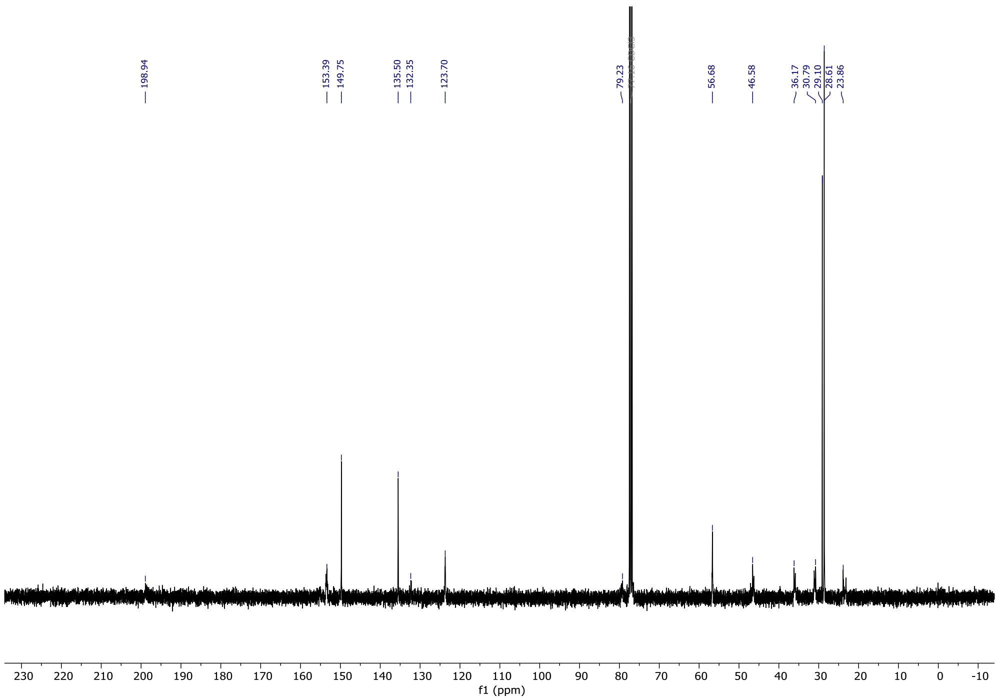

^{1}H NMR of **2a** (600 MHz, $CDCl_3$)

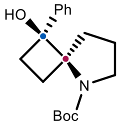

2a

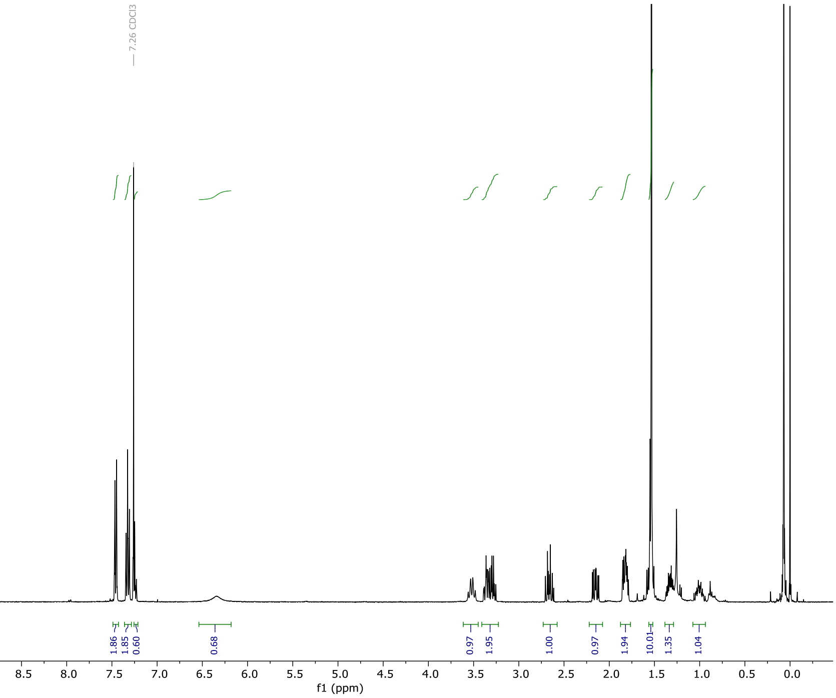

^{13}C NMR of **2a** (151 MHz, $CDCl_3$)

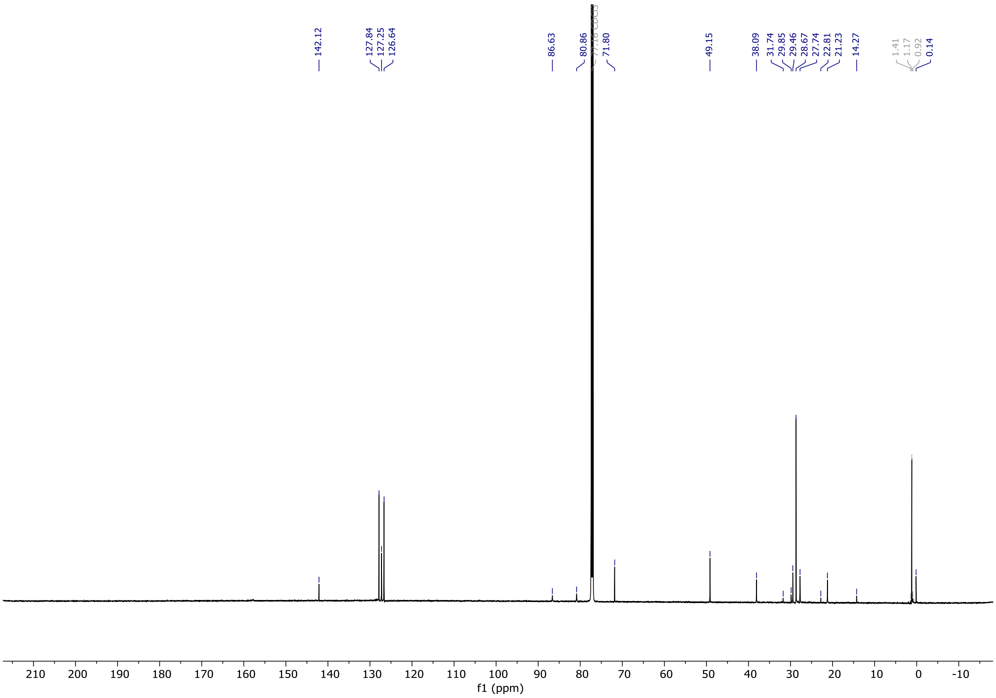

^{1}H NMR of **2b** (600 MHz, $CDCl_3$)

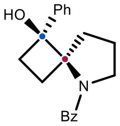

2b

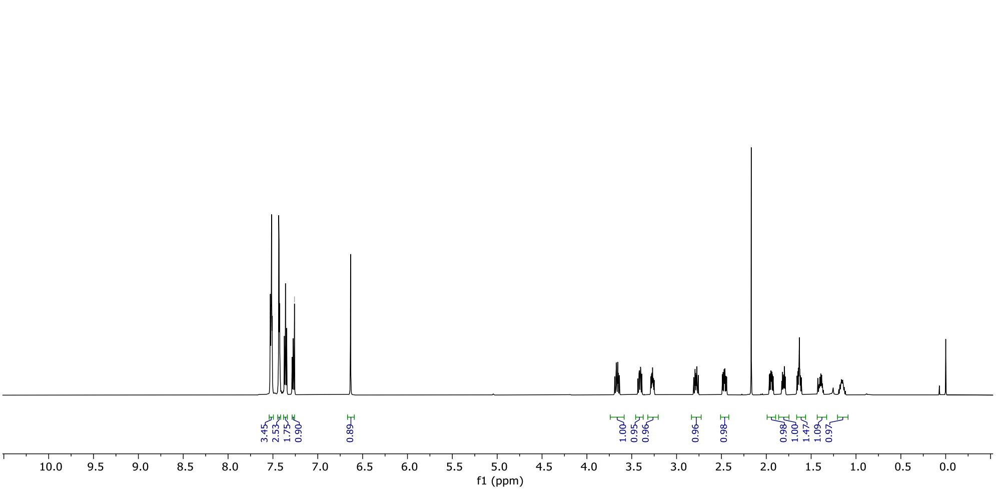

^{13}C NMR of **2b** (151 MHz, $CDCl_3$)

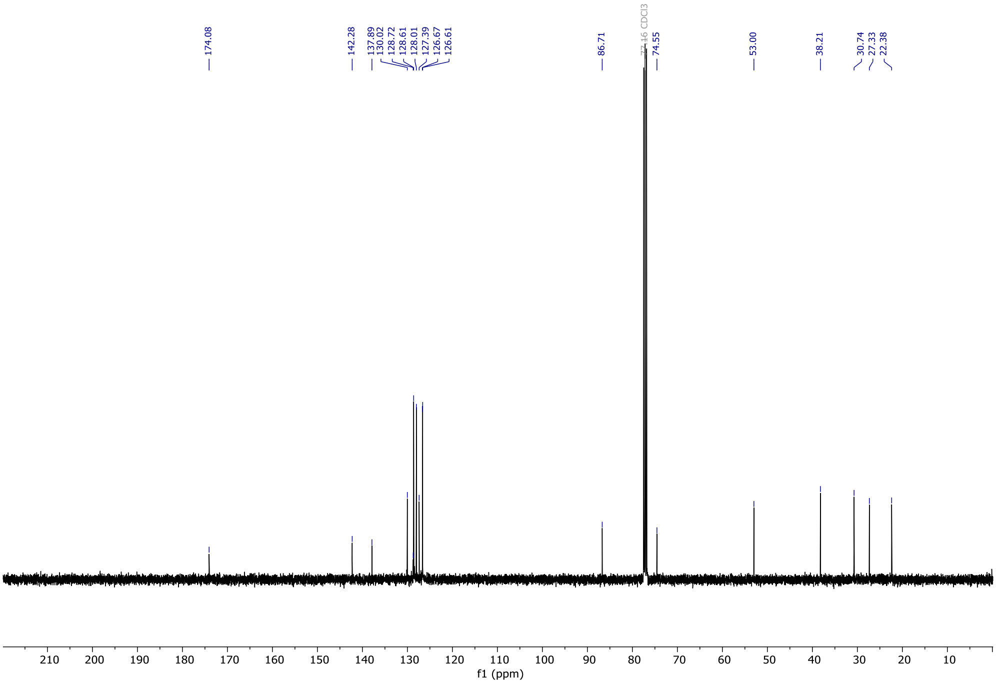

^{1}H NMR of **2c** (600 MHz, $CDCl_3$)

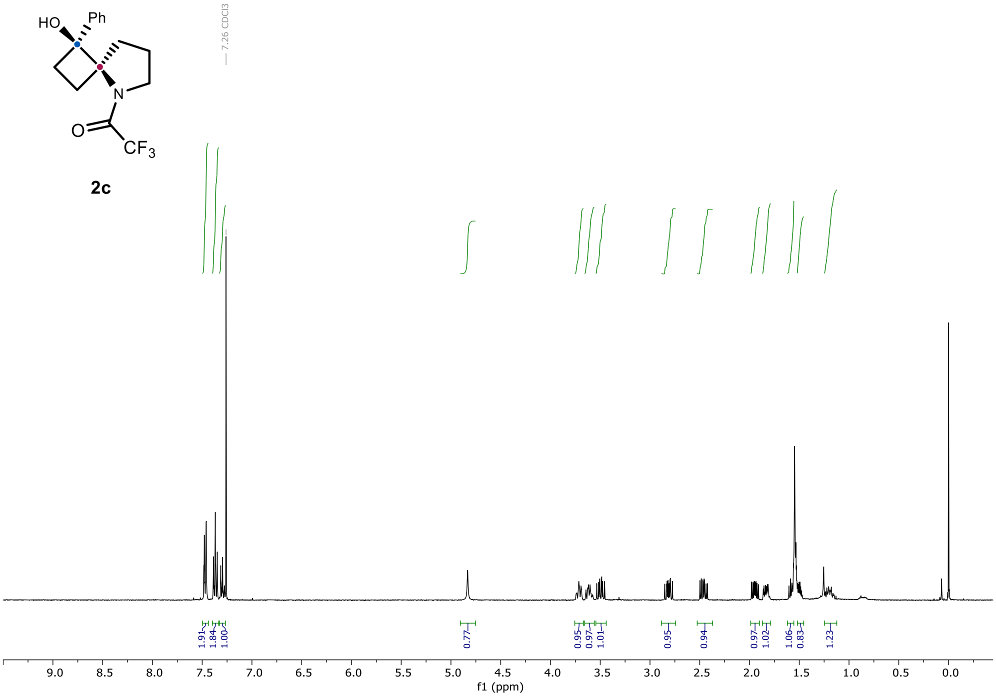

^{13}C NMR of **2c** (151 MHz, $CDCl_3$)

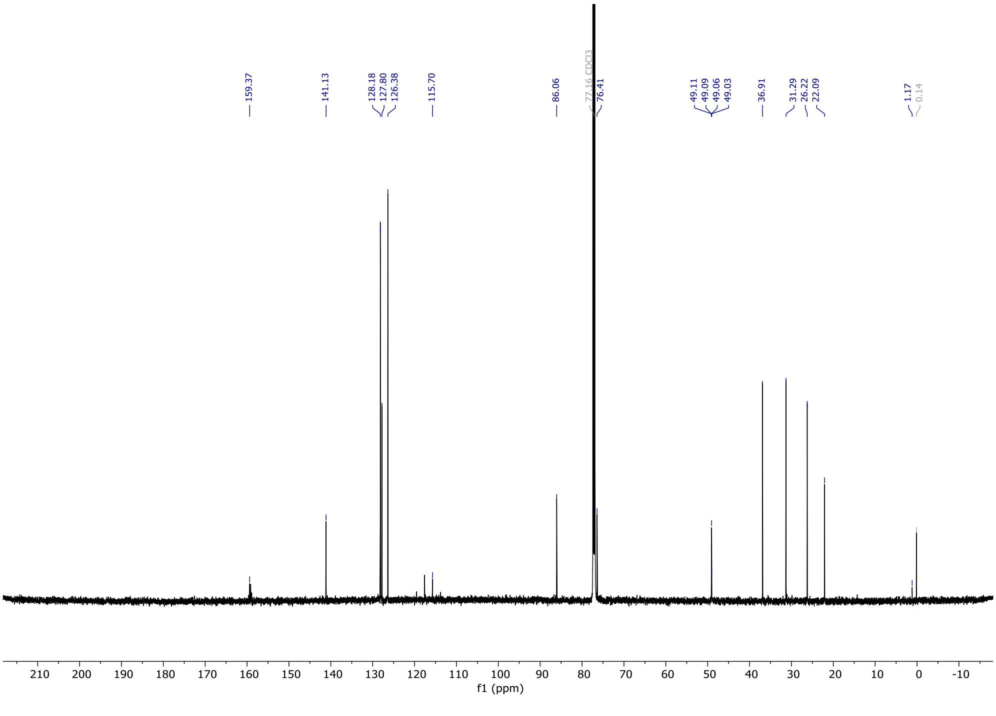

^{19}F NMR of **2c** (376 MHz, $CDCl_3$)

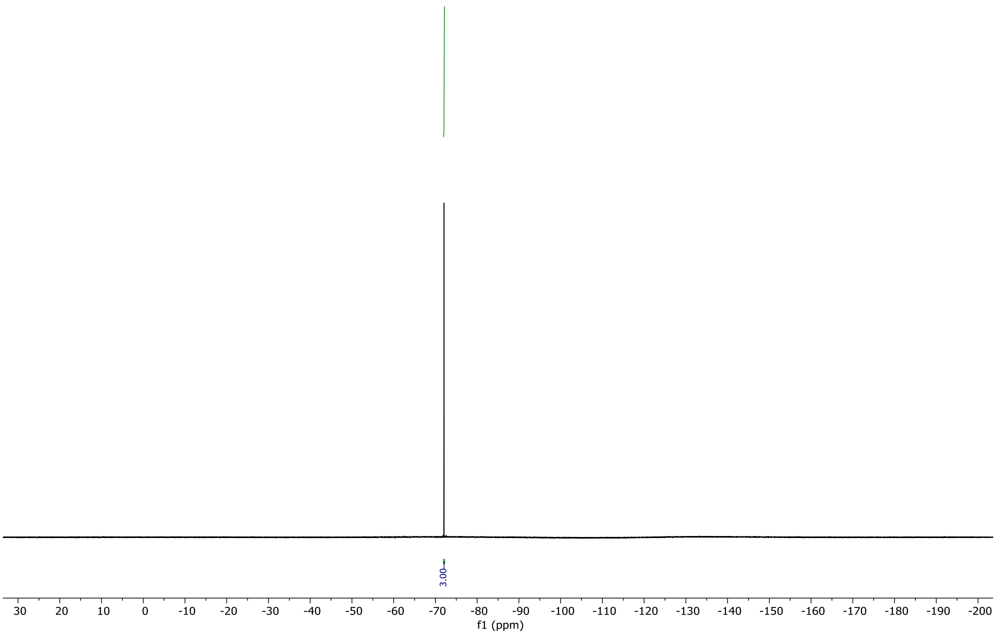

^{1}H NMR of **2e** (600 MHz, $CDCl_3$)

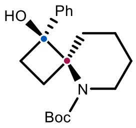

2e

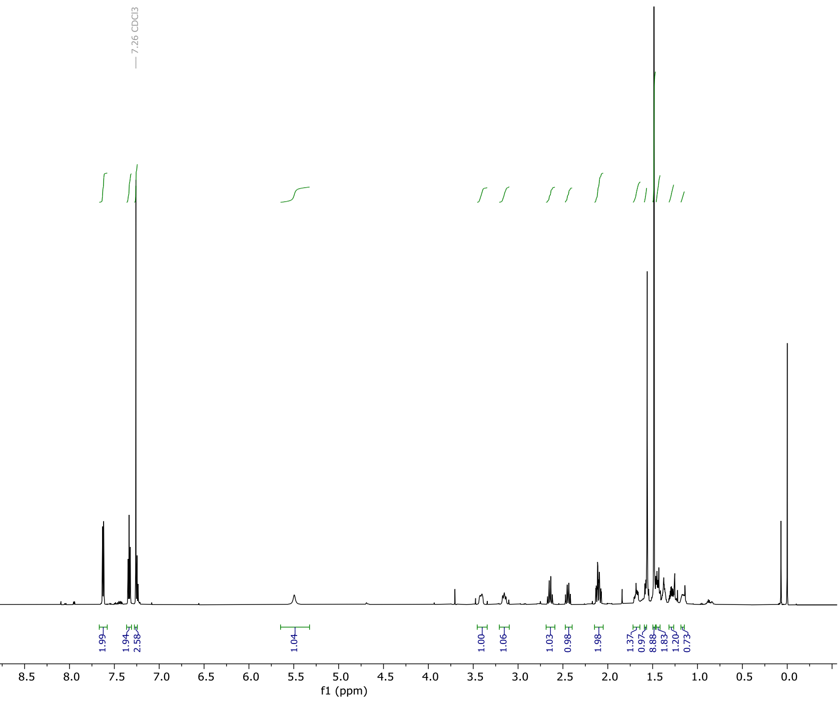

^{13}C NMR of **2e** (151 MHz, $CDCl_3$)

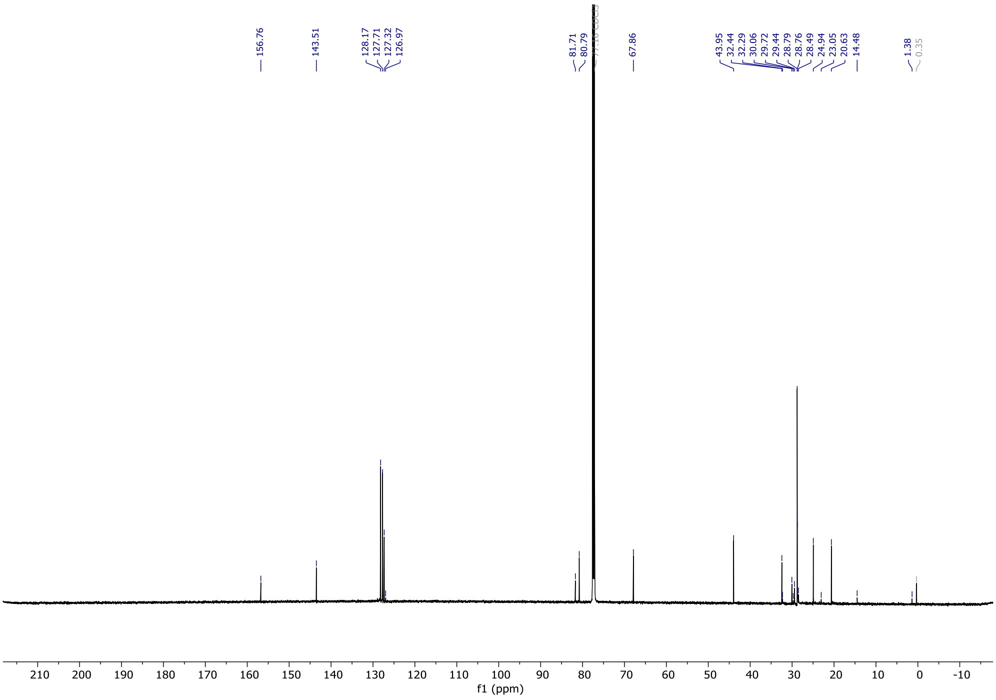

^{1}H NMR of **2f** (600 MHz, $CDCl_3$)

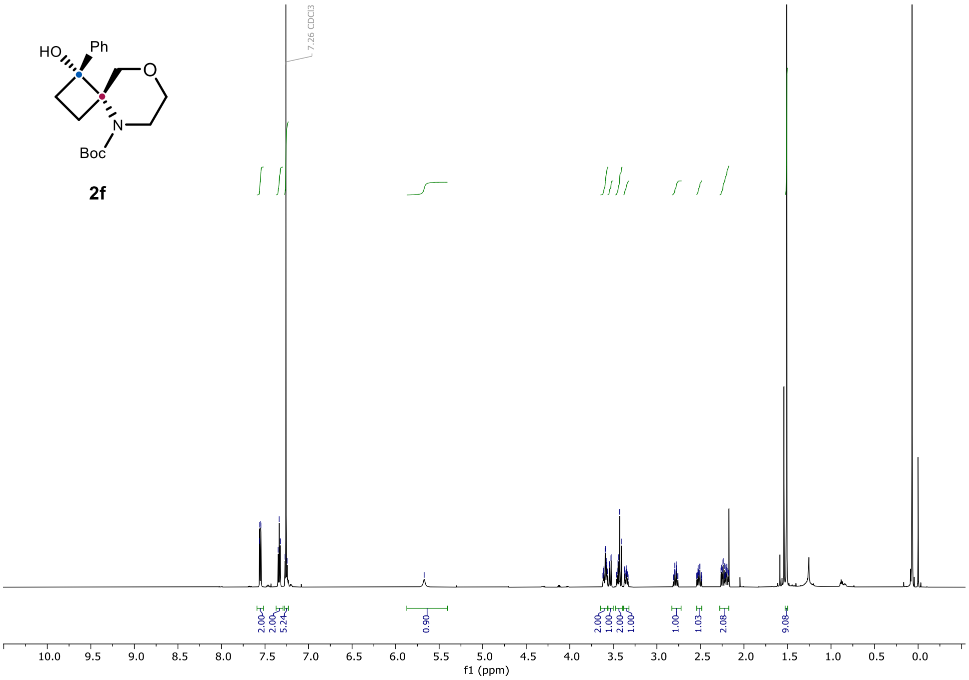

2f

^{13}C NMR of **2f** (151 MHz, $CDCl_3$)

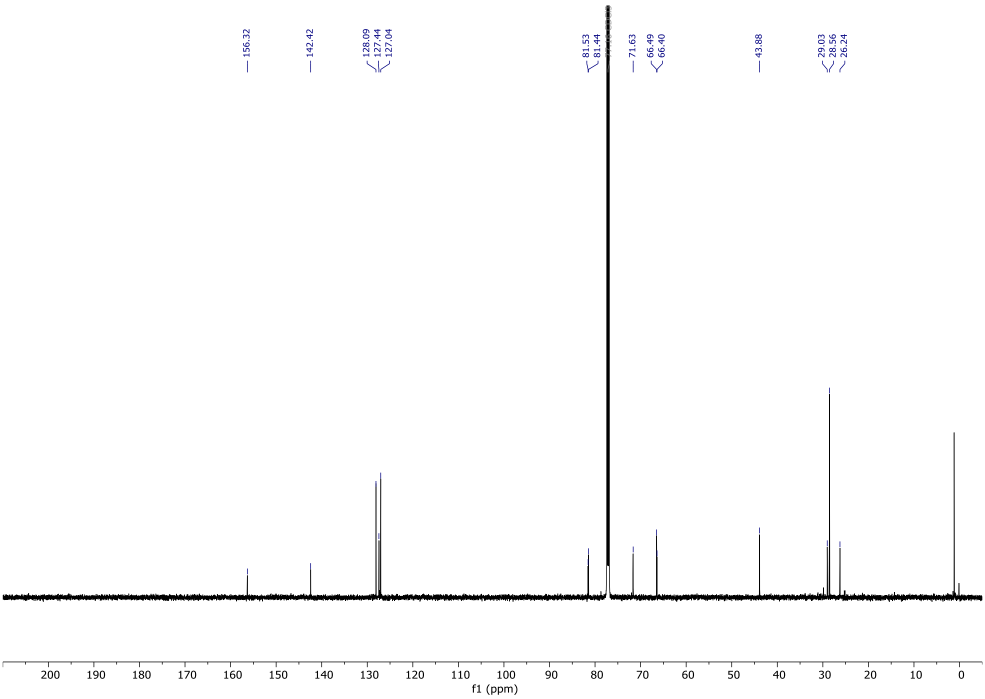

^{1}H NMR of **2g** (600 MHz, $CDCl_3$)

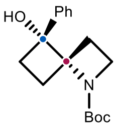

2g

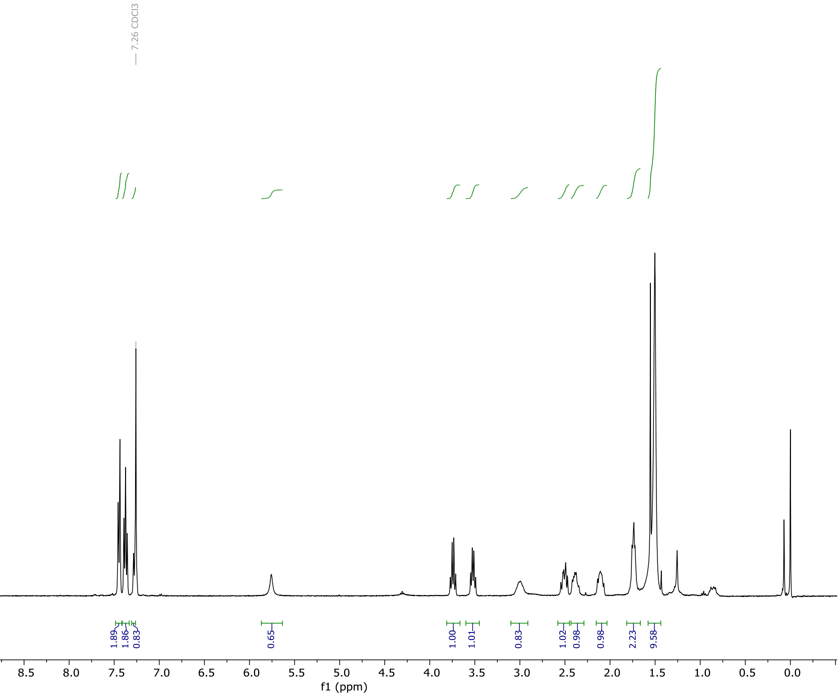

^{13}C NMR of **2g** (151 MHz, $CDCl_3$)

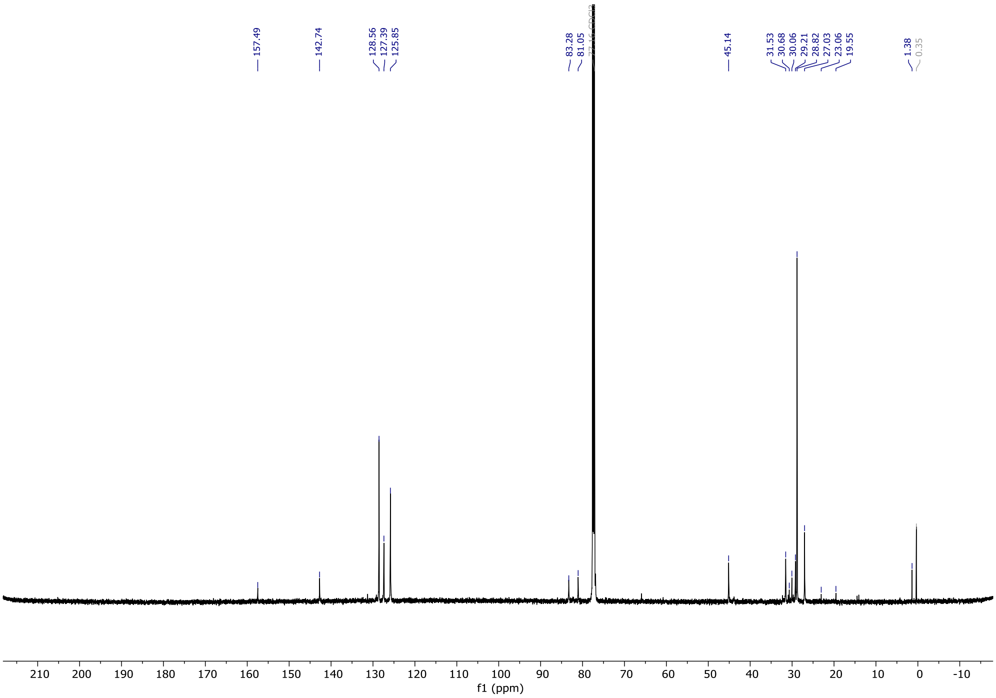

^{1}H NMR of **2h** (600 MHz, $CDCl_3$)

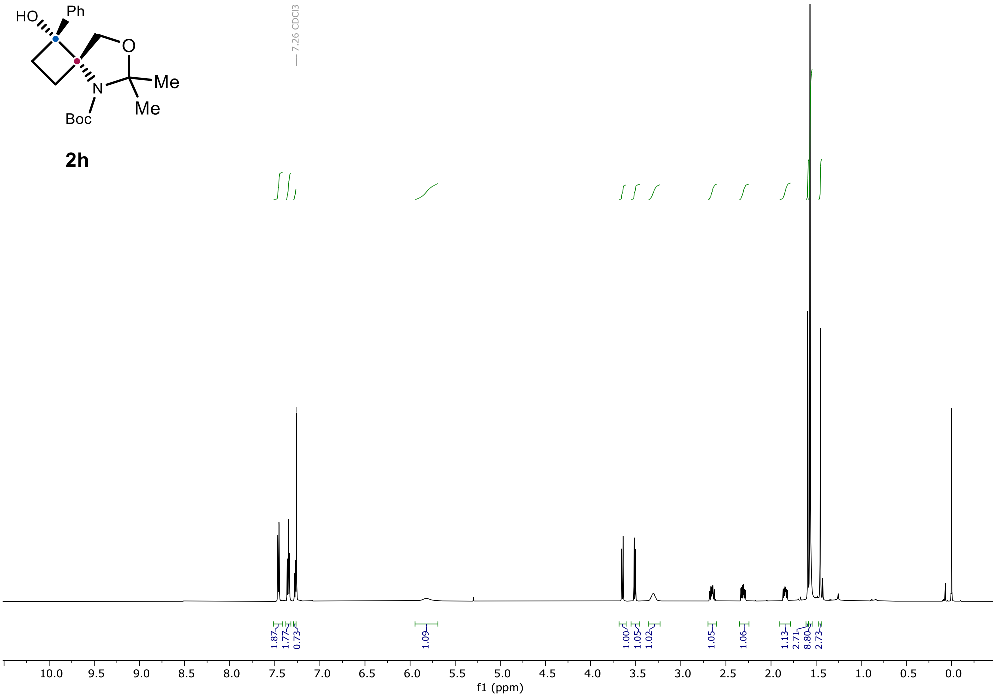

^{13}C NMR of **2h** (151 MHz, $CDCl_3$)

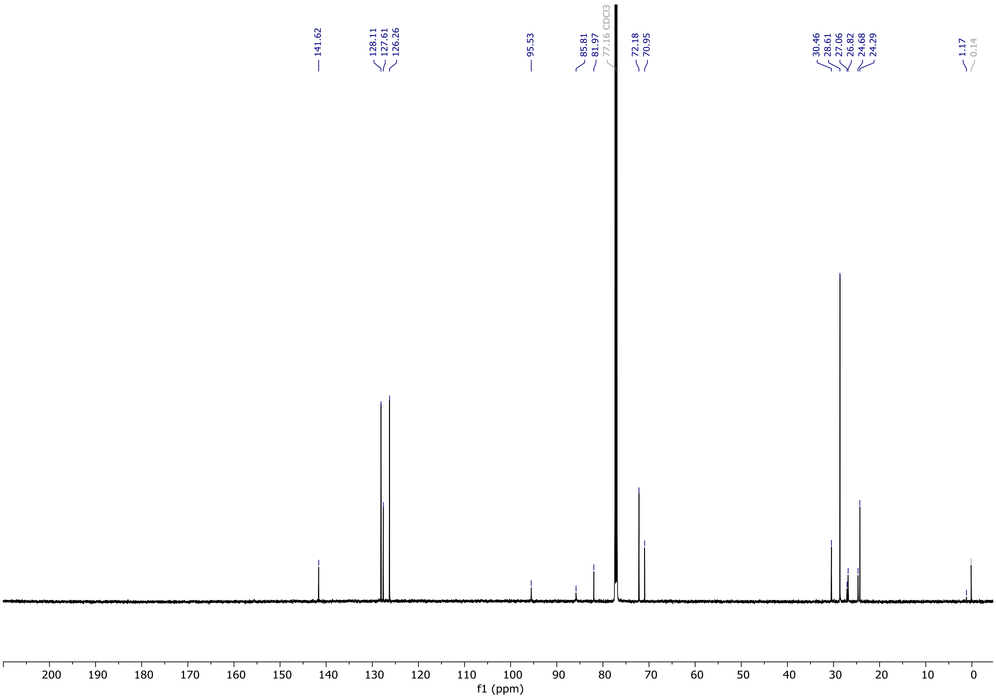

^{1}H NMR of **2i** (600 MHz, $CDCl_3$)

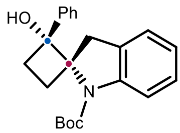

2i

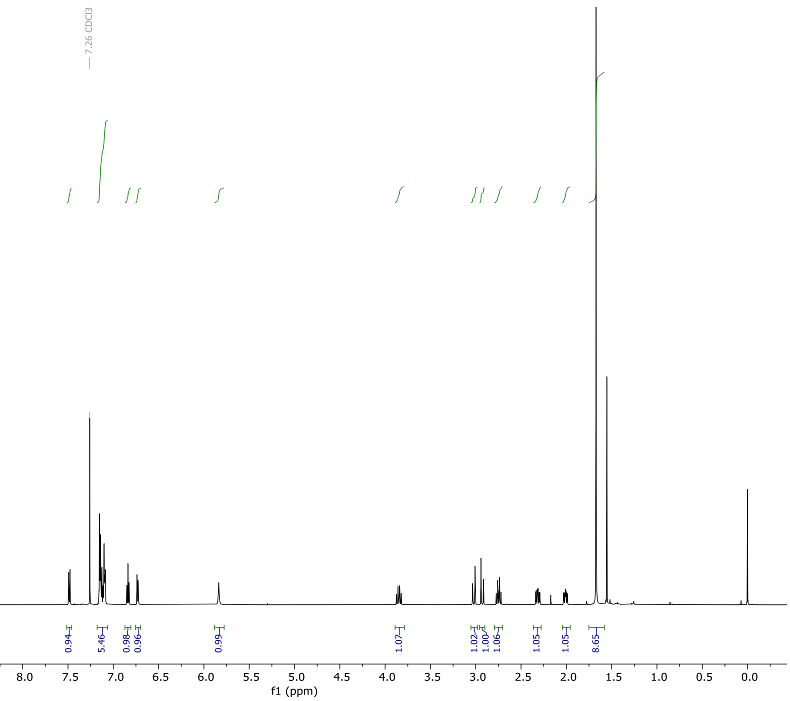

^{13}C NMR of **2i** (151 MHz, $CDCl_3$)

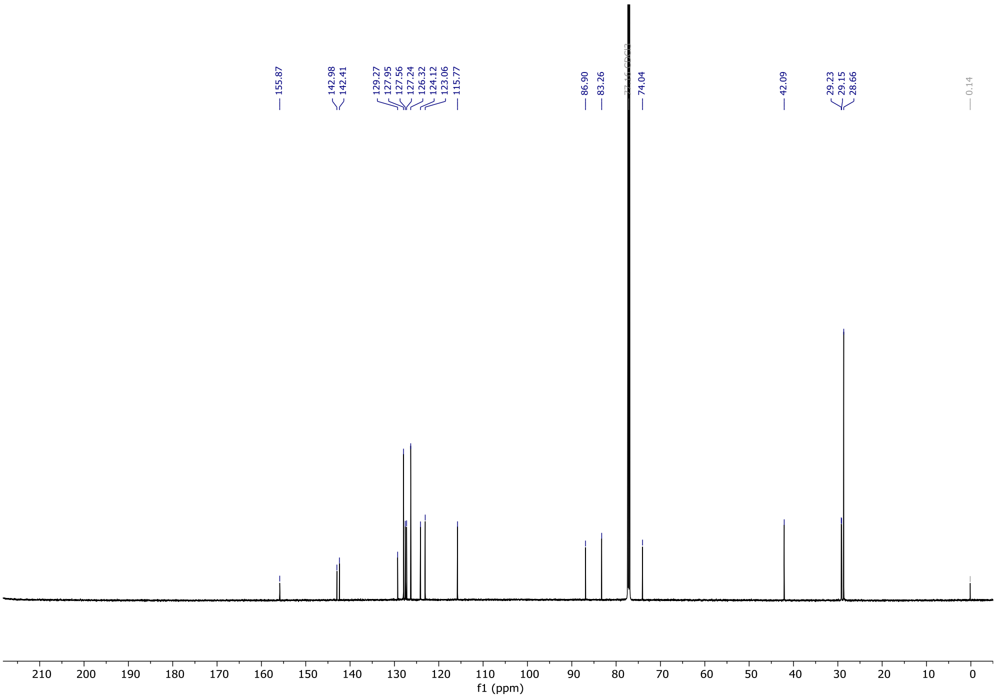

^{1}H NMR of **2j** (600 MHz, $CDCl_3$)

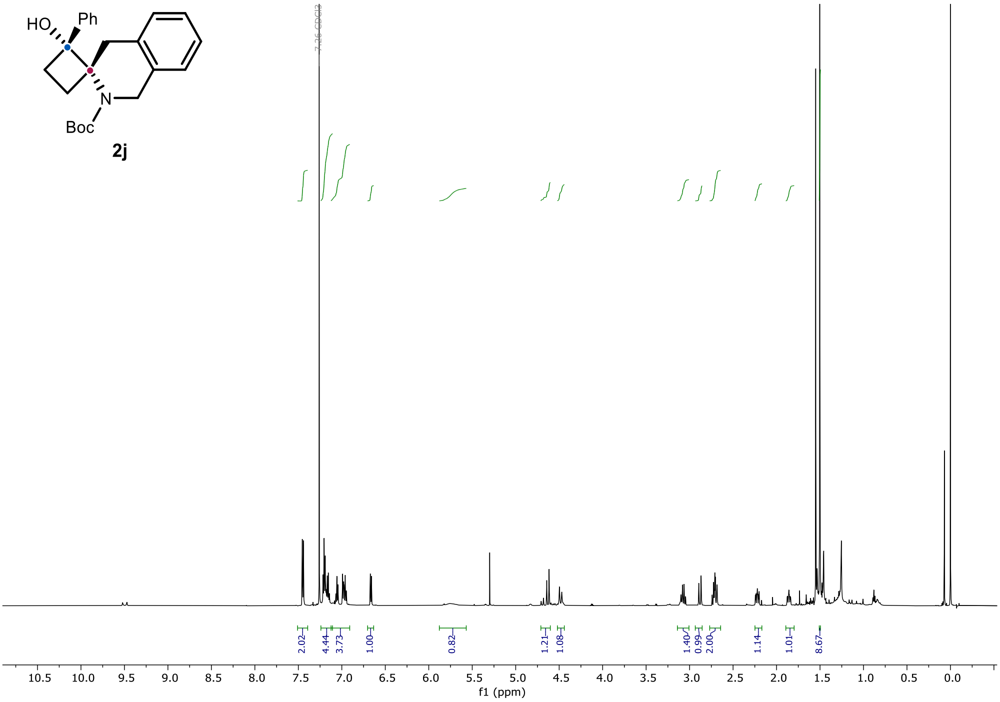

^{13}C NMR of **2j** (151 MHz, $CDCl_3$)

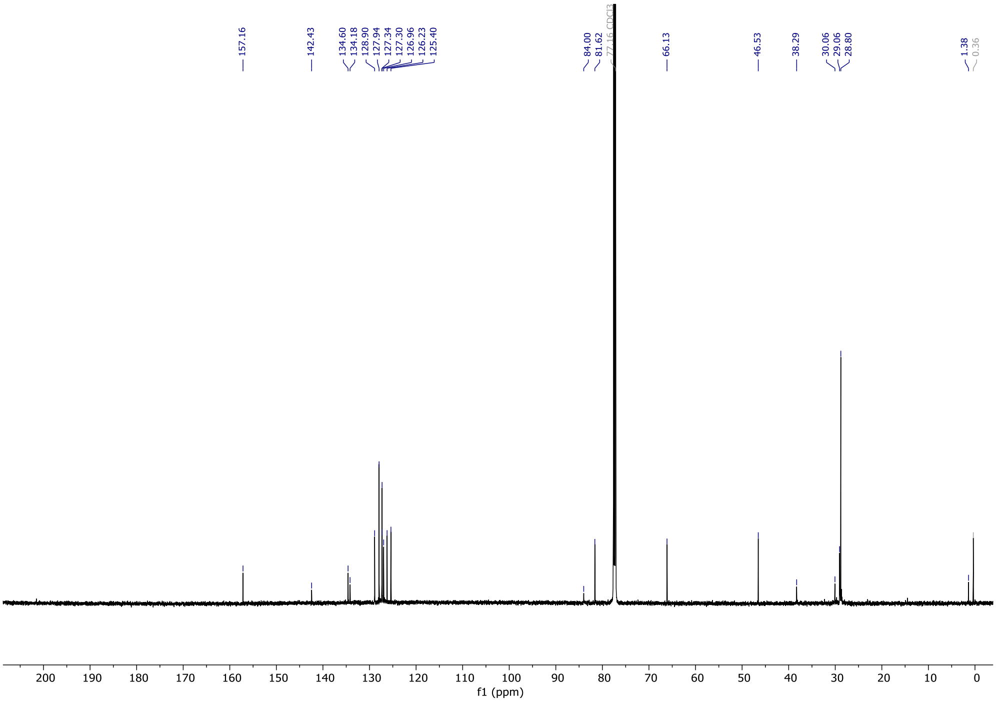

^{1}H NMR of **2k** (600 MHz, $CDCl_3$)

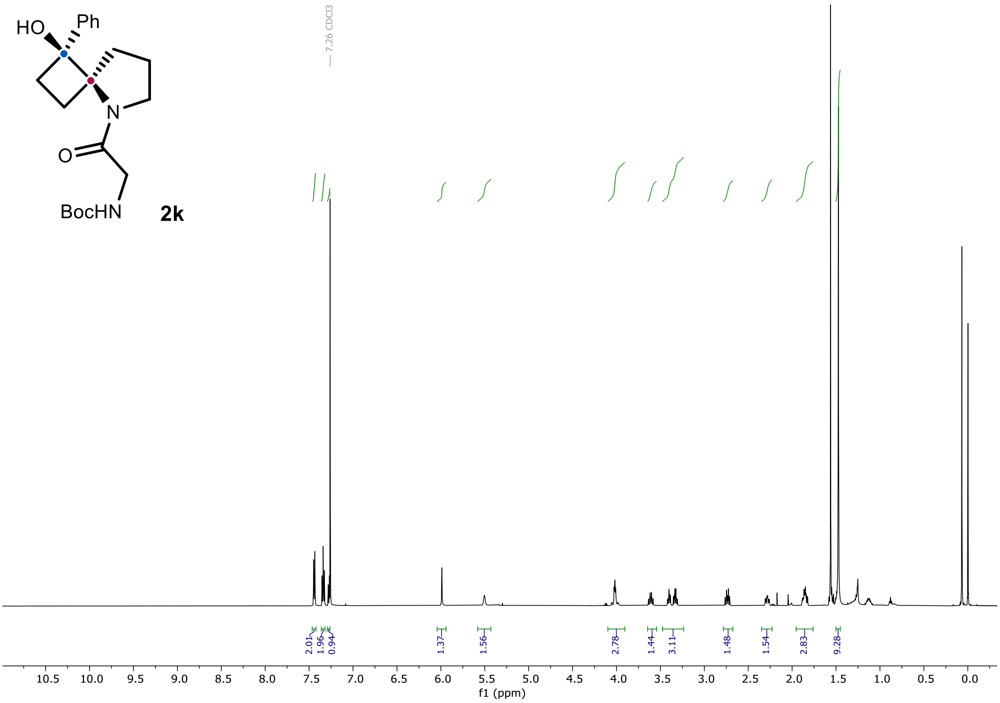

^{13}C NMR of **2k** (151 MHz, $CDCl_3$)

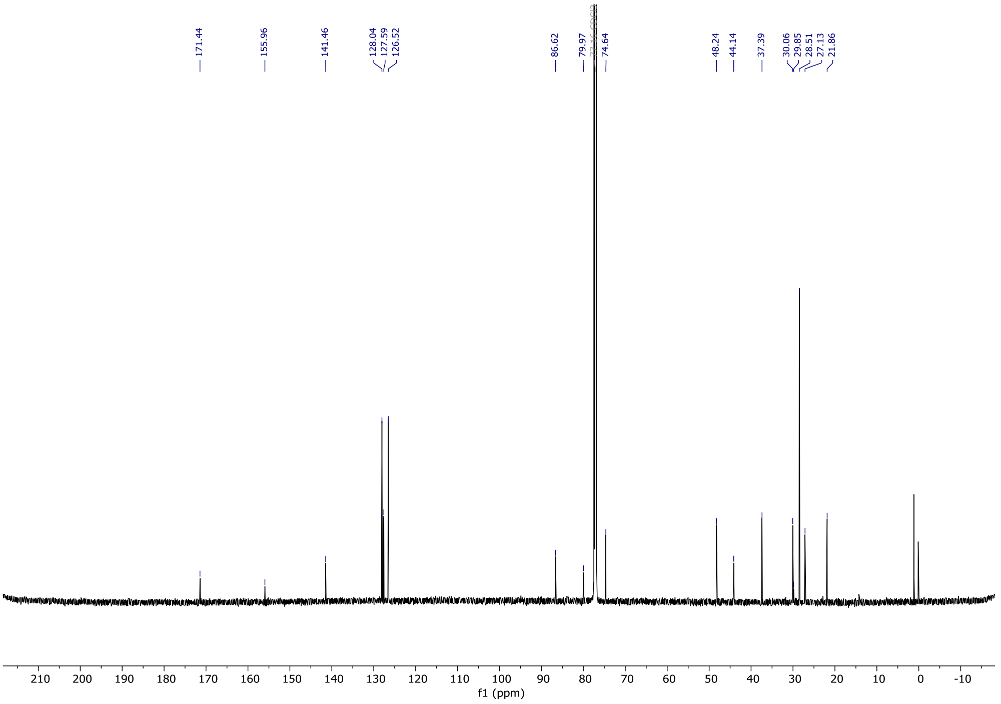

^{1}H NMR of **2l** (600 MHz, $CDCl_3$) – mixture of rotamers

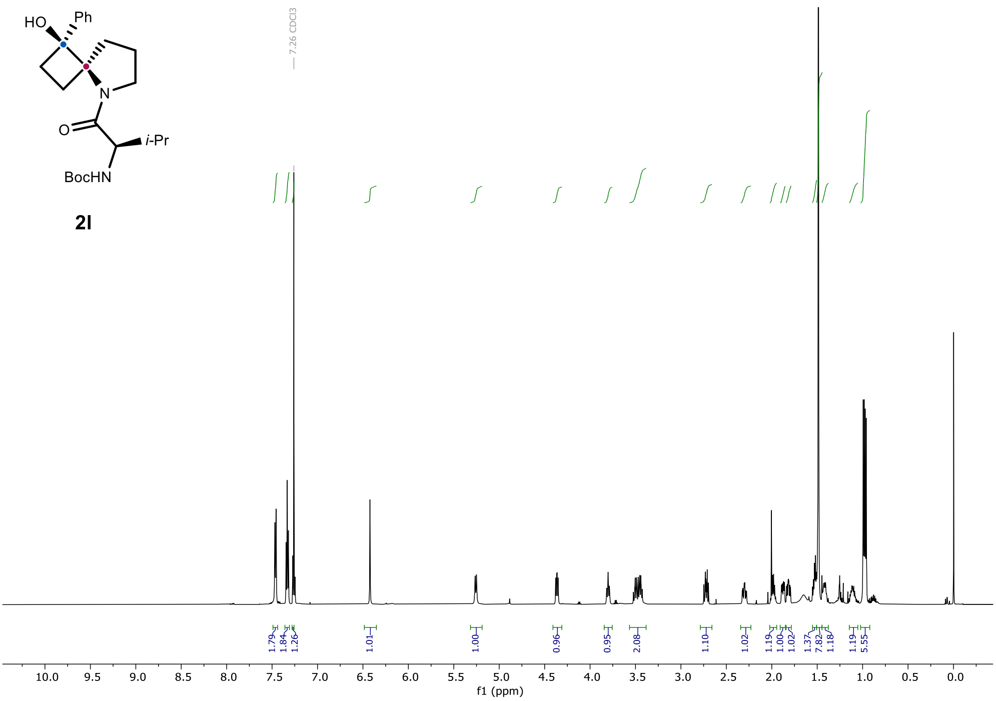

^{13}C NMR of **2l** (151 MHz, $CDCl_3$) – mixture of rotamers

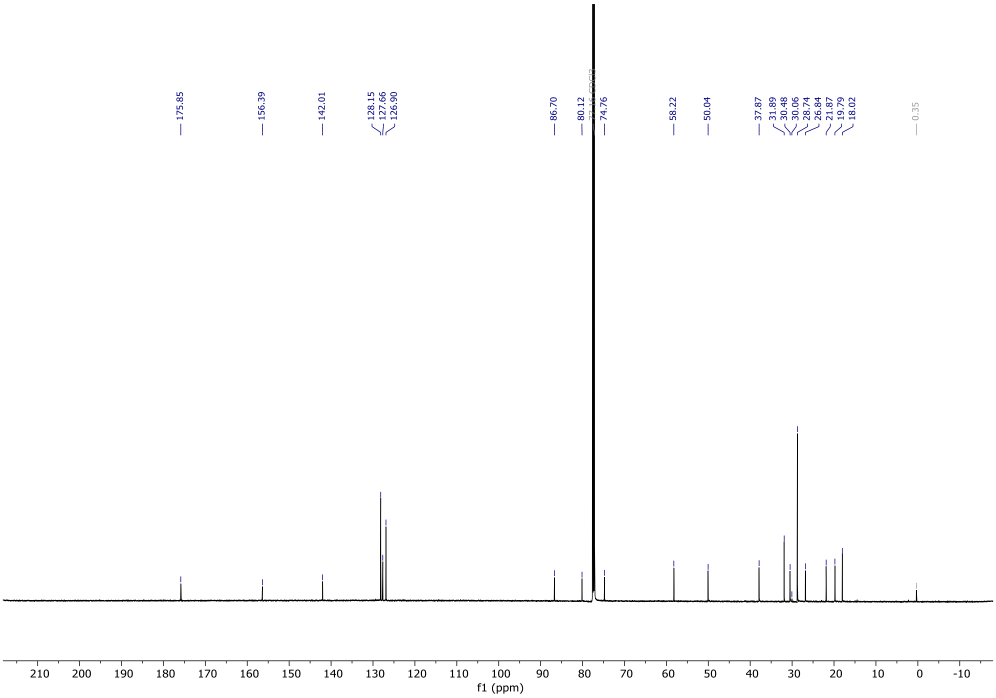

^{1}H NMR of **2m** (600 MHz, $CDCl_3$)

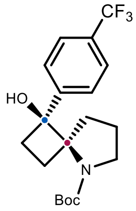

2m

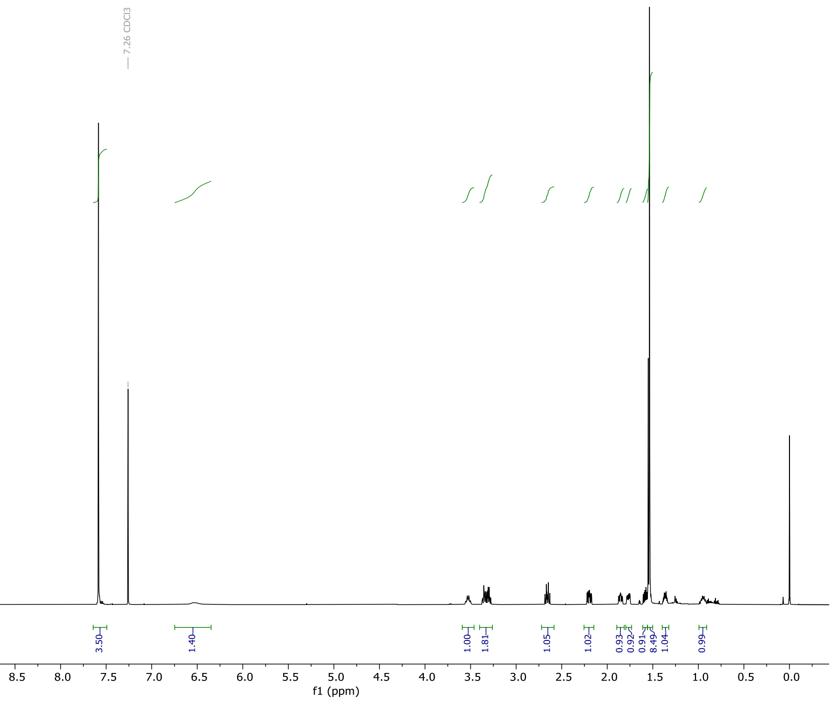

^{13}C NMR of **2m** (151 MHz, $CDCl_3$)

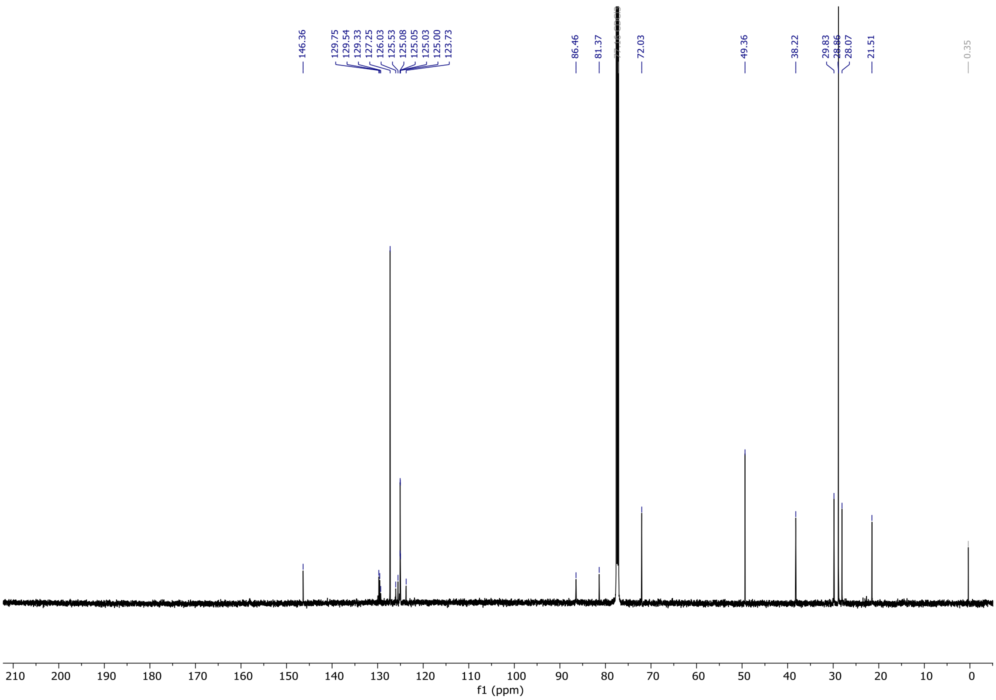

^{19}F NMR of **2m** (151 MHz, $CDCl_3$)

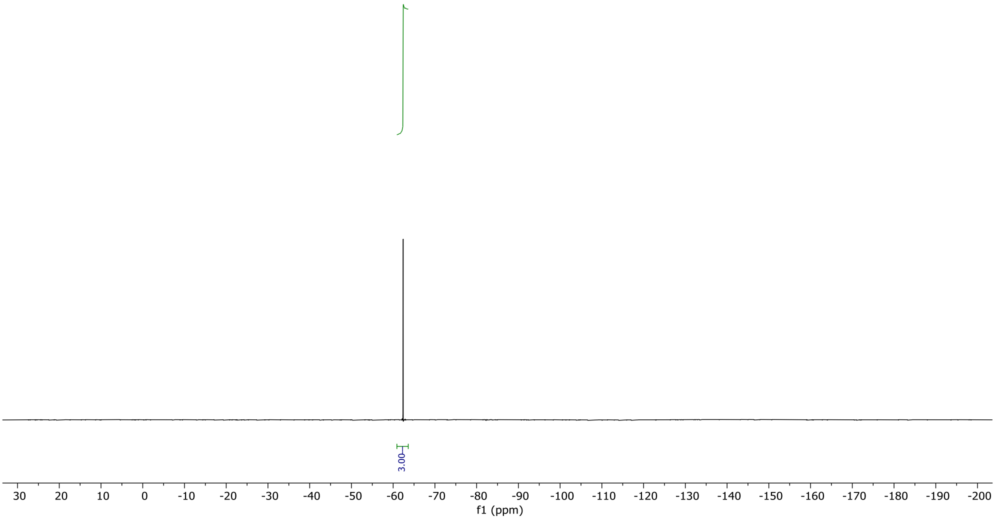

^{1}H NMR of **2n** (600 MHz, $CDCl_3$)

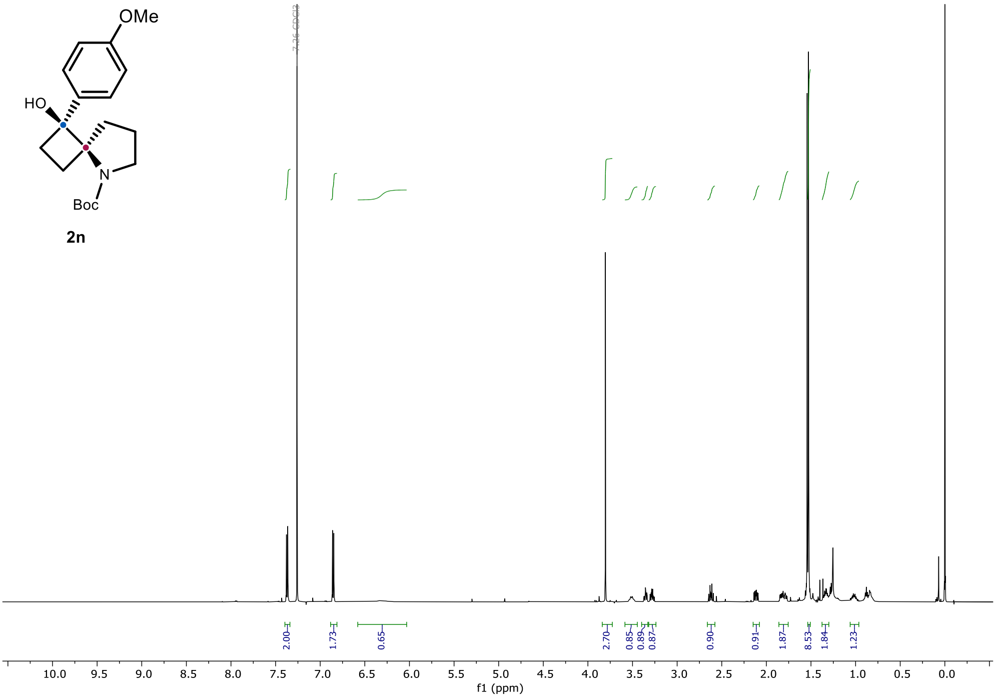

^{13}C NMR of **2n** (151 MHz, $CDCl_3$)

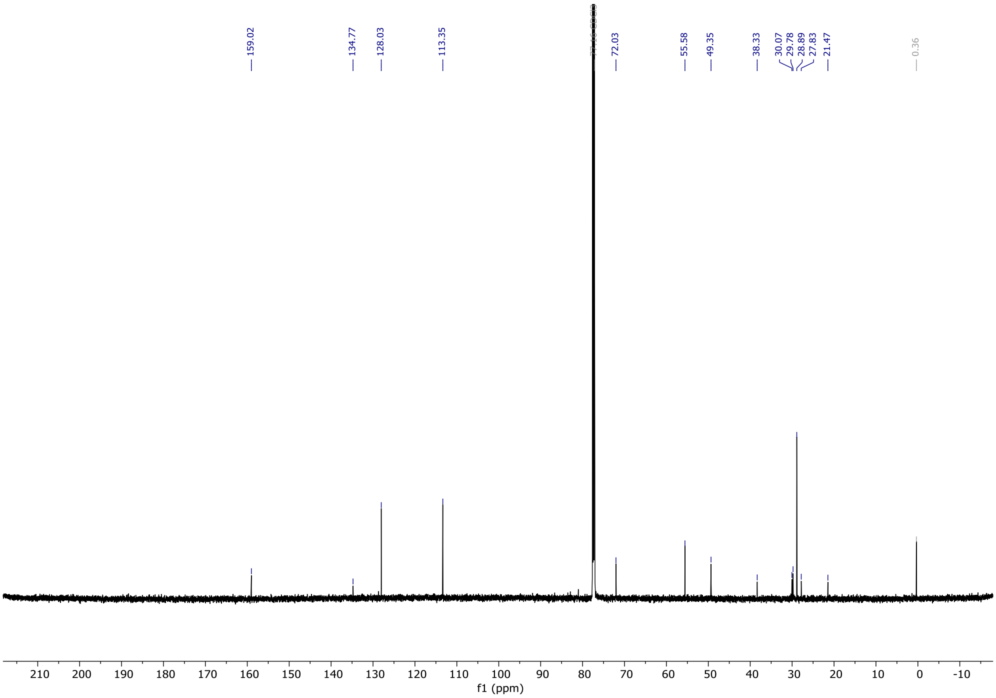

^{1}H NMR of **2o** (600 MHz, $CDCl_3$)

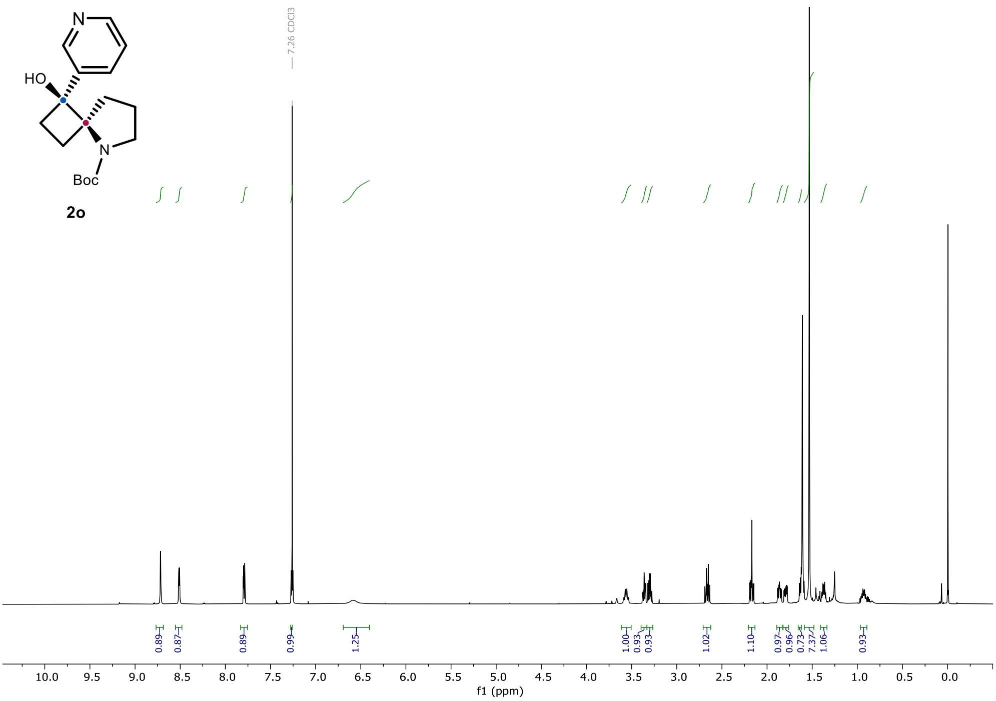

^{13}C NMR of **2o** (151 MHz, $CDCl_3$)

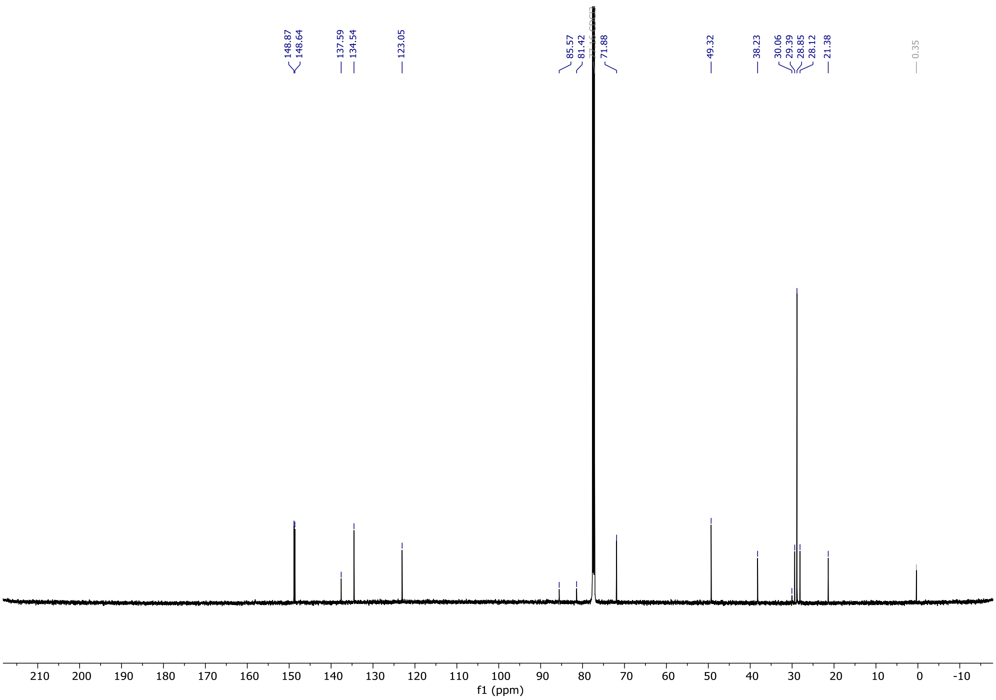

^{1}H NMR of **6b** (600 MHz, $CDCl_3$)

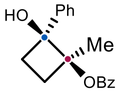

6b

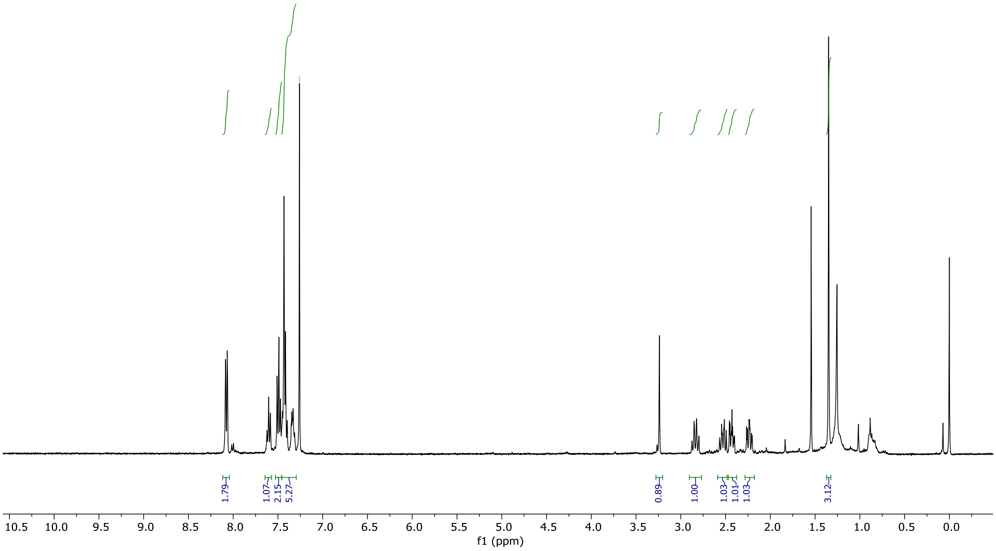

^{13}C NMR of **6b** (151 MHz, $CDCl_3$)

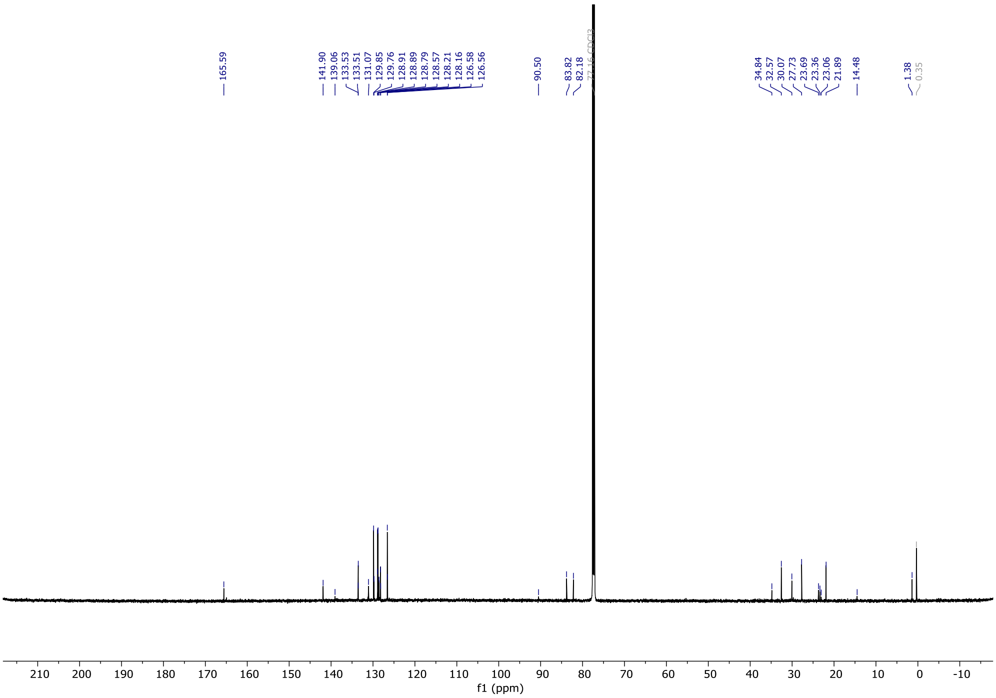

^{1}H NMR of **6c** (600 MHz, $CDCl_3$)

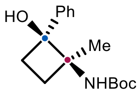

6c

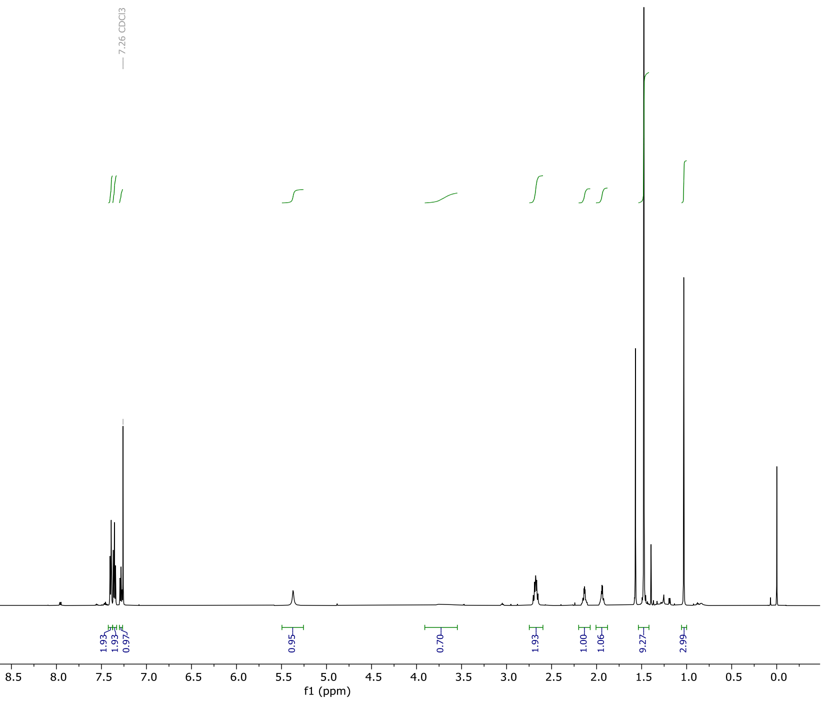

^{13}C NMR of **6c** (151 MHz, $CDCl_3$)

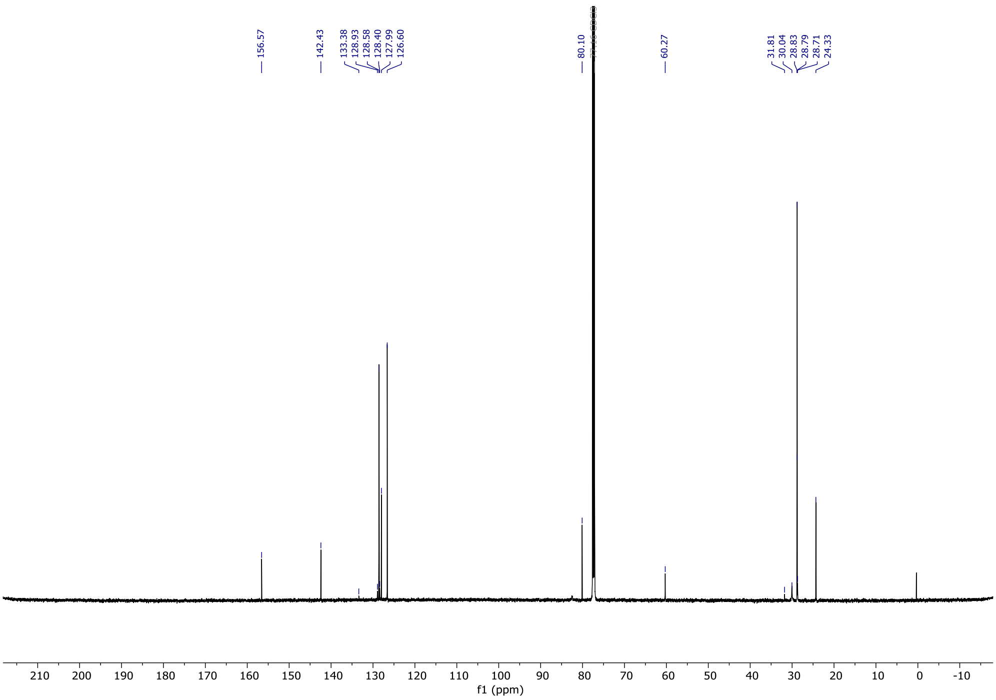

^{1}H NMR of **6d** (600 MHz, $CDCl_3$)

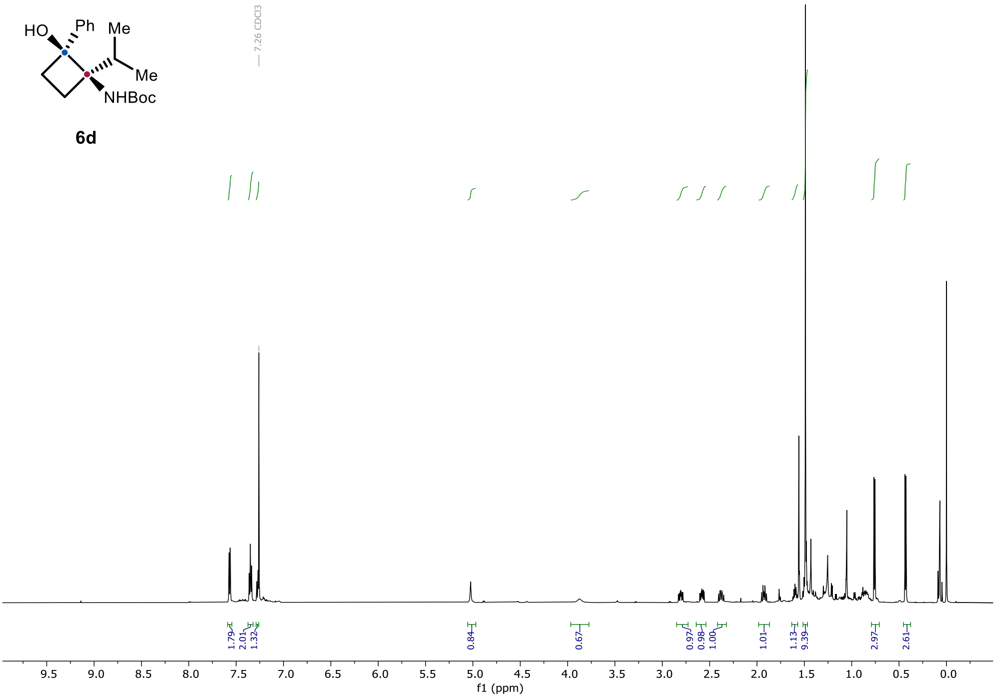

^{13}C NMR of **6d** (151 MHz, $CDCl_3$)

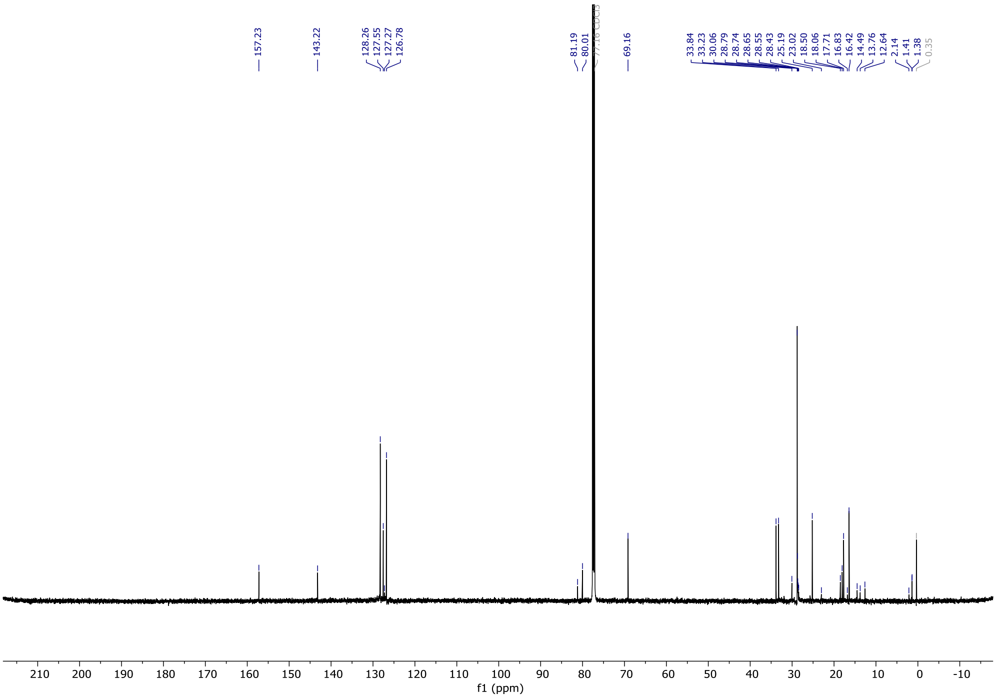

^{1}H NMR of **6e** (600 MHz, $CDCl_3$)

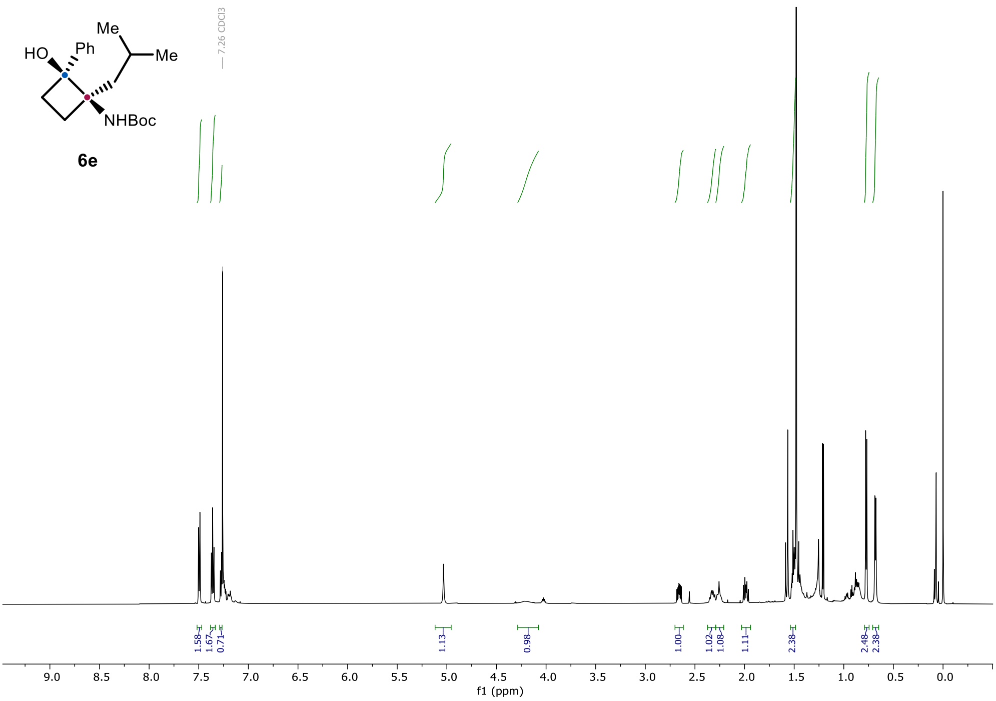

^{13}C NMR of **6e** (151 MHz, $CDCl_3$)

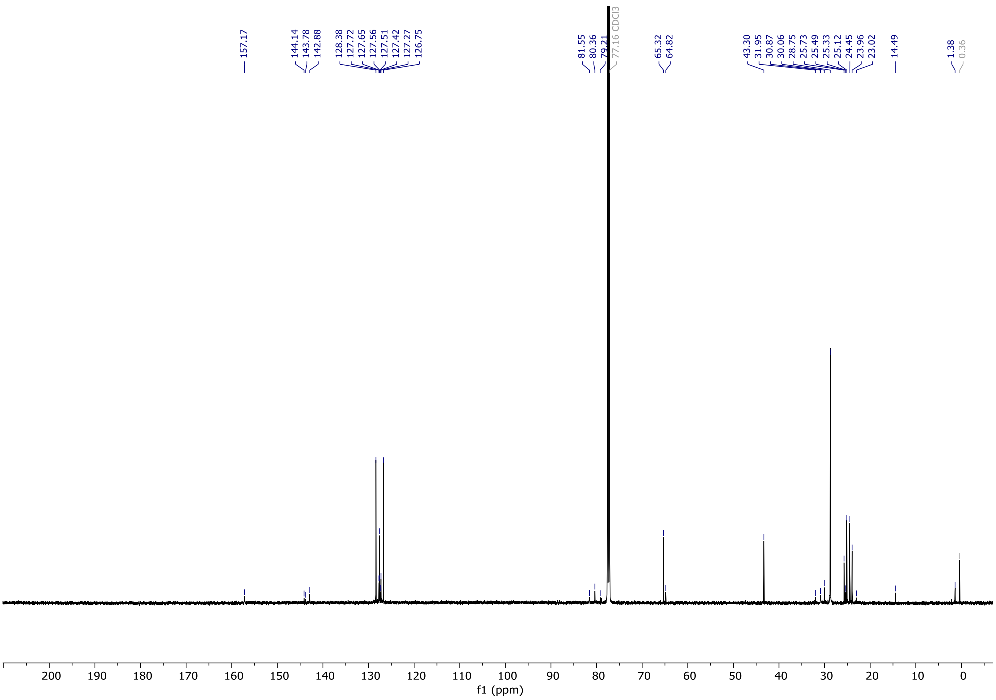

^{1}H NMR of **6f** (600 MHz, $CDCl_3$)

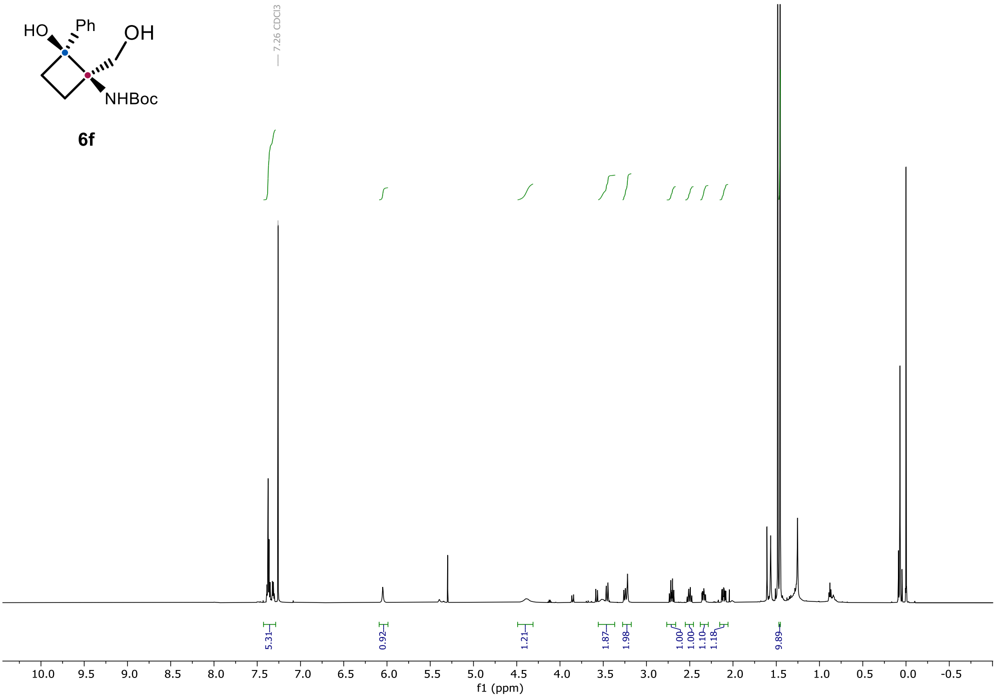

^{13}C NMR of **6f** (151 MHz, $CDCl_3$)

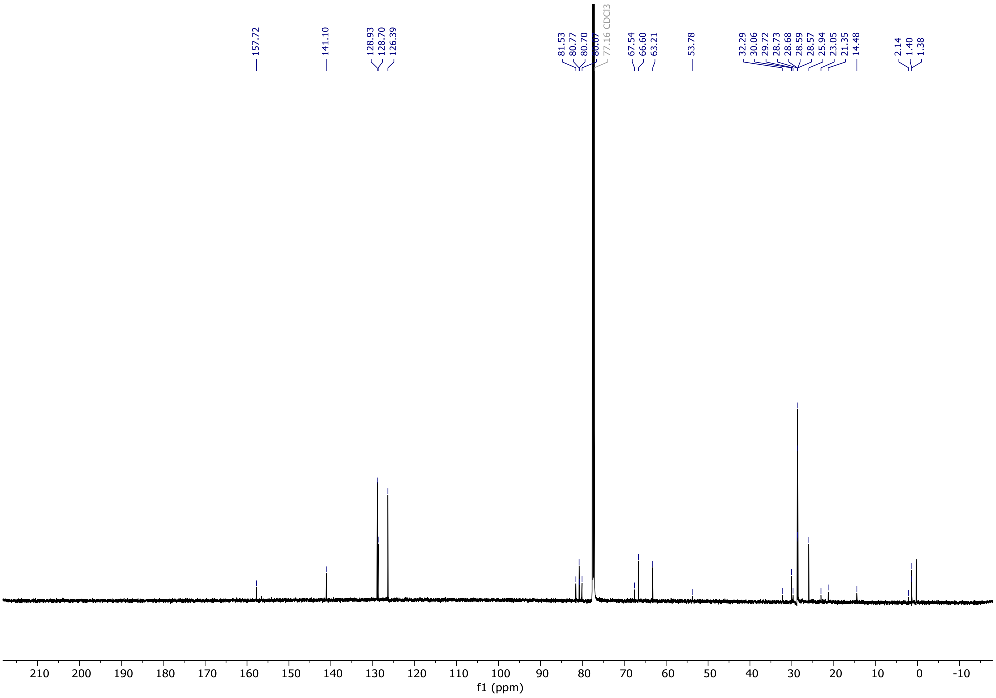

^{1}H NMR of **6g** (600 MHz, $CDCl_3$)

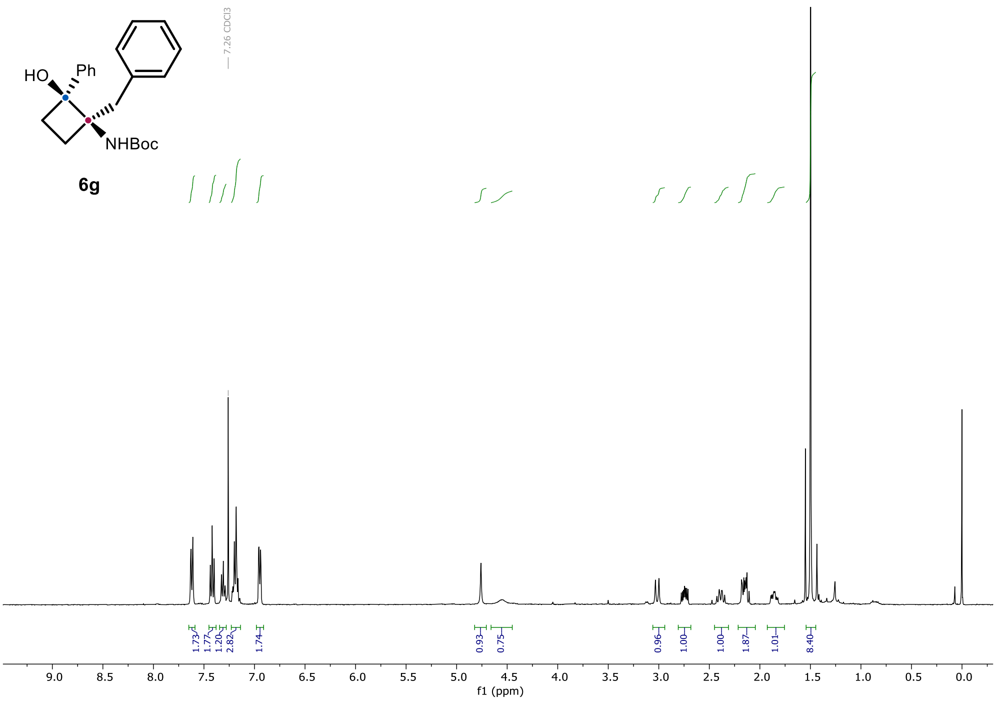

^{13}C NMR of **6g** (151 MHz, $CDCl_3$)

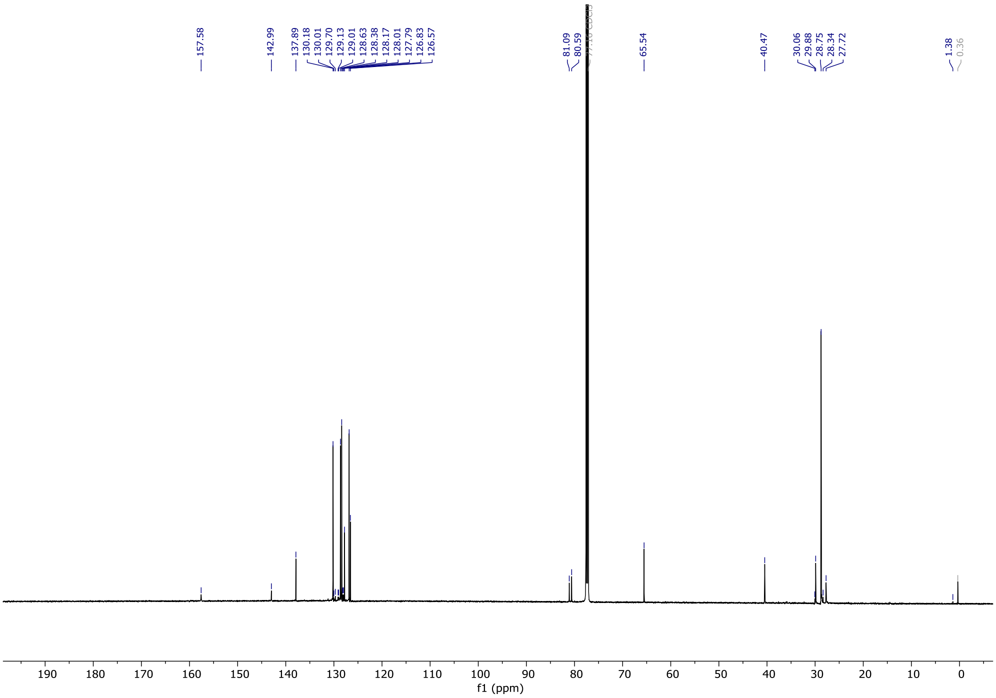

^{1}H NMR of **6h** (600 MHz, $CDCl_3$)

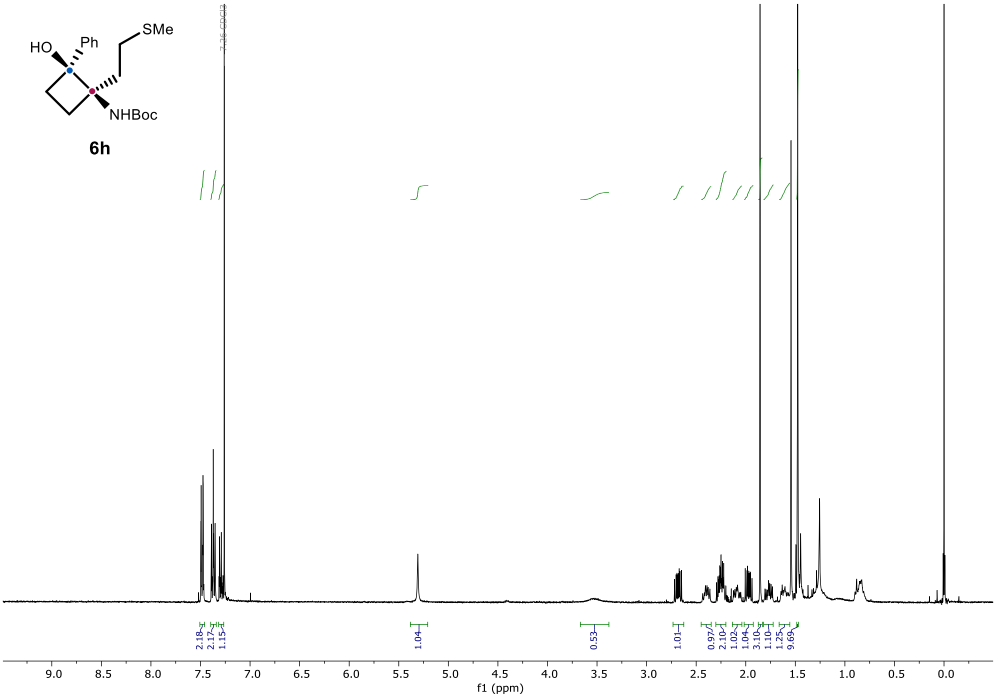

^{13}C NMR of **6h** (151 MHz, $CDCl_3$)

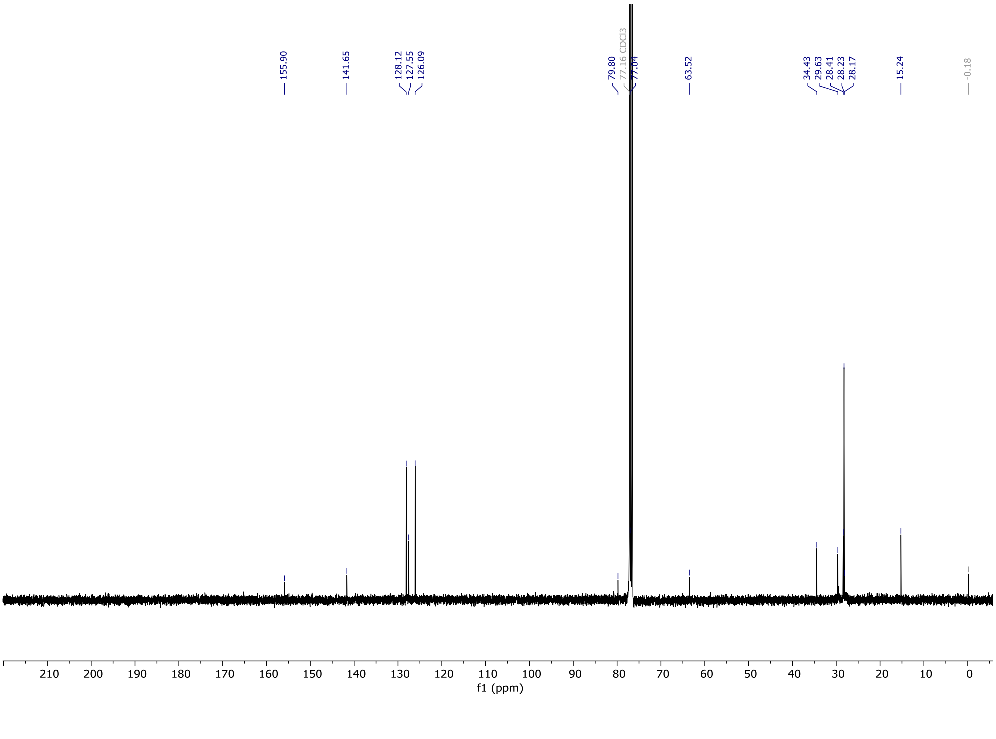

^{1}H NMR of **6i** (600 MHz, $CDCl_3$)

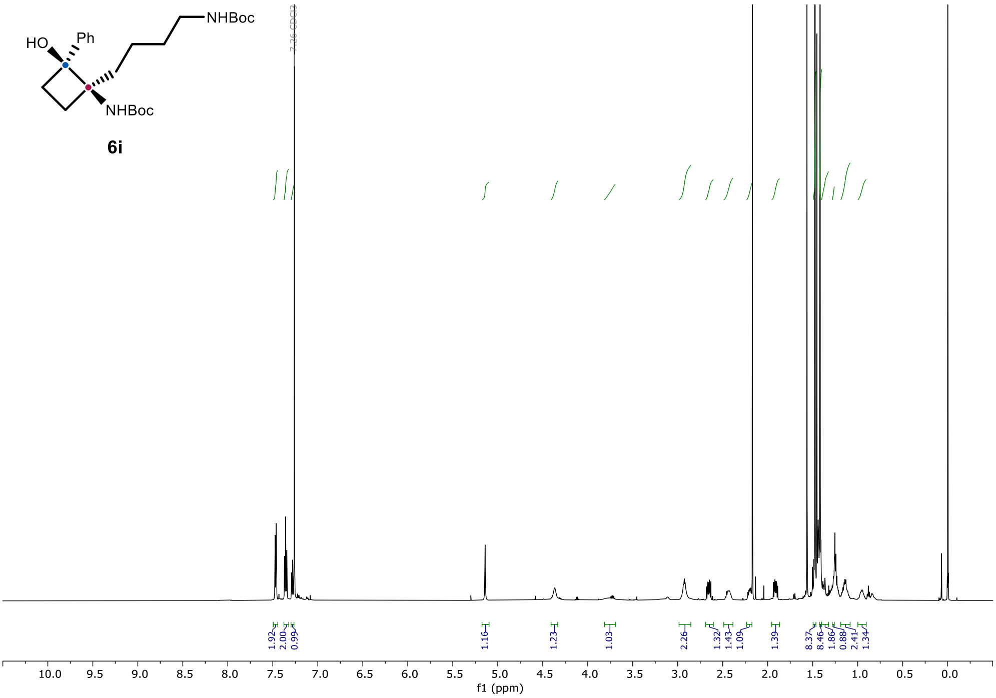

^{13}C NMR of **6i** (151 MHz, $CDCl_3$)

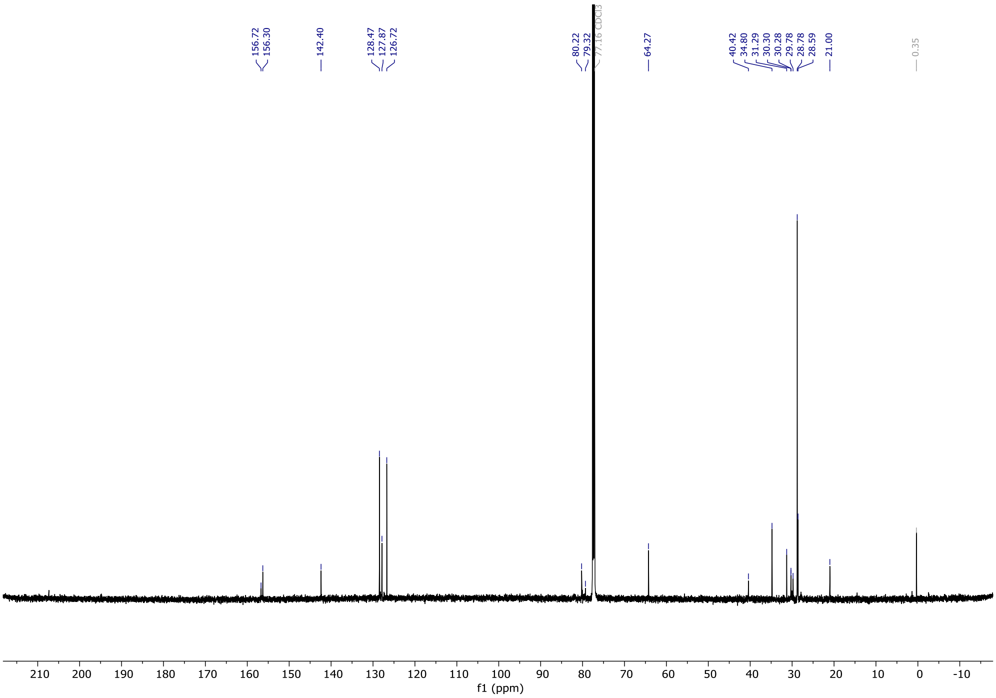

^{1}H NMR of **6j** (600 MHz, $CDCl_3$)

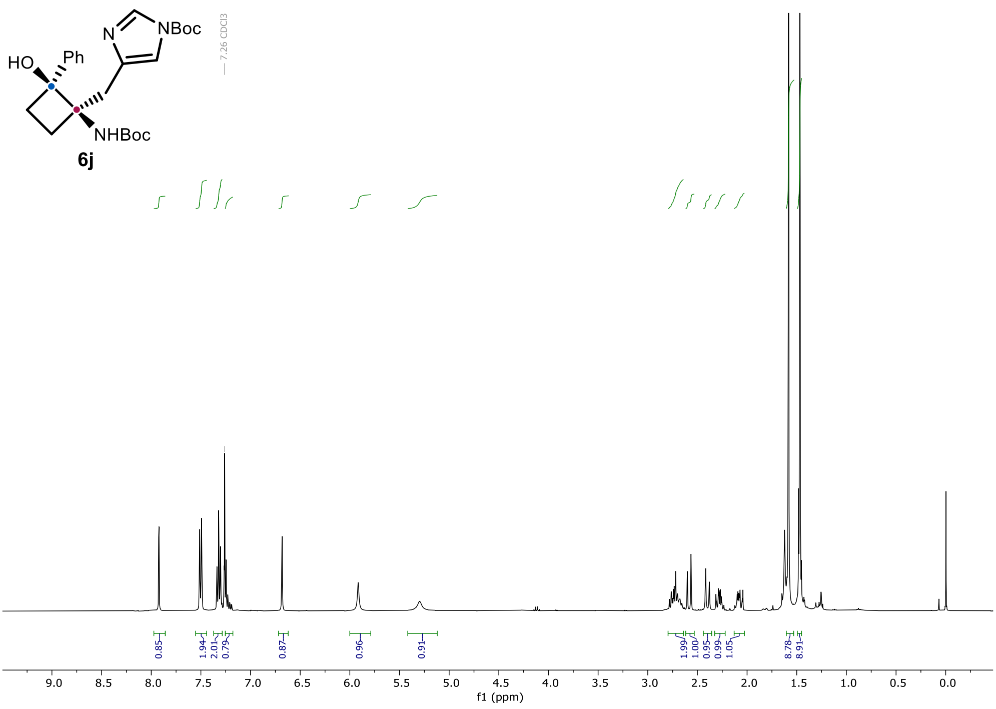

^{13}C NMR of **6j** (151 MHz, $CDCl_3$)

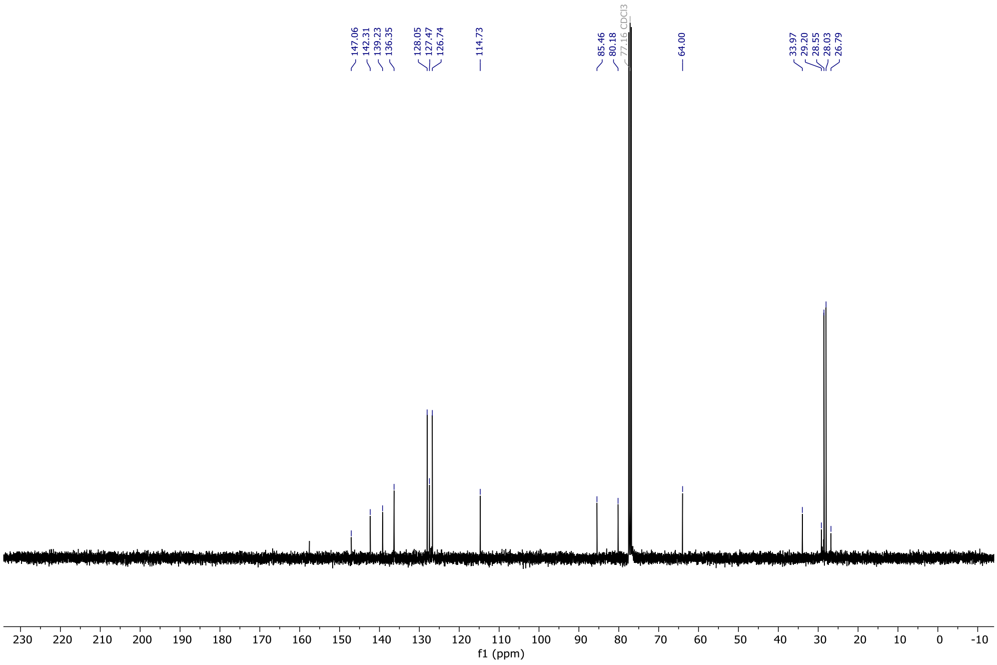

^{1}H NMR of **6k** (600 MHz, $CDCl_3$)

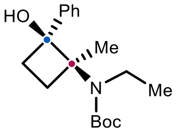

6k

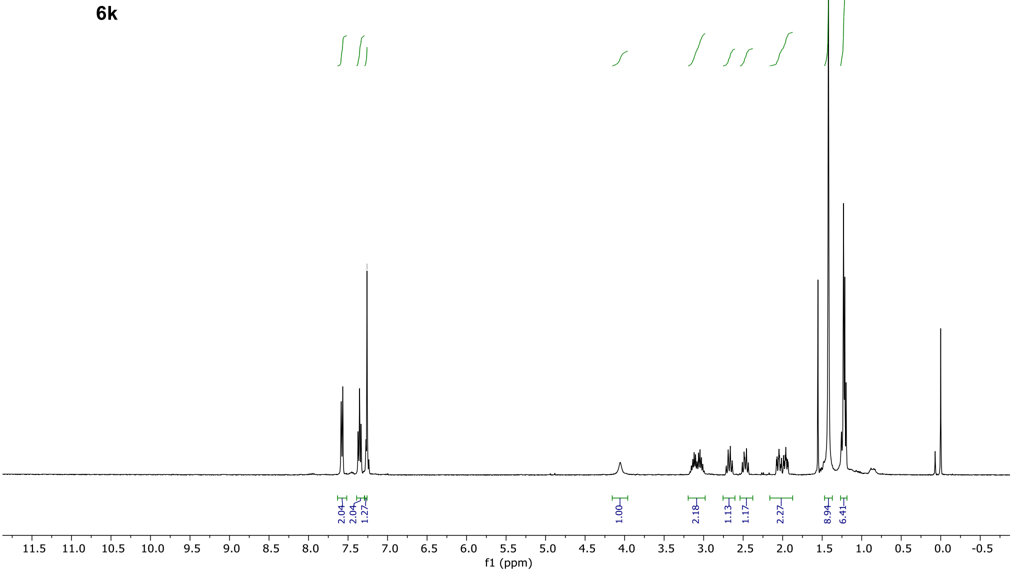

^{13}C NMR of **6k** (151 MHz, $CDCl_3$)

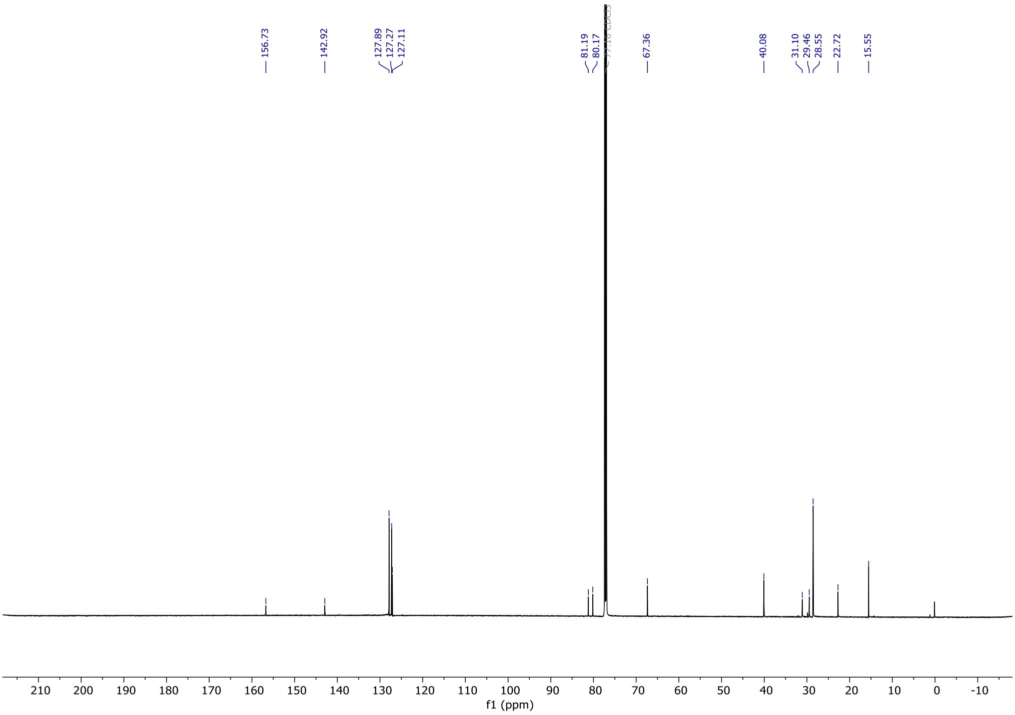

^{1}H NMR of **(1S,4S)-4** (600 MHz, CD_3OD)

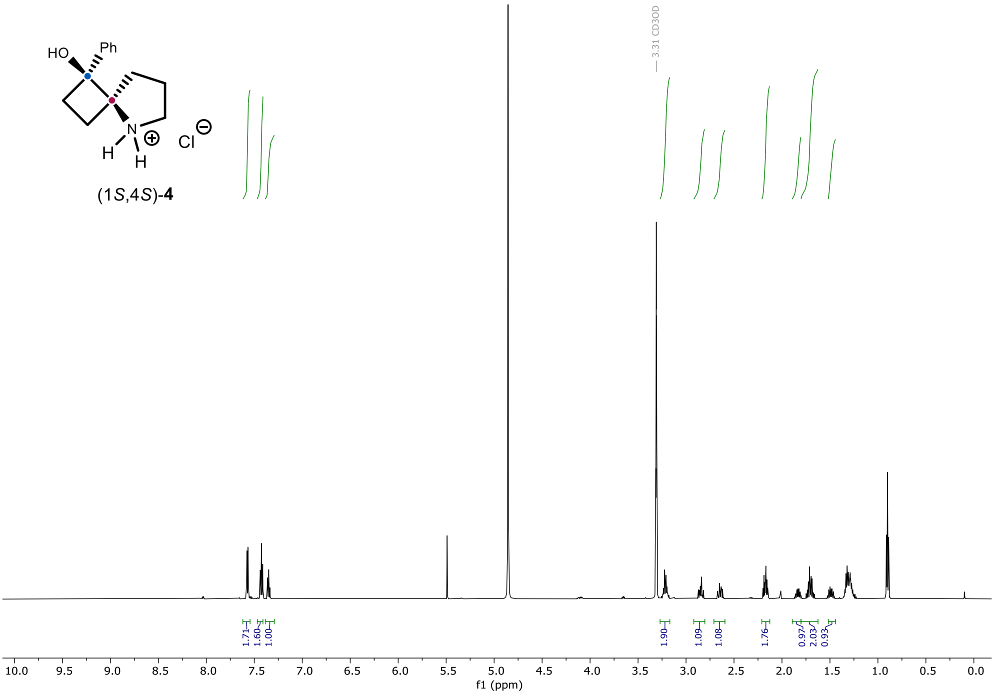

^{13}C NMR of **(1S,4S)-4** (151 MHz, CD_3OD)

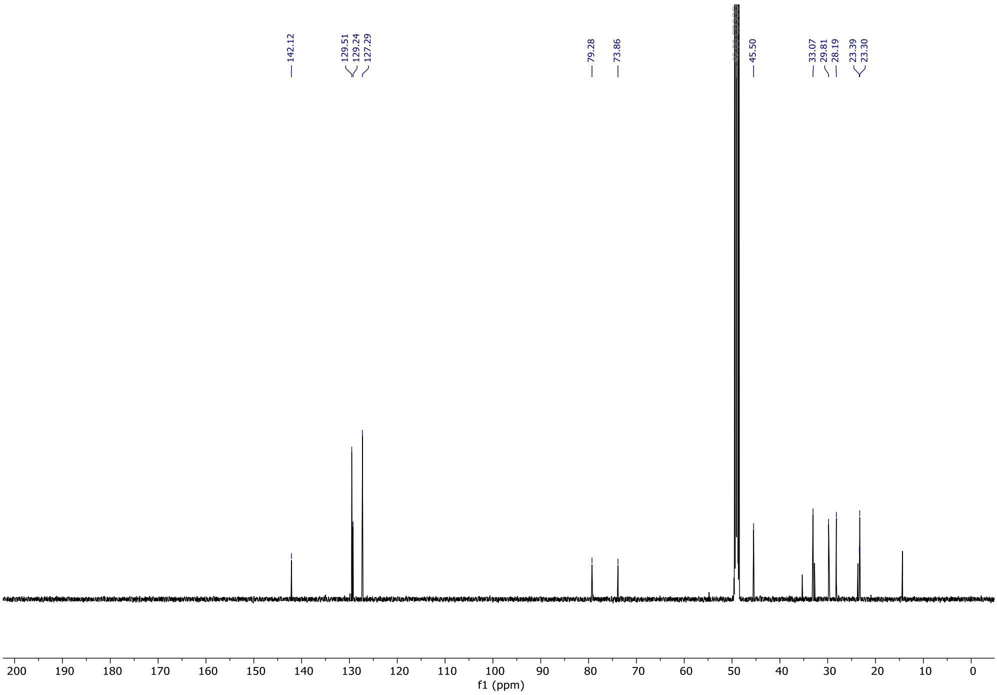

Supplement: Supplementary file 1 [file ja6c09924_si_001.pdf]
